# Synthesis of *m*,*n*‑Diaza[*n*]helicenes via Skeletal Editing of Indeno[2,1‑*c*]fluorene-5,8-diols

**DOI:** 10.1021/jacsau.5c00729

**Published:** 2025-10-03

**Authors:** Marina Degač, Lena Reininger, Hanna Schardax, Erik Andris, Lubomír Rulíšek, Ivana Císařová, Uwe Rinner, Timothée Cadart, Martin Kotora

**Affiliations:** † Department of Organic Chemistry, Faculty of Science, 37740 Charles University, Hlavova 8, 128 00 Praha 2, Czech Republic; ‡ Institute of Organic Chemistry and Biochemistry, Academy of Sciences of the Czech Republic, v.v.i. Flemingovo náměstí 2, 160 00 Praha 6, Czech Republic; § Department of Inorganic Chemistry, Faculty of Science, 37740 Charles University, Hlavova 8, 128 00 Praha 2, Czech Republic; ∥ Institute of Applied Chemistry, IMC Krems University of Applied Sciences, Piaristengasse 1, 3500 Krems, Austria; ⊥ Interns from Institute of Applied Chemistry, IMC Krems University of Applied Sciences, Piaristengasse 1, 3500 Krems, Austria

**Keywords:** skeletal editing, Schmidt reaction, diazahelicene, enantioselectivity, reaction mechanism

## Abstract

Skeletal editing of 5-membered carbocycles to 6-membered heteroaromatic compounds represents an attractive concept that would enable late-stage heteroarene construction. However, the current state of the art does not offer many synthetic strategies on how to achieve such a goal. One of those is based on a reaction of aromatic tertiary alcohols with sodium azide under acidic conditions, i.e., the Schmidt reaction. Herein, we present a hitherto unexplored double Schmidt reaction toward aromatic compounds possessing two pyridine rings. It is the first example of a conversion of 5,8-disubstituted indeno­[2,1-*c*]­fluorene-5,8-diols to *m*,*n*-diaza­[5]­helicenes in high yields. We also demonstrate that the regioisomeric ratio can be controlled, to a certain extent, by tuning conditions. Mechanistic investigation encompassing experimental as well as DFT calculations sheds light on the course of the reaction and provides a rationale for the observed regioselectivity and its control. In addition, structures of several diaza[5]­helicenes and intermediates were unequivocally confirmed by single crystal X-ray diffraction analyses. The double Schmidt rearrangement strategy was also applied for the transformation of enantioenriched [7]­helical indeno­[2,1-*c*]­fluorene-5,8-diols into the corresponding enantioenriched *m*,*n*-diaza­[7]­helicenes without significant loss of enantiopurity. Their structures were also unequivocally confirmed by single crystal X-ray diffraction analyses.

## Graphical abstract

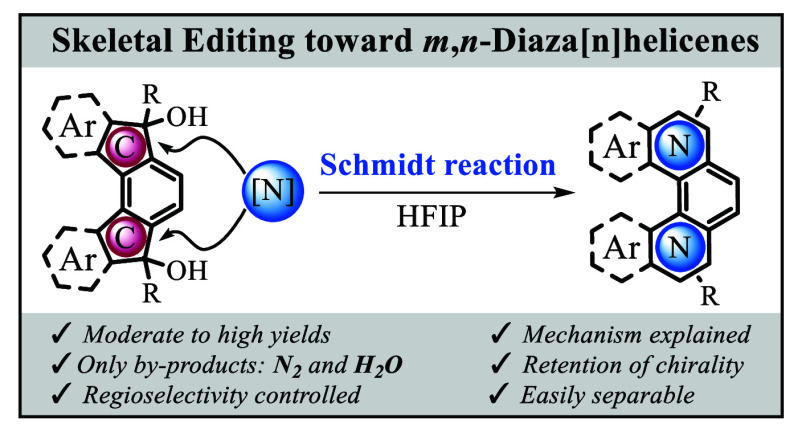

## Introduction

One of the fundamental goals of organic synthesis is to control the regioselectivity of newly built molecular fragments. Such objectives can be achieved by using different methodologies such as “molecular editing”, a new concept in organic synthesis to achieve structural modifications.^1^ Molecular editing encompasses processes such as insertion, deletion, or exchange of atoms directly and selectively. Many of currently existing molecular editing methodologies target a readily available functionality on the exterior of the substrate (peripheral molecular editing), leaving the basic skeleton untouched. However, an opportunity to reshape the core by interconverting (hetero)­cyclic subunits is synthetically highly tempting but challenging. Such a process can be viewed as “skeletal editing” as a subset of “molecular editing”.^2^ From a simplistic point of view, the aforementioned process can be viewed as the insertion of a building block unit into the ring, resulting in the ring expansion. In principle, such building blocks can comprise mono- or multi-atomic fragments.

In general, methodologies based on the insertion of new atoms (mainly C and N atoms) into ring systems are of the foremost importance because of their ability to change or modify chemical reactivity and properties.^2^ A great deal of interest is currently devoted to skeletal editing of the pyrrole functionality to heterocycles such as pyridines, pyrimidines, etc.^3–6^ Interestingly, expansion of PAH carbocycles to heterocycles has not received much attention yet, albeit two pioneering examples have been reported by Gu^7^ and Sakurai,^8^ to the best of our knowledge (*vide infra*). A recent report by Wei et al. on the conversion of substituted cyclopentenes to pyridine deserves to be mentioned as well.^9^ One class of compounds that would benefit from the development of such methodologies are regioisomeric hetero­[*n*]­helicenes. In principle, these could be accessible by ring expansion of suitable [*n*]­helical aromatic compounds, and such a process would allow the formation of various regioisomeric hetero­[*n*]­helicenes in a single synthetic procedure that would otherwise be difficult by using known strategies.

Helical scaffolds have fascinated chemists for several decades, mostly because of their twisted screw shape and inherent chirality. Special attention has been paid to helical aromatic compounds, i.e., [*n*]­helicenes and hetero­[*n*]­helicenes.^10^ Consequently, a plethora of diverse synthetic methodologies have been developed for their preparation. Introducing a heteroatom into the helical all-carbon scaffold allows changing and tuning their physical properties. Therefore, a class of *N*-atom embedded [*n*]­helicenes is attracting considerable interest. First, properties of the heteroaromatic system, such as electron-richness or electron-poorness, redox potentials, aromaticity, and reactivity toward electrophiles and nucleophiles are changed thanks to the electronegativity of the nitrogen atom. Second, the lone electron pair on the nitrogen atom is not involved in the π-conjugation, hence it is available for further interactions with other systems.^11^

There are currently two prevailing synthetic strategies toward aza- and polyazahelical aromatic compounds, depending on the relative positions of the nitrogen atoms within the elementary helical scaffold. The first strategy relies on the assembly of the *N*-embedded helical scaffold by using suitable *N*-containing building blocks, and it has been fruitfully applied in many instances. Photochemical cyclization of heteroaryl substituted ethenes belongs among such approaches, but other methods, such as catalytic cyclotrimerization,^10a,12^ etc. have been applied as well. The second strategy creates the pyridine ring via a ring expansion reaction of the fully carbon-based helical substrates with a suitable *N*-containing functional group. Although the second approach is synthetically more attractive (and falls into the modern concept of skeletal editing),^2^ it has been less explored and only a handful of examples are available. The methodology for the ring expansion can be based on Schmidt reaction^13^ (a reaction of ketones or alcohols with hydrazoic acid under acidic conditions, resulting in the formation of the pyridine ring) or Beckmann rearrangement (a reaction converting oximes to amides).

A typical example of these approaches includes Schmidt reaction of a [7]­helical alcohols to 10-substituted 9-aza[7]­helicenes^7^ (Scheme 1a) and a conversion of sumanenone via Beckmann rearrangement to a substituted azahomosumanene.^14,8^ It is noteworthy that in the case of symmetric secondary aromatic alcohols, e.g., fluorenol, the choice of the migrating carbon is inconsequential because the same product is formed regardless. However, for unsymmetrically substituted substrates, two different products could be formed. A rule of thumb says that the more electron-rich carbon migrates preferentially, as was demonstrated in the case of unsymmetrically substituted diarylmethanols^15^ and fluorenols.^16^

**Scheme 1:**
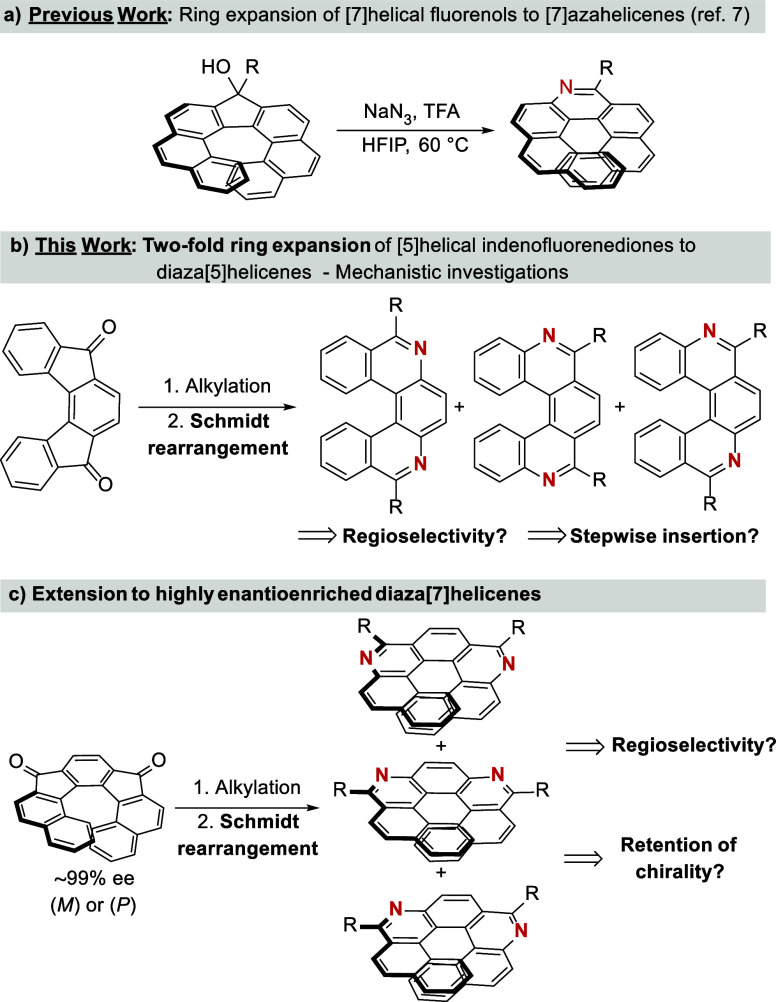
Skeletal Editing of Helical Aromatic Compounds toward Azahelicenes

In this report, we demonstrate that the transformation of [5]­helical and expanded [7]­helical indeno­[2,1-*c*]­fluorene-5,8-diols via double Schmidt rearrangement is feasible and provides high yields of regioisomeric mixtures of diaza[5]- and diaza[7]­helicenes, respectively (Scheme 1b). Moreover, we show that regioselectivity can be controlled by the use of appropriate acidic conditions. This latter methodology was successfully applied to enantioenriched [7]­helical indeno­[2,1-*c*]­fluorene-5,8-diones yielding the corresponding diaza[7]­helicenes without loss of enantiopurity (Scheme 1c). Therefore, these outcomes can pave new pathways for the synthesis and transformation of purely carbon aromatic frameworks to heterocyclic ones and open new horizons in the chemistry of polycyclic aromatic compounds.

## Results and Discussion

### Reaction Development

Our initial endeavors started with the preparation of variously substituted [5]­helical indeno­[2,1-*c*]­fluorene-5,8-diones by using the cyclotrimerization methodology previously reported by our group.^17^ Their subsequent reactions with different aryl and alkyl metals (organolithiums or Grignard reagents) gave rise to a set of 5,8-disubstituted indeno­[2,1-*c*]­fluorene-5,8-diols **1**. For details, see the SI. At the outset, a 2-fold Schmidt rearrangement of **1a** was tested under the previously used conditions utilizing sodium azide and trifluoroacetic acid in HFIP (Scheme 2).^8^ Although the reaction took place, it did not provide the expected diazahelicenes. Instead, two regioisomeric products of mono insertion, **2a** and **3a**, were formed in 70 and 17% isolated yields (64 and 21% with lithium azide, 0.1 mmol scale), respectively, while retaining the azide functional group attached to one carbon atom. Due to the migration of the more electron-rich bond, the major product **2a** is, in this case, less sterically hindered and therefore, a more thermodynamically stable product. The structure of **2a** was unequivocally confirmed by single crystal X-ray diffraction analysis (see the SI).

**Scheme 2:**
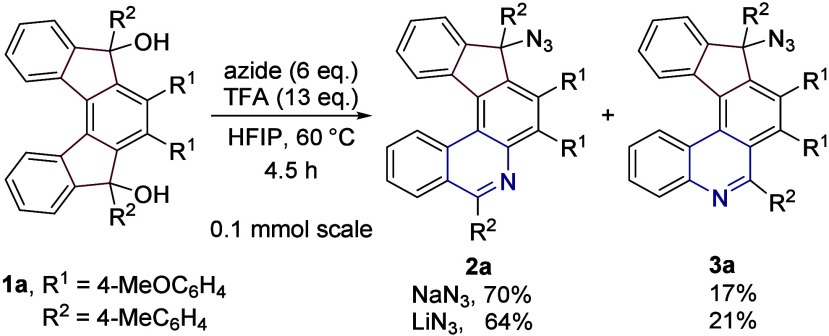
Reaction of **1a** with Azides in the Presence of Trifluoroacetic Acid

Despite not obtaining the expected products, the results indicated that the chosen strategy might be successful. To enable the protonation of the unreacted azide moiety and thus induce its rearrangement, we decided to use a stronger acid—benzenesulfonic acid. Its use in combination with sodium azide furnished a mixture of the desired diaza[5]­helicenes: **4a** (6,9-diaza[5]­helicene), **5a** (5,10-diaza[5]­helicene), and **6a** (5,9-diaza[5]­helicene) in 15, 23, and 40% isolated yields, respectively (Entry 1, Table 1), and no traces of **2a** or **3a** were observed. Structures of **4a** and **5a** were confirmed by single crystal X-ray diffraction analyses (see the SI). Then, the examination of the influence of various acids (namely their p*K*_a_) on the course of the reaction proceeded (Entries 2–6). It could be roughly concluded that the stronger the acid, the higher the preference for regioisomer **5a** and a higher yield, as it is nicely demonstrated in the case of TfOH or H_2_SO_4_ (Entries 4 and 6). On the other hand, the use of weaker acids (PhSO_3_H, MsOH, *p*-TsOH) resulted in the preferential formation of regioisomer **6a** (Entries 1–3). When HNO_3_ and HBr were used, the starting material was consumed, but complex reaction mixtures were obtained. In the case of H_3_PO_4_, the formation of diazide **7a** (a mixture of *syn* and *anti* isomers) was observed in 62% (their structures were confirmed by single crystal X-ray diffraction analyses), and the rest was again a complex mixture (Entry 7). Attempts to use other solvents such as MeCN, CHCl_3_, DMF, DMSO, and toluene with benzenesulfonic acid or triflic acid gave either no consumption of the starting material or, usually, lower yields of the desired products **4a**–**6a**. Interestingly, carrying out the reaction of **1a** in 2-propanol instead of HFIP, and in several other instances, the intermediate diazide **7a** (a mixture of *syn* and *anti* isomers) was isolated in 86% yield.

**Table 1:**
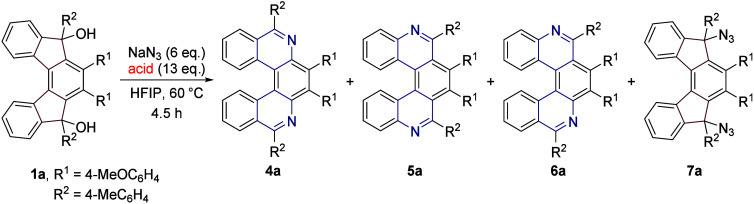
Schmidt Rearrangement of **1a** to **4a**–**6a** under Various Acidic Conditions

Entry	Acid^a^	p*K*_a_ ^b^	**4a** (%)^c^	**5a** (%)^c^	**6a** (%)^c^	**7a** (%)^c^ ^,^ ^d^	Mixed fraction (%)^e^	Yield (%)^f^	**4a**:**5a**:**6a**:**7a** ^g^
1	PhSO_3_H	–2.5	19	23	40	-	-	82	1:1.7:3:0
2	MsOH	–2.0	16	19	24	-	14	73	1:1.3:3:0
3	*p*-TsOH	–2.8	19	21	41	-	-	81	1:1.5:3:0
4	H_2_SO_4_	–3	traces	48	-	-	20	68	1:12:3:0
5	HCl	–8	20	23	45	-	-	88	1:1.2:3.5:0
6	TfOH^h^	–13	4	68	-	-	25	97	1:11:1.5:0
7	H_3_PO_4_	2.1	-	-	-	62	-	-	0:0:0:100

a Before handling azides, read: Treitler, D. S. ; Leung, S. How Dangerous Is Too Dangerous? A Perspective on Azide Chemistry. J. Org. Chem. 2022, 87, 11293–11295 36052475 10.1021/acs.joc.2c01402.

b p*K*_a_ in water. Data are taken from Ionization Constants of Organic Acids–MSU Chemistry. https://www2.chemistry.msu.edu/faculty/reusch/virttxtjml/acidity2.htm.

c Isolated yields.

d Although organic azides are known to be generally unstable compounds, we have not observed any problems with their thermal instability during their isolation and storage.

e Mixtures of regioisomers.

f Combined isolated yields.

g Approximate ratios calculated from ^1^H NMR of the respective crude reaction mixtures.

h 11 eq.

### Scope of the Reaction

With the optimized reaction conditions in hand, we decided to investigate the scope of the reaction with respect to different substituents, R^1^-R^3^. A set of various tertiary alcohols possessing the indeno­[2,1-*c*]­fluorene-5,8-diol scaffold **1** were exposed to the action of a mixture of sodium azide, TfOH, and HFIP on a small or a larger scale which led to the desired products in moderate to high yields (Scheme 3). As mentioned previously, when **1a** was subjected to the general conditions, **4a** was isolated in 4% yield, **5a** in 68% yield, and **6a** was obtained as a mixture with **5a**. In the case of *o*-tolyl substituted indeno­[2,1-*c*]­fluorene-5,8-diols **1b**, only two regioisomers were obtained after the rearrangement: **4b** in 14% and **6b** in 16% isolated yields. Because of the proximity of *o*-tolyl and *p*-methoxyphenyl substituents and resulting restricted bond rotation, compound **6b** was characterized as a mixture of rotamers. A similar result was observed with substrate **1c** bearing *n*-butyl substituents. **4c** and **6c** were isolated in 10 and 30% yield, respectively, while **5c** was not detected even in the reaction mixture. However, we cannot exclude the existence of other reaction pathways that do not lead to the desired products, thus diminishing the overall yield. Substrates bearing *p*-(trifluoromethyl)­phenyl substituents **1d** and **1e** gave similar results. In both instances, **4d** (**4e**) and **5d** (**5e**) were isolated as analytically pure substances, while **6d** (**6e**) was obtained as a mixture with **5d** (**5e**). When the optimized reaction conditions were tested on **1f**, we isolated only regioisomer **5f** in 46% yield. In the case of lower yields, there is a possibility that the rest contained some products, but they were not detected in the NMR due to the complexity of the isolated fractions. Notably, when the methoxy substituents were added to the outer benzene rings, we observed the formation of only the major regioisomer, **5g**, in 82% isolated yield. This correlates to the explanation for the Schmidt rearrangement that the more electron-rich bond migrates. It should be noted that the Schmidt reaction of the secondary indeno­[2,1-*c*]­fluorene-5,8-diol was attempted as well, but a complex reaction mixture was obtained. Lastly, compound **5a** was converted to azonium salt, **5a** ^**+**^, in a reaction with methyl iodide in toluene. Nevertheless, all attempts (under stronger methylating conditions) to synthesize the diazonium salt gave either complex reaction mixtures or decomposition of the product during purification.

**Scheme 3:**
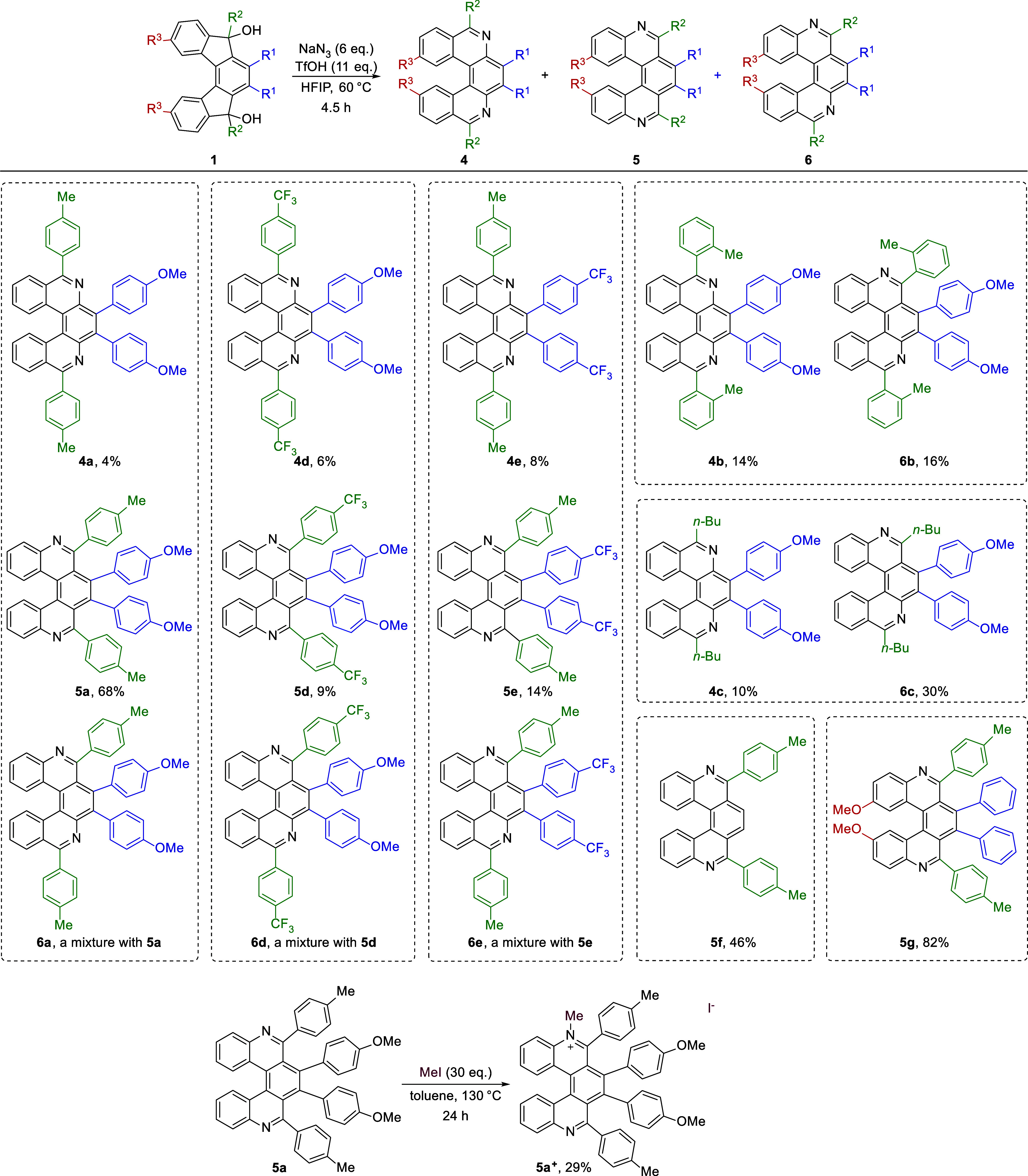
Scope of the Schmidt Reaction with Differently Substituted Diols **1a**–**1g**

### Mechanistic Investigation: A Detailed Quantum Mechanical Investigation

To explain in detail our experimental findings, we calculated possible reaction pathways by quantum chemical (DFT-D3) methods in HFIP as the implicit solvent, represented by the SMD (Solvation Model based on Density)^18^ and COSMO-RS (Conductor like Screening MOdel for Real Solvents)^19^ methods. We chose a system with unsubstituted phenyl groups (**I**), reacting with CF_3_SO_3_H with the aim of understanding the regioselectivity of the Schmidt rearrangements.

Initial structure optimizations were carried out employing Gaussian 16 C.01 program,^20^ with B3LYP^21–24^ -D3BJ^25,26^ DFT functional and def2-SVP basis set in 2-propanol, using SMD model. For all structures, except for the simplest chemical species - H_2_O, CF_3_SO_3_H, CF_3_CO_2_H, HN_3_, N_2_, and corresponding anions - we also performed conformational search using the CREST program^27^ using GFN-2 ^28^ semiempirical method implemented in XTB program,^29^ with energies recalculated (also during the conformer search procedure) at BP86-D3_Rezac_ ^31,32^ /dgauss-dzvp^33,34^ level and COSMO-RS solvation model (ε = 80; BP_TZVPD_FINE_22.ctd parametrization from BIOVIA COSMO*Therm* (Dassault Systèmes) program; dispersion parameters, a_1_ = 0.7182, s_8_ = 3.2176, a_2_ = 3.8572 ^35^). The three energetically lowest structures from this (CREST) conformational search were then calculated at the B3LYP-D3BJ/def2-TZVP//def2-SVP level, and only the most stable conformer was considered further. For final energies, we further reoptimized the geometry at the B3LYP-D3BJ/def2-SVP level in HFIP (SMD model, HFIP parameters taken from ref.^36^) and performed frequency calculations on these equilibrium geometries. Gibbs energy correction (*G*_trans+rot+vib_) was then computed at this level. To choose the DFT method for the calculation of the final single point energies, we performed benchmark calculations on small model systems (Figure S25). To this aim, we compared B3LYP-D3BJ/def2TZVPD, ωB97X-D^37^/def2-TZVPD DFT energies, with the reference CCSD­(T)/aug-cc-pVTZ energies. A better, almost *quantitative* agreement with the CCSD­(T) reference has been found for the *E*_ωB97X‑D_ computed values. The main difference between the two functionals is that B3LYP predicts the charged intermediates to be more stable relative to uncharged ones. Therefore, we decided to use the latter (ωB97X-D) functional for the final single point energies. Solvation energies were calculated with the *COSMOtherm* program at the BP86-D3/def2TZVPD level with the FINE cavity^38^ without reoptimization in the gas phase (Δ*G*_COSMO‑RS_). The final Gibbs free energies were calculated as *G*_ωB97X‑D_ = *G*_trans+rot+vib_ + *E*_ωB97X‑D_ + Δ*G*_COSMO‑RS_. The XYZ coordinates for the reported structures can be found in the SI. The whole calculated reaction pathway, including possible stereoisomers, is given in Figure 1, whereas Figure S26 shows the selected key reaction intermediates and products.

**Figure 1:**
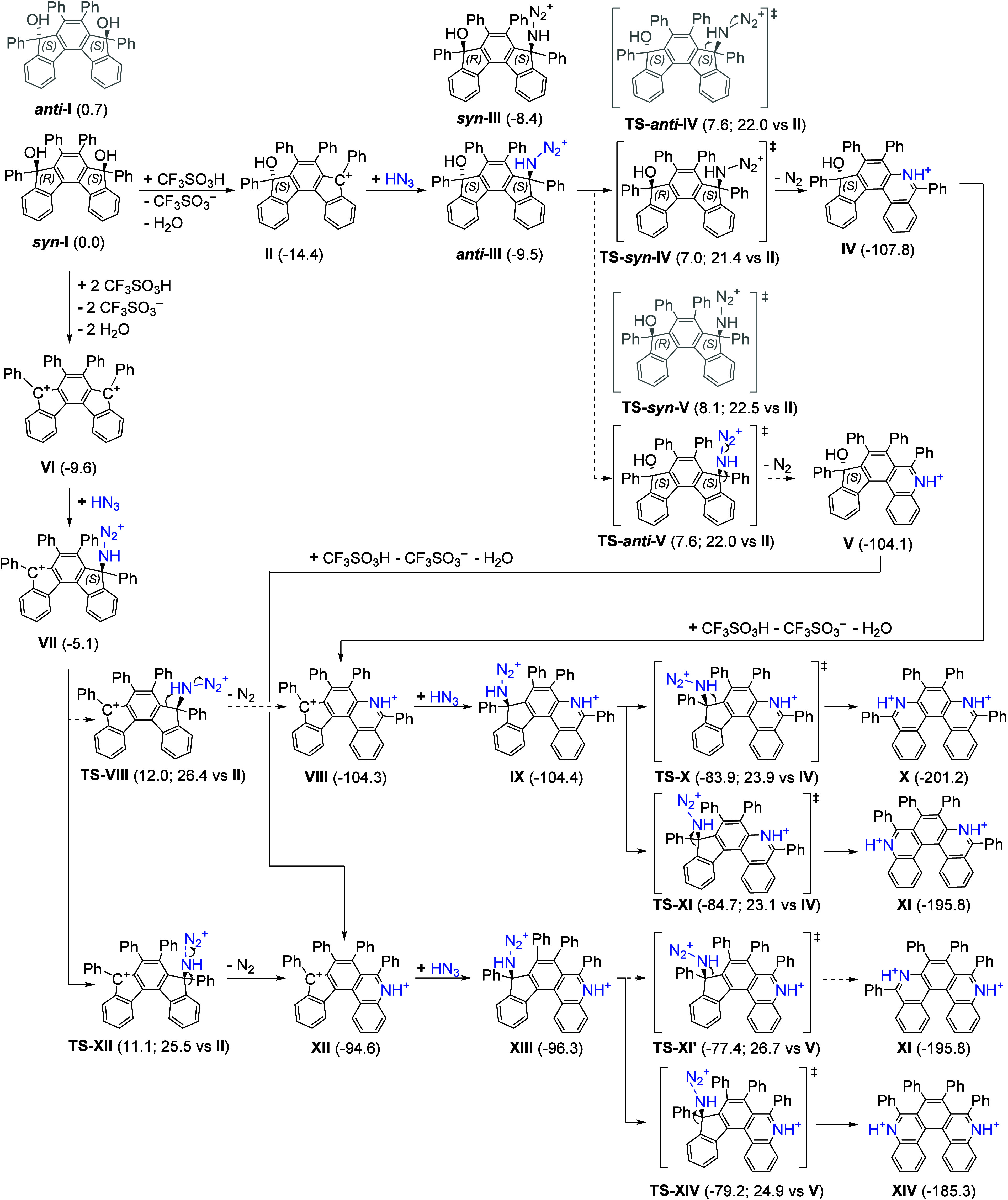
Relative Gibbs free energies for the reaction of **I** with CF_3_SO_3_H calculated at ωB97X-D/def2-TZVPD//B3DLYP-D3JB/def2-SVP level in HFIP (SMD optimization, COSMO-RS single point energies) at *T* = 298.15 K and *c* = 1 M. Alternative stereoisomers are depicted in gray. For transition states, we also list energy relative to the preceding most stable intermediate. The nonpreferred pathways are indicated by dashed arrows. Note that even though reaction energies indicate the preference for a monocationic intermediate, these can shift based on the exact conditions and strength of the actual acid used (cf. Figure S27 for the same reaction network with CF_3_CO_2_H).

The initial diol **I** can, depending on the acidity of the reaction environment, undergo one or two eliminations of OH groups to yield singly charged carbocation **II** and doubly charged carbocation **VI**, respectively. The initial state of the reaction seems to correspond to carbocation **II**, whose energy is −14.4 kcal mol^–1^ with respect to reactants (Figure 1). On the other hand, the energy of the doubly charged cation is −9.6 kcal mol^–1^. We expect that, based on the strength of the acid and water content, the lack of which would shift the equilibrium toward **VI**, as 2 water molecules are released when it is formed compared to one molecule, when **II** is formed, the dicationic intermediates might also play a role in the reaction. However, for weaker acids, such as CF_3_CO_2_H, these dicationic intermediates are not accessible at all (Figure S27). Thus, we will consider both of these pathways (monocationic—relevant for weaker acids and dicationic—relevant for stronger acids). In the monocationic pathway, after the formation of the cation, the reaction proceeds with the addition of azoimide to form key intermediates **III** and **VII**, which can further release N_2_ (Figure S26). In this step, the N_2_ molecule leaves in the opposite direction to the newly formed C–N bond (Figure S26). Therefore, if the C–N bond was formed with the peripheral Ph ring (position B in intermediate **III**), the leaving N_2_ would clash with one of the phenyl rings of the central part of the molecule. This makes position A favored by default for the C–N bond formation by 0.6 kcal mol^–1^ (**TS-** *anti* **-V** and **TS-** *syn* **-IV**; Figure 1). After the N_2_ release takes place, the remaining OH group can be, again, substituted by HN_3_. However, in this case, the central ring is already slightly deactivated by the presence of NH^+^ group in the para position (but not in direct conjugation) to position A and this makes the second step nonselective (calculated energy difference between **TS-X** and **TS-XI** is 0.8 kcal mol^–1^, slightly preferring the unsymmetrical product **XI**, corresponding to “**6**” in the experimental compound numbering, with respect to product **X.** The barrier to form these final products is also higher than that of the first step (23 vs 21 kcal mol^–1^), which explains why the reaction can be stopped after the first step under milder conditions.

To explain the reactivity of doubly charged intermediate **VII**, we need to invoke electronic effects. These were not important in the case of **III,** because the central and peripheral rings are electronically quite similar. In **VII**, however, even though steric effects would still favor attacking position A, this position is strongly deactivated for electrophilic attacks by the presence of a positive charge in the aromatic system and the difference in corresponding transition state energies is 0.9 kcal mol^–1^ in favor of the sterically hindered TS. Energy of the doubly charged transition state **TS-XII** is 4 kcal mol^–1^ higher than that of **TS-** *syn* **-IV**, but it might be accessible when a large excess of acid and low concentration of water is present in the environment, especially given the fact that ωB97X-D function underestimates stability of dicationic intermediates by 2 kcal mol^–1^ with respect to the CCSD­(T) calculations (Figure S27). Therefore, after N_2_ elimination and subsequent addition of HN_3_ to the resulting cation, we obtain intermediate **XIII**. This intermediate, unlike **IX**, has its position A deactivated by the conjugation of this position with the protonated nitrogen center. The second attack thus also proceeds at position B with a predicted barrier difference of 1.8 kcal mol^–1^, resulting in product **XIV** (corresponding to “**5**” isomer in the experimental numbering). Therefore, the regioselectivity of the second ring expansion step is given by the outcome of the first step, which makes the reaction much “cleaner” than would be expected from statistics if these steps were uncorrelated. This is consistent with the reactivity of **XIII**-like **3a** from Scheme 4, which dominantly forms the “**5**” isomer (**XIV**-like), unlike **IX**-like **2a**, which forms a mixture of isomers **4** and **6** (**X** and **XI**-like, respectively).

**Scheme 4:**
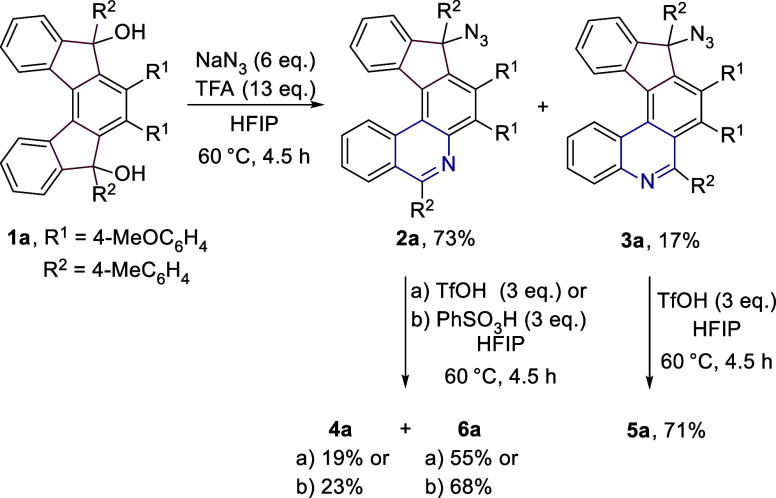
Stepwise Schmidt Reaction of **1a** to **4a**, **5a**, and **6a**

To sum up, the regioselectivity of the first Schmidt rearrangement in “weaker” acids is governed by the TS geometry, which favors the C–N to form a bond with the central ring, because the leaving nitrogen molecule in such a case does not suffer steric clashes with phenyl substituents on the central ring. However, if the molecule is doubly dehydrated to form a dication, the middle ring is so electron-poor that the C–N bond is formed with the outer ring. The outcome of the second step, again, depends on the interplay of steric and electronic factors, although the electronic factors ultimately prevail, and in the second step, the C–N bond is mostly formed with the peripheral ring.

### Stepwise Insertion

In order to gain further experimental evidence for the proposed course of the reaction, we decided to study a stepwise rearrangement (Scheme 4). For that purpose, indeno­[2,1-*c*]­fluorene-5,8-diol **1a** was reacted with sodium azide and trifluoroacetic acid in HFIP to give a mixture of **2a** and **3a**, and the corresponding regioisomers were obtained in 73 and 21% (0.3 mmol scale) isolated yields, respectively. The major regioisomer **2a** was treated with TfOH in HFIP, yielding a mixture of **4a** (19%), and **6a** (55%). The same reaction carried out in the presence of PhSO_3_H provided a mixture of **4a** and **6a** in 23 and 68% yields. Formation of **5a** was not detected. On the other hand, rearrangement of **3a** induced by treatment with TfOH furnished selectively **5a** in 71% yield. The formation of **4a** and **6a** was not detected.

These results clearly indicate that the course of the reaction under the weaker acidic conditions, i.e., the formation of the respective regioisomeric diazahelicenes is dictated by the course of the first ring expansion and can be generalized as follows: a) formation of regioisomer **2** is followed by the second ring expansion, which gives rise to regioisomers **4** and **6**, b) whereas formation of regioisomer **3** is followed by formation of regioisomer **5**. The course of the reaction and preferential regioisomer formation are fully supported by the aforementioned DFT calculations.

However, the proposed course of the reaction under stronger acidic conditions (TfOH) is slightly different. To provide further support for the suggested mechanism, we have performed additional experiments to confirm the formation of carbocations. Due to their electron-deficient nature, carbocations typically exhibit distinct absorption bands;^39^ therefore, we measured absorption spectra under various conditions to elucidate the formation and stability of the carbocations **1a** ^**+**^ and **1a** ^**2+**^. For this experiment, HFIP was used as the solvent since it has proved to be of great importance for the successful course of the rearrangement (see the SI). Because of its polarity, it has a stabilizing effect on carbocations.^40–43^ The absorption spectrum was recorded for **1a**, a mixture of **1a** and benzenesulfonic acid (a weaker acid), and a mixture of **1a** with triflic acid (a stronger acid) (Scheme 5). The spectrum for **1a** with benzenesulfonic acid (the black line) matched that of **1a** alone (the yellow line). However, the mixture of **1a** with triflic acid had different spectral features, including two new broad signals approximately at 515 and 585 nm (the purple line). The calculated values (employing the efficient semiempirical ZINDO method) for **1a** ^**+**^ and **1a** ^**2+**^ were 464 and 598, 449, and 600 nm, respectively (see Figure S6; Figure S8 depicts corresponding TD-DFT/CAM-B3LYP results). Considering very good agreement between computed and experimental spectra for **1a** (c.f. Figures S6–S8), giving us confidence in computed ZINDO values, we conclude that the experimental spectra indicate the presence of **1a** ^**+**^ and possibly also an admixture of **1a** ^**2+**^ or other carbocations, upon addition of triflic acid. To further confirm the carbocation formation, additional characterization using ^13^C NMR spectroscopy was performed. The spectrum of **1a** with triflic acid in HFIP was measured. The signal for quaternary carbon belonging to the tertiary alcohol of **1a** completely disappeared, and three additional signals, corresponding to the carbocation region around 200 ppm, were observed (see the SI). These signals fall into the same region as recently recorded values for stabilized carbocations.^39^ However, in the absence of nucleophiles, the carbocation species undergoes degradation over time. Taken together, these results show the presence of different carbocations in the mixture with TfOH, consistent with the proposed mechanism.

**Scheme 5:**
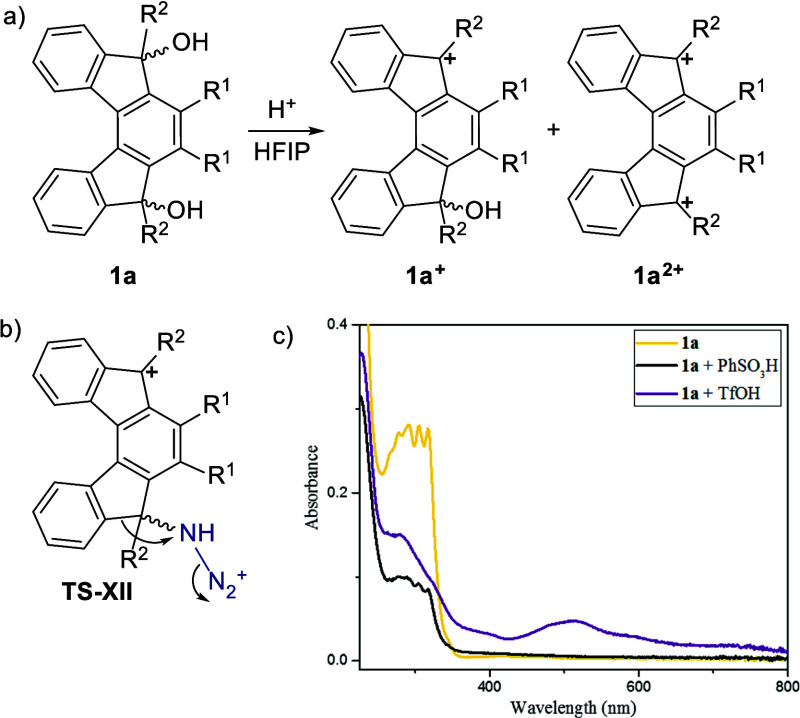
(a) Formation of Carbocations under Acidic Conditions; (b) Leading Intermediate toward Product **5**; (c) Absorption Spectra (*c* = 10^–5^ M) of **1a** (Yellow) in HFIP with Benzenesulfonic Acid (Black) and Triflic Acid (Purple)^a^

a The computed spectra at various levels of theory are depicted in the SI (Figures S7–S8).

On the other hand, access to diazide **7a** by a reaction of **1a** with sodium azide and triflic acid in 2-propanol (Table S2, Entry 25) opens an opportunity to attempt its thermal rearrangement since it has been shown that organic azides undergo thermal decomposition at elevated temperatures.^44^ After several trials, it was apparent that heating to 200 °C in the microwave reactor for 3 h (Scheme 6) provided a mixture of all regioisomers **4a**, **5a**, and **6a** in an approximate ratio of 5.5:3.5:1. The major product **4a** was isolated in 50% yield (on 0.1 mmol scale). Gratifyingly, the thermal rearrangement enabled us to steer the reaction course toward the least sterically encumbered regioisomer, 6,9-diaza[5]­helicene, which has always been formed as the minor product under acidic conditions. It is presumed that in this instance, the course of the reaction proceeds via a nitrene intermediate.^45^

**Scheme 6:**
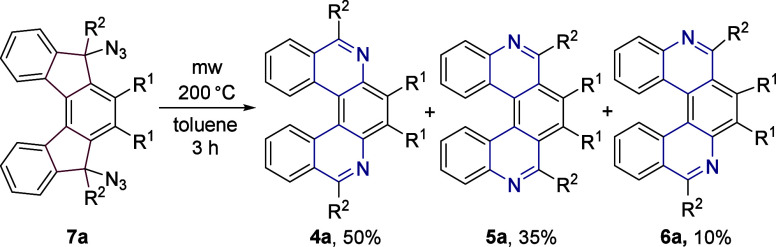
Thermally Induced Rearrangement of **7a**

### Extension to Synthesis of Enantioenriched *m*,*n*-Diaza­[7]­helicenes

In the next step, we synthesized enantioenriched (*M*)- and (*P*)-[7]­helical indeno­[2,1-*c*]­fluorene-5,8-diones **S6** according to our previously published procedure by using Rh­(COD)_2_(BF_4_) (5 mol %) and (*R*) or (*S*)-SEGPHOS (6 mol %) catalytic systems, followed by PCC oxidation.^46^ Both enantiomers were obtained with enantiopurity ∼99% ee (see the SI). Then they were converted to (*M*)- and (*P*)-**8** by arylation with *p-*tolyllithium generated *in situ*. Our initial attempts to use TfOH to induce the rearrangement were not met with success, instead, a black tarry complex reaction mixture was obtained. However, switching to a weaker acid, PhSO_3_H, turned out to be successful, and both enantiomeric diols were converted to the corresponding mixtures of diaza[7]­helicenes (Scheme 7). Both (*M*)- and (*P*)-*m*,*n*-diaza­[7]­helicenes **9–11** were obtained in high combined yields of 73% and 72%. In all cases, the reaction proceeded with minimal erosion of enantiopurity. The structures of all three regioisomeric *m*,*n*-diaza­[7]­helicenes were unequivocally confirmed by single crystal X-ray diffraction analyses (see the SI). It should be noted that the products can be separated by simple column chromatography. (For racemization barriers, see the SI, Ch. 8).

**Scheme 7:**
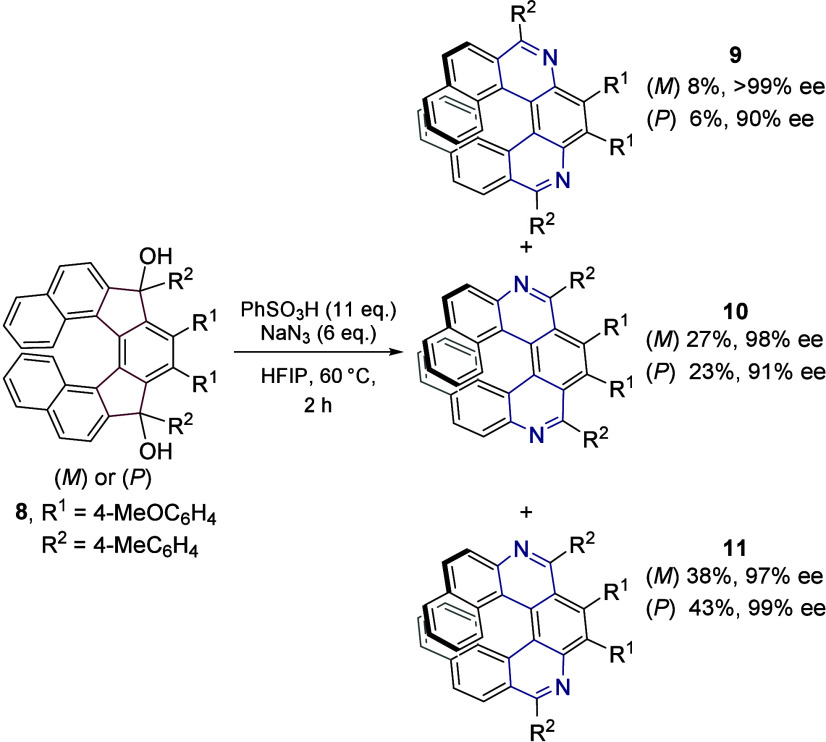
Conversion of (*M*)- and (*P*)-**8** to the Corresponding (*M*)- and (*P*)-**9**–**11** Diaza[7]­helicenes

### X-ray Diffraction Analyses

Single crystal X-ray diffraction analyses were recorded for *m*,*n*-diaza­[5] and [7]­helicenes **4a**, **5a**, **5d**, **6c**, **9**, **10**, and **11**. Their essential characteristics are given in Tables S7–S10 in the Supporting Information (Ch. 7). In addition, structures of azide and diazides **2a**, **7a**-*anti*, and **7a**-*syn* were determined (for details see the SI, Ch. 7). Representative X-ray structures of diaza[5]­helicenes (**4a**, **5a**, and **6c**) and diaza[7]­helicenes (**9**, **10**, and **11**) are displayed in Figures 2 and 3, respectively.

**Figure 2:**
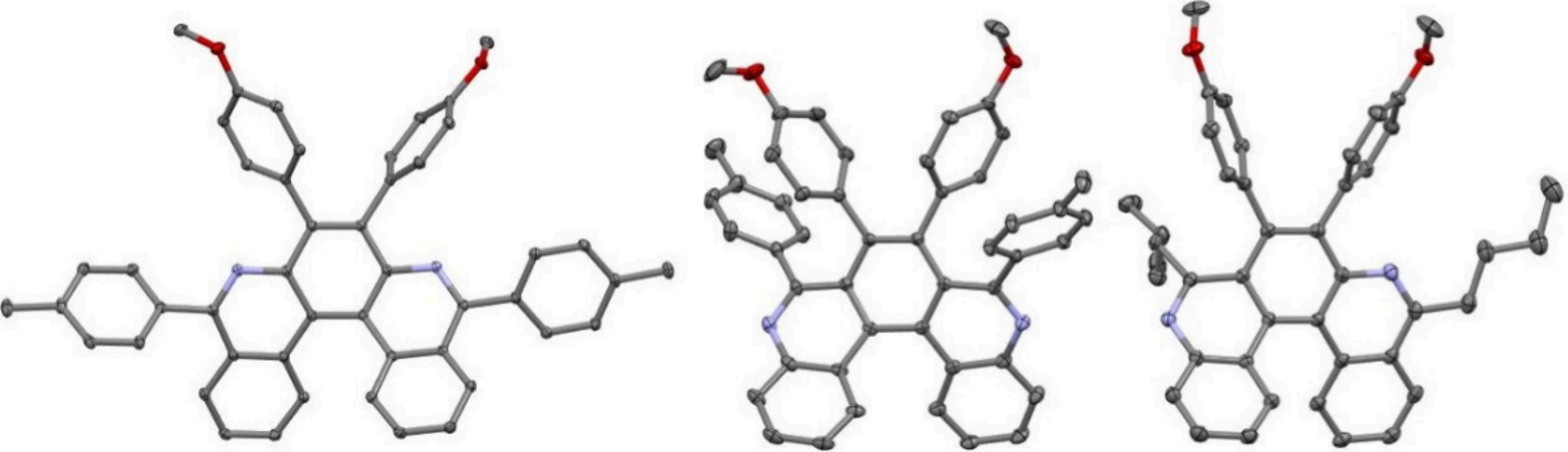
ORTEP drawings of **4a**, **5a** and **6c**. Ellipsoids are drawn with 30% probability.

**Figure 3:**
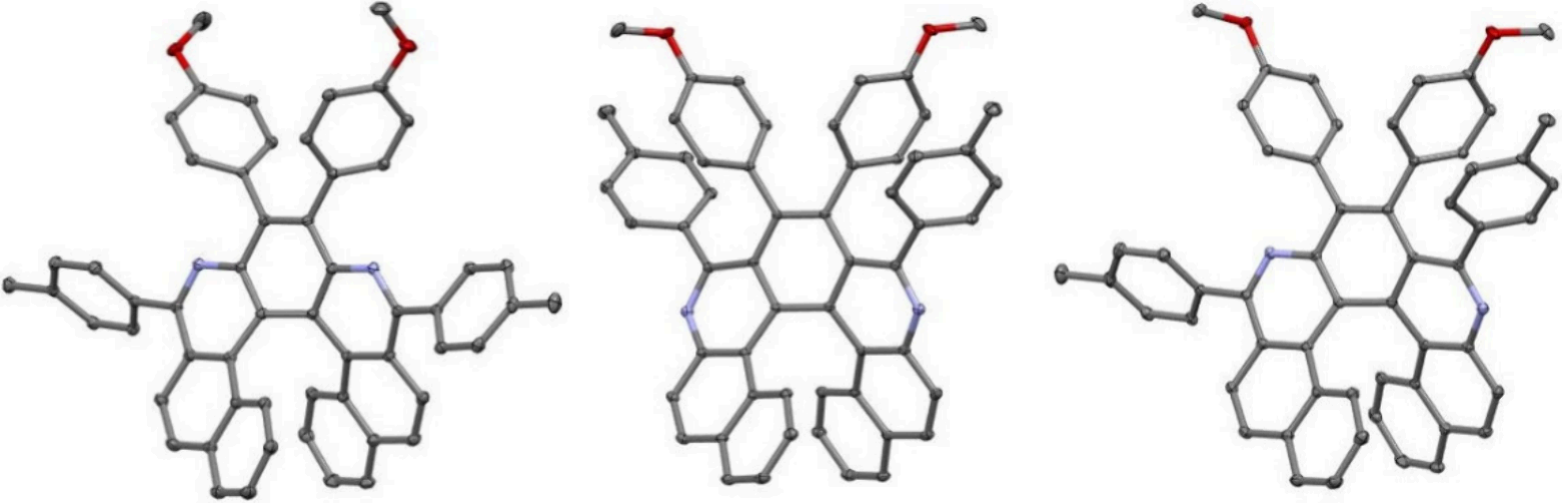
ORTEP drawings of **9**, **10** and **11**. Ellipsoids are drawn with 30% probability.

Sums of three inner rim dihedral angles (φ_5H_) reflect the helical pitch, and the obtained values are, where possible, compared with values known for the maternal *n*,*m*-diaza­[*n*]­helicenes (Table 2). The higher φ_5H_ of *n*,*m*-diaza­[5]­helicenes in comparison with the maternal [5]­helicene and unsubstituted 5,10-diaza[5]­helicene might be caused by the presence of additional substituents on the outer ring and their steric interactions. Such an effect has been observed in the series of substituted indeno­[2,1-*c*]­fluorene, where the helical pitch was bigger (∼38°),^17^ than in the pristine indeno­[2,1-*c*]­fluorene (∼18°).^50^ On the other hand, φ_7H_ for *n*,*m*-diaza­[7]­helicenes is almost the same as for the maternal [7]­helicenes.

**Table 2:** Selected Structural Data

Compound	φ_n_ (°)	CCDC
[5]helicene^a^ ^,^ ^47^	63.9^b^	2007122
5,10-diaza[5]helicene^48^	62.6^b^	686816
**4a**	67.0^b^	2448755
**5a**	65.3^b^	2448757
**6d**	68.6^b^	2448758
**6c**	65.3^b^	2448756
[7]helicene^49^	110.54^c^	2061539
**9**	110.0^c^	2448759
**10**	109.0^c^	2448761
**11**	113.0^c^	2448760

a Dibenzo­[c,g]­phenanthrene.

b φ_5H_ = the sum of 3 dihedral angles of the fjord region (the inner rim) of the [5]­helicene scaffold.

c φ_7H_ = the sum of 5 dihedral angles of the fjord region (the inner rim) of the [7]­helicene scaffold.

### Photophysical Properties

Absorption and fluorescence spectra for most synthesized diazahelicenes were recorded for dichloromethane solutions (Table 3). All diaza[5]­helicenes **4–6** exhibit emission in the blue light region with maxima in the range of 435–481 nm. The emission spectra are devoid of vibronic structure. In comparison with pristine 5,10- (412 and 438 nm) and 6,9-diaza[5]­helicenes (424 and 438 nm)^48^ all newly synthesized diazahelicenes exhibit red-shifted emission maxima with quantum yields in the range ∼2–24%. (Data for the emission spectrum of the maternal 5,9-diaza[5]­helicene are not known.) All three regioisomeric diaza[7]­helicenes **9–11** show emission in the cyan light region with maxima in the range of 492–495 nm with quantum yields from ∼6–16% (Figure 4). Interestingly, all synthesized 6,9-diaza[5]­helicenes (products **4a**, **4b**, **4d** and **4e**), together with 8,11-diaza[7]­helicene (**9**), have a higher Stokes shift (∼6597–8326 cm^–1^) compared to other regioisomers (∼866–1834 cm^–1^). In addition, a large bathochromic shift of emission maximum to 578 nm (the green-yellow light region) was observed for methylazonium salts **5a** ^**+**^ (for details see the SI, Ch. 5.4).

**Table 3:** Photophysical Properties of Studied Derivatives **4a**–**6a**, **4b**, **4e**, **5e**, **6c**, **9**, **10**, and **11**

Compound	λ_abs_ (nm)	*ε* (10^4^ M^–1^ cm^–1^)	Stokes shift (cm^–1^)	λ_em_ (nm)	Φ_F_ (%)
**4a**	345	2.36	8065.0	478	3
**5a**	323, 429	1.40, 0.24	1380.2	456, 472	12
**6a**	330, 426	2.29, 0.17	1640.1	458	12
**4b**	339	3.46	7472.1	454	7
**6c**	240, 318, 408	4.75, 3.04, 0.21	1834.1	441	20
**4d**	254, 347	4.08, 3.07	8326.6	488	8
**5d**	246, 317, 430	5.41, 2.84, 0.46	1657.5	463	24
**4e**	234, 274, 323, 338	2.94, 1.92, 2.04, 2.02	6597.3	435, 452	3
**5e**	242, 292, 318, 399, 422	6.23, 3.07, 3.06, 0.48, 0.39	865.6	438, 460	3
**9**	272, 285, 366	3.68, 3.68, 1.59	6997.2	492	5
**10**	249, 275, 439, 466	4.93, 4.69, 0.44, 0.5	1257.2	495	14
**11**	282, 364, 461	4.41, 1.81, 0.23	1366.8	492	16

**Figure 4:**
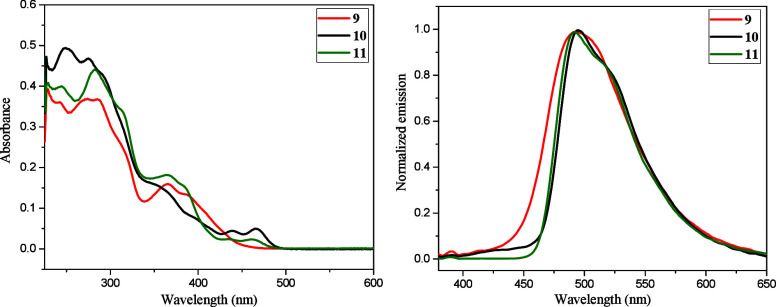
Absorption spectra (10^–5^ M) of **9**–**11** in CH_2_Cl_2_ (left). Normalized and corrected emission spectra (10^–6^ M) of **9**–**11** in CH_2_Cl_2_ using λ_exc_ = 350 nm (right).

## Conclusion

We report an efficient 2-fold skeletal editing procedure for the expansion of five-membered ring alcohols (prepared from ketones), as part of fluorenols, to the pyridine rings. Unlike the recently used methodology based on insertion reactions of reactive nitrene intermediates, our approach is based on Schmidt’s rearrangement. The rearrangement is robust and occurs in the absence of any catalyst. This method is suitable for the synthesis of variously substituted *m*,*n*-diaza­[*n*]­diazahelicenes in moderate to high overall yields. We have successfully shown that all three regioisomeric *m*,*n*-diaza­[5]­helicenes can be regioselectively formed in different acidic conditions or by thermal insertion from a diazide. The mechanism and sequence of steps for its formation were elucidated and supported by DFT calculations. Lastly, the 2-fold Schmidt rearrangement was successfully applied for the synthesis of chiral diaza[7]­helicenes from highly enantioenriched starting material, easily accessible by enantioselective cyclotrimerization, with negligible erosion of enantiopurity.

## Supplementary Material



## Notes

Experimental details, analytical data, crystallographic data, NMR spectra, HPLC data, and computational details (PDF)
