# Supplementary material for: Synthesis of m,n‑Diaza[n]helicenes via Skeletal Editing of Indeno[2,1‑c]fluorene-5,8-diols
Source: JACS Au. 2025 Oct 3;5(10):4788–98. doi: 10.1021/jacsau.5c00729 (PMC12569675; doi:10.1021/jacsau.5c00729)

Synthesis of *m,n*-Diaza[n]helicenes via Skeletal Editing of Indeno[2,1-*c*]fluorene-5,8-diols

Marina Degač,[a] Lena Reininger,[a,†] Hanna Schardax,[a,†] Erik Andris,*[b] Lubomír Rulíšek,[b] Ivana Císařová,[c] Uwe Rinner,[d] Timothée Cadart,[a] and Martin Kotora* [a]

[a] Department of Organic Chemistry, Faculty of Science, Charles University, Hlavova 8, 128 00 Praha 2 (Czech Republic). [b] Institute of Organic Chemistry and Biochemistry, Academy of Sciences of the Czech Republic, v.v.i. Flemingovo náměstí 2, 160 00 Praha 6 (Czech Republic). [c] Department of Inorganic Chemistry, Faculty of Science, Charles University, Hlavova 8, 128 00 Praha 2 (Czech Republic). [d] Institute of Applied Chemistry, IMC Krems University of Applied Sciences, Piaristengasse 1, 3500 Krems, Austria. † Interns from Institute of Applied Chemistry, IMC Krems University of Applied Sciences, Piaristengasse 1, 3500 Krems, Austria.

Table of contents

1 General Information 2
2 Nomenclature and position numbering in starting compounds and products 3
3 Synthesis of the starting materials 4
3.1 Synthesis of enantioenriched starting materials 14
4 Synthesis of diaza[n]helicenes 17
4.1 Reaction conditions assessment 17
4.2 Stepwise Schmidt rearrangement 26
4.3 Thermally induced rearrangement 28
4.4 Synthesis of diaza[5]helicenes 29
4.5 Synthesis of azonium salt 36
4.6 Synthesis of racemic diaza[7]helicenes 37
4.7 Synthesis of enantioenriched diaza[7]helicenes 39
5 Photophysical properties 43
5.1 UV/Vis absorption and emission spectra of **4a**, **5a** and **6a**. 43
5.2 UV/Vis absorption and emission spectra of **4b**–**6c**. 44
5.3 UV/Vis absorption and emission spectra of diaza[7]helicenes. 45
5.4 UV/Vis absorption and emission spectra of azonium salt. 46
6 Mechanistic studies 47
7 X-Ray diffraction data 50
8 Determination of racemization barriers of **10** and **11** 60
9 DFT calculations 63
9.1 XYZ Structures 66
10 Copies of ^{1}H and ^{13}C NMR spectra 72

1 General Information

All reagents were commercially available and purchased from Sigma-Aldrich, BLD Pharmatech, Acros Organics, Fluorochem, Alfa Aesar, and Strem Chemicals. Solvents were purified and dried by distillation: tetrahydrofuran (THF) and toluene from sodium/benzophenone, dichloromethane and 1,2-dichloroethane from calcium hydride. Other solvents and all reagents were used without further purification. Chromatography was performed on Silica gel P60 (40-63 μm) from Silicycle. Thin layer chromatography was performed on Silicycle silica gel 60 F_{254} pre-coated aluminum sheets.

NMR spectra were recorded on Bruker AVANCE III Spectrometer (^{1}H at 400 MHz, ^{13}C at 101 MHz, and ^{19}F at 376 MHz). All NMR spectra were measured in $CDCl_3$, C_6D_6 or d_6-DMSO solutions and referenced to residual solvent signal: $CDCl_3$ (^{1}H, $\delta_H = 7.26$; ^{13}C, $\delta_C = 77.16$), C_6D_6 (^{1}H, $\delta_H = 7.16$; ^{13}C, $\delta_C = 128.06$), d_6-DMSO (^{1}H, $\delta_H = 2.50$; ^{13}C, $\delta_C = 39.52$). Coupling constants J are given in Hz.

Infrared spectroscopy spectra were measured with a Nicolet Avatar 370 FTIR. The method used for measuring was a diffuse reflectance (DRIFT) in KBr or Attenuated Total Reflectance (ATR) with Ge crystal. IR absorptions are given in wavenumbers as cm^{-1}.

MS spectra were recorded on an Agilent Technologies 6530 Accurate-Mass Q-TOF LC/MS Samples were ionized by electrospray technique (ESI) and detected by quadrupole or TOF. Drying and nebulizer gas was nitrogen.

All melting points are uncorrected and were determined on a Kofler apparatus KB T300.

HPLC analysis were performed with a Shimadzu liquid chromatograph with a spectrophotometric detector (SPD-M20A). Chiral columns Daicel Chiralpak IA and IB were used for the separation of enantiomers.

Specific optical rotations were measured with AUTOMATIC polarimetry, Autopol III (Rudolph research, Flandres, New Jersey), and $CHCl_3$ was used as a solvent. Specific optical rotations are given in concentrations c [g/100 mL].

The UV/Vis absorption spectra were recorded in CH_2Cl_2 using Thermo Scientific Helios γ with wolfram and deuterium lamp. Steady-state fluorescence spectra were monitored on an FLS 980 spectrofluorometer (Edinburgh Instruments). Fluorescence quantum yields were determined using a Quantaurus-QY Plus spectrofluorometer (HamamatsuC13534-33). Fluorescence measurements of sample solutions in CH_2Cl_2 were measured in 1 cm pathlength cuvettes using samples with an absorbance of 0.1 or less at the excitation wavelength.

Microwave reactions were performed in an Anton Paar Monowave 400 device.

2 Nomenclature and position numbering in starting compounds and products

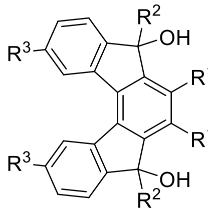

1

Table S1. Compound numbering of [5]helical compounds **1-7**.

Compound **1-7**	R^1	R^2	R^3
**a**	4-$MeOC_6H_4$	4-MeC_6H_4	-
**b**	4-$MeOC_6H_4$	2-MeC_6H_4	-
**c**	4-$MeOC_6H_4$	*n*-Bu	-
**d**	4-$MeOC_6H_4$	4-$CF_3C_6H_4$	
**e**	4-$CF_3C_6H_4$	4-MeC_6H_4	
**f**	H	4-MeC_6H_4	
**g**	Ph	4-MeC_6H_4	2-MeO

Numbering of positions in [7]helical compounds will start with **8** for the starting [7]helical indeno[2,1-*c*]fluorene-5,8-diols and **9-11** for the corresponding *m*,*n*-diaza[7]helicenes: **9** (8,11-diaza[7]helicene), **10** (7,12-diaza[7]helicene), and **11** (7,11-diaza[7]helicene).

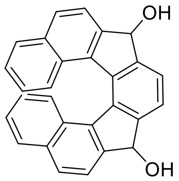

7,10-dihydrobenzo[*c*]benzo[6,7]indeno[1,2-*g*]fluorene-7,10-diol

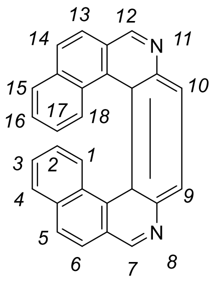

dinaphtho[1,2-*a*:2',1'-*k*][4,7]phenanthroline

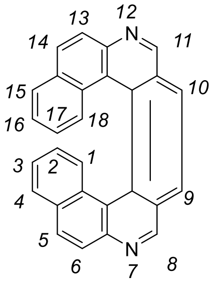

dinaphtho[1,2-*a*:2',1'-*k*][3,8]phenanthroline

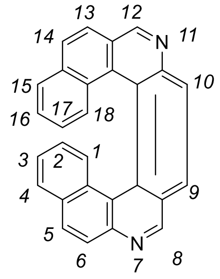

dinaphtho[1,2-*a*:2',1'-*k*][3,7]phenanthroline

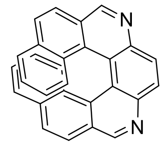

dinaphtho[1,2-*a*:2',1'-*k*][4,7]phenanthroline

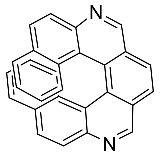

dinaphtho[1,2-*a*:2',1'-*k*][3,8]phenanthroline

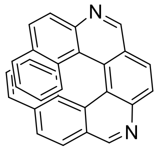

dinaphtho[1,2-*a*:2',1'-*k*][3,7]phenanthroline

Figure S1. IUPAC nomenclature and position numbering of *m*,*n*-diaza[7]helicenes

3 Synthesis of the starting materials

Starting materials were synthesized according to previously reported procedures[1] and new derivatives are described below.

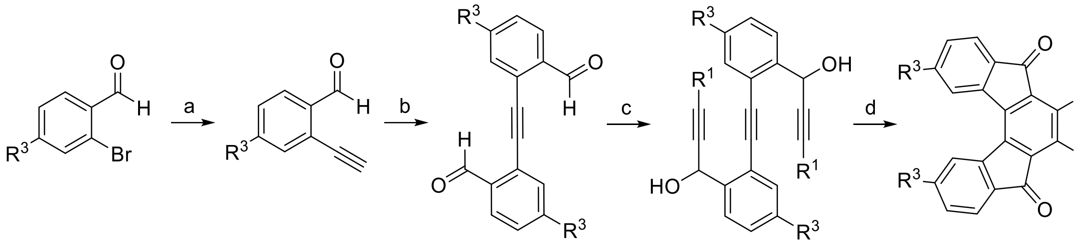

Scheme S1. Reaction conditions: a) 1) trimethylsilylacetylene (1.5 eq.), $PdCl_2(PPh_3)_2$ (1 mol%), CuI (2 mol%), anhydrous THF:Et_3N (4:1), 75 °C, 3 h; 2) K_2CO_3 (10 eq.), MeOH:H_2O (8:2), 0 °C, 1 h; b) 2-bromobenzaldehyde (1 eq.), $PdCl_2(PPh_3)_2$ (2 mol%), CuI (2 mol%), anhydrous THF:Et_3N (4:1), 75 °C, 3 h; c) the corresponding acetylene (3 eq.), *n*-BuLi (3 eq.), anhydrous THF, -78 to 25 °C, 3-24 h; d) 1) $Rh(PPh_3)_3Cl$ (1.5 mol%), anhydrous THF, MW, 170 °C, 2.5 h, 2) PCC (3 eq.), Celite® (same amount as PCC), CH_2Cl_2, 20 °C, 3 h.

Note: The respective triynediols (**S3**) were obtained as mixtures of *rac* and *meso* diastereoisomers in all cases. While in certain cases, complete assignment of NMR signals was achievable, in others, it was impractical due to signal overlaps. Consequently, for those instances, the peaks are documented as observed in the respective ^{1}H NMR spectra are listed and displayed with integration.

1,1'-(Ethyne-1,2-diylbis(2,1-phenylene))bis(prop-2-yn-1-ol) (S3f)

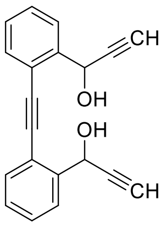

In a flame-dried Schlenk flask, *n*-BuLi 1.6 M (6 mmol, 3.8 mL) was added dropwise to a solution of trimethylsilylacetylene (6 mmol, 0.83 mL) in anhydrous THF (20 mL) at -78 °C under an inert atmosphere. After 1 hour, a solution of the dialdehyde **S2a** (2 mmol, 469 mg) in anhydrous THF (10 mL) was added and the reaction mixture was stirred for 5 min at -78 °C then, it was warmed up to 25 °C and stirred overnight. The reaction mixture was quenched with a saturated aqueous solution of NH_4Cl (10 mL) and extracted with EtOAc (3×10 mL). The combined organic layer was dried over Na_2SO_4, filtered, and concentrated under reduced pressure. The residue was treated with K_2CO_3 pellets in a mixture of MeOH/H_2O (8:2 mL) at 0 °C for 1 hour. The resulting mixture was quenched with a 1M HCl aq. solution (~20 mL), and extracted with CH_2Cl_2 (3×20 mL). The combined organic layer was dried over Na_2SO_4, filtered, and concentrated under reduced pressure. Column chromatography of the residue on silica gel (linear gradient: 4/1 to 2/1 hexanes/EtOAc) yielded 436 mg (76%) of the title compound (~3:1 inseparable mixture of diastereoisomers) as a brownish foam.

[1] Kaiser, R. P.; Nečas, D.; Cadart, T.; Gyepes, R.; Císařová, I.; Mosinger, J.; Pospíšil, L.; Kotora, M. *Angew. Chem. Int. Ed.* **2019**, *58*, 17169 –17174.

R_f (4/1 hexanes/EtOAc) = 0.10.

In a flame-dried Schlenk flask, ethynylmagnesium bromide 0.5 M in THF (10 mmol, 20 mL) was added dropwise to a solution of the dialdehyde **S2a** (3 mmol, 702 mg) in anhydrous THF (20 mL) at 0 °C under an inert atmosphere. The reaction mixture was stirred for 10 min at 0 °C, then warmed up to 25 °C and stirred for 3 hours. The reaction mixture was quenched with a saturated aqueous solution of NH_4Cl (10 mL) and extracted with EtOAc (3×10 mL). The combined organic layer was dried over Na_2SO_4, filtered, and concentrated under reduced pressure. Column chromatography of the residue on silica gel (2/1 hexanes/EtOAc) yielded 748 mg (88%) of the title compound (~3:1 mixture of diastereoisomers) as a brownish foam.

R_f (2/1 hexanes/EtOAc) = 0.23.

M.p. = 96 – 98 °C.

Major isomer: ^{1}H NMR (400 MHz, $CDCl_3$, 25 °C) δ 7.74 (dd, J = 7.6, 1.5 Hz, 2H), 7.61 – 7.58 (m, 2H), 7.43 – 7.33 (m, 4H), 5.97 (d, J = 2.3 Hz, 2H), 2.70 (d, J = 2.2 Hz, 2H).

^{13}C NMR (101 MHz, $CDCl_3$, 25 °C) δ 141.43 (2C), 132.81 (2C), 129.15 (2C), 128.62 (2C), 127.11 (2C), 121.70 (2C), 92.04 (2C), 82.76 (2C), 75.26 (2C), 63.15 (2C).

Minor isomer: ^{1}H NMR (400 MHz, $CDCl_3$, 25 °C) δ 7.78 (dd, J = 7.6, 1.5 Hz, 2H), 7.61 – 7.58 (m, 2H), 7.43 – 7.33 (m, 4H), 5.95 (d, J = 2.3 Hz, 2H), 2.71 (d, J = 2.2 Hz, 2H).

^{13}C NMR (101 MHz, $CDCl_3$, 25 °C) δ 141.33 (2C), 132.78 (2C), 129.15 (2C), 128.58 (2C), 126.92 (2C), 121.63 (2C), 92.11 (2C), 82.71 (2C), 75.33 (2C), 63.13 (2C).

IR (KBr) ν_{max} 3280, 3066, 3028, 2908, 2895, 2110, 2079, 1712, 1489, 1309, 1267, 1186, 1105, 1045, 756 cm^{-1}.

HRMS (ESI^+) m/z calcd for $C_{20}H_{14}NaO_2$ $[M + Na]^+$: 309.08860, found: 309.08896.

1,1'-(Ethyne-1,2-diylbis(4-methoxy-2,1-phenylene))bis(3-(*p*-tolyl)prop-2-yn-1-ol) (S3g)

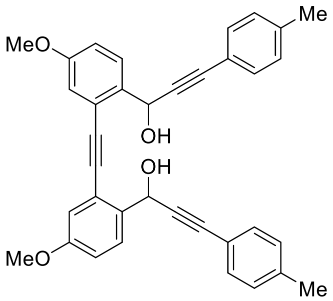

1st step – Sonogashira cross coupling

In a flame-dried Schlenk flask, $PdCl_2(PPh_3)_2$ (0.2 mmol, 140 mg), CuI (0.2 mmol, 38 mg), 2-bromo-4-methoxybenzaldehyde (4 mmol, 860 mg) and 2-ethynyl-4-methoxybenzaldehyde[2] (4 mmol, 641 mg) were dissolved in THF (30 mL) and Et_3N (12 mmol, 1.7 mL) under inert atmosphere. The reaction mixture was stirred at 50 °C overnight and then, it was cooled down to 25 °C, filtered through a pad of silica/Celite® (1:4), and washed with Et_2O. The filtrate was concentrated under reduced pressure and used in the next step as a crude mixture.

2nd step – Alkynylation reaction

In a flame-dried Schlenk flask, *n*-BuLi 1.6 M (6 mmol, 3.75 mL) was added dropwise to a solution of 1-ethynyl-4-methylbenzene (6 mmol, 0.77 mL) in anhydrous THF (15 mL) at -78 °C. After 1

[2] Wang, H.; Zeng, T.; Chang, W.; Liu, L.; Li, J. *Org. Lett.* **2021**, *23*, 3573−3577.

hour, a solution of the crude mixture (~2 mmol) in THF (10 ml) was added, and the reaction mixture was stirred for 5 min at -78 °C, then warmed up to 25 °C and stirred overnight. The reaction mixture was quenched with a saturated aqueous solution of NH_4Cl and extracted with EtOAc (3× 10 mL). The combined organic layer was dried over Na_2SO_4, filtered, and concentrated under reduced pressure. Column chromatography of the residue on silica gel (linear gradient: 3/1 to 1/1 hexanes/EtOAc) yielded 317 mg (15% in two steps) of the title compound (~1:1 inseparable mixture of diastereoisomers) as a brownish foam.

R_f (3/1 hexanes/EtOAc) = 0.10.

M.p. = 92 – 94 °C.

First isomer: ^{1}H NMR (400 MHz, $CDCl_3$, 25 °C) δ 7.77 (d, *J* = 8.6 Hz, 2H), 7.38 – 7.34 (m, 4H), 7.14 (d, *J* = 2.7 Hz, 2H), 7.11 – 7.07 (m, 4H), 6.96 – 6.92 (m, 2H), 6.14 (d, *J* = 8.1 Hz, 2H), 3.82 (s, 6H), 2.34 (s, 6H).

^{13}C NMR (101 MHz, $CDCl_3$, 25 °C) δ 159.3 (2C), 138.6 (2C), 134.8 (2C), 131.7 (4C), 129.0 (4C), 128.6 (2C), 122.9 (2C), 119.4 (2C), 117.3 (2C), 115.4 (2C), 91.9 (2C), 87.66 (2C), 87.1 (2C), 63.42 (2C), 55.5 (2C), 21.50 (2C).

Second isomer: ^{1}H NMR (400 MHz, $CDCl_3$, 25 °C) δ 7.70 (d, *J* = 8.6 Hz, 2H), 7.38 – 7.34 (m, 4H), 7.14 (d, *J* = 2.7 Hz, 2H), 7.11 – 7.07 (m, 4H), 6.96 – 6.92 (m, 2H), 6.14 (d, *J* = 8.1 Hz, 2H), 3.83 (s, 6H), 2.32 (s, 6H).

^{13}C NMR (101 MHz, $CDCl_3$, 25 °C) δ 159.4 (2C), 138.7 (2C), 135.0 (2C), 131.7 (4C), 129.1 (4C), 128.8 (2C), 122.9 (2C), 119.4 (2C), 117.5 (2C), 115.3 (2C), 92.0 (2C), 87.73 (2C), 87.0 (2C), 63.44 (2C), 55.5 (2C), 21.48 (2C).

IR (KBr) ν_{max} 3348, 3080, 3047, 3028, 2997, 2943, 2922, 2866, 2835, 2227, 2197, 1765, 1699, 1603, 1508, 1284, 1236, 1213, 1034, 818 cm^{-1}.

HRMS (ESI$^+$) m/z calcd for $C_{36}H_{30}NaO_4$ $[M + Na]^+$: 549.20363, found: 549.20384.

Indeno[2,1-*c*]fluorene-5,8-dione (S4f)

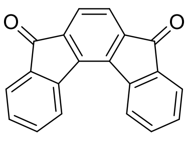

In a flame-dried microwave vial, **S3f** (0.96 mmol, 276 mg) was dissolved in anhydrous THF (10 ml) under an argon atmosphere. After adding the Wilkinson's catalyst (0.02 mmol, 19 mg) the reaction vial was sealed and heated at 170 °C for 2.5 h in a microwave reactor. The reaction mixture was cooled down to 25 °C and the solvent was evaporated under reduced pressure. Then the crude products (diols) were oxidized to the corresponding diketones without any further purification. Pyridinium chlorochromate (PCC, 3 mmol, 647 mg) and Celite® (647 mg) were added to a solution of a crude diol in CH_2Cl_2 (20 mL), and the resulting mixture was stirred at 25 °C for 3 hours. The reaction mixture was filtered through a pad of Florisil®/silica/Celite® (1:1:4), washed with CH_2Cl_2, concentrated under reduced pressure, and used in the next step without further purification. The title compound was obtained as a yellow solid (262 mg, 97%).

M.p. (decomp) > 260 °C.

^{1}H NMR (400 MHz, $CDCl_3$, 25 °C) δ 8.07 (d, *J* = 7.6 Hz, 2H), 7.81 (d, *J* = 7.3 Hz, 2H), 7.66 – 7.63 (m, 4H), 7.43 (t, *J* = 7.4 Hz, 2H).

^{13}C NMR (101 MHz, $CDCl_3$, 25 °C) δ 192.2 (2C), 143.6 (2C), 140.6 (2C), 138.6 (2C), 135.2 (2C), 135.1 (2C), 129.8 (2C), 125.1 (2C), 125.0 (2C), 124.2 (2C).

IR (ATR) ν_{max} 3396, 3057, 3014, 2960, 2920, 2850, 1709, 1606, 1414, 1248, 1059, 924, 752, 704 cm^{-1}.

HRMS (ESI^+) m/z calcd for $C_{20}H_{10}NaO_2$ $[M + Na]^+$: 305.05730, found: 305.05772.

2,11-dimethoxy-6,7-di-*p*-tolylindeno[2,1-*c*]fluorene-5,8-dione (S4g)

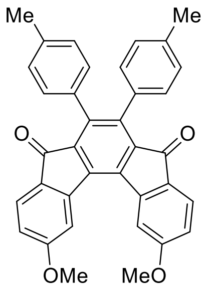

In a flame-dried microwave vial, **S3g** (0.6 mmol, 305 mg) was dissolved in anhydrous THF (8 ml) under an argon atmosphere. After adding the Wilkinson's catalyst (0.01 mmol, 9 mg) the reaction vial was sealed and heated at 170 °C for 2.5 h in a microwave reactor. The reaction mixture was cooled down to 25 °C and the solvent was evaporated under reduced pressure. Then the crude products (diols) were oxidized to the corresponding diketones without any further purification. Pyridinium chlorochromate (PCC, 1.8 mmol, 388 mg) and Celite® (388 mg) were added to a solution of a crude diol in CH_2Cl_2 (20 mL), and the resulting mixture was stirred at 25 °C for 3 hours. The reaction mixture was filtered through a pad of Florisil®/silica/Celite® (1:1:4), washed with CH_2Cl_2, concentrated under reduced pressure, and used in the next step without further purification. The title compound was obtained as a yellow solid (200 mg, 64%).

M.p. = 343 – 345 °C.

^1H NMR (400 MHz, $CDCl_3$, 25 °C) δ 7.72 (d, J = 2.1 Hz, 2H), 7.64 (d, J = 8.2 Hz, 2H), 7.01 (d, J = 7.8 Hz, 4H), 6.89 – 6.84 (m, 6H), 3.97 (s, 6H), 2.30 (s, 6H).

^{13}C NMR (101 MHz, $CDCl_3$, 25 °C) δ 190.3 (2C), 165.3 (2C), 145.0 (2C), 143.2 (2C), 137.1 (2C), 137.0 (2C), 136.7 (2C), 132.2 (2C), 129.4 (4C), 128.8 (2C), 128.1 (4C), 126.7 (2C), 113.1 (2C), 111.2 (2C), 56.0 (2C), 21.4 (2C).

IR (ATR) ν_{max} 3120, 3084, 3047, 3014, 3005, 2978, 2943, 2922, 2852, 2837, 1697, 1604, 1581, 1236, 1020, 843, 825, 785, 758, 721 cm^{-1}.

HRMS (ESI^+) m/z calcd for $C_{36}H_{27}O_4$ $[M + H]^+$: 523.19039, found: 523.18950.

General procedure A: Arylation/alkylation reaction of indenofluorenones

In a flame-dried Schlenk flask, *n*-BuLi 1.6 M (4 mmol) was added dropwise to a solution of the corresponding aryl halide (4 mmol) in anhydrous THF (15 mL) at -78 °C under an inert atmosphere. After 1 hour, a solution of the diketone **S4** (1 mmol) in anhydrous THF (10 ml) was added, and the reaction mixture was stirred for 5 min at -78 °C then, it was warmed up to 25 °C and stirred for 16 hours. The reaction mixture was quenched with a saturated aqueous solution of NH_4Cl and extracted with EtOAc (3×20 mL). The combined organic layer was dried over Na_2SO_4, filtered, and concentrated under reduced pressure. Column chromatography of the residue on silica gel (hexanes/EtOAc) provided products.

Note: In all cases, the respective diols were obtained as mixtures of *rac* and *meso* diastereoisomers. While in certain cases, complete assignment of NMR signals was achievable, in others, it was impractical due to signal overlap. Consequently, for those instances, the peaks are documented as observed, and the respective ^{1}H NMR spectra are listed and displayed with integration.

6,7-Bis(4-methoxyphenyl)-5,8-di-*p*-tolyl-5,8-dihydroindeno[2,1-*c*]fluorene-5,8-diol (1a)

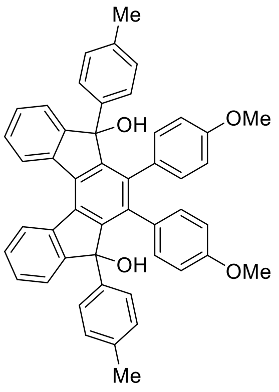

With 4-bromotoluene (4 mmol, 0.49 mL) and the diketone **S4a** (1 mmol, 495 mg) following the general procedure **A**. Column chromatography of the residue on silica gel (4/1 hexanes/EtOAc) provided 646 mg (95%) of the title compound (~2:1 mixture of diastereoisomers) as a yellow solid. One fraction contained 270 mg (40%) of only the major diastereoisomer.

R_f (4/1 hexanes/EtOAc) = 0.34 (major diastereoisomer), 0.17 (minor diastereoisomer).

Major diastereoisomer

M.p. = 245 – 247 °C.

^{1}H NMR (400 MHz, $CDCl_3$, 25 °C) δ 8.47 (d, *J* = 7.8 Hz, 2H), 7.46 – 7.42 (m, 2H), 7.29 – 7.27 (m, 4H), 7.01 (d, *J* = 8.4 Hz, 4H), 6.96 (d, *J* = 8.1 Hz, 4H), 6.84 (dd, *J* = 8.4, 2.3 Hz, 2H), 6.56 (dd, *J* = 8.4, 2.8 Hz, 2H), 6.22 (dd, *J* = 8.5, 2.8 Hz, 2H), 5.94 (dd, *J* = 8.5, 2.3 Hz, 2H), 3.66 (s, 6H), 2.55 (s, 2H), 2.29 (s, 6H).

^{13}C NMR (101 MHz, $CDCl_3$, 25 °C) δ 158.0 (2C), 151.6 (2C), 150.7 (2C), 141.8 (2C), 138.7 (2C), 138.5 (2C), 136.2 (2C), 135.0 (2C), 131.7 (2C), 131.0 (2C), 128.8 (4C), 128.59 (2C), 128.57 (2C), 128.5 (2C), 124.7 (4C), 124.4 (2C), 123.6 (2C), 112.7 (2C), 112.5 (2C), 84.3 (2C), 55.0 (2C), 21.1 (2C).

IR (ATR) ν_{max} 3535, 3444, 3041, 3022, 2999, 2952, 2927, 2860, 2883, 1608, 1510, 1464, 1419, 1284, 1242, 1174, 1107, 1032, 833, 800, 748 cm^{-1}.

HRMS (ESI$^+$) m/z calcd for $C_{48}H_{38}NaO_4$ [M + Na]$^+$: 701.26623, found: 701.26607.

6,7-Bis(4-methoxyphenyl)-5,8-di-*o*-tolyl-5,8-dihydroindeno[2,1-*c*]fluorene-5,8-diol (1b)

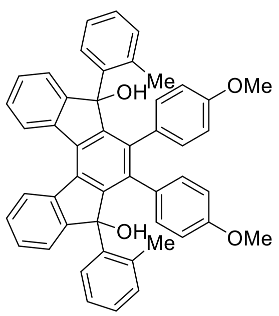

With 2-bromotoluene (4 mmol, 0.48 mL) and the diketone **S4a** (1 mmol, 495 mg) following the general procedure **A**. Column chromatography of the residue on silica gel (5/1 hexanes/EtOAc) provided 598 mg (88%) of the title compound (~3:2 mixture of diastereoisomers) as a bright yellow solid.

R_f (5/1 hexanes/EtOAc) = 0.36 (major diastereoisomer), 0.18 (minor diastereoisomer).

Major diastereoisomer

M.p. = 290 – 292 °C.

^{1}H NMR (400 MHz, $CDCl_3$, 25 °C) δ 8.52 (d, J = 7.8 Hz, 2H), 7.49 (td, J = 7.6, 1.3 Hz, 2H), 7.33 – 7.28 (m, 4H), 7.10 (d, J = 7.5 Hz, 2H), 7.00 (td, J = 7.4, 1.4 Hz, 2H), 6.89 (td, J = 8.3, 7.6, 1.8 Hz, 4H), 6.84 (d, J = 7.4 Hz, 2H), 6.58 (dd, J = 8.3, 2.7 Hz, 2H), 5.92 (dd, J = 8.5, 2.8 Hz, 2H), 5.79 (dd, J = 8.6, 2.2 Hz, 2H), 3.62 (s, 6H), 2.60 (s, 2H), 1.56 (s, 6H).

^{13}C NMR (101 MHz, $CDCl_3$, 25 °C) δ 157.7 (2C), 149.5 (2C), 148.9 (2C), 141.5 (2C), 139.9 (2C), 139.5 (2C), 135.6 (2C), 133.7 (2C), 130.8 (2C), 130.7 (2C), 130.3 (2C), 128.8 (2C), 128.7 (2C), 128.6 (2C), 126.8 (2C), 125.7 (2C), 125.6 (2C), 124.9 (2C), 123.6 (2C), 112.5 (2C), 112.1 (2C), 82.8 (2C), 55.0 (2C), 19.6 (2C).

IR (ATR) ν_{max} 3516, 3492, 3440, 3396, 3059, 3043, 3024, 3005, 2960, 2910, 2883, 1608, 1516, 1508, 1456, 1417, 1230, 1176, 1011, 829, 752 cm^{-1}.

HRMS (ESI$^+$) m/z calcd for $C_{48}H_{38}NaO_4$ [M + Na]$^+$: 701.26623, found: 701.26692.

5,8-Dibutyl-6,7-bis(4-methoxyphenyl)-5,8-dihydroindeno[2,1-*c*]fluorene-5,8-diol (1c)

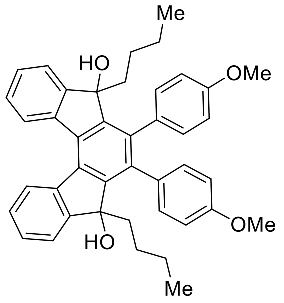

With *n*-BuLi (2.3 mmol, 1.6 M, 1.4 mL) and the diketone **S4a** (0.75 mmol, 370 mg) following the general procedure **A**. Column chromatography of the residue on silica gel (3/1 hexanes/EtOAc) provided 260 mg (57%) of the title compound (~2:1 mixture of diastereoisomers) as a yellow solid.

Major diastereoisomer

R_f (3/1 hexanes/EtOAc) = 0.25.

^{1}H NMR (400 MHz, $CDCl_3$, 25 °C) δ 8.38 (d, J = 7.7 Hz, 2H), 7.48 – 7.42 (m, 4H), 7.35 (t, J = 7.4 Hz, 2H), 7.04 (dd, J = 8.5, 2.1 Hz, 2H), 6.99 (dd, J = 8.3, 1.9 Hz, 2H), 6.75 – 6.69 (m, 4H), 3.76 (s, 6H), 2.07 (br s, 2H), 1.92 (td, J = 12.5, 4.5 Hz, 2H), 1.65 (td, J = 12.5, 4.6 Hz, 2H), 1.07 (p, J = 7.2 Hz, 4H), 0.90 – 0.80 (m, 2H), 0.71 (t, J = 7.3 Hz, 6H), 0.65 – 0.57 (m, 2H).

^{13}C NMR (101 MHz, $CDCl_3$, 25 °C) δ 158.3 (2C), 149.5 (2C), 146.9 (2C), 139.3 (2C), 138.6 (2C), 134.7 (2C), 131.8 (2C), 131.5 (2C), 129.5 (2C), 128.4 (2C), 128.0 (2C), 123.3 (2C), 123.2 (2C), 113.0 (2C), 112.5 (2C), 83.4 (2C), 55.1 (2C), 39.6 (2C), 26.2 (2C), 22.6 (2C), 13.9 (2C).

Minor diastereoisomer

R_f (3/1 hexanes/EtOAc) = 0.48.

M.p. = 137 – 139 °C.

^{1}H NMR (400 MHz, $CDCl_3$, 25 °C) δ 8.38 (d, *J* = 7.6 Hz, 2H), 7.48 (d, *J* = 7.5 Hz, 2H), 7.45 (td, *J* = 7.6, 1.3 Hz, 2H), 7.36 (t, *J* = 7.4 Hz, 2H), 7.28 (dd, *J* = 8.4, 2.1 Hz, 2H), 6.82 – 6.78 (m, 4H), 6.66 (dd, *J* = 8.5, 2.7 Hz, 2H), 3.77 (s, 6H), 2.14 (br s, 2H), 1.88 (td, *J* = 12.4, 4.4 Hz, 2H), 1.52 (td, *J* = 12.5, 4.6 Hz, 2H), 1.07 – 0.96 (m, 4H), 0.88 – 0.75 (m, 4H), 0.67 (t, *J* = 7.3 Hz, 6H).

^{13}C NMR (101 MHz, $CDCl_3$, 25 °C) δ 158.3 (2C), 149.5 (2C), 147.4 (2C), 139.4 (2C), 138.6 (2C), 134.5 (2C), 131.7 (2C), 131.6 (2C), 129.8 (2C), 128.4 (2C), 128.0 (2C), 123.3 (2C), 123.2 (2C), 113.3 (2C), 112.3 (2C), 83.7 (2C), 55.1 (2C), 39.5 (2C), 26.2 (2C), 22.6 (2C), 13.8 (2C).

IR (ATR) ν_{max} 3535, 3417, 3064, 3039, 2999, 2954, 2927, 2858, 2835, 1709, 1608, 1516, 1464, 1284, 1242, 1174, 1032, 833, 756 cm^{-1}.

HRMS (ESI$^+$) m/z calcd for $C_{42}H_{42}NaO_4$ [M + Na]$^+$: 633.29753, found: 633.29724.

6,7-Bis(4-methoxyphenyl)-5,8-bis(4-(trifluoromethyl)phenyl)-5,8-dihydroindeno[2,1-*c*]fluorene-5,8-diol (1d)

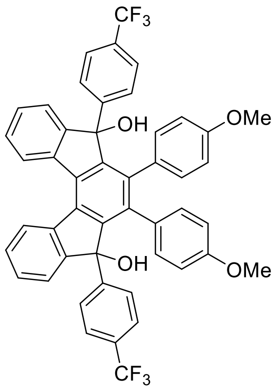

With 4-bromobenzotrifluoride (4.8 mmol, 0.67 mL) and the diketone **S4a** (1.2 mmol, 593 mg) following the general procedure **A** for 48 hours. Column chromatography of the residue on silica gel (5/1 hexanes/EtOAc) provided 896 mg (95%) of the title compound (~2:1 mixture of diastereoisomers) as a yellow solid. One fraction contained 102 mg (11%) of only the major diastereoisomer.

Major diastereoisomer

R_f (5/1 hexanes/EtOAc) = 0.26.

M.p. = 291 – 293 °C.

^{1}H NMR (400 MHz, $CDCl_3$, 25 °C) δ 8.51 (d, *J* = 7.8 Hz, 2H), 7.51 (td, *J* = 7.6, 1.3 Hz, 2H), 7.35 – 7.30 (m, 6H), 7.22 (dd, *J* = 7.7, 1.3 Hz, 2H), 7.14 (d, *J* = 8.1 Hz, 4H), 6.90 (dd, *J* = 8.4, 2.2 Hz, 2H), 6.61 (dd, *J* = 8.4, 2.7 Hz, 2H), 6.11 (dd, *J* = 8.5, 2.7 Hz, 2H), 5.79 (dd, *J* = 8.5, 2.2 Hz, 2H), 3.64 (s, 6H), 2.67 (s, 2H).

^{13}C NMR (101 MHz, $CDCl_3$, 25 °C) δ 158.2 (2C), 150.8 (2C), 150.4 (2C), 148.3 (2C), 139.5 (2C), 138.6 (2C), 135.0 (2C), 131.3 (2C), 130.8 (2C), 129.1 (2C), 128.9 (2C), 128.8 (q, $^2J_{C\text{-}F}$ = 32.2 Hz, 2C), 128.3 (2C), 127.2 (q, $^3J_{C\text{-}F}$ = 3.8 Hz, 2C), 126.9 (q, $^1J_{C\text{-}F}$ = 272 Hz, 2C), 125.2 (4C), 124.8 (q, $^1J_{C\text{-}F}$ = 3.9 Hz, 2C), 123.8 (2C), 115.4 (2C), 112.9 (2C), 112.5 (2C), 83.8 (2C), 55.0 (2C).

^{19}F NMR (376.5 MHz, $CDCl_3$, 25 °C) δ -62.38.

IR (ATR) ν_{max} 3529, 3068, 3005, 2962, 2937, 2914, 2841, 1608, 1512, 1410, 1323, 1286, 1244, 1163, 1103, 1066, 1014, 835, 804, 750 cm^{-1}.

HRMS (ESI$^+$) m/z calcd for $C_{48}H_{32}F_6NaO_4$ [M + Na]$^+$: 809.20970, found: 809.20982.

5,8-Di-*p*-tolyl-6,7-bis(4-(trifluoromethyl)phenyl)-5,8-dihydroindeno[2,1-*c*]fluorene-5,8-diol (1e)

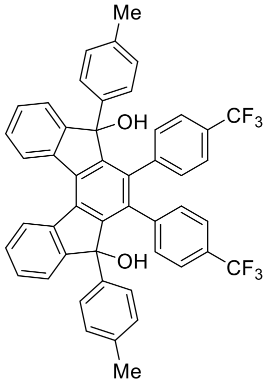

With 4-bromotoluene (3.7 mmol, 0.45 mL) and the diketone **S4e** (1.23 mmol, 700 mg) following the general procedure **A**. Column chromatography of the residue on silica gel (10/1 hexanes/EtOAc) provided 605 mg (65%) of the title compound (one diastereoisomer) as a bright yellow solid.

R_f (10/1 hexanes/EtOAc) = 0.18.

M.p. = 323 – 325 °C.

^{1}H NMR (400 MHz, $CDCl_3$, 25 °C) δ 8.50 (d, J = 7.8 Hz, 2H), 7.49 (td, J = 7.5, 1.3 Hz, 2H), 7.33 – 7.29 (m, 4H), 7.22 – 7.17 (m, 4H), 6.83 (d, J = 7.9 Hz, 4H), 6.80 – 6.76 (m, 6H), 6.04 (d, J = 8.1 Hz, 2H), 2.36 (s, 2H), 2.24 (s, 6H).

^{13}C NMR (101 MHz, $CDCl_3$, 25 °C) δ 152.0 (2C), 150.4 (2C), 140.2 (2C), 139.6 (2C), 138.3 (2C), 137.9 (2C), 136.3 (2C), 135.4 (2C), 130.9 (2C), 130.7 (2C), 129.1 (2C), 128.9 (2C), 128.58 (4C), 128.57 (q, $^2J_{C\text{-}F}$ = 32.1 Hz, 2C), 124.7 (2C), 124.6 (4C), 124.0 (q, $^1J_{C\text{-}F}$ = 273 Hz, 2C), 123.7 (2C), 123.57 (q, $^3J_{C\text{-}F}$ = 4.4 Hz, 2C), 83.8 (2C), 21.0 (2C) (1 carbon signal is probably covered by other signals).

^{19}F NMR (376.5 MHz, $CDCl_3$, 25 °C) δ -62.72

IR (ATR) ν_{max} 3562, 3541, 3024, 2924, 2868, 1616, 1510, 1406, 1321, 1159, 1120, 1109, 1066, 1018, 850, 750 cm^{-1}.

HRMS (ESI$^+$) m/z calcd for $C_{48}H_{32}F_6NaO_2$ [M + Na]$^+$: 777.21987, found: 777.22021.

5,8-Di-*p*-tolyl-5,8-dihydroindeno[2,1-*c*]fluorene-5,8-diol (1f)

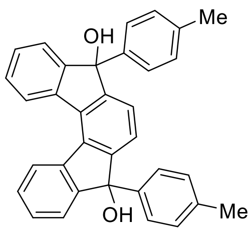

With 4-bromotoluene (7 mmol, 0.9 mL) and the diketone **S4f** (1.77 mmol, 500 mg) following the general procedure **A**. Column chromatography of the residue on silica gel (linear gradient: 5/1 to 1/1 hexanes/EtOAc) of the residue on silica gel provided 774 mg (94%) of the title (~2:1 mixture of diastereoisomers) compound as a yellow foam.

Major diastereoisomer

R_f (5/1 hexanes/EtOAc) = 0.3.

M.p. = 152 – 154 °C.

^{1}H NMR (400 MHz, $CDCl_3$, 25 °C) δ 8.38 (d, J = 7.8 Hz, 2H), 7.49 (td, J = 7.5, 1.3 Hz, 2H), 7.45 – 7.42 (m, 2H), 7.36 – 7.32 (m, 6H), 7.24 (s, 2H), 7.08 (d, J = 7.9 Hz, 4H), 2.44 (s, 2H), 2.30 (s, 6H).

^{13}C NMR (101 MHz, $CDCl_3$, 25 °C) δ 153.4 (2C), 151.6 (2C), 140.3 (2C), 139.4 (2C), 137.0 (2C), 135.0 (2C), 129.01 (4C), 128.98 (2C), 128.7 (2C), 125.3 (4C), 125.1 (2C), 124.5 (2C), 123.7 (2C), 83.0 (2C), 21.1 (2C).

IR (ATR) ν_{max} 3541, 3350, 3045, 3024, 2949, 2920, 2866, 1510, 1419, 1151, 1024, 1014, 926, 831, 810, 800, 762, 748, 719 cm^{-1}.

HRMS (ESI^+) m/z calcd for $C_{34}H_{25}O$ $[M + H]^+$: 449.18999, found: 449.19030.

Minor diastereoisomer

R_f (5/1 hexanes/EtOAc) = 0.06.

^{1}H NMR (400 MHz, $CDCl_3$, 25 °C) δ 8.37 (d, J = 7.8 Hz, 2H), 7.48 (td, J = 7.5, 1.4 Hz, 2H), 7.40 (dd, J = 7.5, 1.9 Hz, 2H), 7.33 (td, J = 7.3, 1.0 Hz, 2H), 7.28 (d, J = 8.4 Hz, 4H), 7.17 (s, 2H), 7.06 (d, J = 7.8 Hz, 4H), 2.52 (s, 2H), 2.29 (s, 6H).

^{13}C NMR (101 MHz, $CDCl_3$, 25 °C) δ 153.1 (2C), 151.4 (2C), 140.2 (2C), 139.5 (2C), 136.9 (2C), 134.9 (2C), 129.0 (2C), 128.9 (4C), 128.6 (2C), 125.4 (4C), 125.3 (2C), 124.6 (2C), 123.7 (2C), 82.9 (2C), 21.1 (2C).

2,11-Dimethoxy-5,8-diphenyl-6,7-di-*p*-tolyl-5,8-dihydroindeno[2,1-*c*]fluorene-5,8-diol (1g)

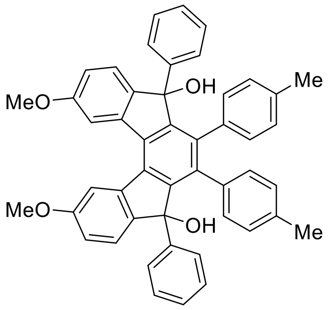

With bromobenzene (1.2 mmol, 0.13 mL) and the diketone **S4g** (0.36 mmol, 190 mg) following the general procedure **A**. Column chromatography of the residue on silica gel (linear gradient: 5/1 to 2/1 hexanes/EtOAc) provided 79 mg (32%) of the title (~2:1 mixture of diastereoisomers) compound as a yellow solid. One fraction contained 29 mg (12%) of only the major diastereoisomer.

R_f (5/1 hexanes/EtOAc) = 0.27 (major diastereoisomer), 0.18 (minor diastereoisomer).

Major diastereoisomer

^{1}H NMR (400 MHz, $CDCl_3$, 25 °C) δ 8.04 (d, J = 2.3 Hz, 2H), 7.17 – 7.07 (m, 12H), 6.83 – 6.81 (m, 6H), 6.43 (d, J = 7.8 Hz, 2H), 5.86 (d, J = 7.9 Hz, 2H), 3.92 (s, 6H), 2.56 (s, 2H), 2.13 (s, 6H).

^{13}C NMR (101 MHz, $CDCl_3$, 25 °C) δ 160.3 (2C), 151.0 (2C), 144.9 (2C), 143.9 (2C), 139.9 (2C), 138.8 (2C), 136.1 (2C), 134.8 (2C), 133.1 (2C), 130.3 (2C), 129.5 (2C), 127.99 (4C), 127.95 (2C), 127.93 (2C), 126.5 (2C), 125.2 (2C), 124.7 (4C), 114.1 (2C), 109.6 (2C), 83.8 (2C), 55.7 (2C), 21.1 (2C).

8,9-Bis(4-methoxyphenyl)-7,10-di-*p*-tolyl-7,10-dihydrobenzo[c]benzo[6,7]indeno[1,2-*g*]fluorene-7,10-diol (8)

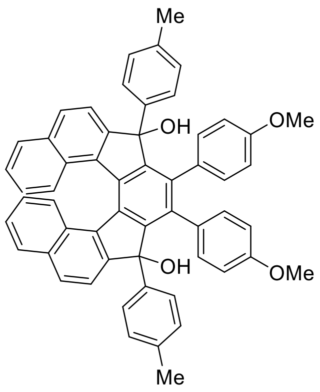

With 4-bromotoluene (2 mmol, 0.24 mL) and the diketone **S6**[1] (0.51 mmol, 303 mg) following the general procedure **A**. Column chromatography of the residue on silica gel (5/1 hexanes/EtOAc) provided 330 mg (83%) of the title compound (one diastereoisomer) as a bright yellow solid.

R_f (5/1 hexanes/EtOAc) = 0.25.

M.p. = 233 – 235 °C.

^{1}H NMR (400 MHz, $CDCl_3$, 25 °C) δ 7.82 (d, *J* = 8.1 Hz, 2H), 7.79 (d, *J* = 8.2 Hz, 2H), 7.70 (d, *J* = 8.6 Hz, 2H), 7.48 (d, *J* = 8.3 Hz, 2H), 7.31 (d, *J* = 8.3 Hz, 4H), 7.20 (ddd, *J* = 8.1, 6.8, 1.2 Hz, 2H), 7.07 (d, *J* = 7.9 Hz, 4H), 6.83 (dd, *J* = 8.4, 2.3 Hz, 2H), 6.72 – 6.65 (m, 2H), 6.57 (dd, *J* = 8.4, 2.7 Hz, 2H), 6.37 (dd, *J* = 8.5, 2.8 Hz, 2H), 6.12 (dd, *J* = 8.4, 2.2 Hz, 2H), 3.70 (s, 6H), 2.59 (s, 2H), 2.34 (s, 6H).

^{13}C NMR (101 MHz, $CDCl_3$, 25 °C) δ 158.2 (2C), 151.8 (2C), 149.6 (2C), 141.7 (2C), 136.6 (2C), 136.4 (2C), 136.3 (2C), 135.4 (2C), 134.0 (2C), 131.5 (2C), 131.4 (2C), 130.1 (2C), 129.5 (2C), 129.2 (4C), 128.5 (2C), 128.2 (2C), 127.0 (2C), 125.5 (2C), 125.0 (2C), 124.8 (4C), 120.8 (2C), 115.1 (2C), 112.9 (2C), 84.9 (2C), 55.1 (2C), 21.1 (2C).

IR (ATR) ν_{max} 3543, 3049, 3020, 2997, 2951, 2922, 2858, 2833, 1608, 1508, 1457, 1415, 1284, 1242, 1174, 1107, 1074, 1030, 820, 808, 791, 746, 723 cm^{-1}.

HRMS (ESI$^+$) m/z calcd for $C_{56}H_{42}NaO_4$ [M + Na]$^+$: 801.29753, found: 801.29903.

3.1 Synthesis of enantioenriched starting materials

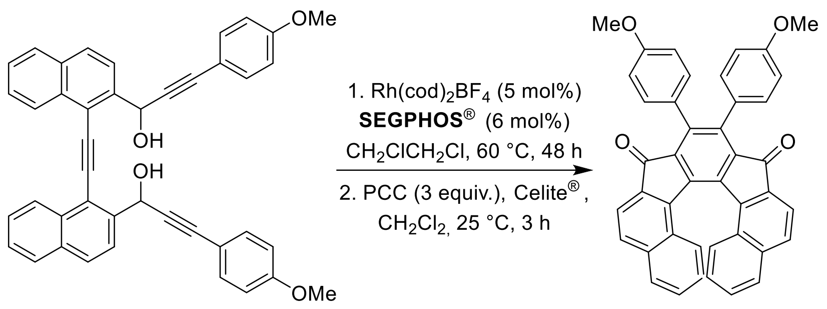

S5 **S6**

Scheme S2. Enantioselective [2+2+2] cyclotrimerization followed by oxidation reaction.

(*M*)-8,9-Bis(4-methoxyphenyl)benzo[*c*]benzo[6,7]indeno[1,2-*g*]fluorene-7,10-dione (S6)

A flame-dried microwave vial was loaded with $Rh(cod)_2BF_4$ (0.05 mmol, 20.3 mg) and (*R*) - SEGPHOS® (0.06 mmol, 36.6 mg) in dry CH_2ClCH_2Cl (10 mL) under an argon atmosphere. Hydrogen gas was bubbled into the reaction mixture for 45 minutes. Subsequently, the respective triynediol **S5** (1 mmol) was introduced under the argon atmosphere. The reaction was allowed to proceed at 60 °C for 48 hours. Afterward, the reaction mixture was concentrated under reduced pressure. The crude diols were directly oxidized to the corresponding diketone **S6** without additional purification steps. The crude diols were dissolved in CH_2Cl_2 (100 mL) and pyridinium chlorochromate (3.0 mmol, 647 mg) and Celite® (700 mg) were added to the solution. The resulting mixture was stirred at 25 °C for 3 hours. Then, the reaction mixture was filtered through a pad of 1:4 silica gel/Celite®. The pad was washed with CH_2Cl_2 and the filtrate was concentrated under reduced pressure. Column chromatography (5/1/1 hexanes/CH_2Cl_2/EtOAc) of the residue on silica gel provided 303 mg (51%) of (*M*)-**S6**.

R_f (5/1/1 hexanes/CH_2Cl_2/EtOAc) = 0.33.

The recorded spectral data were in accordance with the previously published values.[3]

(*M*)- **S6** HPLC analysis: > 99.5:0.5 e.r. (column Chiralpak IB (Heptane/*i*-PrOH = 90/10, flow rate 1 mL/min, temperature 25° C, UV 272 nm, t_{major} = 18.8 min; t_{min} = not detected).

[3] Cadart, T.: Nečas, D.; Kaiser, R. P.; Favereau, L.; Císařová, I.; Gyepes, R.; Hodačová, J.; Kalíková, K.; Bednárová, L.; Crassous, J.; Kotora, M. *Chem. Eur. J.* **2021**, *27*, 11279–11284.

Racemic	(R)-SEGPHOS®, > 99.5:0.5 e.r.

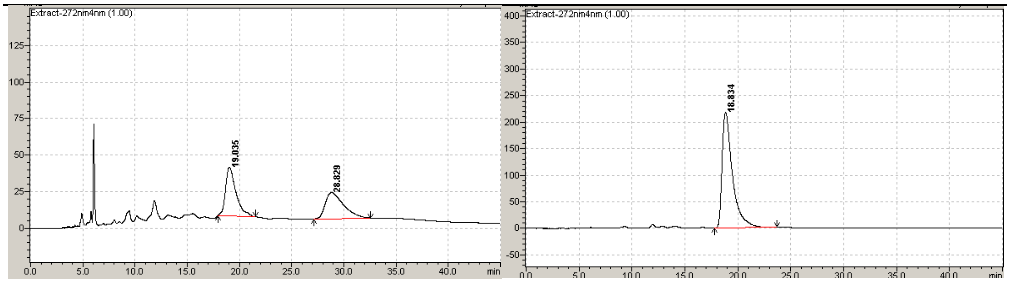

N°	Retention time (min)	Relative area (%)	Retention time (min)	Relative area (%)
1	19.0	53.3	18.8	>99
2	28.8	46.7	/	/

(*P*)-8,9-Bis(4-methoxyphenyl)benzo[*c*]benzo[6,7]indeno[1,2-*g*]fluorene-7,10-dione (S6)

A flame-dried microwave vial was loaded with Rh(cod)$_2$BF$_4$ (0.05 mmol, 20.3 mg) and (*S*)-SEGPHOS® (0.06 mmol, 36.6 mg) in dry CH_2ClCH_2Cl (10 mL) under an argon atmosphere. Hydrogen gas was bubbled into the reaction mixture for 45 minutes. Subsequently, the respective triynediol **S5** (1 mmol) was introduced under the argon atmosphere. The reaction was allowed to proceed at 60 °C for 48 hours. Afterward, the reaction mixture was concentrated under reduced pressure. The crude diols were directly oxidized to the corresponding diketone **S6** without additional purification steps. The crude diols were dissolved in CH_2Cl_2 (100 mL) and pyridinium chlorochromate (3.0 mmol, 647 mg) and Celite® (700 mg) were added to the solution. The resulting mixture was stirred at 25 °C for 3 hours. Then, the reaction mixture was filtered through a pad of 1:4 silica gel/Celite®. The pad was washed with CH_2Cl_2 and the filtrate was concentrated under reduced pressure. Column chromatography (5/1/1 hexanes/CH_2Cl_2/EtOAc) of the residue on silica gel provided 322 mg (54%) of (*P*)- **S6**.

R_f (5/1/1 hexanes/CH_2Cl_2/EtOAc) = 0.33.

The recorded spectral data were in accordance with the previously published values.[3]

(*P*)- **S6** HPLC analysis: 0.6:99.4 e.r. (column Chiralpak IB (Heptane/*i*-PrOH = 90/10, flow rate 1 mL/min, temperature 25° C, UV 272 nm, t_{major} = 27.5 min; t_{min} = 19.2 min).

Racemic	(S)-SEGPHOS®, 0.6:99.4 e.r.

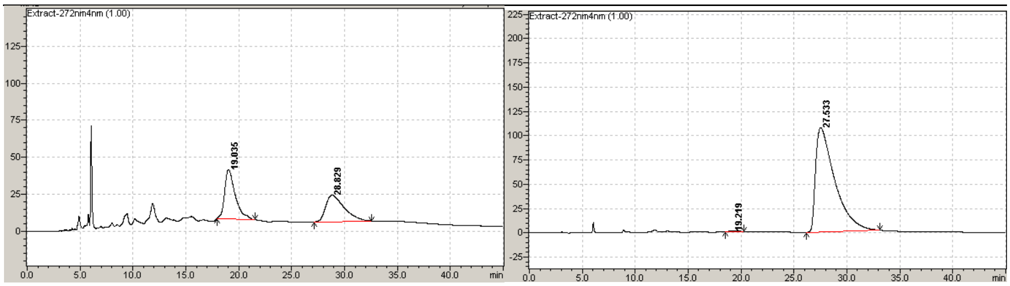

N°	Retention time (min)	Relative area (%)	Retention time (min)	Relative area (%)
1	19.0	53.3	19.2	0.6
2	28.8	46.7	27.5	99.4

(*M*)-8,9-Bis(4-methoxyphenyl)-7,10-di-*p*-tolyl-7,10 dihydrobenzo[*c*]benzo[6,7] indeno[1,2-*g*]fluorene-7,10-diol (8)

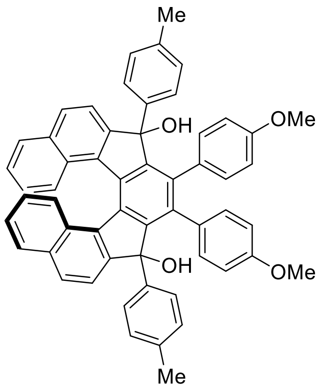

With 4-bromotoluene (0.8 mmol, 0.1 mL) and the diketone (*M*)- **S6** (0.2 mmol, 113 mg) following the general procedure **A**. Column chromatography of the residue on silica gel (5/1 hexanes/EtOAc) provided 105 mg (67%) of the title compound as a bright yellow solid.

The recorded data for (*M*)-**8** agree with those for *rac*-**8** (Chapter 3).

(*P*)-8,9-Bis(4-methoxyphenyl)-7,10-di-*p*-tolyl-7,10 dihydrobenzo[*c*]benzo[6,7] indeno[1,2-*g*]fluorene-7,10-diol (8)

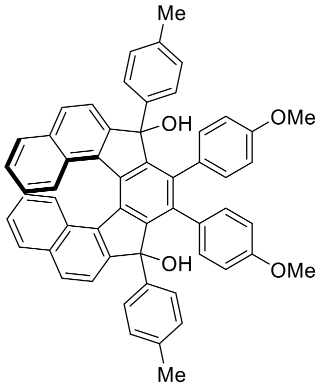

With 4-bromotoluene (2 mmol, 0.24 mL) and the diketone (*P*)- **S6** (0.51 mmol, 303 mg) following the general procedure **A**. Column chromatography of the residue on silica gel (5/1 hexanes/EtOAc) provided 330 mg (83%) of the title compound as a bright yellow solid.

The recorded data for (*P*)-**8** agree with those for *rac*-**8** (Chapter 3).

4 Synthesis of diaza[n]helicenes

4.1 Reaction conditions assessment

Experimental procedure for Schmidt rearrangement on analytical scale

The corresponding diols (0.1 mmol) and an *azide* (0.6 mmol) were dissolved in a *solvent* (1 mL). After adding the *acid* (11-13 eq.), the reaction vial was sealed and stirred at 60 °C in a heat transfer block (stirring 1000 rpm) for 4.5 hours. The reaction mixture was cooled down to 25 °C, quenched with a saturated aqueous solution of K_2CO_3, and extracted with EtOAc (3×10 mL). The combined organic layer was dried over Na_2SO_4, filtered, and concentrated under reduced pressure. Column chromatography of the residue on silica gel (toluene/Et_2O or hexanes/EtOAc/ with 1% Et_3N) provided certain products.

Note: Although organic azides are known to be generally unstable compounds, we neither experienced any problems during the reactions where azides were formed, nor with their thermal instability during their isolation and storage.

Table S2. Schmidt rearrangement of **1a** under different reaction conditions.

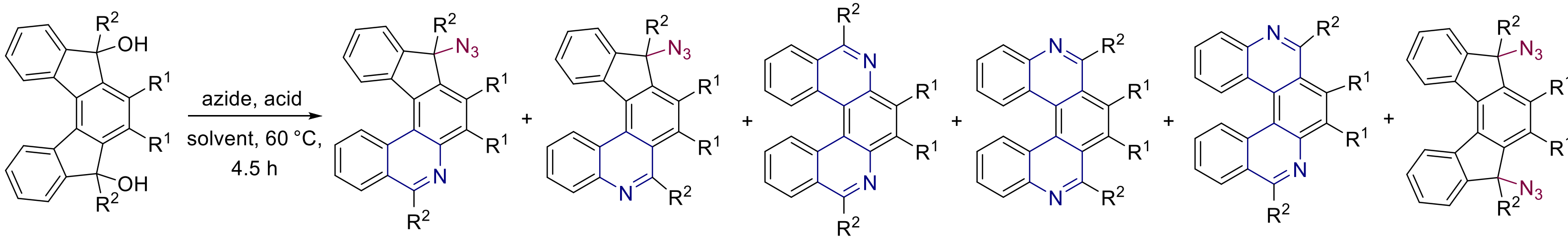

Entry	Azide	Acid	pK_a[a]	Solvent	**2a** (%)[b]	**3a** (%)[b]	**4a** (%)[b]	**5a** (%)[b]	**6a** (%)[b]	**7a** (%)[b]	Mixed fraction (%)[c]	Yield (%)	**4a/5a/6a**[d]
1	LiN_3	CF_3COOH	0	HFIP	64	21	-	-	-	-	-	85	-
2	NaN_3	CF_3COOH	0	HFIP	70	17	-	-	-	-	-	87	-
3	DPPA	CF_3COOH	0	HFIP	-	-	-	-	-	-	-	-	-[e]
4	LiN_3	$PhSO_3H$	-2.5	HFIP	-	-	18	-	-	-	70	88	1:1.5:3.4
5	NaN_3	$PhSO_3H$	-2.5	HFIP	-	-	19	23	40	-	-	82	1:1.7:3
6	DPPA	$PhSO_3H$	-2.5	HFIP	-	-	-	-	-	-	-	-	-[e]
7	NaN_3	MsOH	-2	HFIP	-	-	16	19	24	-	14	73	1:1.3:3
8	NaN_3	*p*-TsOH	-2.8	HFIP	-	-	19	21	41	-	-	81	1:1.5:3
9	NaN_3	H_2SO_4	-3	HFIP	-	-	-	48	-	-	20	68	1:12:3
10	NaN_3	HCl	-8	HFIP	-	-	20	23	45	-	-	88	1:1.2:3.5
11	NaN_3	TfOH	-13	HFIP	-	-	4	68	-	-	25	97	1:11:1.5
12	NaN_3	HNO_3	-2	HFIP	-	-	-	-	-	-	-	-	-
13	NaN_3	H_2SO_4	-3	HFIP	-	-	traces	48	-	-	20	68	1:12:3
14	NaN_3	HCl[f]	-8	HFIP	-	-	-	-	-	-	-	-	-[e]
15	NaN_3	H_3PO_4	2.12	HFIP	-	-	-	-	-	62	-	62	-
16	NaN_3	HBr	-9	HFIP	-	-	-	-	-	-	-	-	-
17	NaN_3	$PhSO_3H$	-2.5	MeCN	-	-	-	-	-	78	-	78	-

18	NaN_3	$PhSO_3H$	-2.5	DMSO	-	-	-	-	-	-	-	-	SM
19	NaN_3	$PhSO_3H$	-2.5	DMF	-	-	-	-	-	-	-	-	SM
20	NaN_3	$PhSO_3H$	-2.5	toluene	-	-	-	-	-	65	-	65	-
21	NaN_3	H_2SO_4	-3	toluene	-	-	7	-	15	15	15	52	1:1:2
22	NaN_3	TfOH	-14	$CHCl_3$	-	-	traces	45	16	-	-	61	1:10:2
23	NaN_3	TfOH	-14	toluene	-	-	-	-	-	-	-	-	-[e]
24	NaN_3	TfOH	-14	MeCN	-	-	19	-	-	-	50	69	1:2:3
25	NaN_3	TfOH	-14	*i*-PrOH	-	-	-	-	-	86	-	86	-
26	NaN_3	TfOH	-14	TFE	-	-	12	19	37	-	19	87	1:2:4
27	NaN_3	TfOH (3eq.)	-14	HFIP	-	-	6	20	2	-	55	82	1:10:2

Before handling azides, read: ”Treitler, D. S; Leung, S. How Dangerous Is Too Dangerous? A Perspective on Azide Chemistry. *J. Org. Chem.* **2022**, *87*, 11293–11295.

All reactions were performed on 0.1 mmol scale with 6 eq. of azide and 13 eq. of acid (11 eq. of TfOH), unless otherwise noted.

[a] pKa in water. Data are taken from: Ionization Constants of Organic Acids – MSU Chemistry. https://www2.chemistry.msu.edu/faculty/reusch/virttxtjml/acidity2.htm.

[b] Isolated yields

[c] Mixture of products **5a** and **6a**

[d] Approximate product ratio calculated from ^{1}H NMR of crude mixtures

[e] Too complex reaction mixture, products not detected

[f] Solution of HCl in dioxane.

SM = Starting material

Details of experimental procedures when products were isolated

Procedure A. Schmidt rearrangement using trifluoroacetic acid (Table S2, Entry 2)

Diols **1a** (0.1 mmol, 68 mg) and NaN_3 (0.6 mmol, 39 mg) were dissolved in HFIP (1 mL). After adding trifluoroacetic acid (1.3 mmol, 0.1 mL), the reaction vial was sealed and stirred at 60 °C in a heat transfer block (stirring 1000 rpm) for 4.5 hours. The reaction mixture was cooled down to 25 °C, quenched with a saturated aqueous solution of K_2CO_3, and extracted with EtOAc (3×10 mL). The combined organic layer was dried over Na_2SO_4, filtered, and concentrated under reduced pressure. Column chromatography of the residue on silica gel (15/1 hexanes/EtOAc) provided 49 mg (70%) of compound **2a** and 12 mg (17%) of compound 3**a**.

Procedure B. Schmidt rearrangement using benzenesulfonic acid (Table S2, Entry 5)

Diols **1a** (0.1 mmol, 68 mg) and NaN_3 (0.6 mmol, 39 mg) were dissolved in HFIP (1 mL). After adding benzenesulfonic acid (1.3 mmol, 206 mg), the reaction vial was sealed and stirred at 60 °C in a heat transfer block (stirring 1000 rpm) for 4.5 hours. The reaction mixture was cooled down to 25 °C, quenched with a saturated aqueous solution of K_2CO_3, and extracted with EtOAc (3×10 mL). The combined organic layer was dried over Na_2SO_4, filtered, and concentrated under reduced pressure. Column chromatography of the residue on silica gel (linear gradient: 35/1 to 25/1 toluene/Et_2O) provided 13 mg (19%) of compound **4a**, 15 mg (25%) of compound **5a**, and 27 mg (40%) of compound **6a**.

Procedure C. Schmidt rearrangement using triflic acid (Table S2, Entry 11)

Diols **1a** (0.1 mmol, 68 mg) and NaN_3 (0.6 mmol, 39 mg) were dissolved in HFIP (1 mL). After adding triflic acid (1.1 mmol, 0.1 mL), the reaction vial was sealed and stirred at 60 °C in a heat transfer block (stirring 1000 rpm) for 4.5 hours. The reaction mixture was cooled down to 25 °C, quenched with a saturated aqueous solution of K_2CO_3, and extracted with EtOAc (3×10 mL). The combined organic layer was dried over Na_2SO_4, filtered, and concentrated under reduced pressure. Column chromatography of the residue on silica gel (linear gradient: 35/1 to 25/1 toluene/Et_2O) provided 3 mg (4%) of compound **4a**, 46 mg (68%) of compound **5a**, and 17 mg (25%) of a mixture of compounds **5a** and **6a**.

Procedure D. Schmidt rearrangement using benzenesulfonic acid in MeCN (Table S2, Entry 17)

Diols **1a** (0.1 mmol, 68 mg) and NaN_3 (0.6 mmol, 39 mg) were dissolved in MeCN (1 mL). After adding benzenesulfonic acid (1.3 mmol, 206 mg), the reaction vial was sealed and stirred at 60 °C in a heat transfer block (stirring 1000 rpm) for 4.5 hours. The reaction mixture was cooled down to 25 °C, quenched with a saturated aqueous solution of K_2CO_3, and extracted with EtOAc (3×10 mL). The combined organic layer was dried over Na_2SO_4, filtered, and concentrated under reduced pressure. Column chromatography of the residue on silica gel (10/1 hexanes/EtOAc) provided 57 mg (78%) of compound **7a**.

Procedure E. Schmidt rearrangement using triflic acid in *i*-PrOH (Table S2, Entry 25)

Diols **1a** (0.1 mmol, 68 mg) and NaN_3 (0.6 mmol, 39 mg) were dissolved in *i*-PrOH (1 mL). After adding triflic acid (1.1 mmol, 0.1 mL), the reaction vial was sealed and stirred at 60 °C in a heat transfer block (stirring 1000 rpm) for 4.5 hours. The reaction mixture was cooled down to 25 °C, quenched with a saturated aqueous solution of K_2CO_3, and extracted with EtOAc (3×10 mL). The combined organic layer was dried over Na_2SO_4, filtered, and concentrated under reduced pressure. Column chromatography of the residue on silica gel (10/1 hexanes/EtOAc) provided 62 mg (86%) of compound **7a**.

9-Azido-7,8-bis(4-methoxyphenyl)-5,9-di-p-tolyl-9H-indeno[1,2-*a*]phenanthridine (2a) and 9-azido-7,8-bis(4-methoxyphenyl)-6,9-di-p-tolyl-9H-indeno[2,1-*k*]phenanthridine (3a)

With **1a** (0.3 mmol, 204 mg) following experimental procedure **A** (Table S2, Entry 2). Column chromatography of the residue on silica gel (15/1 hexanes/EtOAc) provided 153 mg (73%) of compound **2a** and 44 mg (21%) of compound **3a** as yellow solids.

2a

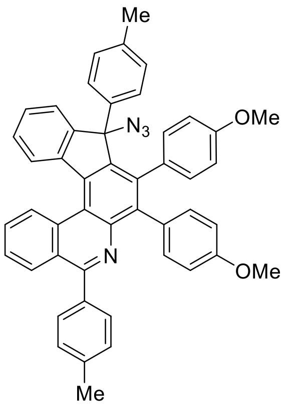

R_f (15/1 hexanes/EtOAc) = 0.20.

M.p. = 196 – 198 °C.

^{1}H NMR (400 MHz, $CDCl_3$, 25 °C) δ 9.21 (d, J = 7.2 Hz, 1H), 8.38 (d, J = 7.7 Hz, 1H), 8.27 (d, J = 8.2 Hz, 1H), 7.76 (ddd, J = 8.3, 7.0, 1.5 Hz, 1H), 7.71 – 7.65 (m, 3H), 7.35 (td, J = 7.6, 1.4 Hz, 1H), 7.29 – 7.24 (m, 3H), 7.18 (d, J = 7.6 Hz, 2H), 7.07 – 7.05 (m, 2H), 6.86 – 6.80 (m, 4H), 6.76 – 6.73 (m, 1H), 6.68 (d, J = 8.9 Hz, 2H), 6.16 – 6.14 (m, 1H), 5.86 – 5.84 (m, 1H), 3.76 (s, 3H), 3.74 (s, 3H), 2.44 (s, 3H), 2.25 (s, 3H).

^{13}C NMR (101 MHz, $CDCl_3$, 25 °C) δ 159.0, 157.8, 157.6, 149.9, 145.5, 143.7, 141.6, 140.4, 139.6, 139.0, 136.6, 136.3, 136.2, 134.9, 133.3, 133.2 (2C), 131.4, 131.2, 130.4 (2C), 130.3, 130.1, 129.0 (2C), 128.6 (2C), 128.4, 128.3, 128.24, 128.17, 127.8, 127.2, 125.6 (2C), 124.9, 124.8, 123.4, 119.8, 112.0 (2C), 111.9, 55.1, 55.0, 21.4, 21.0 (2 carbon signals are probably covered by other signals).

IR (KBr) ν_{max} 3122, 3060, 3024, 2999, 2951, 2929, 2858, 2833, 2100, 1610, 1577, 1516, 1464, 1427, 1356, 1288, 1248, 1176, 1109, 1036, 970, 930, 829, 808, 787, 764, 746, 723 cm^{-1}.

HRMS (ESI$^+$) m/z calcd for $C_{48}H_{37}N_4O_2$ [M + H]$^+$: 701.29110, found: 701.29091.

HRMS (ESI$^+$) m/z calcd for $C_{48}H_{37}N_2O_2$ [M + H]$^+$: 673.28496, found: 673.28474.

3a

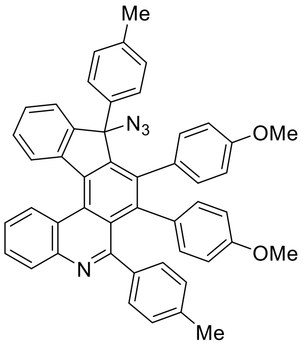

R_f (15/1 hexanes/EtOAc) = 0.08.

M.p. = 181 – 184 °C.

^{1}H NMR (400 MHz, $CDCl_3$, 25 °C) δ 9.09 (d, J = 8.1 Hz, 1H), 8.41 (d, J = 7.8 Hz, 1H), 8.19 (d, J = 8.3 Hz, 1H), 7.76 (t, J = 6.9 Hz, 1H), 7.57 (t, J = 6.9 Hz, 1H), 7.39 (t, J = 6.9 Hz, 1H), 7.30 (t, J = 6.9 Hz, 1H), 7.22 – 7.10 (m, 2H), 7.00 (d, J = 7.9 Hz, 2H), 6.88 – 6.74 (m, 6H), 6.70 (dd, J = 8.4, 2.8 Hz, 1H), 6.44 (dd, J = 8.5, 2.4 Hz, 1H), 6.33 (dd, J = 8.4, 2.3 Hz, 1H), 6.16 (td, J = 11.1, 2.8 Hz, 2H), 6.05 – 5.98 (m, 1H), 5.60 (d, J = 8.5 Hz, 1H), 3.69 (s, 3H), 3.57 (s, 3H), 2.23 (s, 3H), 2.19 (s, 3H).

^{13}C NMR (101 MHz, $CDCl_3$, 25 °C) δ 162.8, 157.9, 157.6, 149.6, 148.9, 143.8, 143.2, 140.5, 139.9, 139.6, 136.7, 136.5, 134.0, 133.4, 133.1, 132.2, 132.0, 131.5, 131.2, 129.4, 128.92,

128.86, 128.82, 128.77, 128.6 (2C), 128.5, 128.0, 127.5, 126.1, 125.7 (2C), 124.80, 124.77, 124.0, 122.5, 112.10, 112.06, 111.9, 111.7, 55.08, 55.06, 21.1, 21.00. (4 carbon signals are probably covered by other signals).

IR (KBr) ν_{max} 3033, 2997, 2951, 2933, 2868, 2883, 2098, 1608, 1512, 1286, 1246, 1178, 1036, 831, 760 cm^{-1}.

HRMS (ESI^+) m/z calcd for $C_{48}H_{37}N_4O_2$ $[M + H]^+$: 701.29110, found: 701.29254.

HRMS (ESI^+) m/z calcd for $C_{48}H_{37}N_2O_2$ $[M + H]^+$: 673.28496, found: 673.28623.

3,4-Bis(4-methoxyphenyl)-1,6-di-*p*-tolyldibenzo[*a*,*k*][4,7]phenanthroline (4a), 3,4-bis(4-methoxyphenyl)-2,5-di-*p*-tolyldibenzo[*a*,*k*][3,8]phenanthroline (5a) and 3,4-bis(4-methoxyphenyl)-2,6-di-*p*-tolyldibenzo[*a*,*k*][3,7]phenanthroline (6a)

With **1a** (0.1 mmol, 68 mg) following experimental procedure **B** (Table S2, Entry 5). Column chromatography of the residue on silica gel (linear gradient: 35/1 to 25/1 toluene/Et_2O) provided 13 mg (19%) of compound **4a**, 15 mg (25%) of compound 5**a**, and 27 mg (40%) of compound **6a** as yellow solids.

With **1a** (0.1 mmol, 68 mg) following experimental procedure **C** (Table S2, Entry 11). Column chromatography of the residue on silica gel (linear gradient: 35/1 to 25/1 toluene/Et_2O) provided 3 mg (4%) of compound **4a**, 46 mg (68%) of compound **5a**, and 17 mg (25%) of a mixture of compounds **5a** and **6a**.

4a

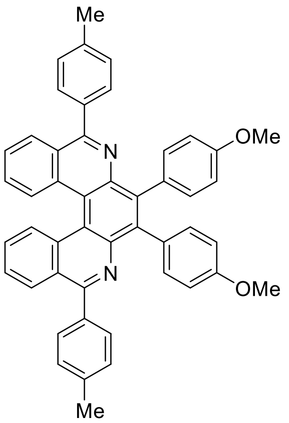

R_f (25/1 toluene/Et_2O) = 0.54.

M.p. = 281 – 283 °C.

^{1}H NMR (400 MHz, $CDCl_3$, 25 °C) δ 8.69 (dd, J = 8.3, 1.4 Hz, 2H), 8.30 (dd, J = 7.8, 1.4 Hz, 2H), 7.87 (d, J = 8.2 Hz, 4H), 7.57 (m, 4H), 7.33 (d, J = 7.8 Hz, 4H), 6.84 (br s, 4H), 3.83 (s, 6H), 2.45 (s, 6H) (Due to the restricted rotation of *p*-methoxyphenyl substituents, four hydrogens are missing (broad signals)).

^{13}C NMR (101 MHz, $CDCl_3$, 25 °C) δ 158.2 (2C), 158.0 (2C), 142.0 (2C), 140.5 (2C), 139.0 (2C), 136.4 (2C), 134.7 (2C), 131.0 (2C), 130.7 (4C), 129.1 (4C), 128.5 (2C), 128.0 (2C), 127.7 (2C), 127.0 (2C), 123.9 (2C), 118.3 (2C), 112.4 (4C), 55.2 (2C), 21.4 (2C) (4 carbon signals are probably covered by other signals).

IR (ATR) ν_{max} 3030, 2997, 2951, 2922, 2852, 2883, 1761, 1732, 1660, 1606, 1508, 1456, 1358, 1286, 1242, 1174, 1032, 972, 827, 764, 721, 667 cm^{-1}.

HRMS (ESI^+) m/z calcd for $C_{48}H_{37}N_2O_2$ $[M + H]^+$: 673.28496, found: 673.28561.

5a

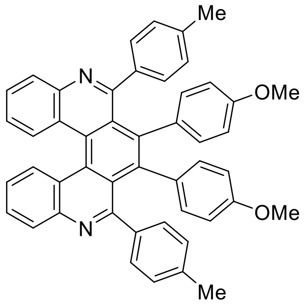

R_f (25/1 toluene/Et_2O) = 0.23.

M.p. = 292 – 294 °C.

^{1}H NMR (400 MHz, $CDCl_3$, 25 °C) δ 8.59 (d, J = 7.8 Hz, 2H), 8.21 (dd, J = 8.3, 1.3 Hz, 2H), 7.69 (ddd, J = 8.2, 6.9, 1.3 Hz, 2H), 7.38 (ddd, J = 8.4, 7.0, 1.4 Hz, 2H), 7.21 – 6.91 (m, 4H), 6.78 (br s, 4H), 6.69 – 6.67 (m, 2H), 6.38 (br s, 2H), 6.09 (br s, 2H), 6.01 (br s, 2H), 3.60 (s, 6H), 2.18 (s, 6H).

^{13}C NMR (101 MHz, $CDCl_3$, 25 °C) δ 160.9 (2C), 157.6 (2C), 143.3 (2C), 140.4 (2C), 139.9 (2C), 136.6 (2C), 133.5 (2C), 132.2 (2C), 131.0 (2C), 129.1 (4C), 128.8 (2C), 128.0 (4C), 127.5 (4C), 125.6 (4C), 125.5 (2C), 124.2 (2C), 112.4 (2C), 112.2 (2C), 55.1 (2C), 21.0 (2C).

IR (ATR) ν_{max} 3055, 3026, 2993, 2952, 2918, 2864, 2883, 1608, 1510, 1367, 1284, 1242, 1176, 1032, 978, 831, 812, 758, 737 cm^{-1}.

HRMS (ESI$^+$) m/z calcd for $C_{48}H_{37}N_2O_2$ [M + H]$^+$: 673.28496, found: 673.28553.

6a

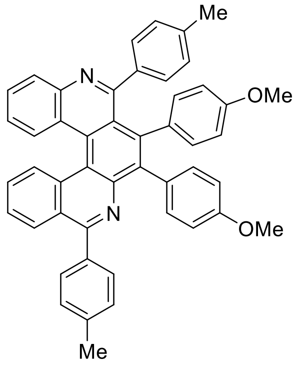

R_f (25/1 toluene/Et_2O) = 0.31.

M.p. = 186 – 188 °C.

^{1}H NMR (400 MHz, C_6D_6, 25 °C) δ 8.71 (d, J = 8.4 Hz, 1H), 8.64 (d, J = 8.4 Hz, 1H), 8.57 (dd, J = 8.2, 1.3 Hz, 1H), 8.30 (d, J = 8.1 Hz, 1H), 7.97 (d, J = 8.1 Hz, 2H), 7.48 – 7.41 (m, 2H), 7.22 – 7.07 (m, 8H,), 7.05 (d, J = 7.7 Hz, 2H), 6.77 (d, J = 8.4 Hz, 3H), 6.57 (dd, J = 8.4, 2.3 Hz, 1H), 6.39 (dd, J = 8.4, 2.7 Hz, 1H), 6.19 (dd, J = 8.6, 2.7 Hz, 1H), 3.21 (s, 3H), 3.13 (s, 3H), 2.12 (s, 3H), 2.01 (s, 3H).

^{13}C NMR (101 MHz, C_6D_6, 25 °C) δ 161.1, 160.3, 158.2, 158.0, 144.3, 143.9, 141.1, 140.8, 140.6, 138.8, 136.6, 136.4, 135.2, 134.1, 133.5, 133.2, 133.0, 131.1, 130.6 (2C), 130.2 (2C), 129.1 (2C), 129.0, 128.9, 128.8, 128.22, 128.15, 127.81, 127.76, 127.4, 127.2, 124.8, 124.5, 124.32, 124.28, 117.0, 112.7, 112.6, 54.2, 54.1, 20.9, 20.7 (4 carbon signals are probably covered by other signals and/or benzene-d$_6$ signals).

IR (ATR) ν_{max} 3122, 3060, 3030, 2955, 2951, 2922, 2852, 1608, 1510, 1454, 1365, 1288, 1244, 1174, 1032, 805, 762 cm^{-1}.

HRMS (ESI$^+$) m/z calcd for $C_{48}H_{37}N_2O_2$ [M + H]$^+$: 673.28496, found: 673.28561.

5,8-Diazido-6,7-bis(4-methoxyphenyl)-5,8-di-*p*-tolyl-5,8-dihydroindeno[2,1-*c*]fluorene (7a)

With **1a** (0.1 mmol, 68 mg) following experimental procedure **D** (Table S2, Entry 17). Column chromatography of the residue on silica gel (10/1 hexanes/EtOAc) provided 57 mg (78%) of compound **7a** (~3:1 inseparable mixture of diastereoisomers) as a bright yellow solid.

With **1a** (0.1 mmol, 68 mg) following experimental procedure **E** (Table S2, Entry 25). Column chromatography of the residue on silica gel (10/1 hexanes/EtOAc) provided 62 mg (86%) of compound **7a** (~3:1 inseparable mixture of diastereoisomers) as a bright yellow solid.

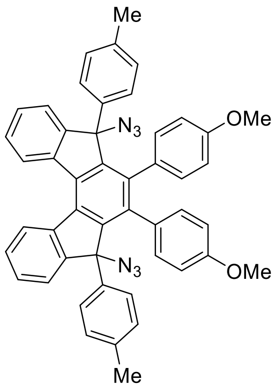

R_f (10/1 hexanes/EtOAc) = 0.19.

M.p. = 214 – 216 °C.

Major diastereoisomer: ^{1}H NMR (400 MHz, $CDCl_3$, 25 °C) δ 8.54 (d, J = 7.8 Hz, 2H), 7.52 (td, J = 7.6, 1.2 Hz, 2H), 7.33 (t, J = 7.5 Hz, 2H), 7.16 (d, J = 9.2 Hz, 2H), 7.00 (dd, J = 8.4, 2.2 Hz, 2H), 6.82 (d, J = 8.6 Hz, 4H), 6.72 – 6.69 (m, 4H), 6.61 (dd, J = 8.4, 2.7 Hz, 2H), 5.97 (dd, J = 8.6, 2.8 Hz, 2H), 5.61 (dd, J = 8.5, 2.2 Hz, 2H), 3.65 (s, 6H), 2.22 (s, 6H).

^{13}C NMR (101 MHz, $CDCl_3$, 25 °C) δ 157.6 (2C), 149.5 (2C), 147.8 (2C), 141.1 (2C), 139.1 (2C), 137.2 (2C), 136.2 (2C), 135.0 (2C), 130.93 (2C), 130.86 (2C), 129.1 (2C), 129.0 (2C), 128.8 (2C), 128.4 (4C), 125.6 (4C), 125.3 (2C), 123.6 (2C), 111.8 (2C), 111.6 (2C), 76.5 (2C), 55.0 (2C), 21.0 (2C).

Minor diastereoisomer: ^{1}H NMR (400 MHz, $CDCl_3$, 25 °C) δ 8.54 (d, J = 7.8 Hz, 2H), 7.52 (td, J = 7.6, 1.2 Hz, 2H), 7.33 (t, J = 7.5 Hz, 2H), 7.16 (d, J = 9.2 Hz, 2H), 6.90 (dd, J = 8.4, 2.2 Hz, 2H), 6.82 (d, J = 8.6 Hz, 4H), 6.72 – 6.69 (m, 4H), 6.56 (dd, J = 8.4, 2.7 Hz, 2H), 6.01 (dd, J = 8.6, 2.8 Hz, 2H), 5.72 (dd, J = 8.5, 2.2 Hz, 2H), 3.65 (s, 6H), 2.22 (s, 6H).

^{13}C NMR. Signals were covered by the major diastereoisomer.

IR (KBr) ν_{max} 3325, 3068, 3035, 3024, 2991, 2954, 2929, 2871, 2833, 2102, 1610, 1516, 1464, 1417, 1286, 1244, 1176, 1032, 930, 837, 800, 748 cm^{-1}.

HRMS (ESI$^+$) m/z calcd for $C_{48}H_{37}N_4O_2$ [M + H]$^+$: 701.29110, found: 701.28933.

HRMS (ESI$^+$) m/z calcd for $C_{48}H_{37}N_2O_2$ [M + H]$^+$: 673.28482, found: 673.28496.

4.2 Stepwise Schmidt rearrangement

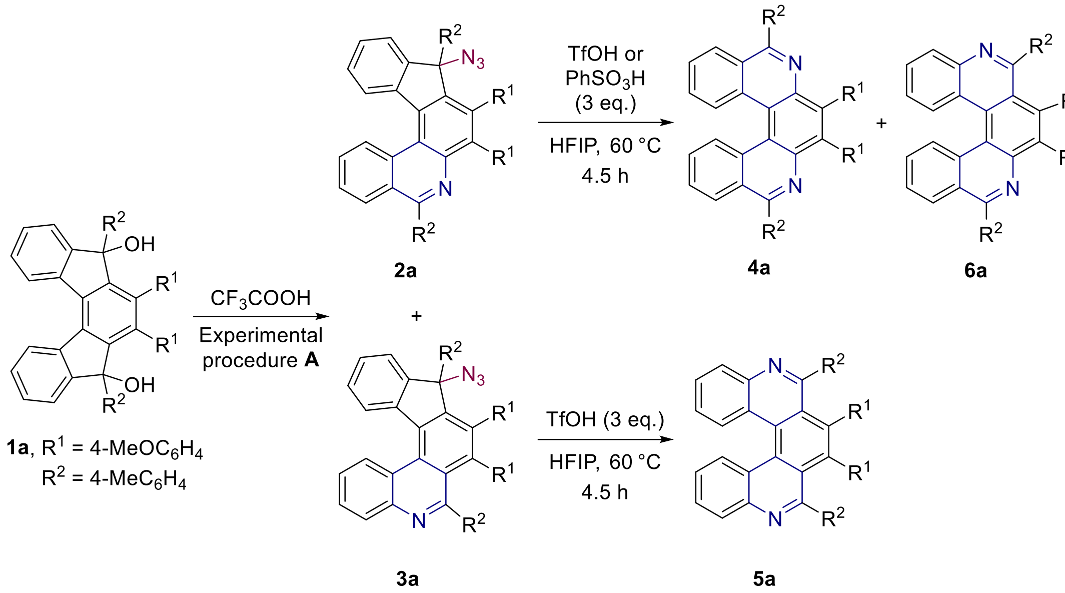

Scheme S3. Stepwise Schmidt rearrangement by using weaker acidic conditions (CF_3COOH) followed by stronger acidic conditions (TfOH or $PhSO_3H$).

9-Azido-7,8-bis(4-methoxyphenyl)-5,9-di-p-tolyl-9H-indeno[1,2-*a*]phenanthridine (2a) and 9-azido-7,8-bis(4-methoxyphenyl)-6,9-di-p-tolyl-9H-indeno[2,1-*k*]phenanthridine (3a)

With **1a** (0.3 mmol, 203 mg) following experimental procedure **A** (Table S2, Entry 2). Column chromatography of the residue on silica gel (15/1 hexanes/EtOAc) provided 154 mg (73%) of compound **2a** and 45 mg (21%) of compound **3a** as yellow solids.

The recorded NMR and other data are described in Chapter 4.1.

3,4-Bis(4-methoxyphenyl)-1,6-di-*p*-tolyldibenzo[*a*,*k*][4,7]phenanthroline (4a) 3,4-bis(4-methoxyphenyl)-2,6-di-*p*-tolyldibenzo[*a*,*k*][3,7]phenanthroline (6a)

Compound **2a** (0.1 mmol, 70 mg) was dissolved in HFIP (1 mL) and triflic acid (0.3 mmol, 0.03 mL) was added. The reaction vial was sealed and stirred at 60 °C in a heat transfer block (stirring 1000 rpm) for 4.5 hours. The reaction mixture was cooled down to 25 °C, quenched with a saturated aqueous solution of K_2CO_3, and extracted with EtOAc (3×10 mL). The combined organic layer was dried over Na_2SO_4, filtered, and concentrated under reduced pressure. Column chromatography of the residue on silica gel (10/1 hexanes/EtOAc) provided 13 mg (19%) of compound **4a** and 37 mg (55%) of compound **6a** as yellow solids.

Compound **2a** (0.1 mmol, 70 mg) was dissolved in HFIP (1 mL) and benzenesulfonic acid (0.3 mmol, 47 mg) was added. The reaction vial was sealed and stirred at 60 °C in a heat transfer block (stirring 1000 rpm) for 4.5 hours. The reaction mixture was cooled down to 25 °C, quenched with a saturated aqueous solution of K_2CO_3, and extracted with EtOAc (3×10 mL).

The combined organic layer was dried over Na_2SO_4, filtered, and concentrated under reduced pressure. Column chromatography of the residue on silica gel (10/1 hexanes/EtOAc) provided 16 mg (23%) of compound **4a** and 46 mg (68%) of compound **6a** as yellow solids.

R_f (**4a**, 10/1 hexanes/EtOAc) = 0.33.

R_f (**6a**, 10/1 hexanes/EtOAc) = 0.20.

The recorded NMR and other data are described in Chapter 4.1.

3,4-Bis(4-methoxyphenyl)-2,5-di-*p*-tolyldibenzo[*a*,*k*][3,8]phenanthroline (5a)

Compound **3a** (0.06 mmol, 45 mg) was dissolved in HFIP (0.6 mL) and triflic acid (0.2 mmol, 0.02 mL) was added. The reaction vial was sealed and stirred at 60 °C in a heat transfer block (stirring 1000 rpm) for 4.5 hours. The reaction mixture was cooled down to 25 °C, quenched with a saturated aqueous solution of K_2CO_3, and extracted with EtOAc (3×10 mL). The combined organic layer was dried over Na_2SO_4, filtered, and concentrated under reduced pressure. Column chromatography of the residue on silica gel (5/1 hexanes/EtOAc) provided 29 mg (71%) of compound **5a**.

R_f (10/1 hexanes/EtOAc) = 0.18.

The recorded NMR and other data are described in Chapter 4.1.

4.3 Thermally induced rearrangement

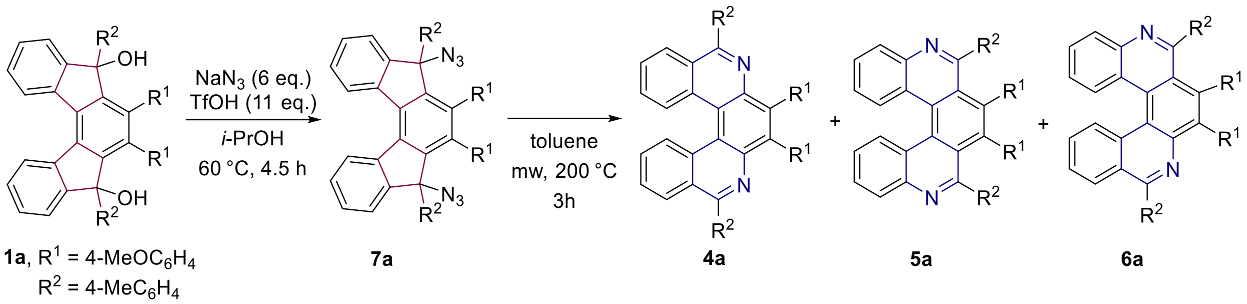

Scheme S4. Thermally induced rearrangement of **7a**.

5,8-Diazido-6,7-bis(4-methoxyphenyl)-5,8-di-*p*-tolyl-5,8-dihydroindeno[2,1-*c*]fluorene (7a)

With **1a** (0.2 mmol, 135 mg) following experimental procedure **E** (Table S2, Entry 25). Column chromatography of the residue on silica gel (10/1 hexanes/EtOAc) provided 139 mg (95%) of compound **7a** (~3:1 inseparable mixture of diastereoisomers) as a bright yellow solid.

R_f (10/1 hexanes/EtOAc) = 0.19.

The recorded NMR and other data are described in Chapter 4.1.

3,4-Bis(4-methoxyphenyl)-1,6-di-*p*-tolyldibenzo[*a*,*k*][4,7]phenanthroline (4a), 3,4-bis(4-methoxyphenyl)-2,5-di-*p*-tolyldibenzo[*a*,*k*][3,8]phenanthroline (5a) and 3,4-bis(4-methoxyphenyl)-2,6-di-*p*-tolyldibenzo[*a*,*k*][3,7]phenanthroline (6a)

In a microwave vial compound **7a** (0.1 mmol, 72 mg) was dissolved in dry toluene. The reaction vial was sealed and stirred at 200 °C in the microwave reactor for 3 hours (stirring 600 rpm). The solvent was removed under reduced pressure and column chromatography of the residue on silica gel (linear gradient: 35/1 to 25/1 toluene/Et_2O) provided 34 mg (50%) of compound **4a**, 7 mg (10%) of compound **5a**, and 24 mg (35%) of compound **6a** as yellow solids.

Approximate product ratio calculated from ^{1}H NMR **4a/5a/6a** =5.5:1:3.5

R_f (**4a**, 25/1 toluene/Et_2O) = 0.54.

R_f (**5a**, 25/1 toluene/Et_2O) = 0.23.

R_f (**6a**, 25/1 toluene/Et_2O) = 0.31.

The recorded NMR and other data are described in Chapter 4.1.

4.4 Synthesis of diaza[5]helicenes

3,4-Bis(4-methoxyphenyl)-1,6-di-*p*-tolyldibenzo[*a*,*k*][4,7]phenanthroline (4a), 3,4-bis(4-methoxyphenyl)-2,5-di-*p*-tolyldibenzo[*a*,*k*][3,8]phenanthroline (5a) and 3,4-bis(4-methoxyphenyl)-2,6-di-*p*-tolyldibenzo[*a*,*k*][3,7]phenanthroline (6a)

With **1a** (1.0 mmol, 682 mg) following experimental procedure **C** (Table S2, Entry 11). Column chromatography of the residue on silica gel (linear gradient: 35/1 to 25/1 toluene/Et_2O) provided traces of compound **4a**, 366 mg (54%) of compound **5a**, and 98 mg (15%) of a mixture of compounds **5a** and **6a** as yellow solids.

The recorded NMR and other data are described in Chapter 4.1.

3,4-Bis(4-methoxyphenyl)-1,6-di-*o*-tolyldibenzo[*a*,*k*][4,7]phenanthroline (4b) and 3,4-bis(4-methoxyphenyl)-2,6-di-*o*-tolyldibenzo[*a*,*k*][3,7]phenanthroline (6b)

With **1b** (0.5 mmol, 339 mg) following experimental procedure **C** (Table S2, Entry 11). Column chromatography of the residue on silica gel (linear gradient: 50/1 to 30/1 toluene/Et_2O with 1% of Et_3N) provided 48 mg (14%) of compound **4b** and 53 mg (16%) of compound **6b** as yellow solids.

4b

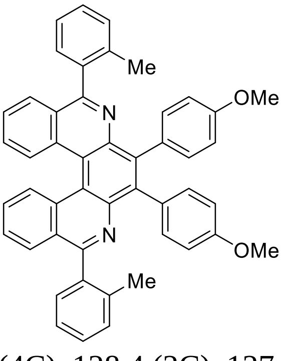

R_f (50/1 toluene/Et_2O) = 0.35.

M.p. = 289 – 291 °C.

^{1}H NMR (400 MHz, $CDCl_3$, 25 °C) δ 8.68 – 8.66 (m, 2H), 7.85 – 7.83 (m, 2H), 7.57 – 7.51 (m, 5H), 7.39 – 7.31 (m, 7H), 7.15 (br s, 4H), 6.76 (br s, 4H), 3.78 (s, 6H), 2.31 (br s, 6H).

^{13}C NMR (101 MHz, $CDCl_3$, 25 °C) δ 159.8 (2C), 157.8 (2C), 142.3 (2C), 141.0 (2C), 133.9 (2C), 131.2 (2C), 131.0 (2C), 130.7 (2C), 128.5 (4C), 128.4 (2C), 127.8 (2C), 127.6 (4C), 127.1 (2C), 125.4 (2C), 118.7 (2C), 112.4 (8C), 55.1 (2C), 20.7 (2C) (2 carbon signals are probably covered by other signals).

IR (ATR) ν_{max} 3059, 3018, 2993, 2952, 2927, 2831, 1606, 1508, 1456, 1286, 1242, 1173, 1107, 1032, 972, 837, 793, 762, 725 cm^{-1}.

HRMS (ESI$^+$) m/z calcd for $C_{48}H_{37}N_2O_2$ $[M + H]^+$: 673.28496, found: 673.28557.

6b

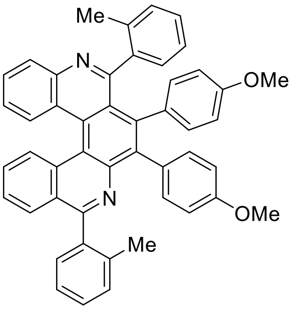

R_f (50/1 toluene/Et_2O) = 0.13.

M.p. = 336 – 338 °C.

^{1}H NMR (400 MHz, $CDCl_3$, 25 °C) δ 8.60 (d, *J* = 8.4 Hz, 2H), 8.24 – 8.18 (m, 2H), 7.71 (td, *J* = 7.0, 1.3 Hz, 2H), 7.41 (t, *J* = 7.7 Hz, 2H), 7.07 – 7.04 (m, 1H), 6.91 – 6.86 (m, 4H), 6.83 – 6.77 (m, 1H), 6.72 – 6.57 (m, 4H), 6.41 – 6.39 (m, 2H), 6.11 (ddd, *J* = 10.9, 8.5, 2.3 Hz,

1H), 6.03 – 5.97 (m, 1H), 5.92 (td, J = 8.7, 2.7 Hz, 2H), 3.60 (s, 3H), 3.59 (s, 3H), 2.31 (s, 3H), 2.29 (s, 3H).

Major atropoisomer: ^{13}C NMR (101 MHz, $CDCl_3$, 25 °C) δ 160.82, 160.58, 157.66, 157.21, 143.72, 143.29, 142.59, 142.12, 140.42, 140.13, 135.08, 134.27, 133.18, 132.47, 131.78, 131.32, 131.30, 131.16, 130.62, 130.50 (2C), 130.27, 129.82, 129.61, 129.15, 129.09 (2C), 128.84, 128.64, 127.75, 127.66, 127.42, 127.22, 126.91, 126.22, 125.70 (2C), 125.57, 124.50, 124.04, 123.83, 112.58 (2C), 112.08, 111.48, 55.15, 20.79, 20.25.

Minor atropoisomer: ^{13}C NMR (101 MHz, $CDCl_3$, 25 °C) δ 160.88, 160.62, 157.70, 157.26, 143.70, 143.29, 142.53, 142.07, 140.48, 140.18, 135.08, 134.31, 133.07, 132.38, 131.69, 131.30, 131.28, 131.23, 130.69, 130.50 (2C), 130.31, 129.82, 129.56, 129.13, 129.09 (2C), 128.87, 128.68, 127.80, 127.72, 127.47, 127.26, 126.98, 126.37, 125.70 (2C), 125.66, 124.55, 124.12, 123.87, 112.48 (2C), 112.11, 111.54, 55.12, 20.74, 20.25.

IR ν_{max} (ATR) 3099, 3057, 3010, 2956, 2922, 2852, 2833, 1608, 1510, 1363, 1246, 1174, 1030, 978, 791, 758, 723, 617 cm^{-1}.

HRMS (ESI$^+$) m/z calcd for $C_{48}H_{37}N_2O_2$ [M + H]$^+$: 673.28496, found: 673.28560.

1,6-Dibutyl-3,4-bis(4-methoxyphenyl)dibenzo[*a*,*k*][4,7]phenanthroline (4c) and 2,6-dibutyl-3,4-bis(4-methoxyphenyl)dibenzo[*a*,*k*][3,7]phenanthroline (6c)

With **1c** (0.1 mmol, 61 mg) following experimental procedure **C** (Table S2, Entry 11). Column chromatography of the residue on silica gel (15/1 hexanes/EtOAc with 1% of Et_3N) provided 6 mg (10%) of compound **4c** and 18 mg (30%) of compound **6c** as yellow solids.

4c

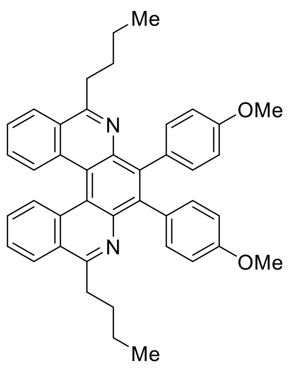

R_f (15/1 hexanes/EtOAc) = 0.32.

^{1}H NMR (400 MHz, $CDCl_3$, 25 °C) δ 8.49 (d, J = 8.4 Hz, 2H), 8.25 (d, J = 8.2 Hz, 2H), 7.61 (t, J = 7.0 Hz, 2H), 7.46 (ddd, J = 8.4, 6.9, 1.3 Hz, 2H), 7.18 (d, J = 6.5 Hz, 4H), 6.80 (d, J = 8.9 Hz, 4H), 3.82 (s, 6H), 3.39 (dt, J = 14.9, 7.5 Hz, 2H), 3.27 (dt, J = 15.0, 7.3 Hz, 2H), 1.87 (p, J = 7.4 Hz, 4H), 1.48 – 1.39 (m, 4H), 0.94 (t, J = 7.3 Hz, 6H).

^{13}C NMR (101 MHz, $CDCl_3$, 25 °C) δ 159.8 (2C), 157.6 (2C), 142.0 (2C), 140.1 (2C), 133.6 (2C), 133.2 (2C), 131.2 (2C), 128.0 (2C), 127.8 (2C), 126.9 (2C), 124.9 (2C), 124.8 (2C), 118.5 (2C), 114.5 (2C), 112.2 (4C), 55.1 (2C), 34.8 (2C), 29.9 (2C), 22.7 (2C), 14.2 (2C).

6c

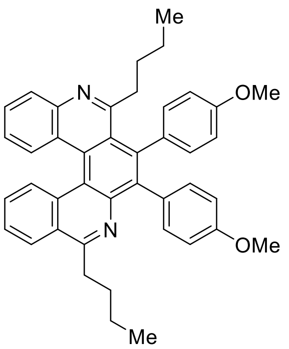

R$_f$ (15/1 hexanes/EtOAc) = 0.16.

M.p. = 174 – 176 °C.

^{1}H NMR (400 MHz, C_6D_6, 25 °C) δ 8.64 (d, J = 8.4 Hz, 1H), 8.46 (dd, J = 8.3, 1.4 Hz, 1H), 8.39 (dd, J = 8.2, 1.2 Hz, 1H), 7.95 (dd, J = 8.3, 1.2 Hz, 1H), 7.43 (ddd, J = 8.2, 6.9, 1.4 Hz, 1H), 7.24 (ddd, J = 8.3, 6.9, 1.2 Hz, 1H), 7.20 – 7.16 (m, 4H), 7.10 (ddd, J = 8.4, 6.9, 1.3 Hz, 1H), 7.02 (ddd, J = 8.4, 7.0, 1.4 Hz, 1H), 6.80 – 6.77 (m, 2H), 6.64 (ddd, J = 8.7, 6.0, 2.5 Hz, 2H), 3.27 (s, 3H), 3.19 (s, 3H), 3.11 (dt, J = 14.9, 7.3 Hz, 1H), 2.96 – 2.88 (m, 1H), 2.71 – 2.63 (m, 1H), 1.87 – 1.83 (m, 4H), 1.36 – 1.31 (m, 3H), 1.16 (dt, J = 14.1, 7.2 Hz, 2H), 0.88 (t, J = 7.3 Hz, 3H), 0.77 (t, J = 7.5 Hz, 3H).

^{13}C NMR (101 MHz, C_6D_6, 25 °C) δ 162.8, 161.8, 158.8, 158.1, 144.1, 143.9, 140.7, 139.5, 133.8, 133.6, 133.2, 132.2, 132.0, 131.7, 131.4, 128.5, 128.4, 128.3, 128.2, 128.1, 127.6, 127.0, 125.1, 125.0, 124.8, 124.21, 124.17, 117.5, 113.3, 113.11, 113.07, 113.0, 54.22, 54.15, 39.3, 34.8, 31.8, 29.9, 22.8, 22.6, 14.0, 13.8.

IR (ATR) ν_{max} 3059, 3032, 3010, 2952, 2929, 2870, 2858, 2831, 1606, 1510, 1456, 1286, 1240, 1174, 1030, 760, 553 cm^{-1}.

HRMS (ESI$^+$) m/z calcd for $C_{42}H_{41}N_2O_2$ [M + H]$^+$: 605.31626, found: 605.31564.

3,4-Bis(4-methoxyphenyl)-1,6-bis(4-(trifluoromethyl)phenyl)dibenzo[*a,k*][4,7]phenanthroline (4d), 3,4-bis(4-methoxyphenyl)-2,5-bis(4-(trifluoromethyl)phenyl)dibenzo[*a,k*][3,8] phenanthroline (5d) and 3,4-bis(4-methoxyphenyl)-2,6-bis(4-(trifluoromethyl)phenyl)dibenzo[*a,k*][3,7]phenanthroline (6d)

With **1d** (0.9 mmol, 708 mg) following experimental procedure **C** (Table S2, Entry 11). Column chromatography of the residue on silica gel (50/1 toluene/Et_2O with 1% of Et_3N) provided 42 mg (6%) of compound **4d**, 61 mg (9%) of compound **5d**, and 305 mg (43%) of a mixture of compounds **5d** and **6d** as yellow solids.

4d

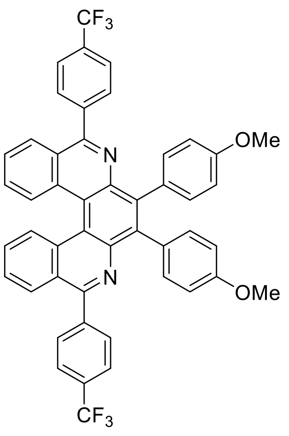

R$_f$ (50/1 toluene/Et_2O) = 0.52.

M.p. = 343 – 345 °C.

^{1}H NMR (400 MHz, $CDCl_3$, 25 °C) δ 8.72 (dd, J = 7.5, 2.3 Hz, 2H), 8.25 – 8.22 (m, 2H), 8.09 (d, J = 7.9 Hz, 4H), 7.80 (d, J = 8.1 Hz, 4H), 7.66 – 7.59 (m, 4H), 7.21 (br s, 4H), 6.86 (br s, 4H), 3.84 (s, 6H).

^{13}C NMR (101 MHz, $CDCl_3$, 25 °C) δ 158.2 (2C), 157.1 (2C), 142.6 (2C), 142.2 (2C), 140.9 (2C), 134.7 (2C), 133.7 (br s, 2C), 130.93 (4C), 130.92 (q, J = 32.3 Hz, 2C), 130.6 (2C), 129.0 (2C), 127.8 (2C), 127.6 (2C), 127.3 (2C), 125.4 (q, J = 3.5 Hz, 4C), 124.2 (q, J = 272 Hz, 2C),

123.8 (2C), 118.7 (2C), 112.5 (4C), 55.2 (2C) (2 carbon signals are probably covered by other signals).

^{19}F NMR (376.5 MHz, $CDCl_3$) δ -62.56.

IR ν_{max} (ATR) 3062, 3001, 2954, 2933, 2910, 2835, 1608, 1508, 1321, 1244, 1161, 1120, 1107, 1065, 1016, 849, 766, 661 cm^{-1}.

HRMS (ESI^+) m/z calcd for $C_{48}H_{31}F_6N_2O_2$ $[M + H]^+$: 781.22842, found: 781.22770.

5d

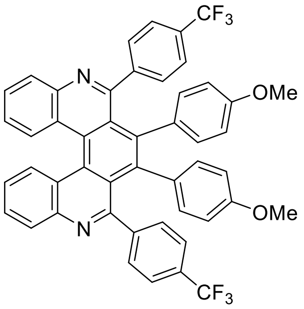

R$_f$ (50/1 toluene/Et_2O) = 0.14.

M.p. = 273 – 275 °C.

^{1}H NMR (400 MHz, $CDCl_3$, 25 °C) δ 8.61 (d, *J* = 8.4 Hz, 2H), 8.23 (dd, *J* = 8.3, 1.3 Hz, 2H), 7.77 – 7.73 (m, 2H), 7.47 – 7.43 (m, 2H), 7.27 (br s, 8H), 6.69 (dd, *J* = 8.4, 2.2 Hz, 2H), 6.40 (dd, *J* = 8.4, 2.7 Hz, 2H), 6.11 (dd, *J* = 8.6, 2.2 Hz, 2H), 6.02 (dd, *J* = 8.6, 2.7 Hz, 2H), 3.57 (s, 6H).

^{13}C NMR (101 MHz, $CDCl_3$, 25 °C) δ 159.2 (2C), 158.0 (2C), 146.4 (2C), 143.2 (2C), 139.7 (2C), 133.6 (2C), 133.5 (2C), 131.3 (2C), 131.2 (2C), 129.8 (br s, 2C), 129.6 (4C), 128.9 (4C), 127.5 (4C), 126.4 (4C), 125.4 (2C), 124.2 (br s, 2C), 124.1 (q, *J* = 272 Hz, 2C), 113.3 (2C), 112.1 (2C), 54.9 (2C).

^{19}F NMR (376.5 MHz, $CDCl_3$) δ -62.87.

IR ν_{max} (ATR) 3068, 3001, 2956, 2933, 2841, 1608, 1510, 1321, 1244, 1161, 1119, 1107, 1063, 1018, 837, 762 cm^{-1}.

HRMS (ESI^+) m/z calcd for $C_{48}H_{31}F_6N_2O_2$ $[M + H]^+$: 781.22842, found: 781.22824.

6d. It was obtained as a mixture with **5d**.

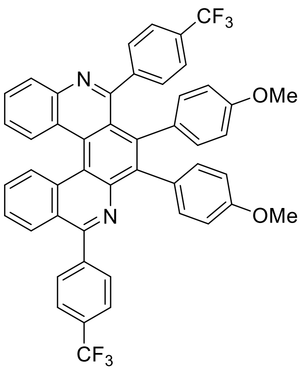

R$_f$ (50/1 toluene/Et_2O) = 0.16.

^{1}H NMR (400 MHz, $CDCl_3$, 25 °C) δ 8.78 (d, *J* = 7.5 Hz, 1H), 8.57 (d, *J* = 8.4 Hz, 1H), 8.06 (d, *J* = 8.3 Hz, 2H), 7.68 – 7.58 (m, 2H), 6.73 (dd, *J* = 8.4, 2.3 Hz, 2H), 6.49 – 6.45 (m, 2H), 6.18 (dd, *J* = 8.6, 2.8 Hz, 1H), 3.77 (s, 3H), 3.62 (s, 3H) (other signals are covered by signals from regioisomer **5d**).

^{13}C NMR (101 MHz, $CDCl_3$, 25 °C) δ 159.7, 159.1, 158.1, 143.5, 143.3, 142.4, 140.6, 140.2, 134.9, 133.9, 133.0, 132.2, 130.1, 129.6, 129.3, 128.9, 128.6, 128.3, 127.99, 127.95, 127.4, 127.3, 125.8, 124.4, 124.2, 123.9, 122.8, 117.4, 113.5, 112.4, 55.1, 55.0 (other signals are covered by signals from regioisomer **5d**).

^{19}F NMR (376 MHz, $CDCl_3$, 25 °C) δ -62.61, -62.85.

1,6-Di-*p*-tolyl-3,4-bis(4-(trifluoromethyl)phenyl)dibenzo[*a*,*k*][4,7]phenanthroline (4e), 2,5-di-*p*-tolyl-3,4-bis(4-(trifluoromethyl)phenyl)dibenzo[*a*,*k*][3,8]phenanthroline (5e) and 2,6-di-*p*-tolyl-3,4-bis(4-(trifluoromethyl)phenyl)dibenzo[*a*,*k*][3,7]phenanthroline (6e)

With **1e** (0.5 mmol, 377 mg) following experimental procedure **C** (Table S2, Entry 11). Column chromatography of the residue on silica gel (linear gradient: 50/1 to 30/1 hexanes/EtOAc with 1% of Et_3N) provided 30 mg (8%) of compound **4e**, 51 mg (14%) of compound **5e** and 95 mg (25%) of a mixture of compounds **5e** and **6e** as yellow solids.

4e

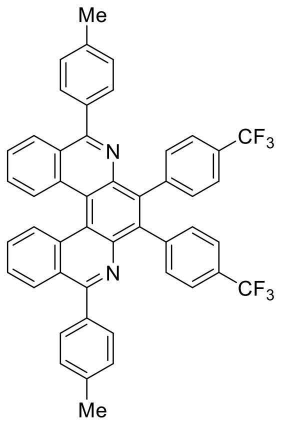

R_f (50/1 hexanes/EtOAc) = 0.12.

M.p. = 307 – 309 °C.

^{1}H NMR (400 MHz, $CDCl_3$, 25 °C) δ 8.75 (d, J = 8.0 Hz, 2H), 8.34 (d, J = 8.0 Hz, 2H), 7.80 (d, J = 7.8 Hz, 4H), 7.76 – 7.43 (m, 8H), 7.34 (d, J = 7.8 Hz, 4H), 2.46 (s, 6H) (Due to the restricted rotation of 4-(trifluoromethyl)phenyl substituents, four hydrogens are missing (broad signals)).

^{13}C NMR (101 MHz, $CDCl_3$, 25 °C) δ 158.9 (2C), 142.03 (2C), 142.02 (2C), 141.2 (2C), 139.50 (2C), 139.48 (2C), 135.9 (2C), 134.5 (2C), 133.2 (br s, 2C), 132.3 (br s, 2C), 130.5 (4C), 129.2 (4C), 128.9 (2C), 128.6 (q, J = 32.4 Hz, 2C), 128.2 (2C), 127.7 (4C), 124.4 (q, J = 272 Hz, 2C), 124.2 (2C), 123.7 (br s, 2C), 119.0 (2C), 21.4 (2C).

^{19}F NMR (376.5 MHz, $CDCl_3$) δ -62.30.

IR (ATR) ν_{max} 3028, 2920, 2862, 1616, 1576, 1545, 1500, 1406, 1360, 1321, 1157, 1126, 1107, 1018, 972, 849, 827, 768, 723, 667 cm^{-1}.

HRMS (ESI$^+$) m/z calcd for $C_{48}H_{31}F_6N_2$ [M + H]$^+$: 749.23859, found: 749.23891.

5e

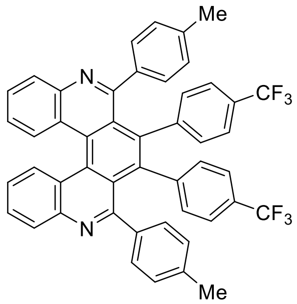

R_f (50/1 hexanes/EtOAc) = 0.02.

M.p. = 336 – 338 °C.

^{1}H NMR (400 MHz, $CDCl_3$, 25 °C) δ 8.62 (d, J = 8.6 Hz, 2H), 8.26 (dd, J = 8.3, 1.5 Hz, 2H), 7.77 – 7.73 (m, 2H), 7.44 (ddd, J = 8.4, 7.0, 1.5 Hz, 2H), 7.10 (d, J = 8.1 Hz, 2H), 6.95 (d, J = 8.1 Hz, 2H), 6.92 – 6.37 (m, 10H), 6.28 (d, J = 7.9 Hz, 2H), 2.17 (s, 6H).

^{13}C NMR (101 MHz, $CDCl_3$, 25 °C) δ 160.0 (2C), 143.6 (2C), 142.8 (2C), 139.7 (2C), 138.4 (2C), 137.6 (2C), 132.8 (2C), 132.4 (2C), 131.9 (2C), 129.8 (4C), 129.0 (4C), 128.0 (q, J = 32.4 Hz, 2C), 127.5 (4C), 126.0 (4C), 124.6 (2C), 124.0 (2C), 123.9 (q, J = 272 Hz, 2C), 123.6 (q, J = 3.8 Hz, 2C), 123.2 (q, J = 3.5 Hz, 2C), 20.8 (2C).

^{19}F NMR (376.5 MHz, $CDCl_3$) δ -63.08.

IR (ATR) ν_{max} 3057, 3028, 2925, 2868, 1616, 1406, 1321, 1161, 1109, 1065, 1016, 841, 814, 768, 723, 604 cm^{-1}.

HRMS (ESI$^+$) m/z calcd for $C_{48}H_{31}F_6N_2$ [M + H]$^+$: 749.23859, found: 749.23786.

6e. It was obtained as a mixture with **5e**.

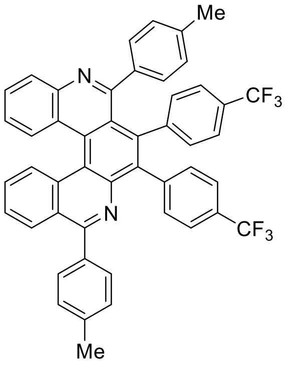

R$_f$ (50/1 hexanes/EtOAc) = 0.03.

^{1}H NMR (400 MHz, $CDCl_3$, 25 °C) δ 8.78 (d, *J* = 8.0 Hz, 1H), 8.33 (d, *J* = 6.5 Hz, 1H), 7.65 – 7.60 (m, 2H), 7.33 (d, *J* = 8.0 Hz, 2H), 7.17 (d, *J* = 5.9 Hz, 1H), 6.87 – 6.77 (m, 3H), 2.45 (s, 3H), 2.18 (s, 3H) (other signals are covered by signals from regioisomer **5e**).

^{13}C NMR (101 MHz, $CDCl_3$, 25 °C) δ 161.0, 160.6, 143.8, 143.2, 142.7, 142.0, 139.9, 139.8, 139.2, 139.1, 137.8, 135.7, 134.8, 133.9, 133.0, 132.2, 129.5, 128.9, 128.24, 128.18, 128.0, 127.9, 127.8, 127.3, 127.1, 125.4, 125.2, 124.3, 123.9, 123.4, 122.5, 21.4, 20.9 (other signals are covered by signals from regioisomer **5e**).

^{19}F NMR (376 MHz, $CDCl_3$, 25 °C) δ -62.38, -62.96.

2,5-Di-*p*-tolyldibenzo[*a*,*k*][3,8]phenanthroline (5f)

With **1f** (0.2 mmol, 93 mg) following experimental procedure **C** (Table S2, Entry 11). Column chromatography of the residue on silica gel (50/1 hexanes/EtOAc with 1% of Et_3N) provided 42 mg (46%) of compound **5f** as a yellow solid.

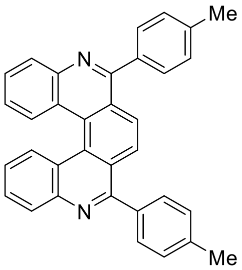

R$_f$ (50/1 hexanes/EtOAc) = 0.10.

M.p. = 126 - 128 °C.

^{1}H NMR (400 MHz, $CDCl_3$, 25 °C) δ 8.64 (d, *J* = 8.4 Hz, 2H), 8.32 (d, *J* = 8.3 Hz, 2H), 8.05 (s, 2H), 7.76 – 6.69 (m, 6H), 7.42 – 7.37 (m, 6H), 2.48 (s, 6H).

^{13}C NMR (101 MHz, $CDCl_3$, 25 °C) δ 160.0 (2C), 144.9 (2C), 139.1 (2C), 136.6 (2C), 130.9 (2C), 130.3 (4C), 129.7 (2C), 129.34 (2C), 129.25 (4C), 127.3 (2C), 126.7 (2C), 126.5 (2C), 125.3 (2C), 124.4 (2C), 21.4 (2C).

IR (ATR) ν_{max} 3055, 3026, 2918, 2852, 1597, 1556, 1439, 1360, 1180, 976, 775, 758, 725, 607 cm^{-1}.

HRMS (ESI$^+$) m/z calcd for $C_{34}H_{25}N_2$ [M + H]$^+$: 461.20122, found: 461.20111.

9,12-Dimethoxy-3,4-diphenyl-2,5-di-p-tolyldibenzo[a,k][3,8]phenanthroline (5g)

With **1g** (0.1 mmol, 68 mg) following experimental procedure **C** (Table S2, Entry 11). Column chromatography of the residue on silica gel (5/1 hexanes/EtOAc with 1% of Et_3N) provided 55 mg (82%) of the **5g** compound as a yellow solid.

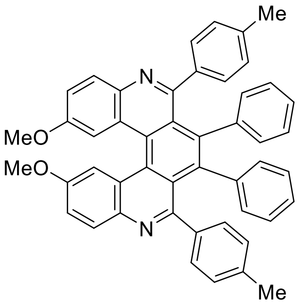

R_f (5/1 hexanes/EtOAc) = 0.26.

M.p. = 353 – 355 °C.

^{1}H NMR (400 MHz, $CDCl_3$, 25 °C) δ 8.15 (d, J = 9.0 Hz, 2H), 7.86 (d, J = 2.7 Hz, 2H), 7.35 (dd, J = 9.0, 2.7 Hz, 2H), 7.07 – 6.83 (m, 6H), 6.73 – 6.46 (m, 6H), 6.34 – 5.98 (m, 6H), 3.65 (s, 6H), 2.02 (s, 6H).

^{13}C NMR (101 MHz, $CDCl_3$, 25 °C) δ 158.7 (2C), 157.0 (2C), 143.3 (2C), 140.1 (2C), 139.1 (2C), 136.4 (2C), 135.2 (2C), 132.3 (4C), 130.6 (2C), 130.5 (4C), 127.2 (4C), 126.6 (4C), 126.3 (2C), 124.3 (2C), 120.5 (4C), 107.9 (4C), 55.5 (2C), 20.9 (2C).

IR (ATR) ν_{max} 3053, 3022, 2999, 2954, 2920, 2850, 1766, 1732, 1508, 1493, 1486, 1444, 1435, 1246, 1228, 1213, 1173, 1076, 1028, 818, 766, 694, 621, 530 cm^{-1}.

HRMS (ESI$^+$) m/z calcd for $C_{48}H_{37}N_2O_2$ [M + H]$^+$: 673.28496, found: 673.28614.

4.5 Synthesis of azonium salt

3,4-Bis(4-methoxyphenyl)-1-methyl-2,5-di-p-tolyldibenzo[a,k][3,8]phenanthrolin-1-ium iodide (5a$^+$)

In a flame-dried Schlenk flask, compound **5a** (0.1 mmol, 68 mg) and MeI (3 mmol, 0.2 mL) were dissolved in anhydrous toluene (5 mL) under an inert atmosphere. The flask was sealed and the reaction mixture was stirred at 130° C in a heat transfer block (stirring 1000 rpm) for 24 hours. After completion, the mixture was cooled down to 25 °C. Column chromatography of the residue on silica gel (20/1 CH_2Cl_2/MeOH) provided 24 mg (29%) of compound **5a**$^+$ as an orange-red solid.

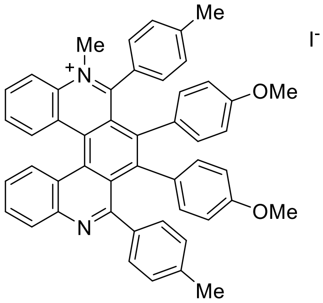

R_f (20/1 CH_2Cl_2/MeOH) = 0.29.

M.p. = 295 – 297 °C.

^{1}H NMR (400 MHz, $CDCl_3$, 25 °C) δ 8.69 (dd, J = 8.5, 1.5 Hz, 1H), 8.60 (d, J = 8.7 Hz, 2H), 8.23 (d, J = 7.0 Hz, 1H), 7.99 (ddd, J = 8.7, 7.2, 1.4 Hz, 1H), 7.76 (ddd, J = 8.3, 7.0, 1.3 Hz, 1H), 7.69 (ddd, J = 8.4, 7.2, 1.1 Hz, 1H), 7.56 (dd, J = 8.9, 2.0 Hz, 1H), 7.46 (ddd, J = 8.4, 7.0, 1.4 Hz, 1H), 7.14 (dd, J = 8.4, 2.3 Hz, 1H), 6.88 (d, J = 8.6 Hz, 2H), 6.83 – 6.77 (m, 2H), 6.70 – 6.66 (m, 2H), 6.64 – 6.21 (m, 3H), 5.97 (br s, 2H), 5.87 (dd, J = 8.7, 2.7 Hz, 1H), 5.61 (dd, J = 8.6, 2.4 Hz, 1H), 4.54 (s, 3H), 3.66 (s, 3H), 3.58 (s, 3H), 2.23 (s, 3H), 2.17 (s, 3H).

^{13}C NMR (101 MHz, $CDCl_3$, 25 °C) δ 163.0, 160.7, 158.3, 158.1, 144.0, 143.5, 142.2, 140.9, 139.6, 137.2, 134.9, 134.6, 134.2, 133.4, 133.0, 132.4, 132.0, 131.7, 130.7, 130.6, 130.5, 130.3, 129.9, 129.5, 129.4, 129.3, 129.2, 129.0, 128.8, 128.53, 128.50, 128.4, 128.1, 128.0, 127.7, 127.0, 126.9, 126.8, 123.1, 121.4, 113.2, 112.7, 112.4, 112.1, 55.3, 55.1, 45.5, 21.4, 21.0.

IR (ATR) ν_{max} 3390, 3124, 3053, 3024, 2997, 2920, 2852, 2833, 1606, 1508, 1373, 1242, 1174, 1028, 825, 754 cm^{-1}.

HRMS (ESI$^+$) m/z calcd for $C_{49}H_{39}N_2O_2$ [M + H]$^+$: 687.30061, found: 687.30118.

4.6 Synthesis of racemic diaza[7]helicenes

9,10-Bis(4-methoxyphenyl)-7,12-di-*p*-tolyldinaphtho[1,2-*a*:2',1'-*k*][4,7]phenanthroline (9), 9,10-bis(4-methoxyphenyl)-8,11-di-*p*-tolyldinaphtho[1,2-*a*:2',1'-*k*][3,8]phenanthroline (10) and 9,10-bis(4-methoxyphenyl)-8,12-di-*p*-tolyldinaphtho[1,2-*a*:2',1'-*k*][3,7]phenanthroline (11)

Diols **8** (0.1 mmol, 79 mg) and NaN_3 (0.6 mmol, 39 mg) were dissolved in HFIP (1 mL). After adding benzenesulfonic acid (1.0 mmol, 158 mg), the reaction vial was sealed and stirred at 60 °C in a heat transfer block (stirring 1000 rpm) for 2 hours. The reaction mixture was cooled down to 25 °C, quenched with a saturated aqueous solution of K_2CO_3, and extracted with EtOAc (3×10 mL). The combined organic layer was dried over Na_2SO_4, filtered, and concentrated under reduced pressure. Column chromatography of the residue on silica gel (linear gradient: 25/4/1 to 20/4/1 to 20/4/2 hexanes/CH_2Cl_2/EtOAc) provided 8 mg (10%) of compound **9**, 23 mg (30%) of compound **10** and 35 mg (45%) of compound **11** as yellow solids.

9

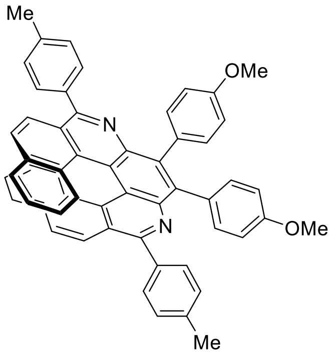

R_f (25/4/1 hexanes/CH_2Cl_2/EtOAc) = 0.35.

M.p. = 280 – 282 °C.

^{1}H NMR (400 MHz, $CDCl_3$, 25 °C) δ 8.16 (d, J = 8.9 Hz, 2H), 7.88 (d, J = 8.1 Hz, 4H), 7.63 (d, J = 8.8 Hz, 2H), 7.52 (d, J = 7.9 Hz, 2H), 7.44 (d, J = 6.6 Hz, 2H), 7.41 – 7.29 (m, 8H,), 7.09 (t, J = 7.4 Hz, 2H), 6.86 (br s, 4H), 6.59 (t, J = 7.0 Hz, 2H), 3.85 (s, 6H), 2.49 (s, 6H).

^{13}C NMR (101 MHz, $CDCl_3$, 25 °C) δ 158.5 (2C), 158.0 (2C), 142.7 (2C), 140.1 (2C), 138.6 (2C), 136.9 (2C), 134.1 (br s, 4C), 133.4 (2C), 132.9 (2C), 131.1 (2C), 130.8 (4C), 129.0 (4C), 128.2 (2C), 127.8 (2C), 127.1 (2C), 127.0 (2C), 125.6 (2C), 124.3 (2C), 123.9 (2C), 122.4 (2C), 117.5 (2C), 112.4 (4C), 55.2 (2C), 21.4 (2C).

IR (ATR) ν_{max} 3053, 3028, 3003, 2954, 2922, 2850, 2837, 1732, 1695, 1606, 1506, 1448, 1439, 1242, 1176, 1032, 829, 796, 764, 727 cm^{-1}.

HRMS (ESI$^+$) m/z calcd for $C_{56}H_{41}N_2O_2$ [M + H]$^+$: 773.31626, found: 773.31727.

10

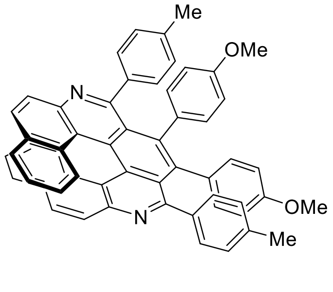

R_f (25/4/1 hexanes/CH_2Cl_2/EtOAc) = 0.10.

M.p. = 153 – 155 °C.

^{1}H NMR (400 MHz, $CDCl_3$, 25 °C) δ 8.09 (d, J = 8.8 Hz, 2H), 7.75 (d, J = 8.8 Hz, 2H), 7.43 (d, J = 6.8 Hz, 2H), 7.18 (d, J = 8.0 Hz, 2H), 7.15 (d, J = 8.0 Hz, 4H), 7.03 (t, J = 6.9 Hz, 2H), 6.91 (d, J = 7.6 Hz, 4H), 6.61 (ddd, J = 8.5, 7.0, 1.5 Hz, 2H), 6.55 (d, J = 8.0 Hz, 4H), 6.23 (d, J = 8.9 Hz, 4H), 3.62 (s, 6H), 2.27 (s, 6H).

^{13}C NMR (101 MHz, $CDCl_3$, 25 °C) δ 161.1 (2C), 157.5 (2C), 143.3 (2C), 140.5 (2C), 139.1 (2C), 136.6 (2C), 133.4 (2C), 132.2 (2C), 131.8 (2C), 130.4 (2C), 130.0 (2C), 129.4 (2C), 128.3 (2C), 128.1 (4C), 127.2 (2C), 127.1 (2C), 127.0 (2C), 125.8 (2C), 124.8 (2C), 124.5 (2C), 120.3 (2C), 112.0 (8C), 55.1 (2C), 21.2 (2C).

IR (ATR) ν_{max} 3053, 3026, 2997, 2951, 2920, 2850, 2833, 1606, 1508, 1242, 1174, 1032, 822, 795, 750 cm^{-1}.

HRMS (ESI$^+$) m/z calcd for $C_{56}H_{41}N_2O_2$ [M + H]$^+$: 773.31626, found: 773.31652.

11

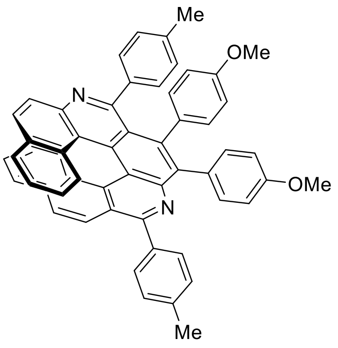

R$_f$ (25/4/1 hexanes/CH_2Cl_2/EtOAc) = 0.13.

M.p. = 344 – 346 °C.

^{1}H NMR (400 MHz, $CDCl_3$, 25 °C) δ 8.15 (d, J = 8.9 Hz, 2H), 7.85 (d, J = 8.2 Hz, 2H), 7.77 (d, J = 8.7 Hz, 1H), 7.65 (d, J = 8.6 Hz, 1H), 7.46 – 7.43 (m, 2H), 7.38 – 7.33 (m, 3H), 7.29 (d, J = 9.4 Hz, 1H), 7.09 (ddd, J = 8.1, 7.0, 1.2 Hz, 1H), 7.02 (ddd, J = 8.1, 6.9, 1.2 Hz, 1H), 6.94 – 6.67 (m, 8H), 6.64 – 6.56 (m, 2H), 6.47 – 6.23 (m, 4H), 3.80 (s, 3H), 3.67 (s, 3H), 2.48 (s, 3H), 2.24 (s, 3H).

^{13}C NMR (101 MHz, $CDCl_3$, 25 °C) δ 161.5, 160.7, 157.8 (2C), 143.9, 140.0, 139.5, 138.9, 136.7, 134.1 (2C), 134.0, 133.1, 133.0, 131.7, 130.80 (4C), 130.76, 130.1, 129.0 (4C), 128.33, 128.30, 128.0 (2C), 127.8, 127.5, 127.3, 127.0, 125.6, 125.5, 125.0, 124.9, 124.8, 124.4, 123.6, 122.6 (2C), 120.5, 115.6, 112.6 (2C), 112.3, 55.2, 55.1, 21.5, 21.1 (5 carbon signals are probably covered by other signals).

IR (ATR) ν_{max} 3076, 3049, 3022, 2995, 2949, 2918, 2850, 2831, 1606, 1508, 1242, 1173, 1034, 831, 816, 748 cm^{-1}.

HRMS (ESI$^+$) m/z calcd for $C_{56}H_{41}N_2O_2$ [M + H]$^+$: 773.31626, found: 773.31694.

4.7 Synthesis of enantioenriched diaza[7]helicenes

(*M*)-9,10-Bis(4-methoxyphenyl)-7,12-di-*p*-tolyldinaphtho[1,2-*a*:2',1'-*k*][4,7]phenanthroline (9), 9,10-bis(4-methoxyphenyl)-8,11-di-*p*-tolyldinaphtho[1,2-*a*:2',1'-*k*][3,8]phenanthroline (10) and 9,10-bis(4-methoxyphenyl)-8,12-di-*p*-tolyldinaphtho[1,2-*a*:2',1'-*k*][3,7] phenanthroline (11)

(*M*) Diols **8** (0.13 mmol, 99 mg) and NaN_3 (0.8 mmol, 52 mg) were dissolved in HFIP (1.3 mL). After adding benzenesulfonic acid (1.3 mmol, 206 mg), the reaction vial was sealed and stirred at 60 °C in a heat transfer block (stirring 1000 rpm) for 2 hours. The reaction mixture was cooled down to 25 °C, quenched with a saturated aqueous solution of K_2CO_3, and extracted with EtOAc (3×10 mL). The combined organic layer was dried over Na_2SO_4, filtered, and concentrated under reduced pressure. Column chromatography of the residue on silica gel (linear gradient: 25/4/1 to 20/4/1 to 20/4/2 hexanes/CH_2Cl_2/EtOAc) provided 8 mg (8%) of compound **9**, 27 mg (27%) of compound **10** and 37 mg (38%) of compound **11** as yellow solids.

The recorded data for (*M*)-**9** agree with those for *rac*-**9** (Chapter 4.6).

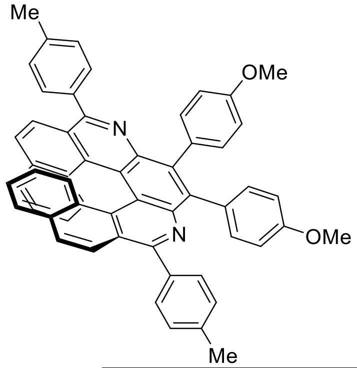

$[\alpha]_D^{20}$ -181.5 (*c* 0.33, $CHCl_3$)

(*M*)-**9** HPLC analysis: > 99:1 e.r. (column Chiralpak IA, Heptane/*i*-PrOH = 99/1, flow rate 0.25 mL/min, temperature 35° C, UV 400 nm, t_{major} = 28.3 min; t_{min} = not detected).

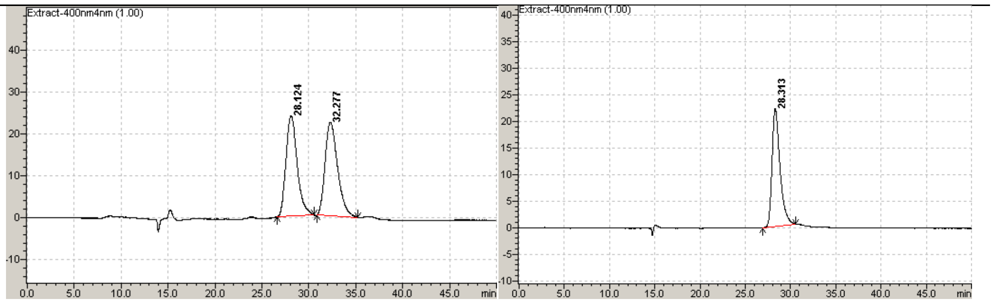

	Racemic		(*M*), > 99:1 e.r.	
N°	Retention time (min)	Relative area (%)	Retention time (min)	Relative area (%)
1	28.1	49.1	28.3	> 99
2	32.3	50.9	-	-

The recorded data for (*M*)-**10** agree with those for *rac*-**10** (Chapter 4.6).

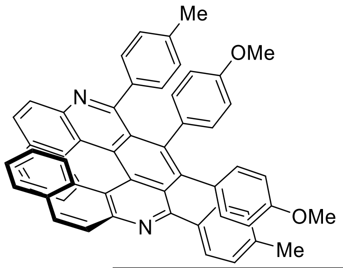

$[\alpha]_D^{20}$ -683 (*c* 0.5, $CHCl_3$)

(*M*)-**10** HPLC analysis: 99.3:0.7 e.r. (column Chiralpak IA, Heptane/*i*-PrOH = 95/5, flow rate 0.25 mL/min, temperature 25° C, UV 400 nm, t_{major} = 28.3 min; t_{min} = 30.7 min).

	Racemic		(*M*), 99.3:0.7 e.r.	

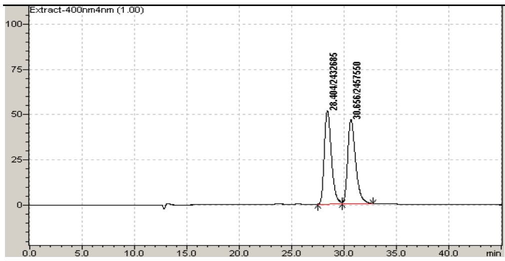

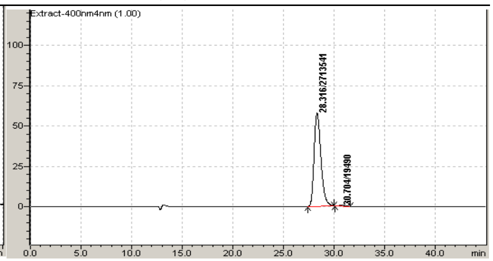

N°	Retention time (min)	Relative area (%)	Retention time (min)	Relative area (%)
1	28.4	49.7	28.3	99.3
2	30.7	50.3	30.7	0.7

The recorded data for (*M*)-**11** agree with those for *rac*-**11** (Chapter 4.6).

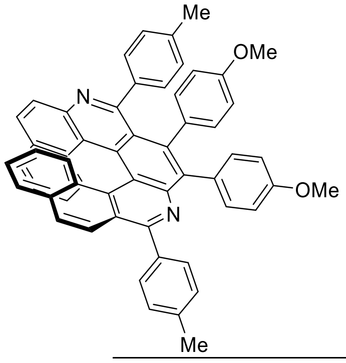

$[\alpha]_D^{20}$ -569 (*c* 0.5, $CHCl_3$)

(*M*)-**11** HPLC analysis: 1.8:98.2 e.r. (column Chiralpack IA, Heptane/*i*-PrOH = 95/5, flow rate 0.25 mL/min, temperature 25° C, UV 400 nm, t_{major} = 38.4 min; t_{min} = 26.8 min).

	Racemic		(*M*), 1.8:98.2 e.r.	

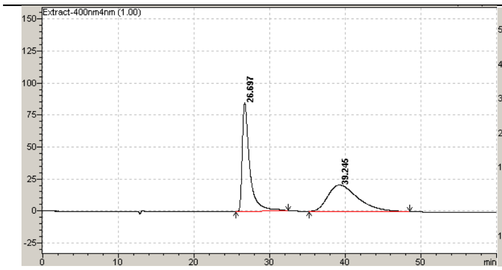

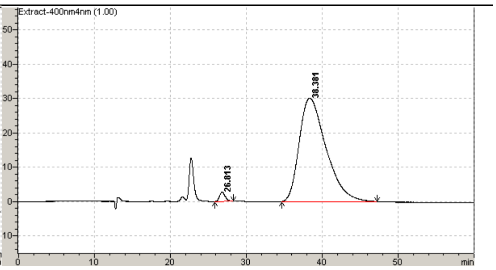

N°	Retention time (min)	Relative area (%)	Retention time (min)	Relative area (%)
1	26.7	50.5	26.8	1.8
2	39.2	49.5	38.4	98.2

(*P*)-9,10-Bis(4-methoxyphenyl)-7,12-di-*p*-tolyldinaphtho[1,2-*a*:2',1'-*k*][4,7]phenanthroline (9), 9,10-bis(4-methoxyphenyl)-8,11-di-*p*-tolyldinaphtho[1,2-*a*:2',1'-*k*][3,8]phenanthroline (10) and 9,10-bis(4-methoxyphenyl)-8,12-di-*p*-tolyldinaphtho[1,2-*a*:2',1'-*k*][3,7] phenanthroline (11)

(*P*) Diols **8** (0.3 mmol, 234 mg) and NaN_3 (1.8 mmol, 117 mg) were dissolved in HFIP (3 mL). After adding benzenesulfonic acid (3 mmol, 474 mg), the reaction vial was sealed and stirred at 60 °C in heat transfer block (stirring 1000 rpm) for 2 hours. The reaction mixture was cooled down to 25 °C, quenched with a saturated aqueous solution of K_2CO_3, and extracted with EtOAc (3×10 mL). The combined organic layer was dried over Na_2SO_4, filtered, and concentrated under reduced pressure. Column chromatography of the residue on silica gel (linear gradient: 25/4/1 to 20/4/1 to 20/4/2 hexanes/CH_2Cl_2/EtOAc) provided 15 mg (6%) of compound **9**, 54 mg (23%) of compound **10** and 101 mg (43%) of compound **11** as yellow solids.

The recorded data for (*P*)-**9** agree with those for *rac*-**9** (Chapter 4.6).

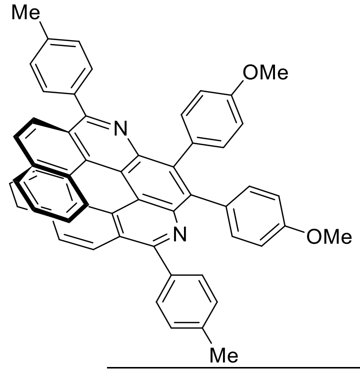

$[\alpha]_D^{20}$ +246.6 (c 0.33, $CHCl_3$)

(*P*)-**9** HPLC analysis: 4.9:95.1 e.r. (column Chiralpak IA, Heptane/*i*-PrOH = 99/1, flow rate 0.25 mL/min, temperature 35° C, UV 400 nm, t_{major} = 30.0 min; t_{min} = 27.8 min).

Racemic			(*P*), 4.9:95.1 e.r.	
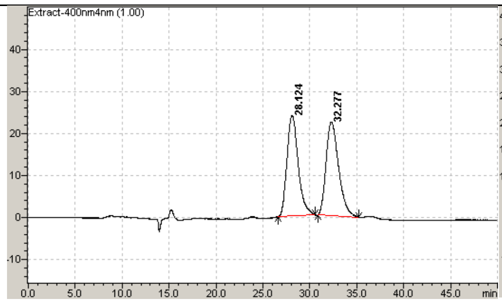			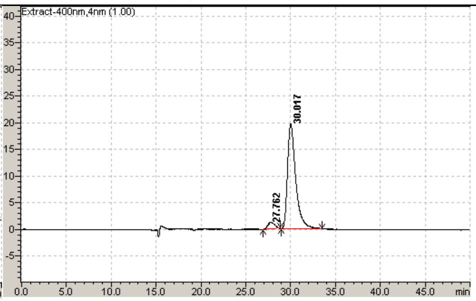	
N°	Retention time (min)	Relative area (%)	Retention time (min)	Relative area (%)
1	28.1	49.1	27.8	4.9
2	32.3	50.9	30.0	95.1

The recorded data for (*P*)-**10** agree with those for *rac*-**10** (Chapter 4.6).

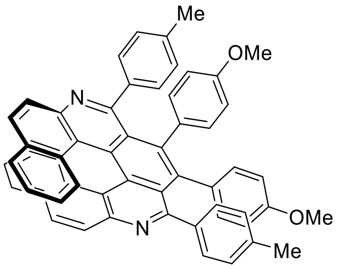

$[a]_D^{20}$ +671 (c 0.5, $CHCl_3$)

(*P*)-**10** HPLC analysis: 4.3:95.7 e.r. (column Chiralpack IA, Heptane/*i*-PrOH = 95/5, flow rate 0.25 mL/min, temperature 25° C, UV 400 nm, t_{major} = 30.7 min; t_{min} = 28.5 min).

	Racemic		(*P*), 4.3:95.7 e.r.	
	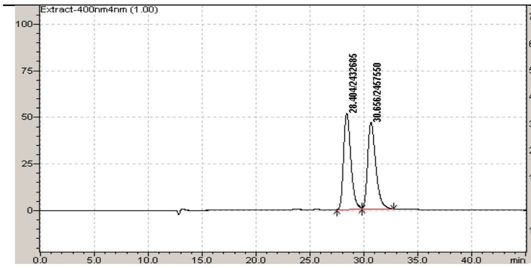		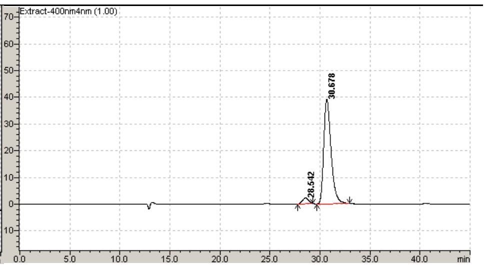	
N°	Retention time (min)	Relative area (%)	Retention time (min)	Relative area (%)
1	28.4	49.7	28.5	4.3
2	30.7	50.3	30.7	95.7

The recorded data for (*P*)-**11** agree with those for *rac*-**11** (Chapter 4.6).

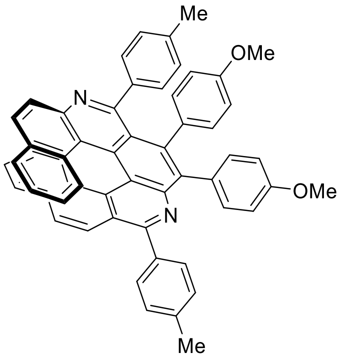

$[a]_D^{20}$ +575 (c 0.5, $CHCl_3$)

(*P*)-**11** HPLC analysis: > 99:1 e.r. (column Chiralpack IA, Heptane/*i*-PrOH = 95/5, flow rate 0.25 mL/min, temperature 25° C, UV 400 nm, t_{major} = 26.8 min; t_{min} = not detected).

	Racemic		(*P*), > 99:1 e.r.	
	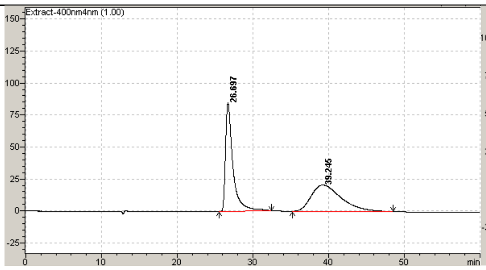		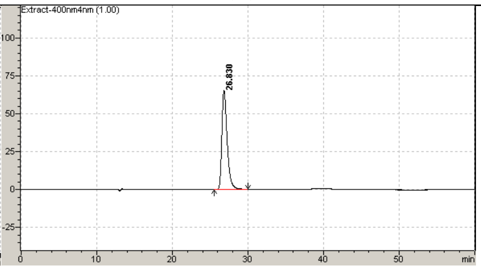	
N°	Retention time (min)	Relative area (%)	Retention time (min)	Relative area (%)
1	26.7	50.1	26.8	> 99
2	39.2	49.9	-	-

5 Photophysical properties

The UV/Vis absorption spectra were recorded using a Unicam 340 spectrometer as CH_2Cl_2 solutions (10^{-5} M). Fluorescence emission spectra were measured using FLS 980 spectrofluorometer (Edinburgh Instruments) and emission absolute quantum yields were determined using a Quantaurus-QY Plus spectrofluorometer (Hamamatsu C13534-33). Fluorescence of air-saturated solutions of samples were measured as CH_2Cl_2 solutions (10^{-6} M) in 1 cm path-length cuvettes using samples with an absorbance of 0.1 or less at the excitation wavelength.

5.1 UV/Vis absorption and emission spectra of 4a, 5a and 6a.

UV/Vis absorption and emission spectra recorded in CH_2Cl_2 are presented in Figure S2. Optical properties are summarized in Table S3.

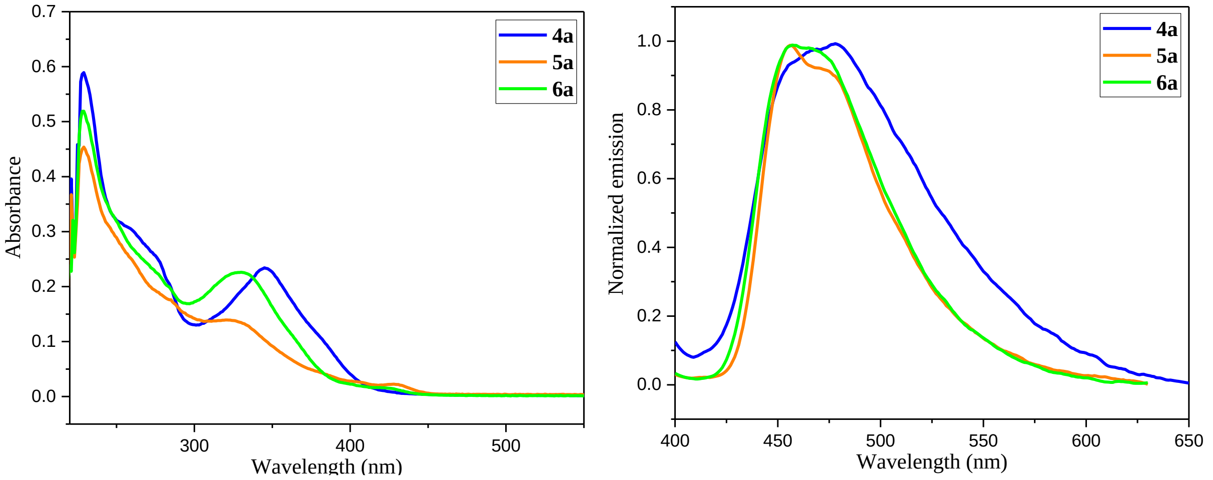

Figure S2. Absorption spectra (10^{-5} M) of **4a**, **5a** and **6a** in CH_2Cl_2 (left). Normalized and corrected emission spectra (10^{-6} M) of **4a**, **5a** and **6a** in CH_2Cl_2 using λ_{exc} (**4a**) = 324 nm and λ_{exc} (**5a**, **6a**) = 345 nm (right).

Table S3. Photophysical properties of **4a**, **5a** and **6a**, in CH_2Cl_2.

Compound	λ_{abs} (nm), ($\varepsilon/10^4$ M^{-1} cm^{-1})	λ_{em} (nm)	Stokes shift (nm)	Stokes shift (cm^{-1})	Φ_f^a
**4a**	345 (2.36)	478	133	8065.0	0.03
**5a**	323 (1.40), 429 (0.24)	456, 472	27	1380.2	0.12
**6a**	330 (2.29), 426 (0.17)	458	32	1640.1	0.12

[a] Absolute quantum yields of **4a**, **5a** and **6a** (10^{-6} M in CH_2Cl_2), λ_{exc} = 340 nm.

5.2 UV/Vis absorption and emission spectra of 4b–6c.

UV/Vis absorption and emission spectra recorded in CH_2Cl_2 are presented in Figure S3. Optical properties are summarized in Table S4.

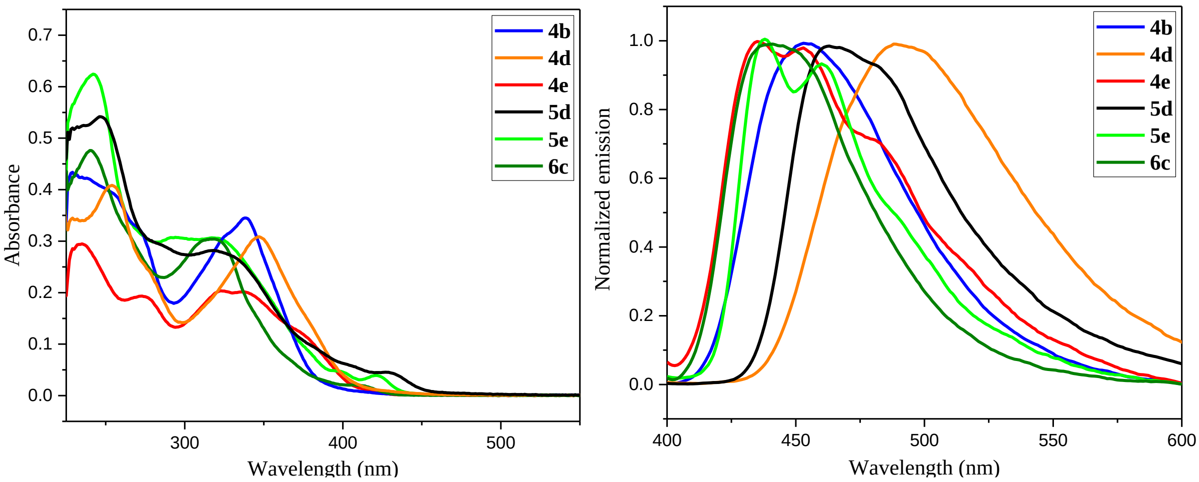

Figure S3. Absorption spectra (10^{-5} M) of **4b–6c** in CH_2Cl_2 (left). Normalized and corrected emission spectra (10^{-6} M) of **4b–6c** in CH_2Cl_2 using λ_{exc} = 320 - 350 nm (right).

Table S4. Photophysical properties of **4b–6c**, in CH_2Cl_2.

Compound	λ_{abs} (nm), ($\varepsilon/10^4$ M^{-1} cm^{-1})	λ_{em} (nm)	Stokes shift (nm)	Stokes shift (cm^{-1})	Φ_f[a]
**4b**	339 (3.46)	454	115	7472.1	0.07
**4d**	254 (4.08), 347 (3.07)	488	141	8326.6	0.08
**4e**	234 (2.94), 274 (1.92), 323 (2.04), 338 (2.02)	435, 452	97	6597.3	0.02
**5d**	246 (5.41), 317 (2.84), 430 (0.46)	463	33	1657.5	0.24
**5e**	242 (6.23), 292 (3.07), 318 (3.06), 399 (0.48), 422 (0.39)	438, 460	16	865.6	0.03
**6c**	240 (4.75), 318 (3.04), 408 (0.21)	441	33	1834.1	0.2

[a] Absolute quantum yields of **4b – 6c** (10^{-6} M in CH_2Cl_2), λ_{exc} = 320 - 350 nm.

5.3 UV/Vis absorption and emission spectra of diaza[7]helicenes.

UV/Vis absorption and emission spectra recorded in CH_2Cl_2 are presented in Figure S4. Optical properties are summarized in Table S5.

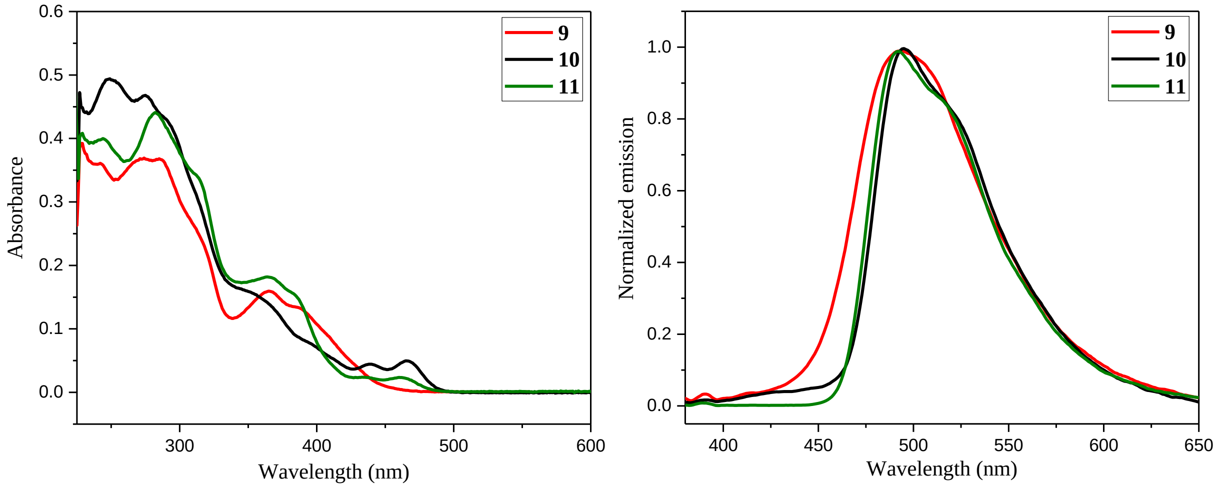

Figure S4. Absorption spectra (10^{-5} M) of **9-11** in CH_2Cl_2 (left). Normalized and corrected emission spectra (10^{-6} M) of **9-11** in CH_2Cl_2 using λ_{exc} = 350 nm (right).

Table S5. Photophysical properties of **9-11**, in CH_2Cl_2.

Compound	λ_{abs} (nm), ($\varepsilon/10^4$ M^{-1} cm^{-1})	λ_{em} (nm)	Stokes shift (nm)	Stokes shift (cm^{-1})	Φ_f[a]
**9**	272 (3.68), 285 (3.68), 366 (1.59)	492	126	6997.2	0.05
**10**	249 (4.93), 275 (4.69), 439 (0.44), 466 (0.50)	495	29	1257.2	0.14
**11**	282 (4.41), 364 (1.81), 461 (0.23)	492	32	1366.8	0.16

[a] Absolute quantum yields of **9-11** (10^{-6} M in CH_2Cl_2), λ_{exc} = 340 nm.

5.4 UV/Vis absorption and emission spectra of azonium salt.

UV/Vis absorption and emission spectra recorded in CH_2Cl_2 are presented in Figure S5. Optical properties are summarized in Table S6.

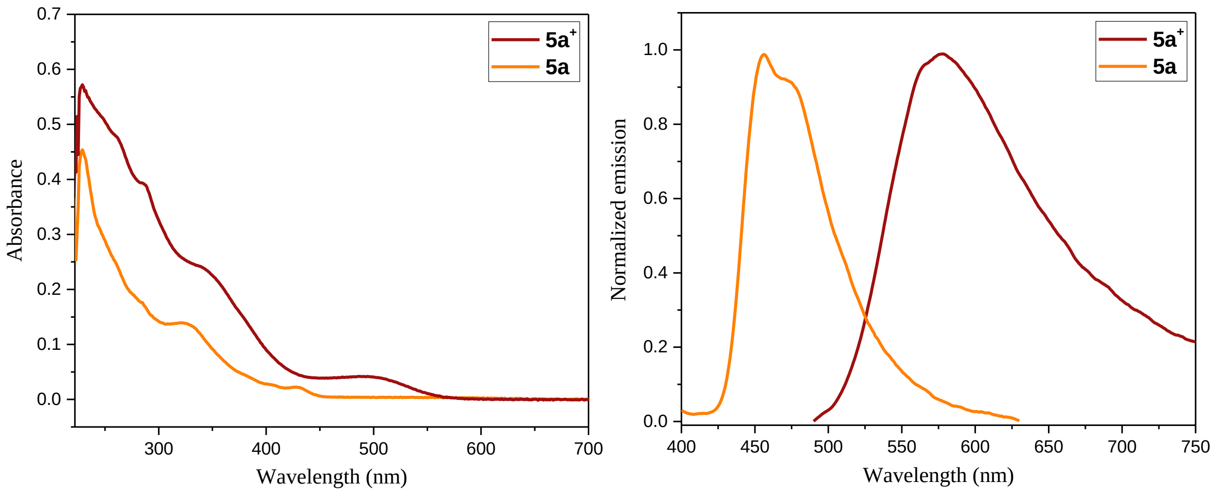

Figure S5. Absorption spectra (10^{-5} M) of **5a**$^+$ and **5a** in CH_2Cl_2 (left). Normalized and corrected emission spectra (10^{-6} M) of **5a**$^+$ and **5a** in CH_2Cl_2 using λ_{exc} = 480 nm (**5a**$^+$) and λ_{exc} = 345 nm (**5a**) (right).

Table S6. Photophysical properties of **5a**$^+$ and **5a**, in CH_2Cl_2.

Compound	λ_{abs} (nm), ($\varepsilon/10^4$ M^{-1} cm^{-1})	λ_{em} (nm)	Stokes shift (nm)	Stokes shift (cm^{-1})	Φ_f^a
**5a**$^+$	344 (2.35), 500 (0.43)	578	78	2699.0	0.02
**5a**	323 (1.40), 429 (0.24)	456, 472	27	1380.2	0.12

^{a} Absolute quantum yields of **5a**$^+$ and **5a** (10^{-6} M in CH_2Cl_2), λ_{exc} = 480 nm (**5a**$^+$) and λ_{exc} = 340 nm (**5a**).

6 Mechanistic studies

To confirm the formation of carbocations **1a**$^+$ and **1a**$^{++}$**,** absorption spectra of **1a**, **1a** with benzenesulfonic acid, and **1a** with triflic acid in HFIP were measured (Scheme S5).

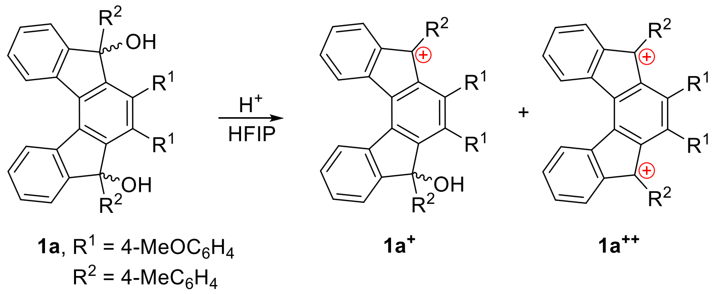

Scheme S5. Formation of carbocations under acidic conditions.

The spectra for **1a** (yellow line), **1a** with benzenesulfonic acid (black line), and **1a** with triflic acid (purple line) are shown in Figure S6. Spectra of **1a** and **1a** with benzenesulfonic acid matched, but **1a** with triflic acid has two new broad signals with absorption maxima at 515 and 585 nm. The calculated values (ZINDO) for **1a**$^+$ and **1a**$^{++}$ were 460 and 600 nm, and 450 and 600 nm (Figure S7). To promote the formation of the elusive dication, the spectrum of **1a** with 30 equivalents of triflic acid was measured. As a result, a sharper absorption spectrum was obtained (Figure S6, right) with new absorption peaks at 520 and 590 nm, and the spectra resemble the calculated spectra of both **1a**$^+$ and **1a**$^{++}$ (Figure S7).

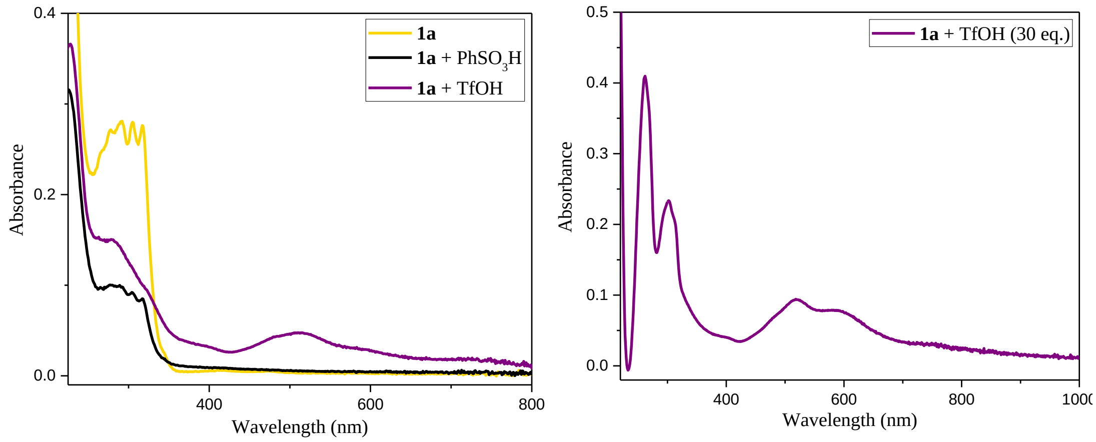

Figure S6. Absorption spectra (10^{-5} M) of **1a** (yellow) in HFIP with benzenesulfonic acid (black) and triflic acid (purple) (left). Absorption spectra (10^{-5} M) of **1a** in HFIP with 30 equivalents of triflic acid (right).

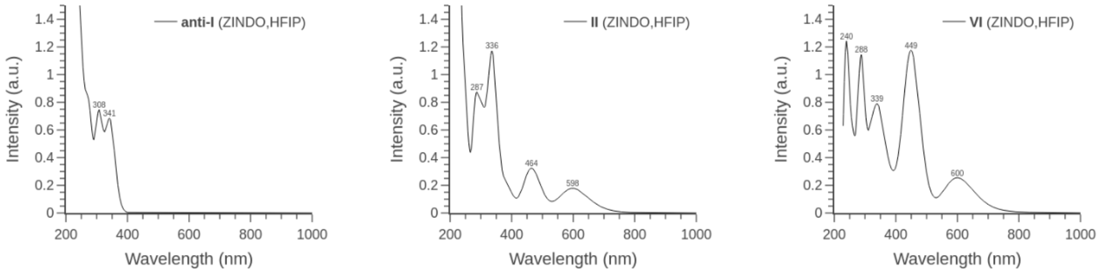

Figure S7. Calculated absorption spectra of **anti-I** (left), **II** (middle), and **VI** (right) in HFIP using semiemperical method ZINDO.

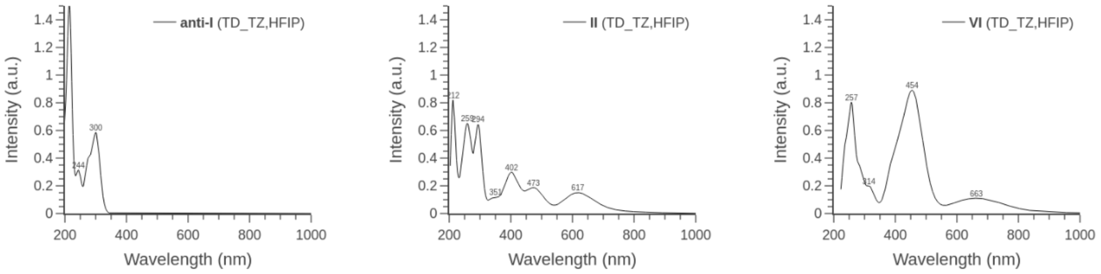

Figure S8. Calculated absorption spectra of **anti-I** (left), **II** (middle), and **VI** (right) in HFIP using TD-DFT (CAM-B3LYP functional with triple-zeta basis set).

To compare the solvent effect and the different results obtained with *i*-PrOH (Chapter 4.1.), measured absorption spectra of **1a** and **1a** with triflic acid in *i*-PrOH were measured. The spectrum for **1a** with triflic acid (the red line, Figure S9) matched that of **1a** alone (the black line, Figure S9). That indicates that the carbocation formation is much slower in *i*-PrOH than in HFIP.

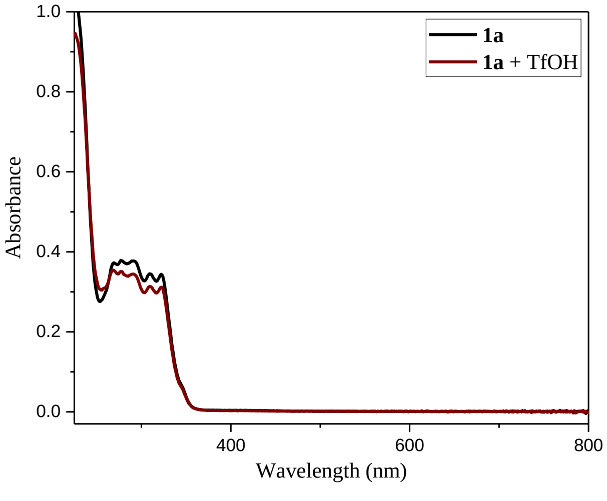

Figure S9. Absorption spectra (10^{-5} M) of **1a** and **1a** with triflic acid in *i*-PrOH.

To further confirm the carbocation formation, the ^{13}C of **1a** with triflic acid was measured. The spectrum of **1a** with triflic acid was measured in HFIP for 16 hours using a capillary insert with deuterated water. The signal for quaternary carbon belonging to the tertiary alcohol of **1a** (85 ppm) completely disappeared, and three additional signals (203, 199, and 194 ppm), corresponding to the carbocation region around 200 ppm, were observed. However, in the absence of nucleophiles, the carbocation species undergoes polymerization.

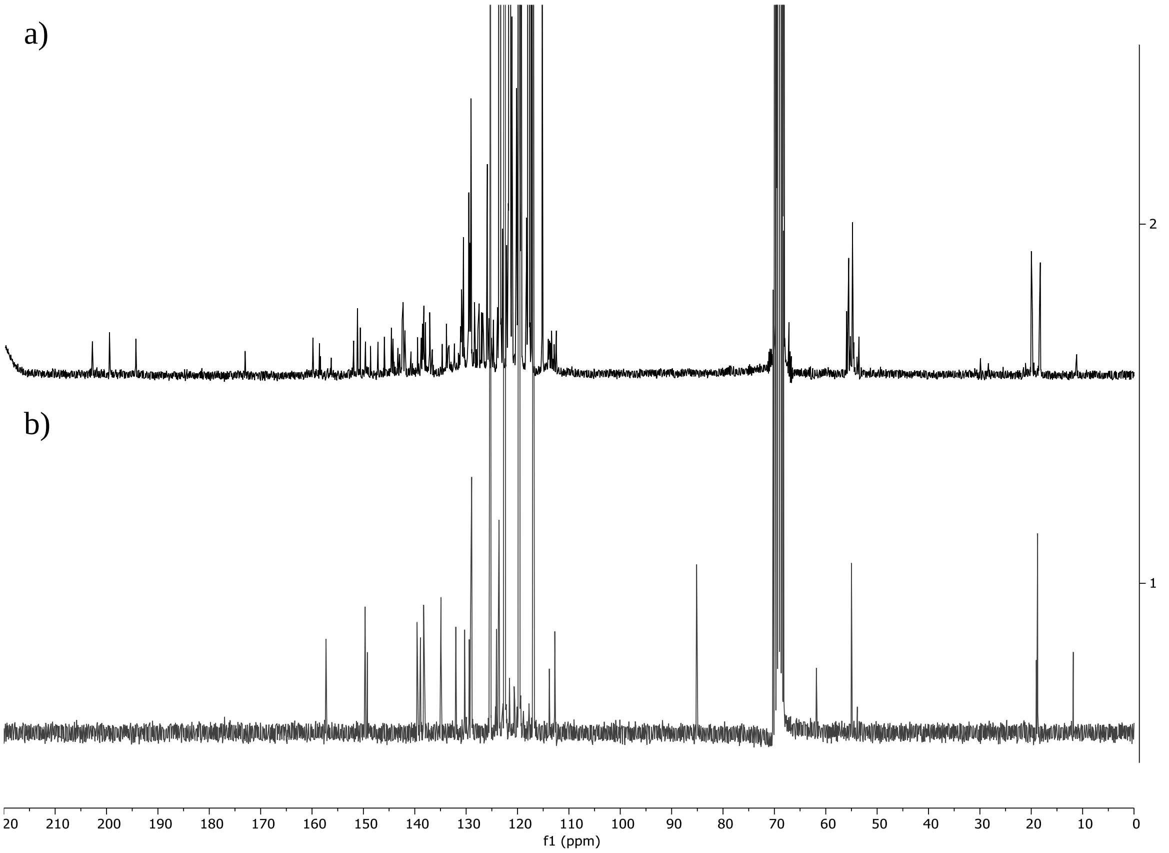

Figure S10. a) ^{13}C NMR spectrum of **1a** with triflic acid using capillary insert (D_2O) in HFIP (600 MHz); b) ^{13}C NMR spectrum of **1a** in HFIP.

7 X-Ray diffraction data

The diffraction data of single crystals **2a** (md_010_1), **4a** (md-17), **5a** (md_0453), **5d** (hs_48f4_sq), **6c** (md_178_2), **7a-anti** (md_411_3), **7a-syn** (md_415), **9** (md_2292), **10** (md_311p3), and **11** (md_311_p2) samples were obtained on Bruker D8 VENTURE Kappa Duo PHOTONIII by IμS micro-focus sealed tube either with CuKα (λ= 1.54178Å) radiation or MoKα (λ= 0.71073Å) at low temperature of the crystal preserved by Cryostream Cooler 800. The structures were solved by direct methods (XT[4]) and refined by full matrix least squares based on F^2 (SHELXL2019[5]). The hydrogen atoms on carbon were fixed into idealized positions (riding model) and assigned temperature factors either $H_{iso}(H) = 1.2\ U_{eq}$(pivot atom) or 1.5 U_{eq}(pivot atom) for methyl moiety.

The most of crystals of studied samples exhibit great propensity of disorders either main molecules or solvent.

Detailed description for individual samples:

a) Solvating acetone in crystal of **4a** (md-17) is disordered over two position.

b) The butyl moiety of **6c** (md_178_2) is disordered, with equal occupancy of two positions of disordered atoms.

c) Both symmetrically independent molecules in unit cell of **5a** (md_0453) are disordered, one in tolyl moiety and the second in methoxyphenyl moiety

d) To improve precision of main molecule of **5d** (hs_48f4_sq) the contribution of the heavily disordered solvent to diffraction pattern was removed using PLATON SQUEEZE procedure.[6]

e) Solvating dichloromethyl molecules in **9** (md_2292), **11** (md_311_p2), and **7a-anti** (md_411_3) disordered over several positions, described with partially occupancy of coordinates of chlorine.

f) Similarly as in **5a** (md_0453) crystal also in **2a** (md_010_1) the methoxyphenyl moiety is disordered. The compound crystalized in non-centrosymmetric space group, however due to lack of strong anomalous scattering together with low precision of structure determination due to disorder, the determination of absolute configuration was not successful.

g) Almost half of molecule of **7a-syn** (md_415) is disordered, hampering severely the precision of resulting structural parameters. However the orientation of azide moieties could be clearly discern.

X-ray crystallographic data have been deposited with the Cambridge Crystallographic Data Centre (CCDC), viz, Table ### and can be obtained free of charge from the Centre via its website (www.ccdc.cam.ac.uk/structures/).

---

4 Sheldrick, G.M. *Acta Cryst.* **2015**, *A71*, 3–8.
5 Sheldrick, G.M. *Acta Cryst.* **2015**, *C71*, 3–8.
6 Spek, A. L. *Acta Cryst.* **2015**, *C71*, 9–18.

Table S7. Crystal data, data collection, and refinement parameters for *m*,*n*-diaza[5] helicenes **4a**, **5a**, and **6c**.

**Compound**	**4a** (md-17)	**5a** (md_0453)	**6c** (md_178_2)
CCDC	2448755	2448757	2448756
Formula	$2(C_{48}H_{36}N_2O_2)\cdot C_3H_6O$	$C_{48}H_{36}N_2O_2\cdot 0.074$ (C_2H_3N)	$C_{42}H_{40}N_2O_2$
M.w.	1403.65	675.82	604.76
Crystal system	Triclinic	Triclinic	Tetragonal
Space group	*P*-1 (No.2)	*P*-1 (No.2)	$P\bar{4}2_1c$ (No.114)
a [Å]	10.6941 (5)	14.4378 (3)	31.0487 (6)
b [Å]	12.1658 (6)	14.5924 (3)	
c [Å]	14.5099 (7)	18.0585 (4)	7.1190 (2)
α [°]	91.449 (2)	98.844 (1)	
β [°]	97.478 (2)	100.183 (1)	
γ [°]	102.643 (2)	98.088 (1)	
Z	1	4	8
$V[Å^3]$	1823.51 (15)	3644.61 (13)	6862.9 (3)
Temperature	120	120	120
D_x [g cm^{-3}]	1.278	1.232	1.171
Wavelength, Å	1.54178	1.54178	1.54178
Crystal size [mm]	0.19 × 0.19 × 0.11	0.75 × 0.12 × 0.08	0.48 × 0.08 × 0.06
Crystal color, shape	Prism, colourless	Prism, gold	Bar, yellow
μ [mm^{-1}]	0.61	0.58	0.55
T_{min},T_{max}	0.892, 0.937	0.668, 0.953	0.778, 0.967
Measured reflections	36180	76966	43513
Independent diffractions ($R_{int}{}^a$)	7092, (0.029)	14274, (0.032)	6079, (0.061)
Observed diffract. [I>2σ(I)]	6653	12688	5723
No. of parameters	500	1022	437
R^b	0.038	0.040	0.040
$wR(F^2)$ for all data	0.099	0.106	0.109
GOF^c	1.04	1.02	1.04
Residual electron density [e/Å^3]	0.29, -0.39	0.28, −0.25	0.29, -0.19
Absolute structure parameter			

$^aR_{int} = \Sigma|F_o^2 - F_{o,mean}^2|/\Sigma F_o^2$;

$^bR(F) = \Sigma||F_o| - |F_c||/\Sigma|F_o|$; $wR(F^2) = [\Sigma(w(F_o^2 - F_c^2)^2)/(\Sigma w(F_o^2)^2)]^{½}$;

$^c\mathrm{GOF} = [\Sigma(w(F_o^2 - F_c^2)^2)/(N_{diffrs} - N_{params})]^{½}$

Table S8. Crystal data, data collection, and refinement parameters for *m*,*n*-diaza[5] helicene **5d**.

Compound	**5d** (hs_48f4_sq)
CCDC	2448758
Formula	$C_{48}H_{30}F_6N_2O_2$
M.w.	780.74
Crystal system	Monoclinic
Space group	*C*2/*c* (No. 15)
a [Å]	44.2132 (8)
b [Å]	7.5129 (1)
c [Å]	30.5429 (6)
α [°]	
β [°]	129.157 (1)
γ [°]	
Z	8
V[Å^3]	7866.9 (3)
Temperature [K]	120
D_x [g cm^{-3}]	1.318
Wavelength [Å]	1.54178
Crystal size [mm]	0.51 × 0.08 × 0.05
Crystal color, shape	Bar, yellow
μ [mm^{-1}]	0.84
T_{min},T_{max}	0.675, 0.958
Measured reflections	36845
Independent diffractions(R_{int}^a)	7742, (0.037)
Observed diffract. [I>2σ(I)]	6573
No. of parameters	525
R^b	0.057
$wR(F^2)$ for all data	0.156
GOF^c	1.04
Residual electron density [e/Å^3]	0.81, −0.74
Absolute structure parameter	

$^aR_{int} = \Sigma|F_o^2 - F_{o,mean}^2|/\Sigma F_o^2$;
$^bR(F) = \Sigma||F_o| - |F_c||/\Sigma|F_o|$; $wR(F^2) = [\Sigma(w(F_o^2 - F_c^2)^2)/(\Sigma w(F_o^2)^2)]^{½}$;
$^cGOF = [\Sigma(w(F_o^2 - F_c^2)^2)/(N_{diffrs} - N_{params})]^{½}$

Table S9. Crystal data, data collection, and refinement parameters for *m,n*-diaza[7] helicenes **9**, **10**, **11**.

Compound	**9** (md_2292)	**10** (md_311p3)	**11** (md_311_p2)
CCDC	2448759	2448761	2448760
Formula	$C_{56}H_{40}N_2O_2{\cdot}CH_2Cl_2$	$C_{56}H_{40}N_2O_2$	$C_{56}H_{40}N_2O_2{\cdot}2(CHCl_3)$
M.w.	857.82	772.90	1011.63
Crystal system	Triclinic	Monoclinic	Triclinic
Space group	*P*-1 (No.2)	*C*2/*c* (No. 15)	*P*-1 (No.2)
a [Å]	11.2697 (6)	13.2260 (4)	15.3519 (4)
b [Å]	13.8021 (7)	27.7359 (8)	17.0755 (5)
c [Å]	16.1418 (8)	10.9097 (3)	21.7630 (6)
α [°]	65.993 (2)		104.827 (1)
β [°]	71.824 (2)	95.905 (1)	106.963 (1)
γ [°]	88.535 (2)		104.401 (1)
Z	2	4	4
$V[Å^3]$	2164.2 (2)	3980.8 (2)	4942.0 (2)
Temperature [K]	120	120	120
D_x [g cm^{-3}]	1.316	1.290	1.360
Wavelength [Å]	1.54178	0.71073	1.54178
Crystal size [mm]	0.42 × 0.14 × 0.09	0.40 × 0.15 × 0.10	0.28 × 0.19 × 0.10
Crystal color, shape	Bar, yellow	Bar, orange-yellow	Prism, yellow
μ [mm^{-1}]	1.72	0.08	3.53
T_{min},T_{max}	0.534, 0.861	0.97, 0.992	0.436, 0.73
Measured reflections	68207	29309	87250
Independent diffractions($R_{int}{}^a$)	9103, (0.027)	4957, (0.036)	18018, (0.027)
Observed diffract. [I>2σ(I)]	8678	4319	17175
No. of parameters	600	273	1246
R^b	0.048	0.046	0.046
$wR(F^2)$ for all data	0.141	0.116	0.117
GOF^c	1.06	1.03	1.01
Residual electron density [e/Å^3]	0.78, -0.81	0.36, −0.25	1.15, -1.07
Absolute structure parameter			

$^aR_{int} = \Sigma|F_o^2 - F_{o,mean}^2|/\Sigma F_o^2$;

$^bR(F) = \Sigma||F_o| - |F_c||/\Sigma|F_o|$; $wR(F^2) = [\Sigma(w(F_o^2 - F_c^2)^2)/(\Sigma w(F_o^2)^2)]^{½}$;

$^c\text{GOF} = [\Sigma(w(F_o^2 - F_c^2)^2)/(N_{diffrs} - N_{params})]^{½}$

Table S10. Crystal data, data collection, and refinement parameters for azide and diazides **2a**, **7a-anti**, and **7a-syn**.

Compound	**2a** (md_010_1)	**7a-anti** (md_411_3)	**7a-syn** (md_415)
CCDC	2448762	2448764	2448763
Formula	$C_{48}H_{36}N_4O_2$	$C_{48}H_{36}N_6O_2{\cdot}CHCl_3$	$C_{48}H_{36}N_6O_2$
M.w.	700.81	848.19	728.83
Crystal system	Monoclinic	Orthorhombic	Monoclinic
Space group	*C*2 (No. 5)	*Pbca* (No. 61)	$P2_1/n$ (No. 14)
a [Å]	39.1376 (16)	20.6839 (7)	8.7607 (3)
b [Å]	6.6970 (3)	18.8234 (6)	15.8193 (6)
c [Å]	14.2576 (6)	21.6349 (6)	27.3472 (11)
α [°]			
β [°]	104.592 (3)		96.335 (2)
γ [°]			
Z	4	8	4
V[$Å^3$]	3616.4 (3)	8423.4 (5)	3766.9 (2)
Tempereture	120	120	120
D_x [g cm^{-3}]	1.287	1.338	1.285
Wavelength	1.54178	1.54178	1.54178
Crystal size [mm]	0.30 × 0.10 × 0.05	0.40 × 0.28 × 0.10	0.33 × 0.32 × 0.21
Crystal color, shape	Bar, yellow	Plate, colourless	Prism, yellow
μ [mm^{-1}]	0.62	2.36	0.64
T_{min},T_{max}	0.836, 0.971	0.452, 0.792	0.817, 0.881
Measured reflections	16070	58374	34275
Independent diffractions (R_{int}^a)	6097, (0.072)	8902, (0.038)	7637, (0.039)
Observed diffract. [I>2σ(I)]	4668	7921	6651
No. of parameters	541	567	591
R^b	0.067	0.079	0.066
$wR(F^2)$ for all data	0.160	0.238	0.179
GOF^c	1.10	1.09	1.03
Residual electron density [e/$Å^3$]	0.24, -0.24	1.03, -0.76	0.31, −0.33
Absolute structure parameter	-0.4 (4)		

$^aR_{int} = \Sigma|F_o^2 - F_{o,mean}^2|/\Sigma F_o^2$;
$^bR(F) = \Sigma||F_o| - |F_c||/\Sigma|F_o|$; $wR(F^2) = [\Sigma(w(F_o^2 - F_c^2)^2)/(\Sigma w(F_o^2)^2)]^{½}$;
^{c}GOF $= [\Sigma(w(F_o^2 - F_c^2)^2)/(N_{diffrs} - N_{params})]^{½}$

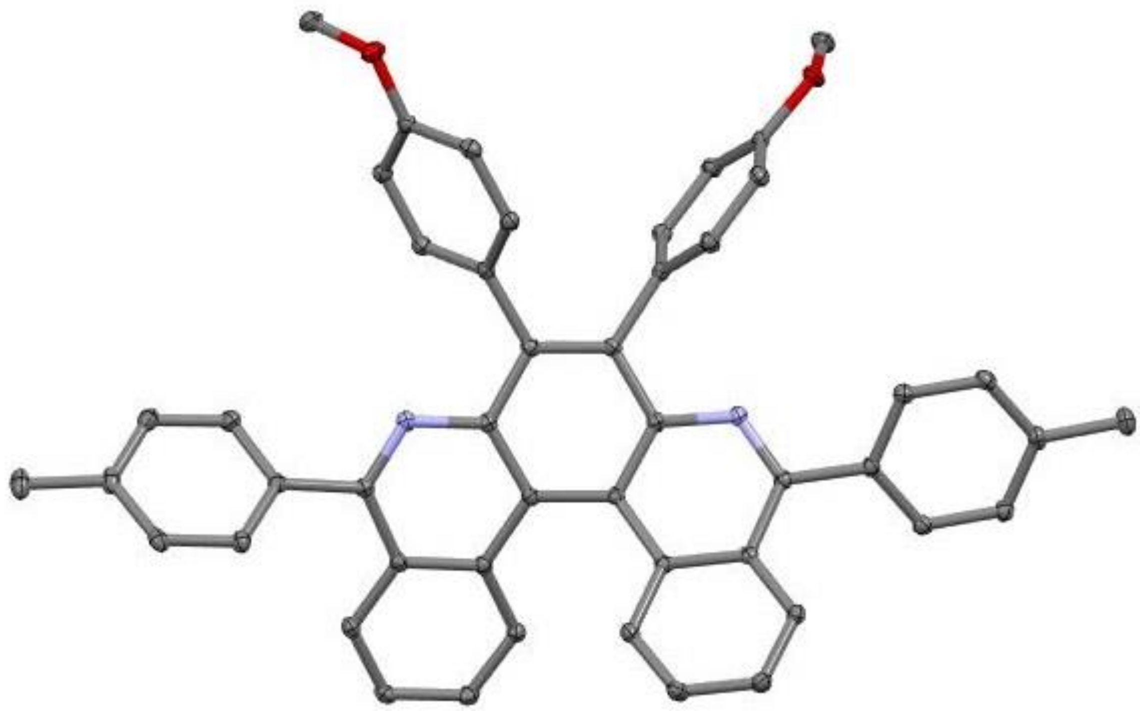

Figure S11. ORTEP drawing of **4a**. Elipsoids are drawn with 30% probability.

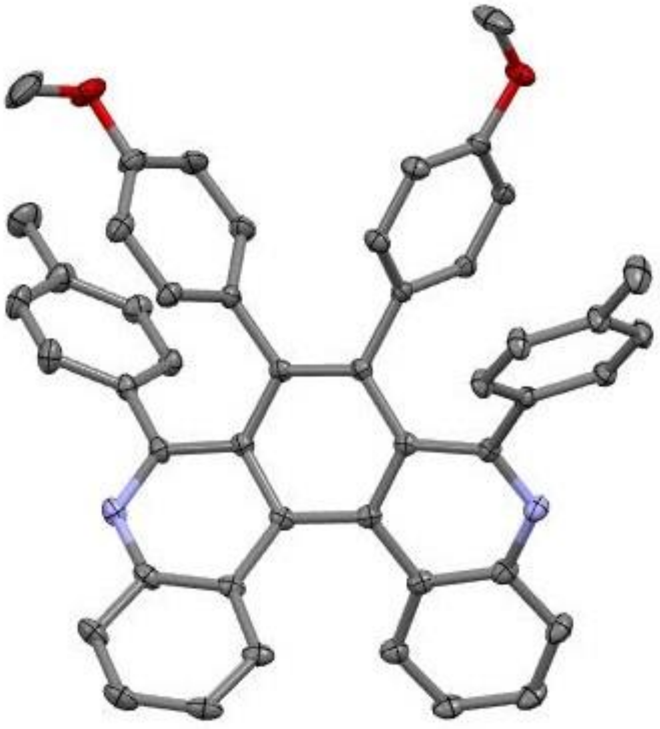

Figure S12. ORTEP drawing of **5a**. Elipsoids are drawn with 30% probability.

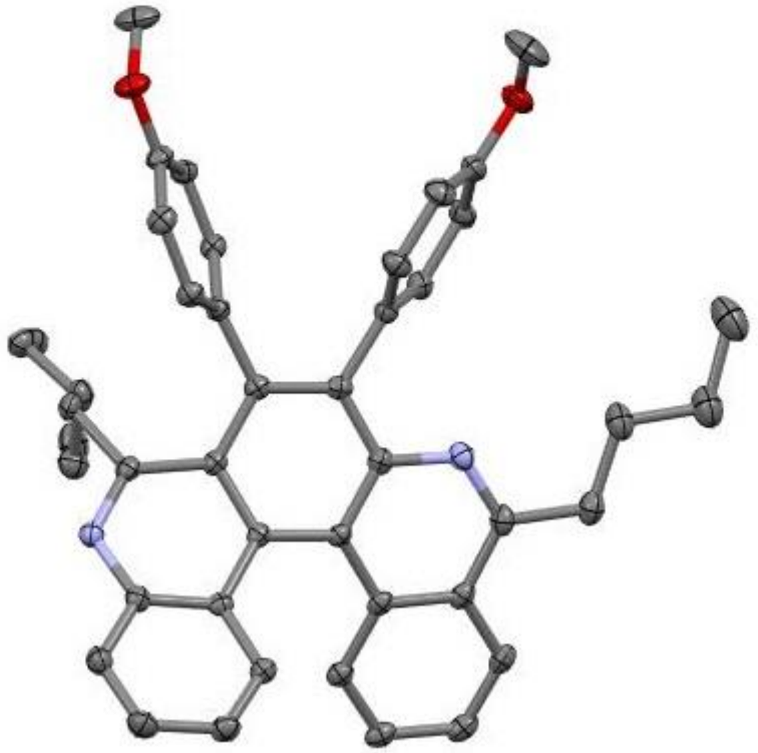

Figure S13. ORTEP drawing of **6c**. Elipsoids are drawn with 30% probability.

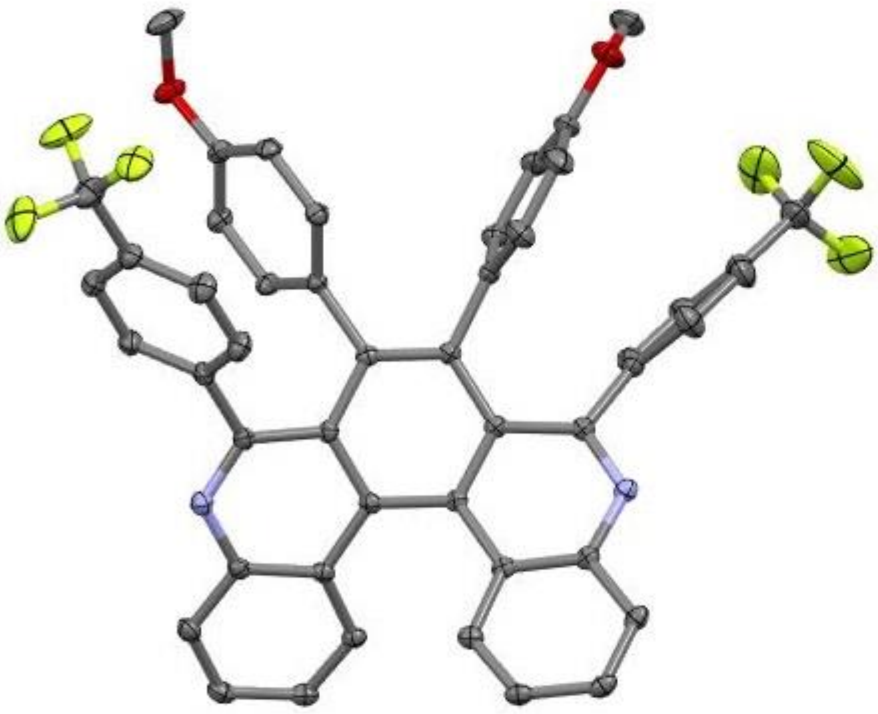

Figure S14. ORTEP drawing of **5d**. Elipsoids are drawn with 30% probability.

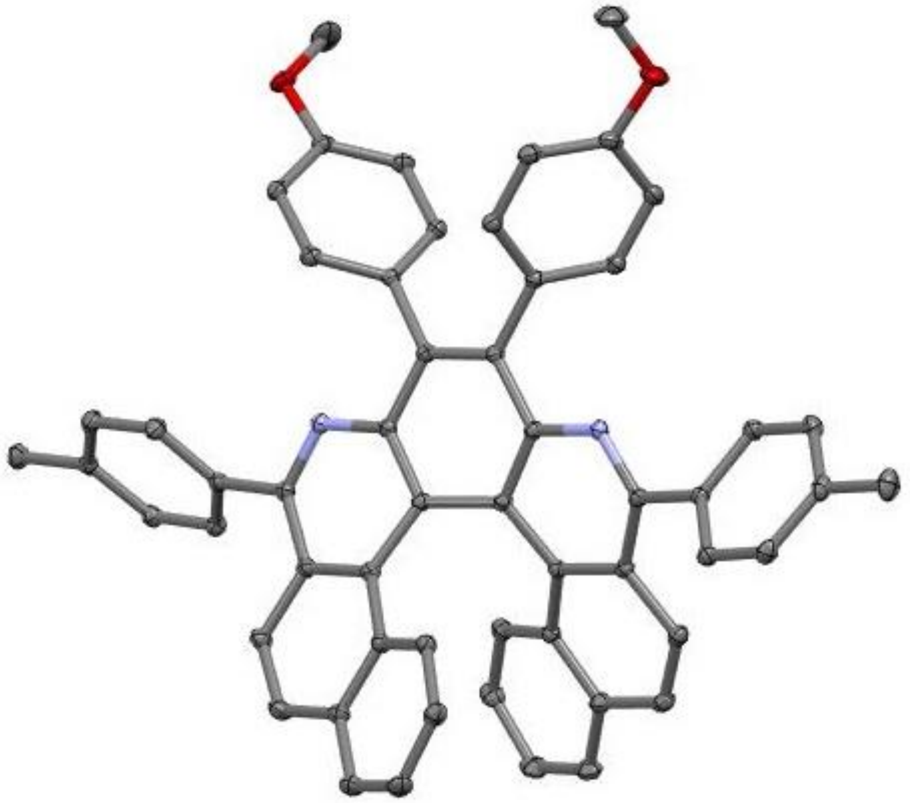

Figure S15. ORTEP drawing of **9**. Elipsoids are drawn with 30% probability.

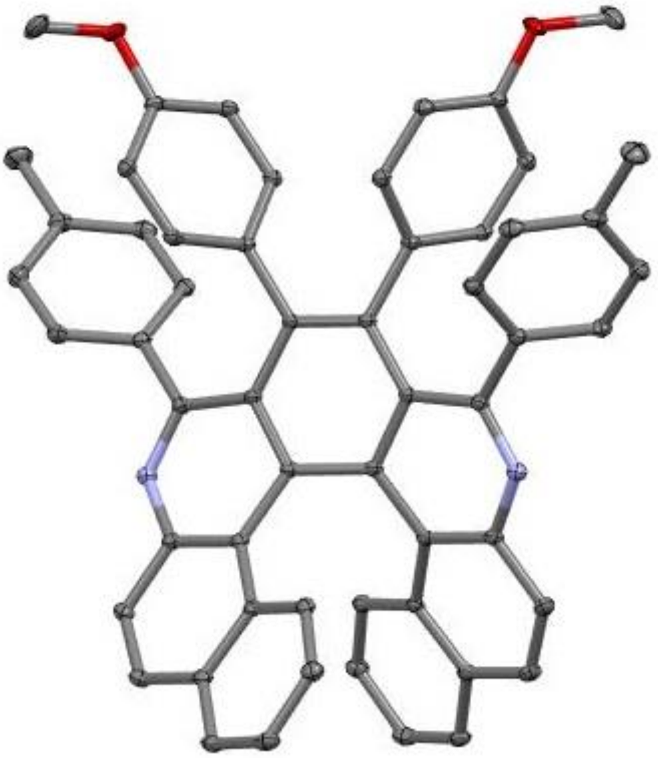

Figure S16. ORTEP drawing of **10**. Elipsoids are drawn with 30% probability.

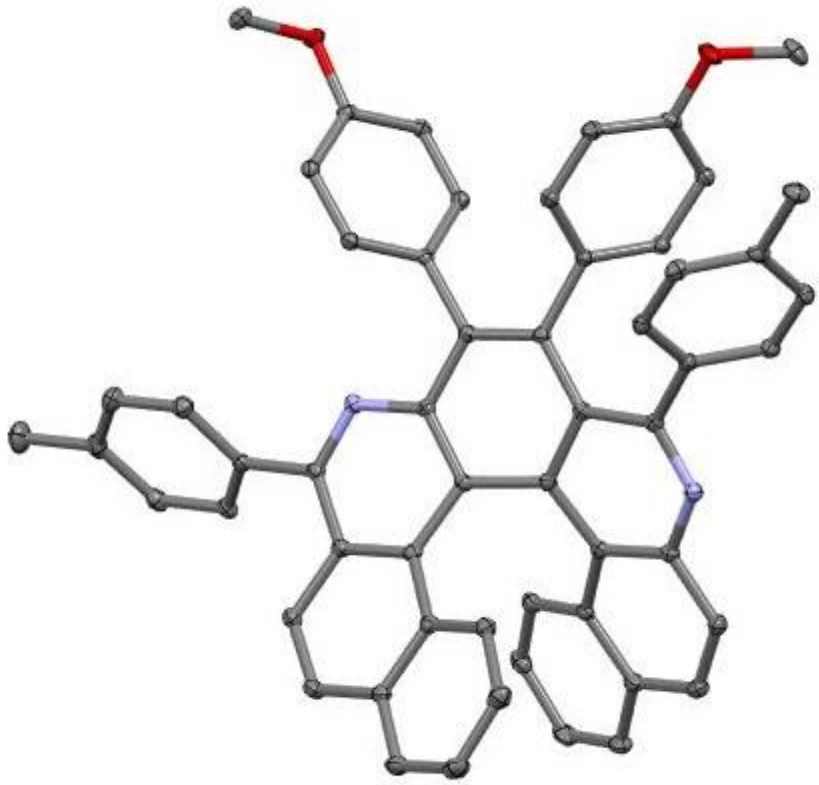

Figure S17. ORTEP drawing of **11**. Elipsoids are drawn with 30% probability.

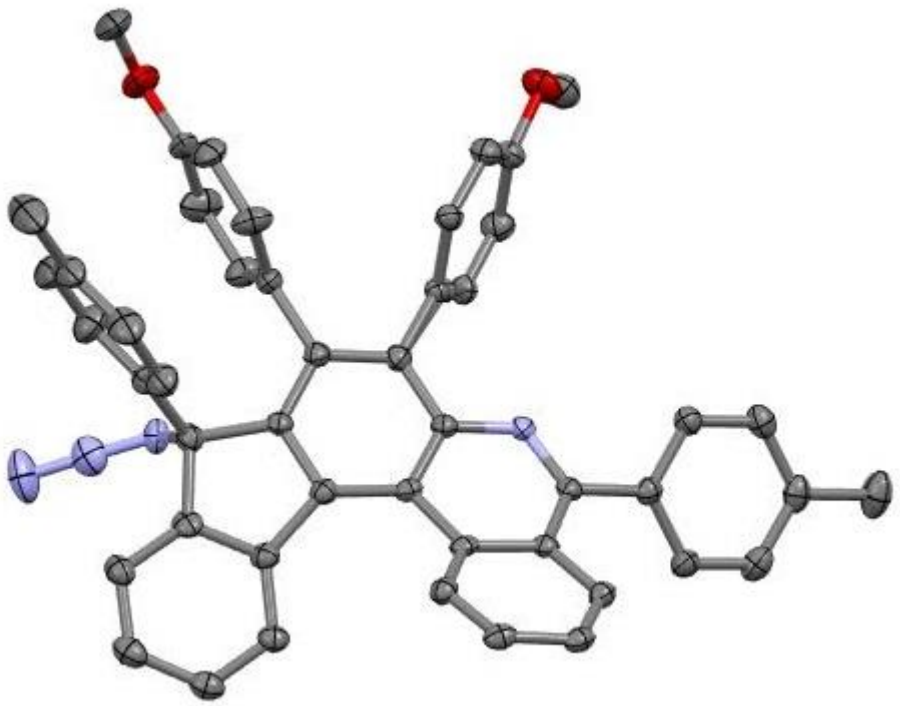

Figure S18. ORTEP drawing of **2a**. Elipsoids are drawn with 30% probability.

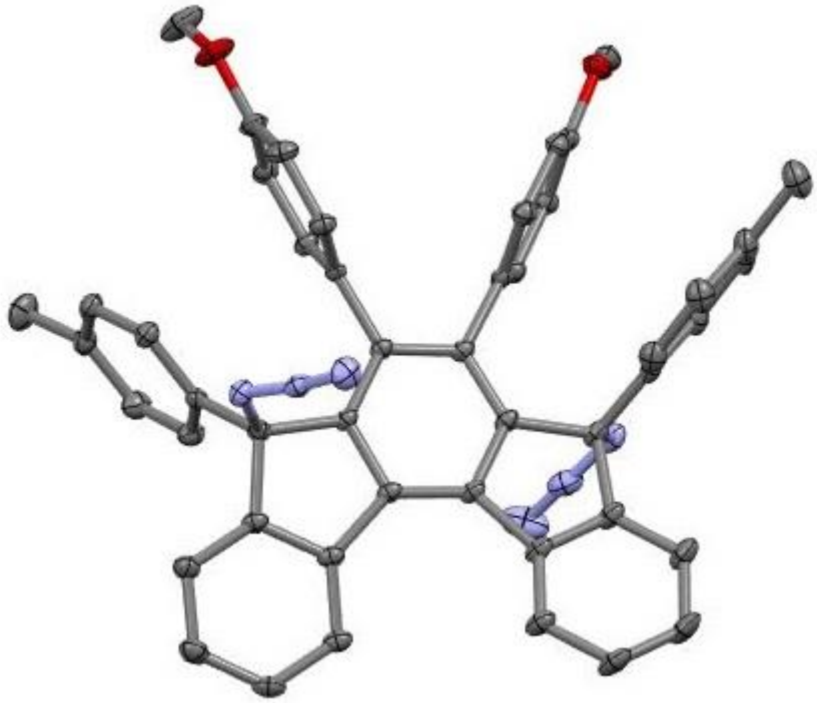

Figure S19. ORTEP drawing of **7a-anti**. Elipsoids are drawn with 30% probability.

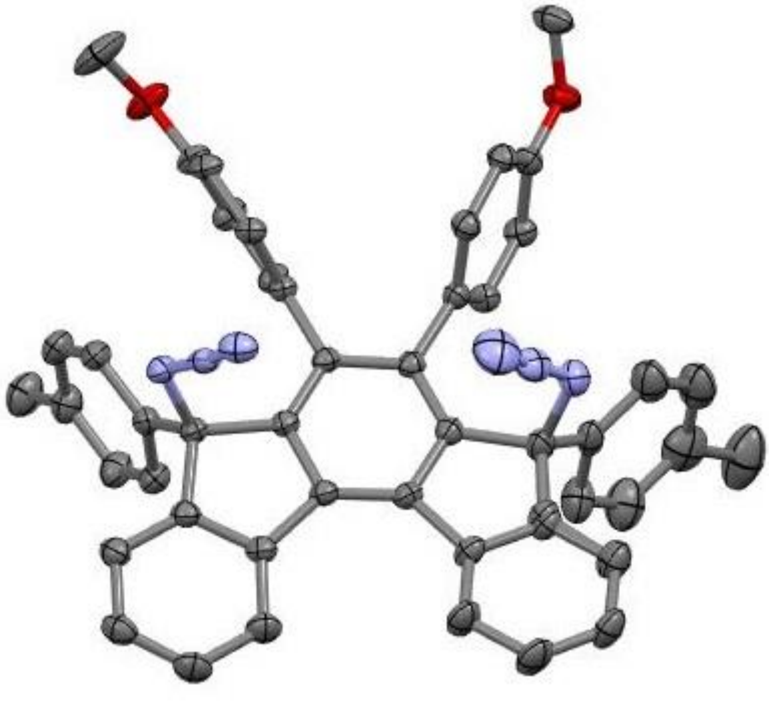

Figure S20. ORTEP drawing of **7a-syn**. Elipsoids are drawn with 30% probability.

8 Determination of racemization barriers of 10 and 11

General procedure. A solution of compound (*M*)-**10** (2.3 mg) or (*M*)-**11** (2.0 mg) in dodecane (5 mL) was heated in an oil bath at 217 °C under argon atmosphere. In time intervals, a small sample (0.1 mL) of the solution was taken. It was cooled to room temperature, diluted with CH_2Cl_2, and analyzed using HPLC on a chiral column (Chiralpak IA). Dependencies of the percentage of the major enantiomers on time are shown in Figures S21 and S23. During the racemization process of **10**, degradation was detected after 20 hours. In the case of **11** degradation was observed already after 4 hours.

Racemization rate constant *k* was calculated using first-order kinetics with the equation:

$$\ln \frac{[M]_0 - [M]_{eq}}{[M]_t - [M]_{eq}} = 2kt$$

where $[M]_0$ is the initial concentration of the major enantiomer, $[M]_{eq}$ is the equilibrium concentration, and $[M]_t$ is the concentration of the major enantiomer at time *t*. Dependencies of $\ln \frac{[M]_0 - [M]_{eq}}{[M]_t - [M]_{eq}}$ on time *t* are shown in Figures S22 and S24.

The activation barrier energy for racemization $\Delta G^{\ddagger}$ is calculated from Eyring's equation:

$$\Delta G^{\ddagger} = -RT \ln \frac{kh}{\kappa k_B T}$$

where *R* is gas constant, *T* is thermodynamic temperature, *k* is reaction rate constant, *h* is Planck's constant, κ is transmission coefficient, and k_B is Boltzmann constant. The transition state associated with racemization can proceed to form either the original enantiomer or its mirror image; therefore, the transmission coefficient is taken to be κ = 0.5.

Racemization of helicenes is also defined by its half-life $t_{1/2}$, which can be calculated from the equation:

$$t_{1/2} = \frac{\ln 2}{k}$$

Table S11. Experimental conditions and results of determination of racemization barrier.[a]

Compound	T (K)	k (s^{-1})	$\Delta G^{\#}$ (kcal/mol)	$t_{1/2}$ (h)
(*M*)-**10**	490.15	9.04×10^{-6}	39.8	21.3
(*M*)-**11**	490.15	5.09×10^{-6}	40.4	37.8

[a] Mobile phase for HPLC analysis: Heptane/*i*-PrOH = 95/5, flow rate 0.25 mL/min, 25° C.

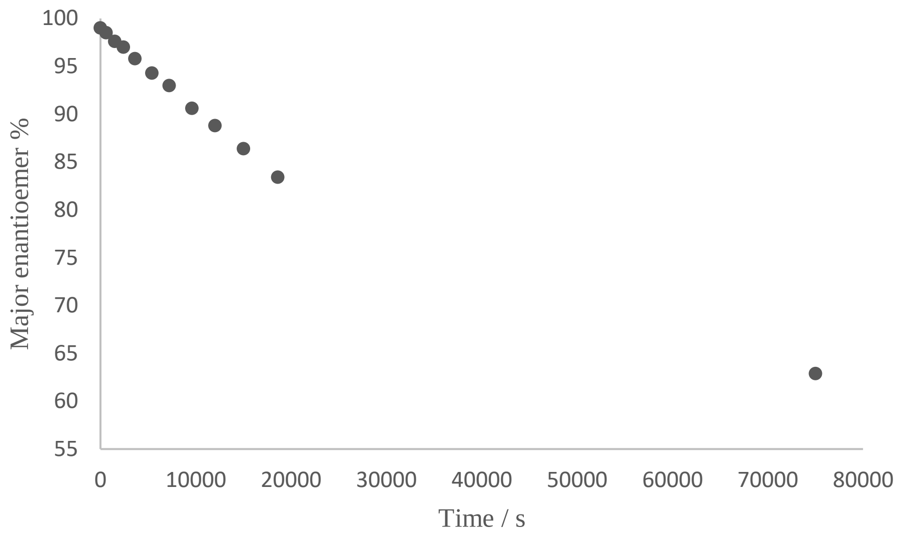

Figure S21. Dependence of % of the major enantiomer of **10** on time.

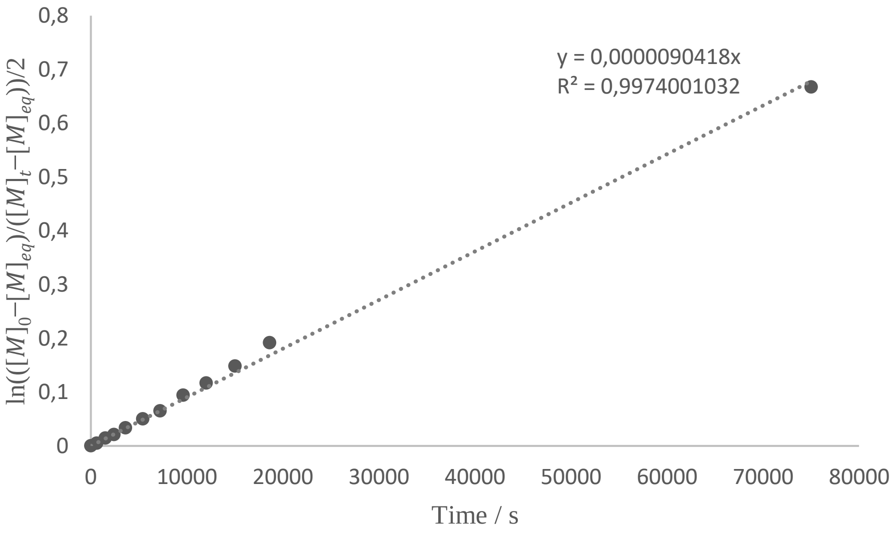

Figure S22. Dependence of $\ln\frac{[M]_0-[M]_{eq}}{[M]_t-[M]_{eq}}$ on time t for compound **10**.

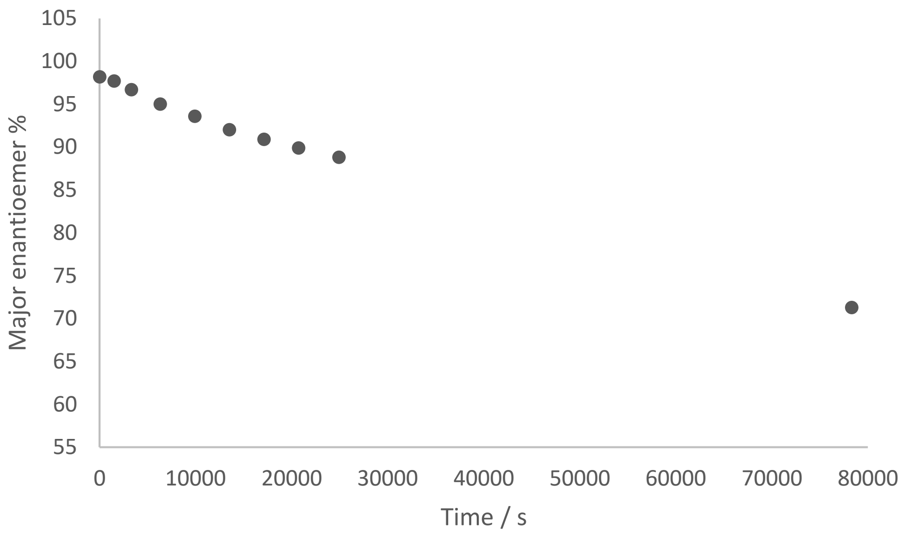

Figure S23. Dependence of % of the major enantiomer of **11** on time.

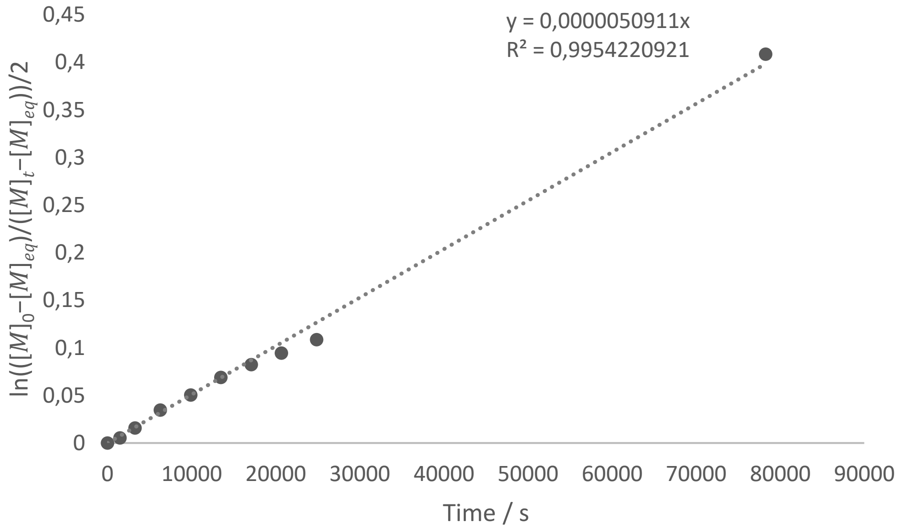

Figure S24. Dependence of $\ln \frac{[M]_0 - [M]_{eq}}{[M]_t - [M]_{eq}}$ on time t for compound **11**.

As for compound **9**, no racemization was noticed under the above conditions. Attempting racemization in tridecane at 230 °C resulted in decomposition to unknown species, but, interestingly, racemization wasn't observed.

9 DFT calculations

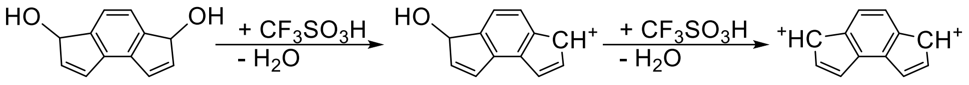

Relative energy	SI_I	SI_II	SI_III
B3LYP-D3/def2TZVP	0	98.5	298.3
ωB97X-D/def2TZVP	0	107.8	315.3
CCSD(T)/aug-cc-pVTZ	0	108.1	313.6

Figure S25. Comparison of reaction energies for model system. The systems were optimized at the B3LYP/def2-SVP level in SMD solvation by HFIP. The energies are in kcal·mol^{-1}.

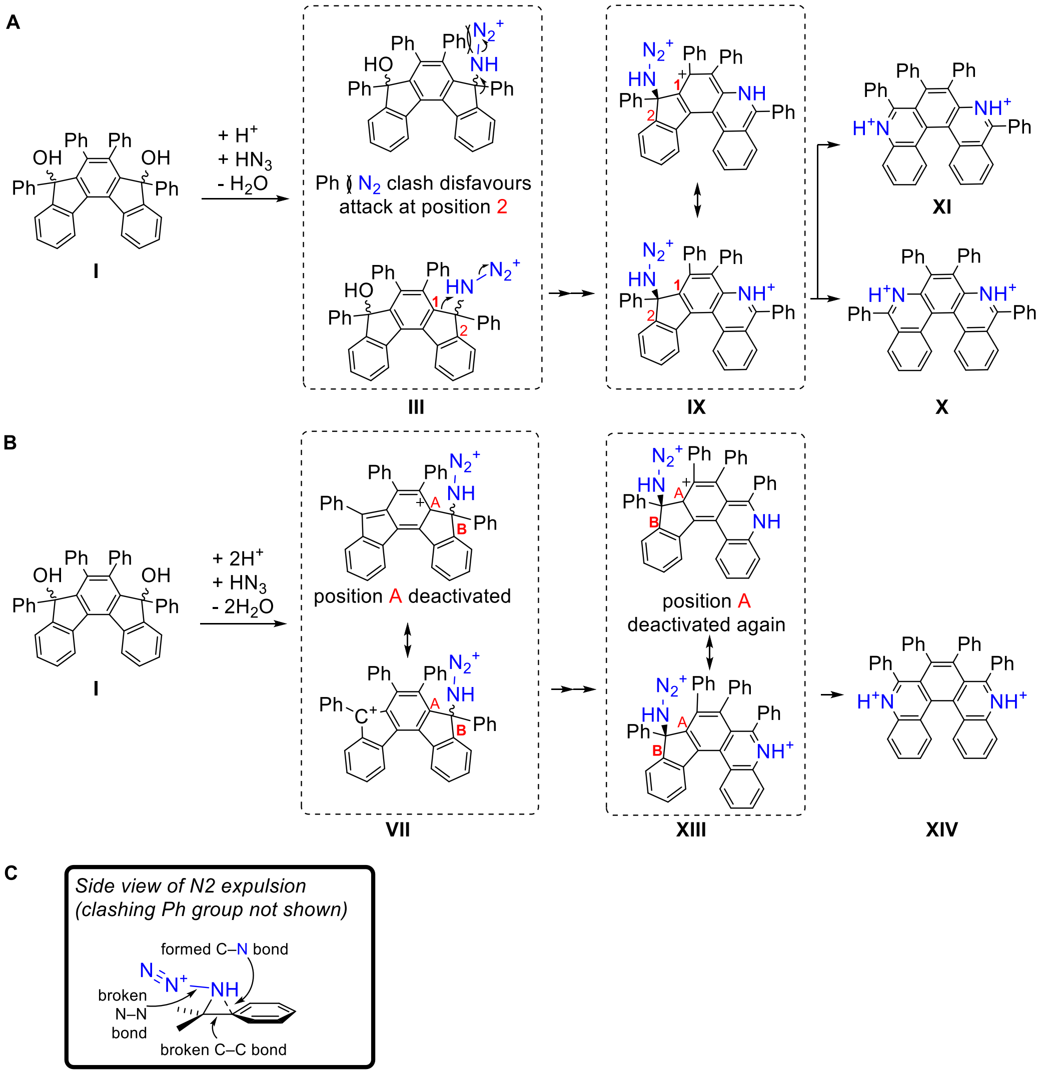

Figure S26. Key reaction intermediates of the Schmidt rearrangement of model reactant I in (A) less strongly acidic conditions and (B) more strongly acidic conditions. Panel (C) shows the geometry of the N_2 expulsion step. The whole reaction scheme with calculated Gibbs free energies can be found in Figure 1.

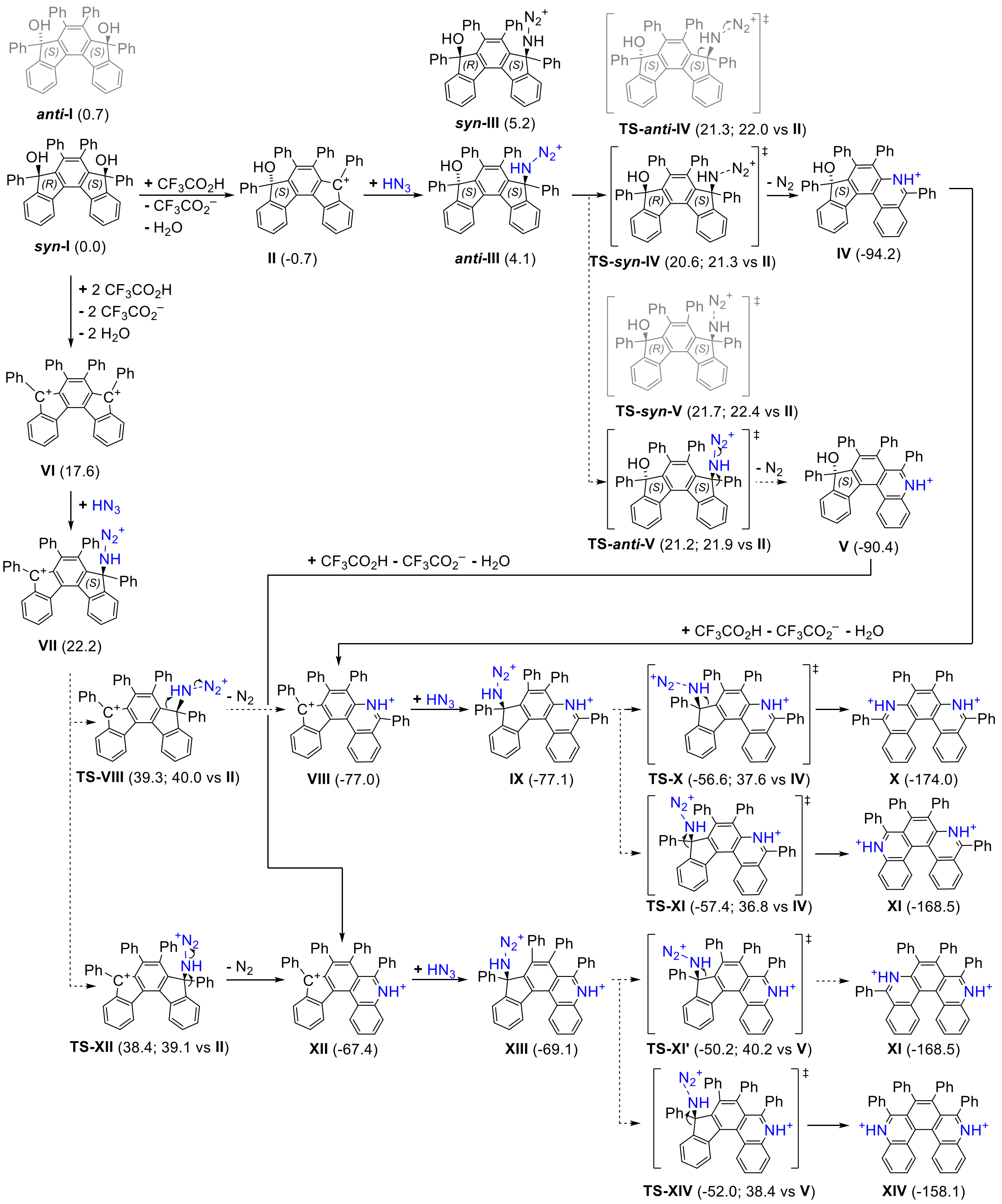

Figure S27. Relative Gibbs free energies for the reaction of **I** with CF_3CO_2H calculated at ωB97X-D/def2-TZVPD//B3DLYP-D3JB/def2-SVP level in HFIP (SMD optimization, COSMO-RS single point energies) at T = 298.15 K and c = 1 M. Alternative stereoisomers are depicted in gray. For transition states, we also list energy relative to the preceding most stable intermediate. The *non-preferred* pathways are indicated by dashed arrows.

9.1 XYZ Structures

Format of individual record:

number_of_atoms (new line) name charge multiplicity key=value

Meanings of keys (all values are in atomic units except where noted):

NImag: number of imaginary frequencies at B3LYP-D3BJ(HFIP,SMD)/def2-SVP level.

CorrGibbs: Gibbs free energy correction at B3LYP-D3BJ(HFIP,SMD)/def2-SVP level (1 atm, 298.15 K)

ZeroPointC: Vibrational zero-point energy at B3LYP-D3BJ(HFIP,SMD)/def2-SVP level

dipole: dipole moment at B3LYP-D3BJ(HFIP,SMD)/def2-SVP level

polarizability: lower-triangular part of polarizability tensor at B3LYP-D3BJ(HFIP,SMD)/def2-SVP level

E_bp86_TZVPD_GAS: gas-phase energy at BP86-DB3J/def2TZVPD level

E_b3lyp_TZVPD_GAS: the same at B3LYP-D3JB/def2-TZVPD level

E_wb97_TZVPD_GAS: gas-phase energy at ωB97X-D/def2-TZVPD level

G_solv_hfip: COSMO-RS free energy of solvation in HFIP

E_b3lyp_d3bj_tzvp_smd: B3LYP-D3BJ(HFIP,SMD)/def2-TZVP energy

E_b3lyp_d3bj_def2SVP_smd_hfip: B3LYP-D3BJ(HFIP,SMD)/def2-SVP energy

LowestF: lowest vibrational frequency at B3LYP-D3BJ(HFIP,SMD)/def2-SVP level [cm^{-1}]

E_ccsd_t_: gas-phase energy at CCSD(T)/aug-cc-pVTZ level (present only in a few cases)

```
9
CF3SO3H 0 1 NImag=0 CorrGibbs=0.005121 ZeroPointC=0.038534
dipole=[0.0800496,1.23586,0.9001318] polarizability=[40.9332347,1.2165305,46.7419596,-
0.9404509,-0.7090604,41.4067807] E_bp86_TZVPD_GAS=-962.4495971402
E_b3lyp_TZVPD_GAS=-962.0849178218 E_wb97_TZVPD_GAS=-962.1744337043
G_solv_hfip=-0.011619473970269129 E_b3lyp_d3bj_tzvp_smd=-962.3378432 E_ccsd_t_=-
960.9852391969249 E_b3lyp_d3bj_def2SVP_smd_hfip=-961.448163086 LowestF=29.8882
C   -0.67572601476451   0.73285993876450  -0.12323961203332
S    0.68274182401808  -0.53442697228725   0.06773740408464
O   -0.16538475636541  -1.74523235018847   0.67691559201397
O    1.54705365712735  -0.05790842364705   1.12713876654764
O    1.15320330121189  -0.85323271408115  -1.26886820225008
F   -0.15188008262675   1.83255803473479  -0.64425919180729
F   -1.19742521598962   0.99897257487766   1.06360249174260
F   -1.61071220895905   0.25230085687986  -0.93156929133119
H   -0.50833314331395  -2.34982337108826  -0.01581612108363


8
CF3SO3m -1 1 NImag=0 CorrGibbs=-0.005722 ZeroPointC=0.027136 dipole=[1.587875,-
0.295007,1.1663082] polarizability=[40.6091979,0.4154682,42.7542911,-
1.6171453,0.3121793,41.6372526] E_bp86_TZVPD_GAS=-961.9593585387
E_b3lyp_TZVPD_GAS=-961.5969175309 E_wb97_TZVPD_GAS=-961.6829956564
G_solv_hfip=-0.11468252349047658 E_b3lyp_d3bj_tzvp_smd=-961.9153882 E_ccsd_t_=-
960.495604642263 E_b3lyp_d3bj_def2SVP_smd_hfip=-961.009898213 LowestF=37.4713
C   -0.95041895062367   0.00213412037527  -0.00004302610118
S    0.91047647210180  -0.00162906167420   0.00007888845210
O    1.23214285039508  -0.74994876975347   1.23367258654000
O    1.23898638728124   1.43948609196203   0.03007019012250
O    1.23313654790258  -0.69724352475572  -1.26375045623026
F   -1.42485813296263   0.62985831811793  -1.08348540603477
F   -1.42507969426418   0.62739529686714   1.08476400055104
F   -1.42775507660853  -1.24893029443921  -0.00138333030343

8
CF3CO2H 0 1 NImag=0 CorrGibbs=0.007548 ZeroPointC=0.038778 dipole=[-
1.0269703,0.5107659,0.2246051] polarizability=[31.2924663,-3.8963456,25.6058203,-
0.9124843,3.3654633,35.7923669] E_bp86_TZVPD_GAS=-527.0764953023
E_b3lyp_TZVPD_GAS=-526.831972814 E_wb97_TZVPD_GAS=-526.8812134669
G_solv_hfip=-0.00824741332823407 E_b3lyp_d3bj_tzvp_smd=-527.0229003
E_b3lyp_d3bj_def2SVP_smd_hfip=-526.387865995 LowestF=34.8704
C    0.58654313440793   0.09061094414952   0.00563929042316
C   -0.90607716760070  -0.29328315864566  -0.01458781732413
O   -1.64900587123031   0.80122192782636   0.05073747766076
O   -1.28274077320011  -1.43223382950203  -0.08344959560660
F    1.34220170429935  -1.00392682845936  -0.01263709333039
F    0.87633549723425   0.79603600932925   1.10603790841904
F    0.88936548237333   0.84041153002559  -1.06185766854705
H   -2.59994680056236   0.57144210232289   0.03149978609816


7
CF3CO2m -1 1 NImag=0 CorrGibbs=-0.004558 ZeroPointC=0.026237 dipole=[1.8315479,-
0.414522,1.2655448] polarizability=[31.0333702,-3.6133509,26.1650706,-
3.7677925,4.2319914,36.1922] E_bp86_TZVPD_GAS=-526.5516360313
E_b3lyp_TZVPD_GAS=-526.306642958 E_wb97_TZVPD_GAS=-526.3505999906
G_solv_hfip=-0.1274905691569912 E_b3lyp_d3bj_tzvp_smd=-526.5690777
E_b3lyp_d3bj_def2SVP_smd_hfip=-525.920130064 LowestF=48.2848
C    0.52570023366464   0.02736660471922   0.00500898505096
C   -1.02548565290428  -0.09454887030120  -0.00664402510560
O   -1.60198908405502   1.00332938130029   0.13624180808478
O   -1.48503223277541  -1.24104067008282  -0.16211666528292
F    1.15759044569083  -1.15602378543773  -0.08632752576252
F    0.96150869204155   0.62395380898952   1.13618604344029
F    0.95811009005443   0.78815707686511  -1.02576861790966
3
H2O 0 1 NImag=0 CorrGibbs=0.002751 ZeroPointC=0.021077 dipole=[0.3263252,-
0.8256376,0.2681946] polarizability=[5.3682,-0.8886772,5.4600547,-1.9729999,-
0.5676011,6.6496255] E_bp86_TZVPD_GAS=-76.47181420523 E_b3lyp_TZVPD_GAS=-
76.43031963622 E_wb97_TZVPD_GAS=-76.4407178721 G_solv_hfip=-0.010735312020640635
E_b3lyp_d3bj_tzvp_smd=-76.4628692 E_ccsd_t_=-76.3421857633823
E_b3lyp_d3bj_def2SVP_smd_hfip=-76.357090423 LowestF=1628.9966
O    0.12067043127680  -0.00000081465925   0.00000000000000
H   -0.48263225436239  -0.75890674185979  -0.00000000002024
H   -0.48273119585202   0.75891325913377   0.00000000002024

4
HN3 0 1 NImag=0 CorrGibbs=-0.001399 ZeroPointC=0.021568
dipole=[0.5684708,0.7854679,0.0]
polarizability=[47.4145489,3.028476,12.7794705,0.0,0.0,10.2739698] E_bp86_TZVPD_GAS=-
164.8871802336 E_b3lyp_TZVPD_GAS=-164.7791488372 E_wb97_TZVPD_GAS=-
164.7931499901 G_solv_hfip=-0.005321337984535465 E_b3lyp_d3bj_tzvp_smd=-164.8624817
E_b3lyp_d3bj_def2SVP_smd_hfip=-164.666160049 LowestF=550.8007
N   -0.94994336378966   0.59954782711065   0.00000000208159
N    0.08698750292372  -0.05995075413865  -0.00000000822411
N    1.11694619640434  -0.53806999175631  -0.00000000140617
H   -1.77793234876876  -0.01068956850982   0.00000005284081


2
N2 0 1 NImag=0 CorrGibbs=-0.01276 ZeroPointC=0.00568 dipole=[0.0,0.0,-1e-07]
polarizability=[6.6188137,0.0,6.6188137,0.0,0.0,15.1455874] E_bp86_TZVPD_GAS=-
109.5815853456 E_b3lyp_TZVPD_GAS=-109.5221928922 E_wb97_TZVPD_GAS=-
109.5326155212 G_solv_hfip=0.0005079923302748182 E_b3lyp_d3bj_tzvp_smd=-109.5696849
E_b3lyp_d3bj_def2SVP_smd_hfip=-109.435998455 LowestF=2493.282
N   -0.55003495130000   0.00000000000000   0.00000000000000
N    0.55003495130000   0.00000000000000   0.00000000000000


78
TS-syn-IV 1 1 NImag=1 CorrGibbs=0.546126 ZeroPointC=0.617001 dipole=[-4.7660034,-
1.5283468,-0.9167286]
polarizability=[935.8387928,5.7835404,833.3268139,5.3988811,9.9681,589.4175127]
E_bp86_TZVPD_GAS=-1934.916136486 E_b3lyp_TZVPD_GAS=-1933.636059366
E_wb97_TZVPD_GAS=-1933.936452378 G_solv_hfip=-0.09178782533249617
E_b3lyp_d3bj_tzvp_smd=-1934.8802301 E_b3lyp_d3bj_def2SVP_smd_hfip=-1932.80638837
LowestF=-613.7327
O   -1.06294097942261   0.41185183987680   3.90358220278590
C   -0.87579848227485  -0.67475184293192   3.01443281466218
C   -2.17109972035254  -1.45611354091241   2.77840946008437
C   -2.21502258516735  -2.47618662381192   1.81702730441500
C   -3.40614693475658  -3.15657692689760   1.56459715340073
C   -4.56776419789819  -2.82777915497551   2.27325214820579
C   -4.52115748642938  -1.82849144861870   3.24708488665262
C   -3.32581717268202  -1.14907161094714   3.50341350766090
C    0.21828843397222  -1.54600809890573   3.60852500301789
C    1.32592520471055  -1.64417256201594   2.74174496733891
C    2.48280846695970  -2.29305436227211   3.19154235240866
C    2.48918518523745  -2.87183137390333   4.46540545264161
C    1.36535440745144  -2.80100202660419   5.29706642174286
C    0.21887659030969  -2.11798693129538   4.87259997233962
C    1.02139887846302  -0.88503377714403   1.50871066174194
C   -0.23575081365202  -0.24510460506564   1.68637991467547
C   -0.80118013871284   0.62558750114886   0.75708664766992
C   -2.14555808648673   1.22807526472864   0.99925990710120
C   -2.26174507239158   2.43831600220974   1.69987168308186
C   -3.52322868019845   2.97812119185541   1.97045777560693
C   -4.67539471924388   2.31691463499105   1.53758124505493
C   -4.56348376087307   1.11830596561163   0.82619480416410
C   -3.30594616067338   0.57741936358274   0.55596350566954
C   -0.06239558041266   0.95236912544984  -0.40561646201346
C   -0.62525254440544   1.91787684962755  -1.38860023866828
C   -0.19743581549383   3.25117487467521  -1.42436836674624
C   -0.73971268223930   4.13833966493598  -2.35842226624753
C   -1.71255663143401   3.70008391994738  -3.25959678924671
C   -2.15393134575235   2.37330624039758  -3.21651095937717
C   -1.62115168691035   1.48862074310333  -2.28017165379841
C    1.14828948267376   0.27858409322101  -0.61050608629468
C    1.68539029553922  -0.69490638467974   0.29043116704022
C    2.81771683677183  -1.36801510538199  -0.37450937405125
C    3.03907718321587  -0.75452647635389  -1.62571799630785
C    4.02771582689161  -1.17957343967612  -2.49982738425148
C    4.81482008626409  -2.27846459409756  -2.12715963429911
C    4.57791214865391  -2.93228094131155  -0.91386897508150
C    3.58208129882054  -2.49164257747795  -0.03527438760804
C    2.06736474374410   0.39309424901321  -1.84561203768721
C    1.37601761681884   0.41538842143396  -3.19003294017443
C    0.78876219876899  -0.77706354072159  -3.63810474232215
C    0.08633372804105  -0.80540937402821  -4.84145897858793
C   -0.02702570928536   0.35429995970094  -5.61647928389027
C    0.56291659123486   1.53977658478268  -5.17657307051759
C    1.25767991818447   1.57402552784287  -3.96490785696852
N    2.63616998363986   1.68163742385689  -1.45942731590736
N    3.91248006674872   2.06885905677193  -2.44924530914602
N    4.44029265316329   2.74213521208951  -3.14789927317403
H   -1.70603533514827   1.03464447929987   3.52582989735832
H   -1.31589762655922  -2.73314733314503   1.25104460322633
H   -3.43081972871598  -3.94426703302866   0.80670520437425
H   -5.50386800334420  -3.35400585795255   2.06833585009566
H   -5.42142660423466  -1.56903174605800   3.81068295691580
H   -3.29662131934521  -0.36381538977880   4.26040265543773
H    3.39075999249266  -2.31754547527380   2.59288696420515
H    3.39218719302269  -3.37721057459962   4.81733446940529
H    1.39116857160357  -3.26169111887431   6.28792121060266
H   -0.65463869894422  -2.02265938503732   5.52244619648275
H   -1.35910316584164   2.95582750757378   2.03487371493188
H   -3.60359677596492   3.91931053003732   2.52060442944739
H   -5.66153172525082   2.73650125552832   1.75317537061755
H   -5.46127232206879   0.59673338804116   0.48492480952453
H   -3.22204822539600  -0.36608533327250   0.01457687980743
H    0.55794149073724   3.59584075914383  -0.71567020080347
H   -0.39900221127276   5.17680735366093  -2.38054202861240
H   -2.13091182564654   4.39310454140910  -3.99425855977545
H   -2.91319082341972   2.02398760684308  -3.92066706427396
H   -1.95595150653604   0.45013859615168  -2.25552246584664
H    4.16916861500990  -0.69700000996787  -3.46989818594982
H    5.59211985584241  -2.64347496883364  -2.80267881240495
H    5.17072186010032  -3.81108762588576  -0.64852027853075
H    3.39418281093422  -3.05043092043846   0.87804986407957
H    0.87021066122602  -1.68362154478557  -3.03379712407118
H   -0.37514953640935  -1.73753572473551  -5.17721409393871
H   -0.57915000341483   0.33198571136001  -6.55950156837046
H    0.47003748069421   2.45425824978989  -5.76703478216924
H    1.65976714387028   2.52585979688352  -3.62135006655495
H    3.19672398249099   1.56657654852535  -0.59810981333023


78
TS-XI 2 1 NImag=1 CorrGibbs=0.552041 ZeroPointC=0.620883 dipole=[0.8259336,-
1.6731418,1.5302] polarizability=[981.5312061,-43.9422212,1008.3051429,-2.3730982,-
15.9430326,529.8176584] E_bp86_TZVPD_GAS=-1914.160359805 E_b3lyp_TZVPD_GAS=-
1912.891122529 E_wb97_TZVPD_GAS=-1913.180763529 G_solv_hfip=-0.18629427097353965
E_b3lyp_d3bj_tzvp_smd=-1914.2562136 E_b3lyp_d3bj_def2SVP_smd_hfip=-1912.21302504
LowestF=-525.0488
N   -4.54124724457529   2.79731290038092  -0.89198943854113
N   -3.75086646721960   2.23379227425063  -0.35823146445134
N   -3.40076296298358   1.36220405040895   1.08085468189965
C   -2.84325633445625   0.06520285415188   0.77932077764672
C   -3.75656385402296  -0.84248230944193  -0.02155458164616
C   -3.24064568639819  -2.04212910732765  -0.53244923071388
C   -4.06284333396846  -2.90950302727385  -1.24999605646456
C   -5.40789379726939  -2.58919000336390  -1.46296149512988
C   -5.92783577622087  -1.40574698179564  -0.93646620402527
C   -5.10946963280310  -0.53721347013582  -0.20824712207691
C   -2.77704029095500  -0.31991094064710   2.26740454987078
C   -1.43181862217666  -0.58047971668023   2.63472995977447
C   -1.15928026401269  -0.82626873681433   3.98281247390991
C   -2.21757105423489  -0.86671977279155   4.90071936402030
C   -3.54752157645830  -0.68309749120198   4.50212601684364
C   -3.84003211548118  -0.38891846819945   3.16965188220023
C   -0.58087141535237  -0.45551447173626   1.43458541013085
C   -1.36339028222270   0.06489786084465   0.38716111409573
C   -0.80078651480126   0.61289365819870  -0.78156174491253
C   -1.62463598234649   1.30305137863505  -1.81601000962820
C   -1.44005531335786   2.68703276999289  -2.01778512393437
C   -2.18156855023900   3.36400074529543  -2.98074813275659
C   -3.11880021182452   2.67132405574021  -3.75581429071078
C   -3.28444739118855   1.29173873595315  -3.58581596239888
C   -2.53867595431681   0.60740239151563  -2.62787319746910
C    0.59720375266148   0.61772787004179  -0.90240919639444
C    1.26742187966399   1.24328740158904  -2.07595541296288
C    2.08019328825485   2.37786753645252  -1.91662270496683
C    2.68687936914684   2.97102759828666  -3.02645170976297
C    2.48571664943776   2.43930465045525  -4.30254382170911
C    1.67835034374067   1.30921666508963  -4.46713295309079
C    1.07228995916025   0.71266049024847  -3.36143758364685
C    1.36917224208607  -0.02940371539440   0.09215466245691
```

C 0.81149927004284 -0.66272965081297 1.22984149491221
C 1.69052259944378 -1.49513796822250 2.03259609221389
C 3.10996665384042 -1.38520813780511 1.86368899071509
C 3.98114684384066 -2.07336678915409 2.74592623731210
C 3.47167116235024 -2.95335349130200 3.68072292837892
C 2.08510422743878 -3.19123809434240 3.73065619347606
C 1.21250579146211 -2.47315025558058 2.92841637465697
C 3.61416166402091 -0.64559073831439 0.74949438424851
C 5.03710747406873 -0.48989244289427 0.40302718803402
C 5.53460323018098 0.79779845775854 0.12848743914658
C 6.87169245425287 0.96579420728794 -0.22826760406274
C 7.71455612778043 -0.14455936934040 -0.33250749259517
C 7.21857382152106 -1.42723656964251 -0.07989876633982
C 5.88672241206628 -1.60492885961283 0.29158375908784
N 2.74259458332200 -0.07792946083303 -0.07602413916371
H -2.65066117317904 1.98990947574129 1.41810509503141
H -2.18973942640435 -2.29558104041667 -0.37917658653147
H -3.64942069331564 -3.83863383007578 -1.65014303548316
H -6.04969470648399 -3.26543269334431 -2.03325705325085
H -6.97994401393727 -1.15175775843143 -1.08735971788366
H -5.54392371834858 0.36996030017063 0.21226373123915
H -0.14206424440906 -0.97356763813163 4.34098795997634
H -1.99349028026227 -1.05452428566030 5.95354103340557
H -4.35296893658387 -0.74533203358613 5.23670854745989
H -4.86399238493891 -0.20641429445744 2.83972373703851
H -0.72469028245862 3.22956801135062 -1.39744984472901
H -2.04207576865477 4.43887508027579 -3.11825105264848
H -3.71205194249917 3.20451615843678 -4.50289024033889
H -4.00009875758782 0.74521535950097 -4.20453590131377
H -2.65605848611748 -0.46937216091855 -2.51515356276689
H 2.21935948954839 2.81118028642085 -0.92257039637548
H 3.31192380708479 3.85749585448344 -2.89253398971645
H 2.95803518174237 2.90591683929315 -5.17078846152147
H 1.51941544922726 0.88901954238503 -5.46349448083070
H 0.44071862209689 -0.16902710060183 -3.49046656118010
H 5.05684284975798 -1.91855506315270 2.67431416471122
H 4.14516431083535 -3.48688029780814 4.35467717879375
H 1.68743479408113 -3.94510562211274 4.41434679784479
H 0.14820421058800 -2.69255737124627 2.97254385670619
H 4.88276993752808 1.66930357890031 0.22165263824040
H 7.25737920091781 1.96926069302251 -0.42305618472768
H 8.76148109623782 -0.01045810269620 -0.61608567293610
H 7.87131965426727 -2.29770708981325 -0.17911433708274
H 5.49675427739372 -2.61023823190919 0.45735192038776
H 3.11370299994239 0.36771259867324 -0.91973843412037

76
syn-I 0 1 NImag=0 CorrGibbs=0.54095 ZeroPointC=0.608564 dipole=[-0.8365464,-0.0061676,-0.8730608] polarizability=[759.7699776,-10.8438641,790.9812056,4.2957745,-16.1149941,548.4899005] E_bp86_TZVPD_GAS=-1846.157449205 E_b3lyp_TZVPD_GAS=-1844.947513076 E_wb97_TZVPD_GAS=-1845.251911183 G_solv_hfip=-0.053569039814205965 E_b3lyp_d3bj_tzvp_smd=-1846.0908802 E_b3lyp_d3bj_def2SVP_smd_hfip=-1844.11798481 LowestF=15.1152
O 0.64676753804545 -1.05408054971988 3.71898429998458
C 1.15890406776941 -0.10305961276576 2.79818515063816
C 2.65076943320274 -0.37101123956983 2.67653096805528
C 3.00347664268033 -0.71134923997354 1.35381338504151
C 4.31563645589826 -1.12653918837032 1.09448141362454
C 5.25030404811892 -1.14187437770978 2.13722407253785
C 4.89196782531442 -0.76188710748538 3.43499317138300
C 3.57180160416753 -0.38408605431018 3.71386028144200
C 0.87010746198531 1.33220798835278 3.24701436057480
C 1.17485596076687 2.41084867208106 2.40400718252534
C 0.87311386800233 3.71680357212312 2.78977208961096
C 0.26279673497649 3.96221904001930 4.02580954135540
C -0.02392625742384 2.89266074051013 4.87602535021186
C 0.28445632205198 1.58368635793256 4.49105286666916
C 0.68614979080843 -0.35639456949920 1.36194783563605
C 1.78510022108691 -0.64242819903791 0.51213044395652
C 1.52937786802755 -0.81782128266977 -0.85821379294394
C 2.39162958451295 -0.92185723068327 -2.05876285095575
C 3.76614831158891 -0.74354677580952 -2.25426890585375
C 4.29729270045333 -0.85318656668063 -3.54577869710068
C 3.47377140067818 -1.11930618322193 -4.64380071398376
C 2.08937985532843 -1.24620116180076 -4.46508414001548
C 1.56662680740354 -1.14043992024415 -3.18325795482047
C 0.09613138147657 -1.18694941657620 -2.78368566068188
O -0.43179052948712 -2.50399499406866 -2.90385459574895
C -0.74102152783846 -0.16452993893112 -3.55099402310884
C -1.87058987051610 -0.55329426582058 -4.27577191353935
C -2.66309573650240 0.40440668335481 -4.91703656068433
C -2.33037597360031 1.75828515892744 -4.84155086808080
C -1.19137075243766 2.15074427185384 -4.12824950209514
C -0.40109183200822 1.19479548318759 -3.49033864561052
C 0.17843514121171 -0.86669996323344 -1.28740006239670
C -0.90153415853949 -0.62274885365354 -0.44077650662891
C -2.30398723565175 -0.63294866704463 -0.94588250934948
C -2.95498755660684 -1.84113574959114 -1.23064182516483
C -4.25716365710272 -1.83932436261814 -1.73758893618265
C -4.92030547589751 -0.63096792753956 -1.96765612627603
C -4.28016067007437 0.57792343314737 -1.67590908911414
C -2.98337734174634 0.57627353608529 -1.16099613373073
C -0.63246611961291 -0.31576442848671 0.91761836413349
C -1.74355367945369 0.00590311974622 1.86173382834851
C -2.40669217255708 -1.02281553005694 2.54912140635818
C -3.41665112196763 -0.72421174477552 3.46954676375894
C -3.77825077450601 0.60449683240600 3.70582853588917
C -3.13188157154031 1.63312386492043 3.01314567388911
C -2.12277677437472 1.33620176060911 2.09600012105073
H -0.32368789343782 -1.01207882860139 3.71507276726146
H 4.61554294916684 -1.47966465144020 0.11062392352854
H 6.27352239399516 -1.46693349047513 1.93077266496023
H 5.63693595775764 -0.77939602087836 4.23477748813666
H 3.26459756246246 -0.11664521381234 4.72831414683516
H 1.63725284978871 2.22683455453108 1.43078371268547
H 1.10821954167494 4.54850867248995 2.11993266390767
H 0.01631588997965 4.98486677844755 4.32384497051998
H -0.49598414097599 3.07366254840410 5.84567315721589
H 0.05120788649174 0.74871018325650 5.15367825489234
H 4.42738880740776 -0.48272556873715 -1.43122116143265
H 5.37145579333845 -0.71432215522120 -3.69421581268989
H 3.90526899509745 -1.19983369522662 -5.64483432257423
H 1.43001295336073 -1.40147465411547 -5.32356485982853
H -0.19517796372991 -2.86468295000041 -3.77229621870248
H -2.14914506909382 -1.60706272782844 -4.31134688219968
H -3.55267310650356 0.08785518274555 -5.46841360821195
H -2.95488079012238 2.50773017856665 -5.33528219854414
H -0.92144510268418 3.20854326094448 -4.06535482341021
H 0.47977583361976 1.50820854473807 -2.92337511700996
H -2.42797519756508 -2.78411280746722 -1.07230522257877
H -4.75368759512394 -2.78788286105547 -1.95993506139143
H -5.93556923303978 -0.63036651383504 -2.37363470781410
H -4.79091380393666 1.52740778539930 -1.85686628949121
H -2.47900186077014 1.52097170839884 -0.94939295108131
H -2.12670081060741 -2.06244773838042 2.35957562630807
H -3.92214107665975 -1.53443599286393 4.00178946038777
H -4.56512954608084 0.83877358160067 4.42775776401114
H -3.41039627839640 2.67482580064760 3.19256112208241
H -1.61064337919657 2.14256569749118 1.56865955010818

76
VII 2 1 NImag=0 CorrGibbs=0.537197 ZeroPointC=0.604293 dipole=[-0.8098565,-2.9932135,-2.2439487] polarizability=[1064.028547,-0.8859493,996.5684832,-66.3943785,30.1221087,514.5450196] E_bp86_TZVPD_GAS=-1858.728455987 E_b3lyp_TZVPD_GAS=-1857.493486779 E_wb97_TZVPD_GAS=-1857.778223364 G_solv_hfip=-0.1921859589126046 E_b3lyp_d3bj_tzvp_smd=-1858.8420802 E_b3lyp_d3bj_def2SVP_smd_hfip=-1856.86562977 LowestF=17.1495
N -0.42894565539887 2.10463183001720 -3.00974315621464
N -1.00792945637012 1.15444159794398 -3.10958654162421
N -1.66792418699347 0.08086369721083 -3.13329630475664
C -1.18758424757705 -1.13659844016649 -2.33808688795928
C -0.13421674510660 -1.86319998312905 -3.15221383894531
C 1.13979045028641 -1.71585480531047 -2.56515554252401
C 2.21871636303503 -2.42277024436801 -3.10862303589191
C 2.01684824240910 -3.19522440109839 -4.25634906595939
C 0.75527472747345 -3.28637498594181 -4.85719138972252
C -0.34250398532747 -2.62824580058705 -4.29213317506977
C -2.40867636832177 -1.98395889243102 -2.02398065975449
C -3.72035668537952 -1.57968288134878 -2.28526405486701
C -4.78872162840781 -2.40800370639826 -1.93076530825331
C -4.55396148057254 -3.63390298173561 -1.30764514144614
C -3.24029540731964 -4.03917791792381 -1.04770597654426
C -2.17110360420310 -3.22244206031856 -1.41022945250426
C -0.38560250648440 -0.62964022608838 -1.13309953739096
C 1.01447004689140 -0.87179920316198 -1.36236562989718
C 1.91624736165803 -0.30208845376430 -0.47995246545983
C 3.38876560283057 -0.14054234594190 -0.47403774195914
C 4.38070110371337 -0.41249339361535 -1.40095057906545
C 5.72067275825160 -0.10940594129654 -1.07642309524371
C 6.07670177243064 0.44024036838370 0.15843467298417
C 5.08661689586278 0.74901707807189 1.09188313453836
C 3.74448799154384 0.50528763892053 0.75306815456205
C 2.55462414697520 0.79122296448292 1.50027428772522
C 2.54147101805931 1.37684012179513 2.81994997432736
C 1.73673288430125 0.82491671315417 3.84301182426851
C 1.70621587743938 1.41429700255814 5.09870871683202
C 2.44746186692250 2.57772955657260 5.34698471571719
C 3.24976940454482 3.13092695327198 4.34385134094602
C 3.31990710377399 2.52581603824046 3.09303213157982
C 1.41250287994868 0.35763523425709 0.69897416518503
C 0.04187125099642 0.58992455715803 0.91085053212285
C -0.46000449799727 1.49752598167103 1.97182414965501
C -0.02689613067859 2.83354544064874 1.97620066503194
C -0.45748567132097 3.71084696509728 2.97083832103264
C -1.33004008712120 3.26448754984553 3.96758565224862
C -1.77742804169493 1.93999901919466 3.95835609198170
C -1.35199278407350 1.06075081890817 2.96307316331494
C -0.88937259251795 0.04547122271304 -0.03599576292109
C -2.35758150611690 0.22823522376550 0.16193917001881
C -3.01199031751670 1.38848139381969 -0.27039653929584
C -4.39059855667582 1.52557951935823 -0.08575753715784
C -5.11911211426192 0.51242112656981 0.54227861222461
C -4.46501020710437 -0.63821522502877 0.99313774373039
C -3.09123945797574 -0.78000030339512 0.80416605707210
H -2.24087643499680 -0.05206393228111 -3.98199374038969
H 3.19569394443148 -2.42045954468733 -2.63177252907679
H 2.85817135435711 -3.74813054054382 -4.68093511545375
H 0.62137125616813 -3.89633056916539 -5.75352713514096
H -1.34138272039264 -2.73085478219384 -4.72233984171439
H -3.93936892550014 -0.60828391586764 -2.72916321683969
H -5.81090841826945 -2.07890687760670 -2.13162172782185
H -5.39229505586608 -4.27462361455096 -1.02304245712387
H -3.04656780340716 -4.99858426172298 -0.56162583557729
H -1.14647463565080 -3.54106671132076 -1.20486243429806
H 4.16078429524162 -0.83558897861468 -2.37772175585155
H 6.49938039969673 -0.32888939775717 -1.81117029702901
H 7.12633719502096 0.63016477945384 0.39069985650677
H 5.35352318774586 1.16654855105174 2.06332533499115
H 1.16718453943220 -0.08293864164517 3.64657662378459
H 1.09446809508044 0.97439699010022 5.88932078248175
H 2.39721131796399 3.05483146790777 6.32895497917602
H 3.81748256676266 4.04410011149743 4.53646447580205
H 3.91098559590663 2.98381560080438 2.29886544893769
H 0.65786482113118 3.17934226706700 1.19857767295907
H -0.10830105101866 4.74635601339156 2.96794879002704
H -1.66297923916413 3.95007388265791 4.75101970494552
H -2.45678390816462 1.58525337521629 4.73751249601900
H -1.68199538753860 0.02206940149861 2.98018723843524
H -2.44429753805615 2.19376543133445 -0.74292893558787
H -4.89508675674909 2.43220912941441 -0.42972596915108
H -6.19742090668636 0.62111186349482 0.68457925176408
H -5.02871755846737 -1.43339247968376 1.48675976310241
H -2.58185789122595 -1.68294226590509 1.14541543469178

78
TS-anti-V 1 1 NImag=1 CorrGibbs=0.548647 ZeroPointC=0.617795 dipole=[-4.2419741,-0.1479603,-2.3452708] polarizability=[845.2569831,-15.8307052,965.462997,42.4430885,16.9097593,580.2642658] E_bp86_TZVPD_GAS=-1934.917987814 E_b3lyp_TZVPD_GAS=-1933.638479965 E_wb97_TZVPD_GAS=-1933.937177919 G_solv_hfip=-0.09258413203486786 E_b3lyp_d3bj_tzvp_smd=-1934.8807628 E_b3lyp_d3bj_def2SVP_smd_hfip=-1932.80750276 LowestF=-530.7094
O -2.40895482800661 1.71289530943297 2.46101057992726
C -1.11504129761490 2.09322530760535 2.02620190705561
C -1.10813621257296 3.49338712353217 1.40452692313787
C 0.04027665363706 3.98087413220315 0.76462960956727
C 0.03611391255724 5.24398003447159 0.17239057351993
C -1.11614282612775 6.03793532516934 0.21636070602979
C -2.25431149492660 5.56376873997271 0.87144116948588
C -2.24859194992710 4.29897210990861 1.46837531997852
C -0.19682235486916 2.02647460274240 3.23612965780118
C 0.86217802238782 1.11896223240555 3.02753886965736
C 1.73046138839496 0.83744312870495 4.08994997613928
C 1.55631645149009 1.50155340862659 5.30958154420909
C 0.52373993904650 2.42943446701330 5.48622941132917
C -0.37373477797667 2.68841983328339 4.44238537161657
C 0.72070742712530 0.54632643424348 1.66960026536516
C -0.47606123993006 1.04814820145921 1.10282933370449
C -0.98792063970268 0.60775601141081 -0.11489454931990
C -2.27824440855954 1.14536684035430 -0.64031551996958
C -2.34348672207678 2.39003822997233 -1.28413787645106
C -3.56510448003750 2.88088405032449 -1.74780594166396
C -4.73447687943666 2.13442031468481 -1.57579178557842
C -4.67722285783205 0.89006526387906 -0.94182495688510
C -3.45501107001876 0.39567149419670 -0.47847291003929
C -0.31089133961488 -0.43187088922228 -0.80726356150032
C -0.83554181287510 -0.88588086815039 -2.12817831215706
C -0.82752922865742 0.02816439354977 -3.20297094278473
C -1.30494536366872 -0.34673873178831 -4.45538048012197
C -1.79657546475890 -1.64144971495125 -4.65628927996046
C -1.84465738214519 -2.54406284108341 -3.58699953590201
C -1.37747898089908 -2.16902711572978 -2.32857360363854
C 0.87469754888129 -0.92124916209289 -0.25900321929162
C 1.46119151934683 -0.38219281018811 0.91930841562532
C 2.81303568360588 -0.94339999780589 1.06098895132046
C 3.00896408759884 -1.93927376296980 0.06889778891421
C 4.17955720801615 -2.69238184283253 -0.03132350006013
C 5.21151535077678 -2.42076392032576 0.86849201488995
C 5.06181992719225 -1.39988805771013 1.81624706489247
C 3.87472852329992 -0.66322087068264 1.92458171363898
C 1.75778334305826 -2.03682088980485 -0.82274793456094
C 1.16131277264910 -3.43130459152293 -0.82329440499950
C 0.28237087351822 -3.78913148832869 0.20944878119667
C -0.26608521064719 -5.06991894248692 0.25200431916042
C 0.05699660255807 -6.00742799220096 -0.73509339616414
C 0.94827730550244 -5.65997768836294 -1.75133303227425
C 1.50884112791972 -4.37965213832834 -1.79167974211532
N 2.48783996437541 -1.70318222814868 -2.02350685143792
N 1.46322879371112 -1.72609541885092 -3.38809472108046
N 1.35726880248331 -2.12185974386522 -4.41789653746732
H -2.99281365589790 1.61255659230084 1.69095580098669
H 0.94174618869867 3.36476146064902 0.71615213884394
H 0.93500212425522 5.61040574975384 -0.33093155193795
H -1.12232698568297 7.02441638161230 -0.25488457042496
H -3.15717434478074 6.17889134098893 0.91727460110622
H -3.14169702561667 3.92669764333410 1.97268347378430
H 2.50827142035682 0.08209280289062 4.00513154340346
H 2.23348673248384 1.28029178510651 6.13860843214051
H 0.40576272402173 2.93743427377898 6.44681922539602
H -1.20529051301042 3.38547202403693 4.57355391206302
H -1.43457061374150 2.97773006032746 -1.41811554830097
H -3.60272879757653 3.85454102759437 -2.24301362427107
H -5.69088012215141 2.52231428516194 -1.93633762345574
H -5.58732379003002 0.29979312700553 -0.80653686351606
H -3.40857948063585 -0.57701984655763 0.01762821971797
H -0.41859423611879 1.02847066410043 -3.05062418240446
H -1.27812412097176 0.36532275024487 -5.28376665289864
H -2.15651265266380 -1.94338113479836 -5.64311838146012
H -2.24982990161335 -3.54789634511318 -3.73565537002994
H -1.43659292424863 -2.86988343730571 -1.49709945345504
H 4.28246954733823 -3.46562728722526 -0.79453241147536
H 6.14135402868383 -2.99146111919648 0.82051705573667
H 5.88847272362403 -1.17074461476505 2.49334449169094
H 3.80792842328988 0.13397175331983 2.66119347556272
H 0.01344492148917 -3.06039781938229 0.97652459030137
H -0.95578626566262 -5.33585603006662 1.05715197931908
H -0.38150719553376 -7.00814551501506 -0.70687462573103
H 1.21670654451318 -6.38822411343854 -2.52073805024639
H 2.21925559621015 -4.13889676157118 -2.58288564690627
H 2.67847730891355 -0.68712540312206 -2.03261356584137

76
V 1 1 NImag=0 CorrGibbs=0.546784 ZeroPointC=0.612852 dipole=[4.2366619,2.7190065,-0.801347] polarizability=[793.6501041,-26.0781726,881.3314855,-57.6481998,-69.5677458,559.4809861] E_bp86_TZVPD_GAS=-1825.481311688 E_b3lyp_TZVPD_GAS=-1824.277366946 E_wb97_TZVPD_GAS=-1824.571159766 G_solv_hfip=-0.09282607260512957 E_b3lyp_d3bj_tzvp_smd=-1825.4702502 E_b3lyp_d3bj_def2SVP_smd_hfip=-1823.52852914 LowestF=22.0436
O -0.50226013119080 0.23272550221542 -3.66528841612966
C -0.10308948760400 -0.80194678958707 -2.78475362193229
C -1.08749360057701 -1.97584663091962 -2.78380372741593
C -2.19381617856926 -1.97785987225612 -3.63837157554296
C -3.12418379897284 -3.02083176203401 -3.58552500411253
C -2.94952867482222 -4.07542381705519 -2.68779097530888
C -1.82833749240751 -4.09037358697012 -1.85012374148412
C -0.90310356279881 -3.04800140470000 -1.89859508834572
C 1.29918293327896 -1.20533625914755 -3.20710855318658
C 2.22509890819011 -1.00294110910119 -2.16517519999580
C 3.59193244410228 -1.15377267197012 -2.43458541030975
C 3.99365836614785 -1.55494669376932 -3.71334470192313
C 3.05614353510266 -1.79763176491093 -4.72435345371149
C 1.69148128523107 -1.60654727760077 -4.47623987083922
C 1.48165098513000 -0.51047540398125 -0.97675781102325
C 0.14479249651427 -0.29749593071427 -1.35435240492522
C -0.83016831891825 0.21864397236605 -0.48367208281737
C -2.26995927597362 0.18583305164574 -0.87514525339691
C -3.08751735739587 -0.85141670621730 -0.40341496616429
C -4.41132778339175 -0.95369647150332 -0.83081048835220
C -4.93616905894289 -0.01593938484849 -1.72622385727735
C -4.13574290863228 1.03530590906157 -2.17860912011699
C -2.80779772982561 1.13853751869330 -1.75258144785440
C -0.42264980567790 0.68750829577474 0.76957665315103
C -1.43954576977162 1.11070720350293 1.76770675995387
C -1.57666651087065 0.35812956726057 2.94547358227615
C -2.53369736915575 0.70479900929429 3.90030723741033
C -3.35782317784288 1.81326905709683 3.69257345320586
C -3.23132947742044 2.56302833188388 2.51799261784848
C -2.28793830560509 2.20752091132898 1.55470616578031
C 0.95622218426244 0.57076556592772 1.13461214580337
C 1.87074687368106 -0.21491897089539 0.35265423908622
C 3.06876956159245 -0.69906184100018 1.02042897363873
C 3.46112110373366 -0.09131636810453 2.23785171084444
C 4.60139164100390 -0.50243838105564 2.94601233858518
C 5.33507745427487 -1.57830765177452 2.47511709704472
C 4.91706274746959 -2.25847798164330 1.31624790271854
C 3.80816095970093 -1.82901521750248 0.60415864171352
N 2.68486051087312 0.94517458682859 2.73044089717272
C 1.48992159135493 1.27929732183856 2.26237653761123
C 0.86191143617550 2.48542520082060 2.84270533827888
C 0.59631848043182 3.57459311382464 1.99741514011779
C 0.02899999108587 4.73494281719379 2.52040927761372
C -0.29435268222308 4.80614036476061 3.87856202761846
C -0.02780255585425 3.72244999232645 4.72073984104948
C 0.56405147542969 2.56693035913394 4.21072255927353
H -1.37458541679161 0.56558834597458 -3.39987581724633
H -2.34033481823175 -1.15654208148211 -4.34133036357037
H -3.99278735329182 -3.00228561374055 -4.24921830339552
H -3.68079857355397 -4.88672941389965 -2.64205151034136
H -1.67779759605451 -4.91679322185863 -1.15028629550564
H -0.03746155960401 -3.06369298970952 -1.23119665949302
H 4.34945137570953 -0.93925701019569 -1.68180965312102
H 5.05970710574899 -1.67295066035420 -3.92406537127085
H 3.39270363205512 -2.11522945758799 -5.71457133729225
H 0.94781802781597 -1.75117767283249 -5.26391283102980
H -2.67524258680955 -1.59124935446654 0.28539098657504
H -5.03474142443811 -1.77551066031166 -0.46953668835000
H -5.97142095056461 -0.10228896332634 -2.06621459232943
H -4.54358039147690 1.78098725746567 -2.86592038106122
H -2.18704478777875 1.97271321424206 -2.09171491419441
H -0.92724578199209 -0.50516142636492 3.11055982901299
H -2.63114942035149 0.10966821230064 4.81201773448992
H -4.10040251770142 2.09377087640136 4.44409998541414
H -3.87110939919695 3.43372736628089 2.35278952548374
H -2.18446624929353 2.80277458537310 0.64622281049073
H 4.87910396707163 0.01625175491708 3.86649007221748
H 6.21971164050587 -1.91457352737249 3.02042216968041
H 5.46412731907383 -3.14139092656264 0.97779624074813
H 3.47669372500673 -2.40061707099704 -0.25960713542228
H 3.06854908959613 1.49748803272664 3.50047989595286
H 0.83603429124999 3.50676192373754 0.93407960949197
H -0.17211805236973 5.58341186543860 1.86192321102059
H -0.75779620318855 5.70956496109037 4.28303023200276
H -0.28752102143444 3.77426358903181 5.78081343490385
H 0.75582797986258 1.71160625372346 4.86234585744221

76
anti-I 0 1 NImag=0 CorrGibbs=0.541167 ZeroPointC=0.608346 dipole=[0.2374395,-0.1640511,0.4786596] polarizability=[752.5097704,2.3167624,803.8284753,-47.1492804,-1.3769496,552.3011763] E_bp86_TZVPD_GAS=-1846.159635948 E_b3lyp_TZVPD_GAS=-1844.949009326 E_wb97_TZVPD_GAS=-1845.253131153 G_solv_hfip=-0.05148021079381188 E_b3lyp_d3bj_tzvp_smd=-1846.0932291 E_b3lyp_d3bj_def2SVP_smd_hfip=-1844.1211236 LowestF=19.6042
O 0.33273732252365 -0.57136584383295 -3.66036170644934
C 0.74755402786065 -1.35863555437923 -2.55319753060827
C 2.25682157053594 -1.56362403814079 -2.55194569868507
C 2.84754338851264 -0.99525624058075 -1.40378828742830
C 4.21643890196522 -1.19142174291990 -1.18437952364321
C 4.97409512504157 -1.87379507659222 -2.14491462247677
C 4.38392955650709 -2.37120065290335 -3.31185317200458
C 3.00465484264201 -2.22766155527514 -3.51362484937339
C -0.01127879901224 -2.67943731237403 -2.61204112547768
C -0.91475695360175 -2.95154524518768 -3.64339388000439
C -1.65512103365059 -4.13843497013673 -3.63554006732283
C -1.48947597740829 -5.06678428459403 -2.60602517922125
C -0.56846583836153 -4.80711529531246 -1.58437571705143
C 0.16603113036909 -3.62167493055088 -1.58888277569774
C 0.53640128146184 -0.64287886788901 -1.21195444181576
C 1.78016927280567 -0.35931287643034 -0.59363231481117
C 1.77310140434288 0.37566932511486 0.60424939839665
C 2.82463763454663 1.04510969921001 1.40560873132122
C 4.18969902162881 1.26684519616356 1.18511049887240
C 4.93154307591540 1.97870932430719 2.13607172382162
C 4.32927083483607 2.48086757755639 3.29460004570426
C 2.95312195981185 2.31076036377639 3.49728963500656
C 2.22154962072895 1.61534558380977 2.54577402583219
C 0.71842807905542 1.38489255937846 2.54919575046034
O 0.42929701100806 0.57968980673481 3.68267110164504
C -0.05792265072045 2.70311653021324 2.57069761386562
C 0.05510173063879 3.59533841697215 1.49418163542835
C -0.69149153641118 4.77259394670849 1.46932677895627
C -1.56314881120870 5.07486819881063 2.52251067286703
C -1.66276353407653 4.20009030647976 3.60555963249401
C -0.90661741547230 3.02308922854431 3.63393193903641
C 0.52322341451923 0.63318447973095 1.22244294984510
C -0.69755269468947 0.28728754017293 0.64328476867199
C -1.98206823436953 0.63291333074772 1.31685246321731
C -2.72872917287669 1.73721606617023 0.87809903595207
C -3.88621478557816 2.12199133091506 1.55480815335299
C -4.32302115850117 1.39924645482645 2.67027406410369
C -3.60476646729129 0.27903968556830 3.09404611276698
C -2.44198998530906 -0.10456060801494 2.41803964849229
C -0.69327409889516 -0.33321213643941 -0.63173459571964
C -1.97402965319676 -0.71475139687332 -1.29153409617641
C -2.44096492707071 -0.03260974999027 -2.42352033051621
C -3.61844967523511 -0.43795794489690 -3.05702083911159
C -4.33935008919295 -1.53095844169313 -2.56875216579552
C -3.88579080120375 -2.20864528083462 -1.43252746592231
C -2.71596096604465 -1.79721617665068 -0.79316038675661
H 0.83166202465909 0.26013243881708 -3.65586299039991
H 4.69868010534787 -0.85705184140843 -0.26839121842235
H 6.04309792004293 -2.02395474027928 -1.97203041080586
H 4.99460099578785 -2.89316855417493 -4.05318944307105
H 2.51734828642125 -2.64505725585407 -4.39887405821604
H -1.05669695530720 -2.22096651080071 -4.43961359098979
H -2.37184305238064 -4.33189178031458 -4.43840058986139
H -2.07419532363703 -5.99061961633084 -2.59759630787271
H -0.42730109351486 -5.52985709932006 -0.77607529582532
H 0.87173566795495 -3.41756269291263 -0.77917884962740
H 4.68062837632442 0.92734975376264 0.27578992854547
H 5.99749571230579 2.14842315385781 1.96234689846227
H 4.92740803460082 3.02670852521417 4.02887070688996
H 2.45664903149288 2.73266455029843 4.37510621638762

H -0.51114996765909 0.34226137517028 3.67971511463588
H 0.72032968326869 3.35736563435071 0.65995304782054
H -0.60052440045719 5.45521059141843 0.61994772213546
H -2.15867721638811 5.99140909011505 2.49771383298348
H -2.33646662727491 4.42872312515787 4.43589030981514
H -0.99219871612649 2.34346570962270 4.48309039566346
H -2.38198469114690 2.31163834389911 0.01693942430463
H -4.44672167729347 2.99674403132301 1.21482751716373
H -5.22656863094294 1.70619649568323 3.20364922557222
H -3.94882106025078 -0.30199381107689 3.95384375452089
H -1.89050379016936 -0.99285524592816 2.73917950048733
H -1.86858183250907 0.80845930531390 -2.81854828588358
H -3.97072112010346 0.09994330859893 -3.94146477492474
H -5.25460627078440 -1.85421994522909 -3.07204041191483
H -4.44303769118165 -3.06577646272771 -1.04526855114681
H -2.35597407544628 -2.33523017856310 0.08625239093669

78
IX 2 1 NImag=0 CorrGibbs=0.552217 ZeroPointC=0.62322 dipole=[-0.426373,0.9030768,-1.9323036] polarizability=[924.179347,12.4245808,831.8404195,-23.8921913,-49.5939125,560.36011] E_bp86_TZVPD_GAS=-1914.177131195 E_b3lyp_TZVPD_GAS=-1912.912253026 E_wb97_TZVPD_GAS=-1913.212782537 G_solv_hfip=-0.18582522626240064 E_b3lyp_d3bj_tzvp_smd=-1914.2807601 E_b3lyp_d3bj_def2SVP_smd_hfip=-1912.24259278 LowestF=17.5764
N -1.90308204075728 4.29791748020090 0.72717669448270
N -2.43301133089759 3.34765741929307 0.47844021555702
N -3.04155436031810 2.25999165739108 0.27459792692177
C -2.58012955539623 1.01176729848129 1.00495036030585
C -3.72301742746447 0.00333785684132 0.93548709980189
C -5.05172620982378 0.40188613907731 0.75521053749881
C -6.07147203144467 -0.55432268634122 0.72652042100089
C -5.77410341281648 -1.90735372746599 0.89355022461814
C -4.44846138243959 -2.30308138627686 1.09880696293810
C -3.42666535358100 -1.35482380026077 1.11891165706573
C -2.20540263338377 1.37181013899098 2.43266090470705
C -0.87255302970754 0.98979859011505 2.69379578724434
C -0.29996969948937 1.35455457455473 3.91762220906946
C -1.08075415132587 2.03157697504369 4.86161008710748
C -2.41847442021128 2.34871134539371 4.60593104489700
C -2.99331903166999 2.02308537892944 3.37115176343009
C -0.31532059547567 0.36626489195289 1.46617209050696
C -1.25756822409150 0.50553438771429 0.43562349219292
C -0.97215868432248 0.26622644525872 -0.91797623414538
C -1.98702934044266 0.53315680136911 -1.97764059696466
C -1.85799835781865 1.68526312462981 -2.77182430148222
C -2.81509957247600 1.98082775237849 -3.74402242582066
C -3.90447339382664 1.12532269331663 -3.93828675585327
C -4.03021765470799 -0.02800241039776 -3.15997771966007
C -3.07640127307750 -0.32609285054765 -2.18366134997855
C 0.33796072843597 -0.09589394584416 -1.25821973564570
C 0.74525141539688 -0.24978841624893 -2.68151950830477
C 1.67202133143490 0.63481055479400 -3.25775092987796
C 2.03689785815940 0.49686527802792 -4.59977923495653
C 1.47834769482256 -0.52074370317299 -5.37658900996046
C 0.55406503102637 -1.40341396388602 -4.80824031253757
C 0.18859664799936 -1.27085319794074 -3.46847971971427
C 1.26201327192857 -0.33698822609055 -0.21342984926278
C 0.94408238777028 -0.21688844612825 1.16468207360008
C 1.89617277748777 -0.76284167125520 2.11826545868146
C 3.22254873491028 -1.08552862524093 1.68355770450037
C 4.15516271798876 -1.63455517383518 2.59933026635186
C 3.78467550393758 -1.88828371462247 3.90580961264926
C 2.46007229070475 -1.64764994525700 4.31358399925612
C 1.53315590151260 -1.10796124133300 3.43548558783865
C 3.53560155210495 -0.99091919155993 0.29023434578787
C 4.86235188936508 -1.24813502182997 -0.29052604535443
C 4.99029528402893 -2.10161661577463 -1.40159599500997
C 6.24306030021962 -2.31320295842136 -1.97504210825134
C 7.36973638267941 -1.66703229475478 -1.45765275535081
C 7.24435703329348 -0.80880658371401 -0.36075462648357
C 5.99849820850860 -0.60162017913190 0.22905430268807
N 2.55303897110216 -0.70681617319287 -0.55584541420927
H -3.50684896668263 2.20118294884365 -0.64571097317715
H -5.31374335759547 1.45416361523206 0.63472757940397
H -7.10444883742882 -0.23205667327238 0.57401943189739
H -6.57256896957917 -2.65306070367421 0.86844137707635
H -4.20507519343431 -3.35938660691135 1.23762153630338
H -2.39524422541198 -1.67745361412712 1.27038132227858
H 0.74497748869251 1.14754164990049 4.14261701407051
H -0.63045065374039 2.31809824645888 5.81512661370749
H -3.01207305468120 2.86690421789493 5.36256065417726
H -4.02906222585508 2.28848412744670 3.14712234342956
H -1.00811157317689 2.35399230747379 -2.61643098844650
H -2.70830785685580 2.88181732334382 -4.35356124613078
H -4.65345989087660 1.35687635813053 -4.70002851141127
H -4.87449979579287 -0.70484451616880 -3.31307765965932
H -3.17506616213781 -1.23179246480811 -1.58491996636731
H 2.09352671201999 1.44730772128331 -2.65952730838726
H 2.75432021071326 1.19395513126199 -5.04008215617766
H 1.76159459498676 -0.62635861876512 -6.42699122073991
H 0.11475728745821 -2.20136247796073 -5.41235636367107
H -0.53223841128129 -1.96101095454779 -3.02514427136114
H 5.15458034772727 -1.90241960941724 2.25903248751725
H 4.50433891933679 -2.31988131060269 4.60444620180859
H 2.14736913023735 -1.91172871859159 5.32677983674902
H 0.50097031790197 -0.99748185018171 3.75898858798337
H 4.11610835739388 -2.62341521277606 -1.79801445485677
H 6.34020260108178 -2.98994481134456 -2.82725503079657
H 8.34998767882858 -1.83230305333358 -1.91174664679465
H 8.12228928233202 -0.29367104849234 0.03621044886906
H 5.89853715197954 0.08525918176246 1.07154786431345
H 2.77424336759824 -0.69506476712547 -1.55559513561436

76
IV 1 1 NImag=0 CorrGibbs=0.545934 ZeroPointC=0.612787 dipole=[4.5203604,-1.1034042,0.317499] polarizability=[907.2138203,-34.1596656,850.7141294,43.5184077,5.8040154,505.331613] E_bp86_TZVPD_GAS=-1825.485771868 E_b3lyp_TZVPD_GAS=-1824.285179017 E_wb97_TZVPD_GAS=-1824.579519636 G_solv_hfip=-0.08965498104039461 E_b3lyp_d3bj_tzvp_smd=-1825.4738647 E_b3lyp_d3bj_def2SVP_smd_hfip=-1823.5313813 LowestF=17.5996
O -3.16615505958010 2.09835730643266 0.94878636857011
C -2.75478241438086 1.49266667259417 -0.26766623338029
C -3.76691948621978 0.46179453127016 -0.76974670795695
C -4.94273194414701 0.20710306540879 -0.05861520333311
C -5.82090645125804 -0.79460831526811 -0.48322665886189
C -5.53065591877992 -1.54857474367241 -1.62202767163681
C -4.36089927408431 -1.28665502431720 -2.34490096144368
C -3.48608898055155 -0.28529337126514 -1.92258533709013
C -2.40537239461845 2.56194791201774 -1.29458192269784
C -1.02369127376185 2.56111552920570 -1.57558373050265
C -0.48370268526331 3.61666499742735 -2.32247105369916
C -1.33886327499299 4.59766581450628 -2.83660837611321
C -2.71908513522539 4.54111227469348 -2.61443283943454
C -3.26024709655376 3.51995654122246 -1.82334199213612
C -0.40968044324432 1.41166230159346 -0.86225863344272
C -1.38954097397820 0.84253013867681 -0.03128332640393
C -1.12838638642890 -0.21009975123900 0.85958957336814
C -2.23198907941235 -0.90360907011019 1.58358485412855
C -2.65353824466519 -2.15890901099463 1.11990023288943
C -3.72109731885639 -2.81544330769358 1.73336567377680
C -4.36454466899652 -2.23194143044214 2.82893102013511
C -3.93268577662973 -0.99212148932110 3.30859630690287
C -2.87294120182781 -0.32712274940749 2.68752355607019
C 0.19665571959391 -0.64389856878779 1.00379824458030
C 0.56285721544791 -1.70569727745841 1.97910357089461
C 1.07064584914834 -2.93673582479644 1.52919442539686
C 1.40348321500784 -3.94047748498037 2.44198812246101
C 1.23230139319810 -3.72559852781052 3.81199240283148
C 0.72744128463059 -2.50338296565613 4.26728545874837
C 0.39340620419167 -1.49892485386064 3.35811720992950
C 1.18499485801029 -0.06905947846647 0.17190235544842
C 0.90024271304364 0.86283858641359 -0.86174406322959
C 1.95166612142458 1.08555249291259 -1.84389720614434
C 3.29591023440445 0.68974489353229 -1.54261719202578
C 4.32268618893769 0.87214956117854 -2.50323945073780
C 4.03363377875745 1.40639039494638 -3.74382976099668
C 2.70345346773869 1.72739669058529 -4.06974904242268
C 1.68530972339367 1.56090258924696 -3.14448472007258
C 3.55236478946614 -0.01673142987645 -0.32330487412343
C 4.88625701160860 -0.39492744197311 0.16905937666810
C 5.11497251199535 -1.69588143033264 0.65532599081163
C 6.36799205713625 -2.04127039881080 1.15860049070564
C 7.39541286185979 -1.09351347579401 1.19525765843958
C 7.16876124012618 0.20380131812132 0.72568668891233
C 5.92270292058270 0.55545246910448 0.20888596082369
N 2.51031403230902 -0.40876158908031 0.39718358027110
H -3.86325356050224 2.74864970595394 0.76809889279690
H -5.15707389019833 0.77427088275612 0.84756072170280
H -6.73104504978806 -0.99249284378659 0.08932097916905
H -6.21246499768186 -2.33856907725295 -1.94813480113047
H -4.12781569345856 -1.86948869946163 -3.24019476040009
H -2.56794522922472 -0.09345537130586 -2.48475841412837
H 0.58807479432513 3.70539345864937 -2.49392910091868
H -0.91713523170675 5.41928272069731 -3.42122297578100
H -3.37268819100456 5.30574486624664 -3.04177002524821
H -4.33302052224711 3.48644657785804 -1.61375551665689
H -2.15590731514842 -2.60913113135663 0.25789438077718
H -4.05411157041443 -3.78417403911136 1.35193504522394
H -5.20189525753000 -2.74455128542441 3.31000199403294
H -4.42858973856325 -0.53495889233366 4.16909093917123
H -2.55115438021326 0.65114736812862 3.04730115752431
H 1.18662486497830 -3.11355163036594 0.45663337886895
H 1.79042560893501 -4.89629630071490 2.07946425295117
H 1.49140579162432 -4.51138058580551 4.52637077594656
H 0.59318336815923 -2.33095102648144 5.33828207776750
H 0.00279942176382 -0.54383710101666 3.71505422554300
H 5.33697058358719 0.54356533597419 -2.28015798581980
H 4.82692465773036 1.53319392990304 -4.48356983871687
H 2.46168874744109 2.08791152381184 -5.07265430813174
H 0.65975021305270 1.75323518930921 -3.44877529870071
H 4.32269866702642 -2.44778024309253 0.61443799485487
H 6.54414427695657 -3.05757919314340 1.51896114304406
H 8.37624531572372 -1.36669298514163 1.59258523176698
H 7.96637533395372 0.94947598102204 0.76622657199553
H 5.74252981385796 1.57528790930638 -0.13555827988061
H 2.69249580408052 -0.97207048565893 1.23173223569790

72
VI 2 1 NImag=0 CorrGibbs=0.514263 ZeroPointC=0.578074 dipole=[-0.0057986,3.6317966,0.0005619] polarizability=[1323.130631,0.0793378,1264.1859913,65.5675719,0.3279596,433.9374546] E_bp86_TZVPD_GAS=-1693.849517346 E_b3lyp_TZVPD_GAS=-1692.720186244 E_wb97_TZVPD_GAS=-1692.973826994 G_solv_hfip=-0.18402675142394362 E_b3lyp_d3bj_tzvp_smd=-1693.9740317 E_b3lyp_d3bj_def2SVP_smd_hfip=-1692.18346104 LowestF=14.7662
C -0.60850224927291 -0.52784715971287 0.50252509303542
C -1.89238607435598 -0.98942241994138 1.07223935360577
C -2.10762113102792 -0.89118668827186 2.45701338081837
C -3.29286293018703 -1.36319812650310 3.01737369867668
C -4.27207654738217 -1.93898799300702 2.20172556879798
C -4.06841030187069 -2.02732372613261 0.82090668276531
C -2.89136632681415 -1.54412183509605 0.25391864055347
C -0.60836666555045 0.52776896774889 -0.50269638759639
C -1.89217893712299 0.98983885455823 -1.07217523527790
C -2.89091439639788 1.54435000045360 -0.25344406295366
C -4.06787039907667 2.02821353659089 -0.82004680465745
C -4.27167824093147 1.94071097829146 -2.20089783254642
C -3.29271669393826 1.36508348488066 -3.01696512785114
C -2.10757079791289 0.89237964481941 -2.45696971330337
C 0.62007333136254 1.02996844207086 -0.93246609632937
C 0.90728208546345 2.11342618304573 -1.86939589775116
C -0.00947533832444 3.09064443070050 -2.41559474014323
C -0.91207754215736 3.77288338318996 -1.57007762653643
C -1.79554811282494 4.70123577804024 -2.10311230910337
C -1.81621192294113 4.94036121767411 -3.48416798158608
C -0.92658601338019 4.26990543664488 -4.32896764311766
C -0.00961663149274 3.36518634027188 -3.80110103881633
C 2.31205131012547 2.09635749206958 -2.13283715831615
C 2.93844157444828 1.08319836351370 -1.33216330819779
C 4.29009160616630 0.82019504133096 -1.50340307372136
C 5.02135813604706 1.60976081522108 -2.40817602302305
C 4.42785630163608 2.65978384354192 -3.12727802441290
C 3.06765617807809 2.91698076950027 -2.99418186771434
C 1.89481411036021 0.46408153653182 -0.50322671195047
C 1.89470531592687 -0.46483422256116 0.50289382552660
C 0.61983540107076 -1.03028568913232 0.93234184961356
C 0.90682135642159 -2.11392751732443 1.86913035503855
C -0.01028531657432 -3.09077378959775 2.41556558255226
C -0.01058498917182 -3.36483870393381 3.80117262496052
C -0.92808381586430 -4.26880186214971 4.32941669509163
C -1.81816592513759 -4.93900555381284 3.48492078068951
C -1.79728889183797 -4.70048889037466 2.10377285669520
C -0.91321213891334 -3.77293554901531 1.57033267889740
C 2.31166332435976 -2.09741473004278 2.13228733190812
C 2.93823814926232 -1.08430642442387 1.33168785156207
C 4.28995864667803 -0.82163184726148 1.50281067352410
C 5.02117664641734 -1.61161466464460 2.40726142260210
C 4.42748624650642 -2.66157766915880 3.12630114287666
C 3.06717384131593 -2.91833104367398 2.99345029575769
H -1.34078546322483 -0.44290455959764 3.09187611652008
H -3.45233795559611 -1.28392002113136 4.09530544366083
H -5.19749497430021 -2.31768152178763 2.64291375837589
H -4.83080595665075 -2.47898232704929 0.18153428444723
H -2.72782644615530 -1.63107235982595 -0.82113332618543
H -2.72724292866503 1.63059805378279 0.82163979619770
H -4.83007358248072 2.47974540433998 -0.18035285491222
H -5.19700368988180 2.31999306326266 -2.64177322013417
H -3.45228214203355 1.28652232016276 -4.09493522994130
H -1.34086571445077 0.44429524033844 -3.09215591753506
H -0.88931457188564 3.58755243191588 -0.49605933782362
H -2.48061214619512 5.23796683236431 -1.44317813208373
H -2.53220812726183 5.65252638779243 -3.90180150006707
H -0.95126684878904 4.44919867418823 -5.40629990037394
H 0.65713464545464 2.81158186739416 -4.46405708054501
H 4.79045184929741 0.01048225376490 -0.97679823522061
H 6.08651360359373 1.40580812424599 -2.54256484433868
H 5.03713864774828 3.27123247213108 -3.79561191214628
H 2.59988758106960 3.73403356014825 -3.54405815583437
H 0.65654864909064 -2.81148267657508 4.46393510542421
H -0.95280176071345 -4.44766336572155 5.40681996023829
H -2.53462207775556 -5.65052479365043 3.90286679861509
H -2.48265317787636 -5.23709127366244 1.44404555201634
H -0.89039813615105 -3.58832186055321 0.49620388500440
H 4.79035323248006 -0.01193081249367 0.97626076561995
H 6.08642546812565 -1.40803720247904 2.54146057430324
H 5.03670239993531 -3.27333873883822 3.79440716882445
H 2.59928823616233 -3.73533578197593 3.54328811403595

78
TS-X 2 1 NImag=1 CorrGibbs=0.552284 ZeroPointC=0.621357 dipole=[0.9520456,1.50296,-1.4023409] polarizability=[1100.6855062,19.3100221,888.1083667,52.7719779,-29.1253773,563.0765188] E_bp86_TZVPD_GAS=-1914.158842601 E_b3lyp_TZVPD_GAS=-1912.88980297 E_wb97_TZVPD_GAS=-1913.181872735 G_solv_hfip=-0.18414880535805203 E_b3lyp_d3bj_tzvp_smd=-1914.2556073 E_b3lyp_d3bj_def2SVP_smd_hfip=-1912.21143535 LowestF=-621.3567
N -1.07969921700404 5.78941717515405 -0.27318910050673
N -1.32584067363687 4.76161454869584 -0.59974364266796
N -0.90123418220631 3.15385789696157 -0.63254461334220
C -1.53270044033152 2.42450659486722 0.46006426045390
C -3.03144654604171 2.24475651026866 0.44738405747740
C -3.58067386699350 1.37234541823355 1.39936204973805
C -4.95163212442445 1.12962301781480 1.41805344888035
C -5.78921348209366 1.76107809546470 0.49047857033704
C -5.24695148435693 2.64002955654618 -0.44682522448133
C -3.87004423688730 2.88258554726967 -0.47152802185637
C -0.91306489524768 2.92367369327156 1.75237491923993
C 0.22606339295785 2.15955331766152 2.08286501003206
C 1.08603817045442 2.62785391635900 3.08536922365563
C 0.74066233702871 3.78528061196929 3.78944448239995
C -0.44418233362194 4.47535423109266 3.51138113909425
C -1.27973427701180 4.05327377915171 2.46743583106174
C 0.29696901977236 1.00684313496516 1.16636592410484
C -0.68474169855690 1.16730812799766 0.15841890539282
C -0.85776427758746 0.27860030154040 -0.92517713914616
C -2.00926697882837 0.40320480788819 -1.86454516261499
C -1.94877239550473 1.20770038413776 -3.01092061729248
C -3.07228066992299 1.34351144900290 -3.83279525277224
C -4.25335704430374 0.66762793392599 -3.52133589266787
C -4.30692466309931 -0.15954958594618 -2.39455150106913
C -3.18956709744094 -0.29696572406338 -1.57204522560211
C 0.05149486182783 -0.77263932948877 -1.04905594354399
C -0.03710461061057 -1.75042708823239 -2.16759971254053
C 0.26906845204774 -1.37024264630370 -3.48348971208297
C 0.17360112038990 -2.29837254313532 -4.52167746461848
C -0.23012245459892 -3.61039785016131 -4.25678864709056
C -0.53452584996028 -3.99615100514121 -2.94833344409645
C -0.43706726060321 -3.07191339197804 -1.90609725102072
C 1.04165274617131 -0.93313237691629 -0.04611258401223
C 1.09990549695505 -0.16093937367359 1.14421858463000
C 1.91700387113678 -0.69123096613548 2.22824736757298
C 2.90638441812651 -1.68560842560939 1.93477085712382
C 3.68518135198866 -2.24114262182902 2.98045073875909
C 3.48000668343149 -1.84674170732077 4.28910542499130
C 2.46175507076084 -0.92485750014040 4.58686859075630
C 1.68994192447141 -0.36616613361969 3.57838282918730
C 2.98904734628555 -2.20887306032516 0.60442641566367
C 4.02750383946352 -3.12336426714081 0.11978985559023
C 3.67625186920410 -4.22120143946722 -0.69020111918890
C 4.66713008268163 -5.06652135109713 -1.18301585229521
C 6.01199705876804 -4.81585167398134 -0.89088921723105
C 6.36707378875937 -3.71676535573227 -0.10272873060719
C 5.38292580201663 -2.87364635793368 0.40917659222319
N 2.02287929795241 -1.88466904535753 -0.25196197329594
H -1.35109842308509 2.92555553565544 -1.53764552271739
H -2.92559534077068 0.87146797071964 2.11677483373767
H -5.37100326597651 0.44215766726840 2.15681411237482
H -6.86415998614746 1.56408984803147 0.49999954435420
H -5.89195719802551 3.13564487305698 -1.17606609496040
H -3.47746414833167 3.55382998488430 -1.23706998485336
H 2.02213940097079 2.12136596350721 3.31499934804845
H 1.40610455887583 4.14884330923763 4.57599960965176
H -0.71386856842640 5.35582924346946 4.09913901170132
H -2.19291412537268 4.60216405599034 2.22682368529395
H -1.01796363490650 1.71780434421821 -3.27497402052729
H -3.01831222458488 1.97592653701030 -4.72237269018363
H -5.13234707440676 0.77848474126976 -4.16129139021809
H -5.22707613533343 -0.69582658277873 -2.15037810965180
H -3.23273775616737 -0.93427017755316 -0.68692240184909
H 0.58970780269769 -0.34730608879393 -3.68973030789195
H 0.41594135147687 -1.99477876052307 -5.54327768201652
H -0.30633270183783 -4.33415236960611 -5.07236038786033
H -0.85406971674247 -5.01977332915944 -2.73724565317759
H -0.68352775365873 -3.36935645456624 -0.88363423865089
H 4.41171770047105 -3.02199684462927 2.75966007107885
H 4.07392889610171 -2.28788898245003 5.09210590207529
H 2.25341891713269 -0.66249182556944 5.62689543533761
H 0.86550830090448 0.29004812669911 3.84660353239483
H 2.62642508804344 -4.43181952132337 -0.90948457597239
H 4.38856045426720 -5.92828956505985 -1.79386508773557
H 6.78743680419622 -5.47923285442460 -1.28207124049454
H 7.41860482018472 -3.51134076585635 0.11095280804784
H 5.66539992373145 -2.00099234192948 1.00052987996821
H 2.04367735895300 -2.31729168930912 -1.17916125182337

78
XIII 2 1 NImag=0 CorrGibbs=0.554503 ZeroPointC=0.623036 dipole=[-0.4181446,-2.6562841,1.8116216] polarizability=[813.275209,12.0089929,926.6579773,-1.0151907,-89.8529708,558.8094793] E_bp86_TZVPD_GAS=-1914.16768664 E_b3lyp_TZVPD_GAS=-1912.898689541 E_wb97_TZVPD_GAS=-1913.198011036 G_solv_hfip=-0.19008462012092509 E_b3lyp_d3bj_tzvp_smd=-1914.2765806 E_b3lyp_d3bj_def2SVP_smd_hfip=-1912.2398716 LowestF=18.2298
N 1.83918398631761 -1.90661015524235 -4.12247211169451
N 0.91400024935606 -1.36779123834473 -3.80621244948576
N -0.10870758330400 -0.70027505656592 -3.48576425463940
C 0.06667248200511 0.57065342340565 -2.67050346313589
C -1.11989362388468 1.47667981835851 -2.96036242597497
C -2.01359878542985 1.25348209450399 -4.01084623983470
C -3.09883648162919 2.11575553837865 -4.19831937731396
C -3.28539205418117 3.20866943985315 -3.35196074641394
C -2.37270665001438 3.44825114279888 -2.31855180670275
C -1.29538475340018 2.58731500548715 -2.12226223941410
C 1.40339700711370 1.21302164695319 -2.98969858468909
C 2.24501495957087 1.17475717148623 -1.85791620945004
C 3.45363140171925 1.87766781934367 -1.89558308304942
C 3.82381519760569 2.52959439817803 -3.07780917442658
C 3.00553737087319 2.50195532439767 -4.21207214363681
C 1.76567941933030 1.85315408048967 -4.16661164870131
C 1.53779842845112 0.45124474648371 -0.77136431740097
C 0.22946414448256 0.18410587475918 -1.19634790018662
C -0.75721060631083 -0.40059331657849 -0.38521951566697
C -2.06541902540106 -0.80006828332143 -0.98220192884145
C -2.19281868025482 -2.05016338859485 -1.61048928665199
C -3.38235365852725 -2.39360797918123 -2.26247068540536
C -4.45351598990884 -1.49698974626247 -2.28102420642734
C -4.33983889859374 -0.26408547364404 -1.63170575564253
C -3.15358756883519 0.08289785389674 -0.98426202863444
C -0.45414523914945 -0.61638288760324 0.96363215997959
C -1.39425277015263 -1.38374219296133 1.82320062148461
C -0.98544037936580 -2.64618444289047 2.28392982690845
C -1.83174356575265 -3.41643481999409 3.08271285173164
C -3.09116806651626 -2.92841039249564 3.43831847029310
C -3.50580634360127 -1.67381833230550 2.97882025461539
C -2.66911097190140 -0.90982454267234 2.16590351452877
C 0.85178595504217 -0.27306071926967 1.44148292997061
C 1.91526991848060 0.06853992371640 0.54073955576698
C 3.27851107401899 -0.08429816590081 1.01976917643885
C 3.49556087707396 -0.21043423255806 2.41344541404337
C 4.77807984619861 -0.37479451843552 2.95881634993597
C 5.86369071426955 -0.47701009190445 2.10554559175466
C 5.66735337392303 -0.44209754720368 0.71229049826876
C 4.40237626122822 -0.25074244068465 0.17953820588734
N 2.39508050254567 -0.16340550260160 3.25293598677362
C 1.13422588928197 -0.21262348244258 2.85079228193423
C 0.09967254606942 -0.03299251275630 3.88986164622598
C 0.00026223667475 -0.91509355641275 4.97554245740950
C -0.98218266765843 -0.70803765526391 5.94374369710414
C -1.84132094511380 0.39095072453415 5.84957108137856
C -1.72185076916319 1.28603682686541 4.78229440009952
C -0.76147830646340 1.07210505382178 3.79578274616397
H -0.97322729241716 -1.26590992986286 -3.37505608426445
H -1.89168365167487 0.40711284149721 -4.68775491718982
H -3.80109716105312 1.92402868895390 -5.01313851380059
H -4.13702239552334 3.87772772577710 -3.49838836491667
H -2.50546813729944 4.30703178804304 -1.65592270927406
H -0.59595011695351 2.77060409718301 -1.30373455311110
H 4.10103984077081 1.94589562426740 -1.02249074922462
H 4.76926960628254 3.07674317901538 -3.10802257089508
H 3.31929854145571 3.01305408116019 -5.12506814178892
H 1.08878453413297 1.87136009454298 -5.02394363666588
H -1.36214951454454 -2.76078721170442 -1.57555554645109
H -3.47084130440668 -3.36745588397245 -2.75061518973265
H -5.38047826570953 -1.76214328519355 -2.79577037302320
H -5.17662647708441 0.43849550501867 -1.63718248697395
H -3.06251409154208 1.05421776123140 -0.49732478123189
H 0.00398888752133 -3.02367671218794 2.01401522021020
H -1.50230175156979 -4.39769998227252 3.43383045199543
H -3.75133541073654 -3.52374795522819 4.07432441132390
H -4.48749506793499 -1.28444318879717 3.26018826286379
H -2.99034059752835 0.07555437249459 1.82761305766630
H 4.89651673473868 -0.44008798379513 4.04269246534040
H 6.86688742705244 -0.61356719683284 2.51528453589257
H 6.51798814562532 -0.57924073901247 0.04096364830179
H 4.27063219569136 -0.27983745922245 -0.89988504549709
H 2.57028451552461 -0.07305528170121 4.25719600103256
H 0.66290021631900 -1.78131177721105 5.03689618748121
H -1.07985614565597 -1.41171404623219 6.77382643770795
H -2.60855014406728 0.54926841984788 6.61165679315476
H -2.38790168195753 2.14932314832580 4.71167914152034
H -0.67210533508459 1.75875185690413 2.95137106693547

78
TS-anti-IV 1 1 NImag=1 CorrGibbs=0.547989 ZeroPointC=0.617435 dipole=[4.3369865,-0.3087339,-1.7396612] polarizability=[938.8031271,9.6822081,828.8828994,-95.4882538,-9.2353263,612.4237994] E_bp86_TZVPD_GAS=-1934.920835115 E_b3lyp_TZVPD_GAS=-1933.639223974 E_wb97_TZVPD_GAS=-1933.938498135 G_solv_hfip=-0.0905886721227048 E_b3lyp_d3bj_tzvp_smd=-1934.8816803 E_b3lyp_d3bj_def2SVP_smd_hfip=-1932.80825663 LowestF=-645.1881
O -1.79362476366146 3.00931331055494 1.77517358145596
C -0.64288589023044 2.95343186570809 0.94915323017996
C -0.79583910287919 3.80002112932207 -0.31489798156931

C 0.19390377541474 3.76133615944324 -1.30779529694943
C 0.03306473522717 4.47972116971807 -2.49234941084628
C -1.11880478413267 5.24821274392521 -2.69743124517971
C -2.09530800381091 5.30655527792108 -1.70126684855538
C -1.93240062384427 4.58973222206825 -0.51157329130130
C 0.51872050404263 3.38395116140546 1.82619065151590
C 1.44203049461920 2.33484326385935 2.01465911829607
C 2.48690622903277 2.51996623187395 2.92940241968522
C 2.61514835454669 3.74848919151167 3.58678559469502
C 1.71206685886933 4.79167122591690 3.35475490872709
C 0.63998164417097 4.60458815186079 2.47438475443226
C 1.00888512167304 1.17767986738221 1.19192626931852
C -0.24449619599875 1.51178613829449 0.60517283183777
C -0.99787150064112 0.63904804658723 -0.18149605260047
C -2.28403465204904 1.07771050492220 -0.79542167087767
C -2.30360564776882 1.51995312678343 -2.12643716331563
C -3.48349039838571 2.00802779529120 -2.68829931646837
C -4.65843499671792 2.04964206430303 -1.93073343521896
C -4.65255604732958 1.58572159931162 -0.61328879732907
C -3.47046083496882 1.09699988488072 -0.04760050131728
C -0.50205962199681 -0.66011946916108 -0.41241426974738
C -1.32417563386576 -1.65029875128083 -1.16353362623337
C -1.14781754937738 -1.85919113088458 -2.53862678657757
C -1.90281083019203 -2.82925504583255 -3.20599041751237
C -2.84498319577431 -3.58567102440397 -2.50670888975850
C -3.04625904131115 -3.35779877641943 -1.14106395384457
C -2.29625372518716 -2.39082129889974 -0.47315751324227
C 0.74576659992799 -0.98587951445238 0.14077993926065
C 1.55101923337128 -0.08498762266613 0.90638945757885
C 2.78932421439228 -0.79240221947676 1.28989825596208
C 2.67990345705716 -2.14520888960961 0.89888361954533
C 3.65860916359708 -3.08480067737702 1.18598648942651
C 4.82234821659760 -2.65472225854931 1.84152305335493
C 4.98585564191827 -1.30480093359873 2.16129696283199
C 3.98023160652996 -0.36866169885818 1.89045103298062
C 1.45121520704975 -2.35144282576993 0.03411861531907
C 0.61790442491166 -3.57975147286017 0.31161288073757
C 0.09756499298714 -3.72411890991321 1.60646328066599
C -0.71334879964447 -4.81246218815034 1.91918313311514
C -1.00781321233696 -5.77236192294775 0.94341227095261
C -0.48110093123790 -5.63783109756577 -0.34108863125449
C 0.32900808209109 -4.54342898484798 -0.65934860385687
N 1.84358579842607 -2.07976150035698 -1.35182118949074
N 2.88507823526233 -3.21547779618283 -1.89454491760575
N 3.93867633415189 -3.56030418694252 -1.89327662705602
H -2.56243362661047 2.67076013648091 1.28805913166622
H 1.09028015990661 3.15320584739828 -1.15994426600402
H 0.80705332267971 4.43582609734788 -3.26335536769278
H -1.25007476147438 5.80280552750117 -3.63036743153361
H -2.99531189906409 5.90933856359095 -1.84938275254497
H -2.70251065795523 4.63293082303596 0.26000154914466
H 3.17988881066199 1.72088880135894 3.17625321904786
H 3.43246009627239 3.88733315614479 4.29908065691177
H 1.83246635999565 5.74460371137760 3.87630501137493
H -0.09737358217552 5.39351934669093 2.30619649631344
H -1.38259900294025 1.50674812230506 -2.71202013189529
H -3.48353380442546 2.36666245277213 -3.72072167477766
H -5.58018751383508 2.43955345206599 -2.37025983471679
H -5.57051382989319 1.60247064706009 -0.02013760604326
H -3.46682261684680 0.72495006151795 0.98060581690309
H -0.43302595213212 -1.24748088336552 -3.09595634025958
H -1.75478523015002 -2.98797289105228 -4.27717561693448
H -3.43011801470173 -4.34838274772703 -3.02680862237787
H -3.78737765749981 -3.94258483993957 -0.59078899820551
H -2.44452036714982 -2.22058925484400 0.59475796313230
H 3.52742412875255 -4.13451791095647 0.91404309680058
H 5.60510987040583 -3.37574806960748 2.08841532357799
H 5.91100801156013 -0.96840298333343 2.63562195322130
H 4.15670247563638 0.67820211255613 2.11972349874884
H 0.31714643733168 -2.96926608698260 2.36566409483006
H -1.12184704531557 -4.91215251597395 2.92793410817643
H -1.64961179427006 -6.62277766913441 1.18716471781907
H -0.70829029106627 -6.37916039721536 -1.11079860874636
H 0.69828356970233 -4.45414541612620 -1.68228409848218
H 1.06484590893045 -2.27075194559847 -2.00514520121160

76
TS-VIII 2 1 NImag=1 CorrGibbs=0.534963 ZeroPointC=0.601985 dipole=[0.0481451,1.8675071,-1.1761813] polarizability=[1221.3285548,-26.8450347,1068.7273913,-88.4814537,28.5288987,553.415358] E_bp86_TZVPD_GAS=-1858.718715019 E_b3lyp_TZVPD_GAS=-1857.480360167 E_wb97_TZVPD_GAS=-1857.757075056 G_solv_hfip=-0.18383623637207436 E_b3lyp_d3bj_tzvp_smd=-1858.8180004 E_b3lyp_d3bj_def2SVP_smd_hfip=-1856.83490073 LowestF=-587.8098
N 3.99187245447183 -4.06386817937240 -1.81896079130139
N 2.98881168953284 -3.65332909965309 -1.60698066428640
N 1.98983243959981 -2.32090072606177 -1.30124979365594
C 1.52795441326330 -2.34191370709659 0.06915989294198
C 2.72130490080616 -2.11188013938117 0.97886258109993
C 2.91117931348497 -0.73891951977393 1.23720019944687
C 4.08847151550180 -0.32818155015317 1.87373785138088
C 5.00011826108241 -1.29490945178601 2.30802962794551
C 4.75366556195409 -2.65873052196682 2.11596632407840
C 3.60667608612396 -3.07988826232414 1.43039403096257
C 0.55886076273415 -3.41681216882375 0.49775137008706
C -0.00283687055465 -3.29689890377349 1.77916981446294
C -0.92802296379738 -4.23457739584995 2.22843341329717
C -1.29717522882421 -5.30596333503571 1.40539466221589
C -0.73225639564068 -5.43368365571975 0.13632383053798
C 0.19173662626674 -4.49037603932522 -0.32134868012249
C 0.93571143738237 -0.90416870238480 -0.04943378051698
C 1.76246117846909 0.00692586206383 0.70411472332055
C 1.28879967242201 1.29834205365821 0.86008348669880
C 1.69645243124267 2.45240701957519 1.69503908299052
C 2.62152507277029 2.61186028968757 2.71260285634835
C 2.75483688702101 3.87707073178523 3.32288973571570
C 1.99444093472694 4.97972021422331 2.91850127878652
C 1.03545591776301 4.83058672196431 1.91869413942274
C 0.85021936843650 3.55671221246265 1.34873233817272
C -0.10789587886328 3.13236655397034 0.37570810673968
C -1.06707309833458 3.99216335460053 -0.27101303198198
C -1.27394819681457 3.90900490104145 -1.66826555417012
C -2.22157290601985 4.71645642770350 -2.27958922902972
C -3.00016860626141 5.59286349823840 -1.51040614687991
C -2.80471880400326 5.68415392949335 -0.12840018079431
C -1.83078894205695 4.90823397217204 0.49061326791135
C 0.10284040414832 1.69988704201745 0.14463456438760
C -0.72967684770691 0.78632092084504 -0.52315271226600
C -2.09301029551927 1.13497485911463 -0.98787439460246
C -2.47658341854315 0.96558262695051 -2.32749357907133
C -3.75451642993942 1.33785251897492 -2.74212794752176
C -4.66576034273385 1.86728748197665 -1.82341614904336
C -4.29679014856672 2.01710400671969 -0.48311616593687
C -3.01844933264277 1.65011298648842 -0.06543054608698
C -0.27047226132294 -0.56536144311977 -0.65302767566393
C -1.12945246577843 -1.58768278453228 -1.30940376541007
C -0.85950168056958 -2.05651445477733 -2.60380727507943
C -1.65402554661430 -3.06024183733391 -3.16659315939283
C -2.72375881474185 -3.59121530851603 -2.44446454734309
C -3.01253969920365 -3.10492733250333 -1.16410510588665
C -2.22882601978933 -2.10011302775567 -0.60159974061224
H 1.23129205280063 -2.60299005676153 -1.94849209621994
H 4.32415535926495 0.72504322484516 2.00231761737543
H 5.91815150625055 -0.97612766968184 2.80722726529642
H 5.46627547721040 -3.39933183339551 2.48620390638916
H 3.42050226925313 -4.14135156322368 1.25296029781772
H 0.28070041199372 -2.45663548310351 2.41763585440314
H -1.36640499799924 -4.13110964390545 3.22399108322778
H -2.02860304179185 -6.03848610157340 1.75569661094068
H -1.01879859449768 -6.26317788090413 -0.51410293154429
H 0.58396051644014 -4.60206789020740 -1.33353583799875
H 3.23625911727549 1.79056870503664 3.07062879256020
H 3.49020538783096 3.99679393140389 4.12241503873622
H 2.15063764534723 5.95285078105397 3.38770541943404
H 0.44478611044581 5.68471543072534 1.58656710576108
H -0.66161843345681 3.23194680999767 -2.26414634130924
H -2.36601475889486 4.66023012154379 -3.36066583808894
H -3.76608467805068 6.20493280326268 -1.99313884507689
H -3.42292573963955 6.35703099957888 0.47015620932933
H -1.71590050462833 4.94144502499290 1.57464387668473
H -1.75993810347955 0.58056743607377 -3.05381086605388
H -4.03784927413505 1.22103401030007 -3.79112772944571

H -5.66605666798920 2.16124050634128 -2.15145115794047
H -5.00783269561335 2.42223051283823 0.24098170020762
H -2.72777019633934 1.76846816004300 0.98072951666800
H -0.05032872017611 -1.61173582459307 -3.19043183675602
H -1.43774443929072 -3.41962948885259 -4.17558580720169
H -3.34146964335611 -4.37974258456671 -2.88142092715605
H -3.85200944464679 -3.51583609640519 -0.59829500543761
H -2.45052930990005 -1.72437416801898 0.39887680086881

78
anti-III 1 1 NImag=0 CorrGibbs=0.551369 ZeroPointC=0.620015 dipole=[-5.0418564,-0.6597297,-3.297126] polarizability=[778.0347758,3.3216571,812.5585399,10.9325313,-19.1458312,576.8362506] E_bp86_TZVPD_GAS=-1934.926189922 E_b3lyp_TZVPD_GAS=-1933.649345697 E_wb97_TZVPD_GAS=-1933.958215838 G_solv_hfip=-0.10155937501169846 E_b3lyp_d3bj_tzvp_smd=-1934.9051378 E_b3lyp_d3bj_def2SVP_smd_hfip=-1932.83744083 LowestF=24.3248
O 1.73166543010067 -1.16031921228435 3.29401133815638
C 1.94034043223703 -0.07569949151064 2.40552112266999
C 1.74874120273654 1.27826658643413 3.09297586470890
C 1.82962089336784 2.45971641725520 2.34130527586452
C 1.59580595254570 3.69650732129294 2.94115508397329
C 1.27859424036309 3.76871743648504 4.30293408745054
C 1.21859052154007 2.59689871830699 5.05880648779245
C 1.46033873578043 1.35673783712852 4.45800586453706
C 3.34181469289615 -0.22737997748174 1.83635052479389
C 3.31196357161633 -0.42243876662631 0.44020737218074
C 4.52268137358742 -0.46490035033361 -0.26219601641919
C 5.72749035716312 -0.38789622268479 0.44636548509108
C 5.73917037865417 -0.25826203720617 1.83962807253682
C 4.53269312585076 -0.16214574374169 2.54501510375820
C 1.89729336498182 -0.44914203645012 0.00284862505021
C 1.08360813272328 -0.18006574797285 1.13268198006473
C -0.29345621333481 0.01408989616707 1.05552532726615
C -1.08525328172664 0.30870823015300 2.28306663981667
C -1.49601116629814 1.62057053401569 2.56259105098072
C -2.16072075870122 1.91231921829033 3.75367346985899
C -2.43534235078163 0.89515914507677 4.67391778084654
C -2.05621332639110 -0.41869471579757 4.38954407595540
C -1.38696377494335 -0.71224742506304 3.19707481099012
C -0.91701858707334 -0.02097635132807 -0.21799406218943
C -2.38480501100378 0.21308931414507 -0.33304527231515
C -2.89471522985599 1.44919615884987 -0.75089519275996
C -4.27395168423754 1.63984076634881 -0.87212364313912
C -5.15430095137670 0.59739250904912 -0.57350017627168
C -4.65151929353411 -0.63288069688146 -0.13750294125642
C -3.27561087099074 -0.82202341065837 -0.01023684658957
C -0.12567806584024 -0.33986132994845 -1.32108476344044
C 1.26333461846893 -0.62787292396164 -1.23585835964103
C 1.71176501040286 -1.13337344623768 -2.55371783944475
C 0.63275007418296 -1.05047445690685 -3.45925596505990
C 0.72174443174865 -1.50190404768997 -4.76811959540908
C 1.92791369502552 -2.07285834194990 -5.19415551003012
C 2.99311900400975 -2.20490289717156 -4.29808083443763
C 2.89396795839460 -1.74876315761298 -2.97724219794384
C -0.58997647741685 -0.46955541181369 -2.77067598673689
C -1.86991949394893 -1.26149378337196 -2.97851868910397
C -1.83101671104495 -2.62008951560266 -2.63111988934631
C -2.98132470639011 -3.40104074962514 -2.72342316152285
C -4.18108430709388 -2.83694209417310 -3.17043526045809
C -4.21779977724154 -1.48942961395780 -3.52841576052709
C -3.06680439822247 -0.70117399930324 -3.43315904175544
N -0.82450616730947 0.93943166483962 -3.33973934694120
N -0.17634816495857 1.91297972894695 -2.87253662967019
N 0.37613475678369 2.74921989731455 -2.37839817787904
H 0.82663477938670 -1.12135245891469 3.64286946260688
H 2.06248903748446 2.41030008552149 1.27426615556459
H 1.65423590621909 4.60993305451849 2.34288914536207
H 1.08385399056734 4.73718454801696 4.77128526890196
H 0.97769091903417 2.64228713878493 6.12430452946651
H 1.40823626796962 0.44504068447157 5.05496677668513
H 4.54882001757304 -0.52446494161410 -1.34805087828820
H 6.67218383178438 -0.42340157125011 -0.10245340555717
H 6.69087465321100 -0.20838271136987 2.37483780055620
H 4.52291482438705 -0.01945235538653 3.62864597905603
H -1.26597651689825 2.41927519213771 1.85514691055562
H -2.45805010661262 2.94172471576928 3.96961928759530
H -2.94998364076228 1.12689882039790 5.61007088402825
H -2.28109869466444 -1.22138040178033 5.09672732559556
H -1.10233416384438 -1.74294307789046 2.96679545461883
H -2.21228079158466 2.27334911659521 -0.97172853271575
H -4.66049996651351 2.60875583432327 -1.19900422294732
H -6.23282109306987 0.74433713576576 -0.67447314850047
H -5.33519256432360 -1.45179494534298 0.10029572068856
H -2.88309757344798 -1.78528823342563 0.32196363953800
H -0.13511880805049 -1.43379612659500 -5.44292998994056
H 2.02563857834758 -2.43967837409964 -6.21863232894891
H 3.91810937428111 -2.68615392498800 -4.62558605879773
H 3.72191656790727 -1.91836261727217 -2.29288449487409
H -0.90005947555928 -3.06343015019006 -2.27044844090236
H -2.94114181032708 -4.45621027289660 -2.44152950692063
H -5.08456379411595 -3.44836511886376 -3.23657749057325
H -5.15022860151256 -1.03565751738809 -3.87266311595885
H -3.13772643300698 0.35662201112513 -3.68715202266099
H -1.12684311124750 1.05921304679415 -4.31856492938586

74
XII 2 1 NImag=0 CorrGibbs=0.532392 ZeroPointC=0.596954 dipole=[-0.1395481,3.9605654,0.3466846] polarizability=[1036.0475028,18.1203898,1135.5194375,-102.5264658,23.6646949,505.7255653] E_bp86_TZVPD_GAS=-1749.273887656 E_b3lyp_TZVPD_GAS=-1748.110785354 E_wb97_TZVPD_GAS=-1748.37990617 G_solv_hfip=-0.18652408423686095 E_b3lyp_d3bj_tzvp_smd=-1749.3996544 E_b3lyp_d3bj_def2SVP_smd_hfip=-1747.54912684 LowestF=19.5802
C -0.64678603431200 -0.06720332519666 -0.67727434535132
C -1.87335772198890 0.18170183938561 -1.48134749585226
C -1.92743767442454 1.29171765956745 -2.33714928398646
C -3.06259062064712 1.52527369328509 -3.11362777147490
C -4.15517168959741 0.65670611876654 -3.03549795594547
C -4.11332053091906 -0.43870122177245 -2.16781204283144
C -2.98105831796399 -0.67311566520991 -1.38797169491826
C -0.70346348395997 0.04539697971154 0.73400515974479
C -2.01200979245058 0.17472705109075 1.42112684374651
C -2.40246417603529 -0.85463856428178 2.29351969162937
C -3.62797638613716 -0.79005881623857 2.95773895546260
C -4.46629938650458 0.31015315090910 2.76738626656020
C -4.08315158883080 1.33751247434386 1.89626241172503
C -2.87195235709698 1.26467020583889 1.21343421815428
C 0.48420729532427 -0.10635649845055 1.49794936515296
C 0.54272064088700 0.30351039730310 2.88480474766343
C -0.36938119409660 1.28777994191187 3.50272613032910
C -1.04267129771194 1.00277951747263 4.70008618371584
C -1.87117185453627 1.96827398522263 5.27141058787030
C -2.00552026798601 3.22374302564272 4.67133108677956
C -1.31263985674237 3.51583697441584 3.49281763582785
C -0.50312125217352 2.54926101200622 2.90038676232397
N 1.56684049191838 -0.09117986318941 3.62086961583954
C 2.60610453378698 -0.90073113416627 3.18150771300570
C 2.71546500694561 -1.14989822664279 1.79382115077593
C 3.72037496175780 -2.04881478546294 1.38145502104901
C 4.60872633607987 -2.59359682875896 2.29905466614915
C 4.52288253564612 -2.26672986814847 3.66294004336089
C 3.51313524473376 -1.42847863861153 4.11063475189985
C 1.70969005070364 -0.58278604422191 0.90576145449546
C 1.79100969379238 -0.51245585259000 -0.49042074289531
C 0.60901129555712 -0.34057702083127 -1.26251058245092
C 0.98270332550479 -0.41276212428962 -2.68836242035103
C 0.10002748305224 -0.49401804626192 -3.82042036390841
C 0.30892247971649 0.32756763030498 -4.95486980160600
C -0.58414866970369 0.27750407154919 -6.01885019807751
C -1.65586766878805 -0.62211491404574 -5.99424537993743
C -1.84447183917039 -1.47243746386318 -4.89529407191230
C -0.99062653490826 -1.39762235345169 -3.80588802952787
C 2.41212493058937 -0.43417388058676 -2.75714033786439
C 2.94331936464379 -0.49424098680752 -1.42924267043977
C 4.31130876258834 -0.41620927113399 -1.24530809727712
C 5.14803125464803 -0.34697295487147 -2.38304461219712
C 4.63556069822527 -0.38254358817209 -3.68231146840517
C 3.25540507605105 -0.43617610441596 -3.88318997259606
H -1.07244875917231 1.96883540202444 -2.39825623967157
H -3.09289800653549 2.38835178614652 -3.78325812936409
H -5.04202382604895 0.83618493963862 -3.64859950877762

H -4.96695191910724 -1.11783597099928 -2.10028614922091
H -2.94723870298970 -1.53457836209599 -0.71850713325463
H -1.74182995583413 -1.71191763984294 2.44454488660369
H -3.92431279652618 -1.59836501418693 3.63088834904649
H -5.42130036627898 0.36991216174979 3.29570978821616
H -4.73465801499004 2.20271893002666 1.75050208571901
H -2.57391823579030 2.06961817641888 0.54033301732110
H -0.95090761382168 0.01482733442420 5.15705111137789
H -2.41861908757987 1.73610534735705 6.18796428509499
H -2.65503797402343 3.97728348349497 5.12378538746704
H -1.41054326420026 4.49892862613284 3.02622795239030
H 0.03073867917364 2.76797679060225 1.97339340840639
H 1.61664777784358 0.25126628321289 4.58434676609270
H 3.77599348165729 -2.36083590277669 0.34069637754022
H 5.37266262944989 -3.29440247087828 1.95523193055242
H 5.23292049577450 -2.69184287911687 4.37556854495752
H 3.40050619454907 -1.18777726812789 5.16999314621931
H 1.11735409464825 1.05974152414129 -4.95524981178731
H -0.44964259592046 0.94723876610851 -6.87109448876062
H -2.35178401318320 -0.66085236254821 -6.83597700268260
H -2.67065848463648 -2.18630531259847 -4.88871627565127
H -1.12675681962187 -2.06083236585449 -2.95262347031348
H 4.76463699768575 -0.40060156162808 -0.25536791942679
H 6.22869710656890 -0.28416482992387 -2.23341832263177
H 5.31275082143350 -0.36575374392739 -4.53823670514531
H 2.84631407275680 -0.47887412611171 -4.89302223688551

76
TS-XII 2 1 NImag=1 CorrGibbs=0.535121 ZeroPointC=0.601783 dipole=[-0.230865,-2.5720119,1.3187199] polarizability=[1097.9950652,-0.9892368,1162.1840551,26.3850417,-37.2038958,505.0265969] E_bp86_TZVPD_GAS=-1858.719645 E_b3lyp_TZVPD_GAS=-1857.481649996 E_wb97_TZVPD_GAS=-1857.756593063 G_solv_hfip=-0.18591042019525658 E_b3lyp_d3bj_tzvp_smd=-1858.8195026 E_b3lyp_d3bj_def2SVP_smd_hfip=-1856.83748982 LowestF=-520.557
N -0.83351255157692 0.65988234282255 5.00585435413867
N -0.25982236255433 0.43301364088730 4.08623828910693
N 1.33075193004962 0.35089376665969 3.41065300783544
C 1.40989227387278 1.10370965030310 2.18382119433774
C 2.91569472797141 0.80782562190085 2.04095154564875
C 3.15716293561821 0.08213037292893 0.84681974218864
C 4.46122322301638 -0.34274793613379 0.58872805549961
C 5.48447545836145 -0.00115924020057 1.48314414326682
C 5.23444589436876 0.76856110645634 2.62622101602458
C 3.93206049200234 1.17107711667110 2.92615837846615
C 1.11212127275947 2.58238754527691 2.32014311857713
C 0.98097960233645 3.35134744551747 1.15465769049447
C 0.72231834834274 4.71808353080985 1.24317403422115
C 0.59588265290913 5.33001065428197 2.49498325901448
C 0.75028629371772 4.56973993139234 3.65542677919269
C 1.01788970829119 3.20026193926588 3.57207279264362
C 0.82626834672491 0.40463427705055 0.95078543333459
C 1.89243713268530 -0.08081003369814 0.11676672939244
C 1.56307841738320 -0.60842016531593 -1.12128643215886
C 2.35413607100449 -1.06212445782325 -2.28880771212124
C 3.67965548921241 -0.90543332447306 -2.65530698059312
C 4.10814816013125 -1.40418425468759 -3.90558633144600
C 3.22516537473110 -2.01565181615484 -4.79826923461198
C 1.86927592813259 -2.11012258736604 -4.47758491632270
C 1.44038680300717 -1.64429557410586 -3.22344277215685
C 0.09917928425879 -1.46164568935849 -2.74397849716609
C -1.07773412079439 -1.81189711673309 -3.50304711640722
C -2.12733228042441 -0.88214823658913 -3.68131293796377
C -3.26177843017880 -1.24363270334544 -4.39340397144906
C -3.38399268126550 -2.54228627865856 -4.90719722550920
C -2.34855405192012 -3.46813814919795 -4.74240233741742
C -1.18698355125812 -3.10281543972374 -4.06900828165322
C 0.17500543141754 -0.82069914469084 -1.43382346414258
C -0.85569207370857 -0.53444005781384 -0.51787809646641
C -2.25834026163121 -0.96715790764650 -0.72907507725237
C -3.30542778304783 -0.03484018593362 -0.77996624048732
C -4.61462170585318 -0.46410496828009 -0.99415879732950
C -4.89287667429003 -1.82647024805114 -1.14064895787133
C -3.85596890183638 -2.76148651756334 -1.06642245356759
C -2.54479041577565 -2.33509591363633 -0.85931331579710
C -0.51359568571786 0.15569858267950 0.69158889460733
C -1.58505311135227 0.46806464614117 1.68376604839503
C -2.26640752213946 -0.60226616479298 2.30103904322256
C -3.28361560824889 -0.35542088701210 3.21704364489711
C -3.63506823191405 0.96213585233721 3.53272604524545
C -2.98595517176320 2.02987855583455 2.90165102836452
C -1.97195341414820 1.78793401828549 1.97662595830070
H 1.30617485377517 -0.66039672604136 3.19454950926688
H 4.69483563403902 -0.95398398652579 -0.27885163343584
H 6.50167741318651 -0.34208073588781 1.27638842967442
H 6.05460403077254 1.03695420454694 3.29532121734855
H 3.70918476351351 1.74635454817871 3.82607113138306
H 1.06890396108359 2.88040183260657 0.17327676563632
H 0.61349493054290 5.30795530552053 0.32970688974424
H 0.38407139207669 6.39997781104332 2.56371119100694
H 0.66563574000586 5.04131253154238 4.63754449834244
H 1.15405588784889 2.63070698230882 4.49187079515813
H 4.39269652895956 -0.37064220521751 -2.03301882444608
H 5.15780972526385 -1.28381314872038 -4.18502458665945
H 3.58638548783302 -2.38141689217415 -5.76128504304454
H 1.15726333320547 -2.51130907135583 -5.20014493837614
H -2.02375963184252 0.12701996786690 -3.28474403714099
H -4.06310166383811 -0.51676651405989 -4.54260011144710
H -4.29257736323762 -2.83183189070908 -5.44101077795013
H -2.44979715554451 -4.48103015148885 -5.13861677048842
H -0.39254108971630 -3.83394660489985 -3.91054299935371
H -3.08936616426357 1.03044088793608 -0.68252241544991
H -5.42220719099559 0.26999203087717 -1.05130006832264
H -5.91966918080877 -2.16019622417014 -1.31055112593696
H -4.06873159698687 -3.82807116588633 -1.17246254335730
H -1.73345291453262 -3.06382484847428 -0.80253176720375
H -1.98138300311032 -1.62919238372350 2.06525842328974
H -3.79565973004069 -1.19142376167849 3.69919147095883
H -4.42527884634330 1.15771357697001 4.26185157366304
H -3.27291704462683 3.05869354925446 3.13144526764236
H -1.48683608757944 2.62195974089279 1.47156036926303

78
TS-syn-V 1 1 NImag=1 CorrGibbs=0.54949 ZeroPointC=0.617731 dipole=[4.7677045,-0.559432,-0.7053775] polarizability=[847.7479886,16.2943684,975.4264607,31.6544568,-49.0466204,571.6222816] E_bp86_TZVPD_GAS=-1934.918629648 E_b3lyp_TZVPD_GAS=-1933.638252396 E_wb97_TZVPD_GAS=-1933.936562612 G_solv_hfip=-0.0932470064888664 E_b3lyp_d3bj_tzvp_smd=-1934.8819891 E_b3lyp_d3bj_def2SVP_smd_hfip=-1932.80905466 LowestF=-527.9338
O -1.56166704916487 -3.60933814005847 1.14554282176174
C -1.24016992220580 -2.92721508882463 -0.05476453165803
C -2.47785095569512 -2.64834002676880 -0.91108725803873
C -2.35410376452417 -1.89908494464399 -2.09036142952730
C -3.48093238977796 -1.58118600459644 -2.84742243107950
C -4.74853541250482 -2.00896108271845 -2.43485510286927
C -4.87288626085397 -2.77303173937203 -1.27346387217291
C -3.74090177476506 -3.09933566817636 -0.51851249901292
C -0.22215616178263 -3.78257988786195 -0.79121465410819
C 1.00987975019207 -3.11105274309195 -0.93068165329112
C 2.02303041870470 -3.70921896817388 -1.69046763168228
C 1.81439198550601 -4.98655578177737 -2.22346185521189
C 0.60857729825301 -5.66652579336769 -2.01913264220486
C -0.43035707482288 -5.05325068741465 -1.30711131496053
C 0.89614045727503 -1.78995308782472 -0.27117526865630
C -0.43333546112981 -1.64167440334289 0.19084685119546
C -0.92680766199107 -0.45301653340246 0.72601290479511
C -2.35358195935823 -0.35235188829666 1.14693669653732
C -2.80729649661063 -0.96128107731696 2.32681352017578
C -4.16373109035839 -0.92825141443702 2.66375883061928
C -5.07664015019848 -0.28060347474711 1.82830841800850
C -4.62614392171953 0.35105143515670 0.66442269567903
C -3.27248764200735 0.32290158323237 0.32933268709460
C -0.07905101963054 0.68542799739630 0.76698226364626
C -0.59705219850275 1.95274305830598 1.35881818678297
C -0.93800965744792 1.96559811773890 2.72779221667900
C -1.42425892644469 3.12478900810102 3.32512593450210
C -1.57945683226530 4.29002868254016 2.56543497206746
C -1.28313432175263 4.27654445080625 1.19718398087669
C -0.80370769148636 3.11551414473782 0.59356498172643
C 1.23729671446085 0.53854065313153 0.32850853201031
C 1.77541310396273 -0.70732262690371 -0.09742330254094

C 3.23353711082601 -0.56067171692172 -0.22545386367510
C 3.58335847135431 0.80062158945703 -0.02822873573311
C 4.89375421445245 1.27095945387823 -0.12590262909804
C 5.90313154139010 0.34516875383622 -0.39460972395002
C 5.58723260398822 -1.01277236246752 -0.53187408598346
C 4.26611527732747 -1.47312059214961 -0.45529112630662
C 2.31834388710626 1.62098922787257 0.28443786242746
C 2.10381388956972 2.74362082234286 -0.71243494161723
C 2.59008480432197 4.03537370484992 -0.48270811229285
C 2.38159607605475 5.04113429896192 -1.43156907342793
C 1.70859341067201 4.75946100679778 -2.62136894063984
C 1.25176760922361 3.45990333920095 -2.86661208831044
C 1.44773924623573 2.45715357812160 -1.91848927280654
N 2.81596428388901 2.05284958715172 1.56955718230685
N 1.70191790735784 3.10016964657115 2.33492377302880
N 1.60045186582918 4.07930503369717 2.84399937195594
H -2.16806759422921 -3.06475438986258 1.67165543457674
H -1.36974854898992 -1.54666605325565 -2.40965999299556
H -3.37237529336808 -0.98910895229932 -3.76021945526657
H -5.63413486770669 -1.74907913363444 -3.02078867515504
H -5.85731128390234 -3.11764825192701 -0.94546983561704
H -3.84724085678468 -3.69630215471582 0.38850076046738
H 2.95539007233532 -3.19172342670153 -1.90454356817514
H 2.60647204184350 -5.45350626913456 -2.81456307947600
H 0.46892190298109 -6.66697920037395 -2.43662787973377
H -1.39386350024581 -5.55184438578453 -1.17418514711615
H -2.09044351850720 -1.45341921720668 2.99032237969303
H -4.50586121103193 -1.41044051001164 3.58316227578711
H -6.13880643136852 -0.26248440397575 2.08593759064019
H -5.33553429500301 0.86266254922852 0.00890701242863
H -2.92476875775596 0.80389602734832 -0.58691182602424
H -0.79352662815383 1.06202831263798 3.32285642839950
H -1.66807061530755 3.12756479848941 4.39028522566663
H -1.94662899145744 5.20553684509843 3.03610763680582
H -1.42689043853534 5.17799454171118 0.59660170905504
H -0.59371385610635 3.10601840556073 -0.47498145286401
H 5.11849353822282 2.33037174703351 0.00851488356908
H 6.93995372017549 0.67736180624547 -0.47910338776886
H 6.38826227478508 -1.73536248153953 -0.70647516925859
H 4.06733558015686 -2.53819562323937 -0.54770312320519
H 3.13954499336652 4.27468396319606 0.42820482128762
H 2.75361843534304 6.04986680003478 -1.23542480846606
H 1.54499548035958 5.54778500732320 -3.36053302364461
H 0.73324325576009 3.22671667475367 -3.79999376518025
H 1.07713062864553 1.44887845039993 -2.11334248132874
H 2.73136802002008 1.27357990192320 2.24414468395730

76
XIV 2 1 NImag=0 CorrGibbs=0.549285 ZeroPointC=0.615469 dipole=[-0.0583671,4.7969574,0.0776566] polarizability=[798.5922899,-1.0592933,992.1284687,-100.3323747,4.1737,577.729409] E_bp86_TZVPD_GAS=-1804.706765717 E_b3lyp_TZVPD_GAS=-1803.510019874 E_wb97_TZVPD_GAS=-1803.793458216 G_solv_hfip=-0.1894002798555616 E_b3lyp_d3bj_tzvp_smd=-1804.8365438 E_b3lyp_d3bj_def2SVP_smd_hfip=-1802.92583601 LowestF=18.4266
C -0.70650857551491 0.61516959714821 0.32361582181107
C -1.98845909296227 1.19475766421237 0.81161609252723
C -2.21213666086459 1.22535434388666 2.19637382357040
C -3.40974409750597 1.72886364888355 2.70677421685271
C -4.38921371276670 2.21390291993791 1.83725227757239
C -4.17230141280476 2.17970440195237 0.45533231887613
C -2.98391462298858 1.66180878923922 -0.05727944051143
C -0.70817574050699 -0.61352213620871 -0.32313232644680
C -1.99235246749230 -1.19014698458070 -0.80871319545139
C -2.22281204568169 -1.21101035955172 -2.19247821937116
C -3.42271231712612 -1.71175267880924 -2.70035378413436
C -4.39767121058996 -2.20321357720643 -1.82938563537683
C -4.17405199159715 -2.17824182359501 -0.44833925989187
C -2.98333206754660 -1.66323485332911 0.06183022733066
C 0.54372647200556 -1.22536341809514 -0.67563457824612
C 0.60931817364499 -2.56736484461037 -1.19784356730780
C -0.42200934391227 -3.59878770919103 -0.96690151343656
C -1.00795619694203 -4.29832761382591 -2.03112896745208
C -1.95294365789822 -5.28948066531085 -1.76618262589808
C -2.29107648358003 -5.60403039941799 -0.44662765935348
C -1.68735761648339 -4.92253692227108 0.61415997836159
C -0.76156621050843 -3.91313453059641 0.35895086872263
N 1.72551157915445 -2.96706649633110 -1.78821123953375
C 2.85595088965310 -2.19038724685318 -1.95361067761142
C 2.92339379707639 -0.95788244691824 -1.25564949038401
C 4.05929203328248 -0.14621542041064 -1.49529203233587
C 5.07876453639055 -0.58156024229990 -2.32210081843629
C 5.01533896387452 -1.84442590077883 -2.94615432592583
C 3.90243096173660 -2.64615751233610 -2.77289788841426
C 1.77836629731865 -0.55569404456267 -0.46218529427170
C 1.77994903631199 0.55068503561478 0.45811182970229
C 0.54756691825090 1.22445767382124 0.67265387912768
C 0.61922043221899 2.56772722730793 1.19110663612064
C -0.41005009417239 3.60188428717206 0.96517072901960
C -0.98410312460605 4.30560712407185 2.03341755373543
C -1.92973974982473 5.29773121296748 1.77480507215201
C -2.27986583116397 5.60936865304819 0.45765348589149
C -1.68708707521146 4.92454051751494 -0.60718607173097
C -0.76093238099861 3.91393838282031 -0.35817675847240
N 1.73862314169638 2.96499602937520 1.77722408670904
C 2.86554139985291 2.18407134354894 1.94474847384642
C 2.92654446480336 0.94932321704418 1.25075311954610
C 4.05833207390515 0.13254252603588 1.49193925694349
C 5.07994943158030 0.56503508327467 2.31760239996313
C 5.02257157756702 1.82980319446187 2.93865928963423
C 3.91395896126412 2.63701702120743 2.76305284522786
H -1.44129331699831 0.85084329373668 2.87481376830494
H -3.57453908376796 1.74711774312456 3.78713502858725
H -5.32384653386813 2.61833894545389 2.23445977368890
H -4.93558288372651 2.56021169222325 -0.22811861812047
H -2.81720517548951 1.63679436319990 -1.13525311920500
H -1.45602675420917 -0.83061331010751 -2.87232954920419
H -3.59312863968909 -1.72235899850104 -3.77993764946978
H -5.33397033923057 -2.60555024320057 -2.22479462846234
H -4.93370745622357 -2.56383358101208 0.23630435056772
H -2.81139242889141 -1.64521896027419 1.13912829229981
H -0.75502046608290 -4.03961291329326 -3.06143123338657
H -2.43059226864859 -5.81597914494924 -2.59584850985623
H -3.03054036796673 -6.38300289526969 -0.24465067535279
H -1.94623198374719 -5.17107182751385 1.64616070505942
H -0.29586301377609 -3.36746608131288 1.18244814466540
H 1.76640618805561 -3.93525464857821 -2.11926148673348
H 4.12084996702389 0.84596711894396 -1.05221619748769
H 5.93882331566056 0.06745757874717 -2.50086352539813
H 5.83396995316512 -2.17992742188791 -3.58639030797634
H 3.81111011900995 -3.61411432458032 -3.27079337527886
H -0.72165215602979 4.04884923568618 3.06192714688149
H -2.39905014663909 5.82705411796489 2.60742015585788
H -3.01996954273920 6.38905836197746 0.26086401359994
H -1.95505565239738 5.17135052655005 -1.63726216375126
H -0.30321232380066 3.36573883005428 -1.18460265687345
H 1.78537201679851 3.93460805871836 2.10319194203122
H 4.11356388894544 -0.86098966487647 1.05096038531286
H 5.93687742941519 -0.08759662361605 2.49821158723934
H 5.84253333493043 2.16246700189362 3.57867453556172
H 3.82657543803677 3.60629183291376 3.25900754022487

76
XI 2 1 NImag=0 CorrGibbs=0.549189 ZeroPointC=0.616236 dipole=[0.7707805,3.4091279,-0.0773916] polarizability=[967.0648203,26.8566959,907.7705589,71.4021099,-109.1311807,520.7367999] E_bp86_TZVPD_GAS=-1804.72188809 E_b3lyp_TZVPD_GAS=-1803.528534916 E_wb97_TZVPD_GAS=-1803.812590886 G_solv_hfip=-0.1868556170799465 E_b3lyp_d3bj_tzvp_smd=-1804.8456223 E_b3lyp_d3bj_def2SVP_smd_hfip=-1802.93372129 LowestF=15.4779
C -1.28911198475574 -0.51498492508454 -0.33526849107436
C -2.44358447271573 -1.21736977155733 -0.95480584228831
C -2.69658183737543 -2.57681508318706 -0.72355264855397
C -3.79078833726436 -3.19691399472898 -1.32469189764834
C -4.62726167525762 -2.47433050093599 -2.18235787624423
C -4.36440953542758 -1.12633367504193 -2.43711273889003
C -3.28048116255600 -0.49858464839627 -1.82223894501545
C 0.00398708695331 -0.89985410685242 -0.65233823970715
C 0.28733556170361 -2.07358633852494 -1.52518701352198
C -0.03556625819193 -2.03444044407012 -2.89060610249149
C 0.22241466587740 -3.13815024076612 -3.70380236790863
C 0.80373186899617 -4.28973985910937 -3.16341823366986
C 1.12984121274912 -4.33435554513544 -1.80573410732742
C 0.87398016942381 -3.23095559225828 -0.98758042224754
C 1.08703156977707 -0.13348745337974 -0.13191085904235
N 2.36997093291459 -0.52431337309079 -0.45585218840560
C 3.49404794059663 0.04991638657255 -0.03077234939190
C 4.75458876765671 -0.44921109135802 -0.59500177313206
C 5.74252708210568 0.45472848729625 -1.02949308672121
C 6.91523814190209 -0.02526810555243 -1.60947198577763
C 7.11909755653776 -1.40139816433072 -1.74954463143757
C 6.14031619034254 -2.30333410014562 -1.32021387133918
C 4.95799150902124 -1.83388003826542 -0.75272296316172
C 3.38645007131613 1.06467531307510 0.96952315526405
C 2.08531470729540 1.54726825736366 1.33259435153679
C 1.98734759474698 2.39828557887020 2.45468631668237
C 3.11897164370140 2.81246978195086 3.13561839111388
C 4.40017404879030 2.38129604602419 2.74088753947017
C 4.53128851940372 1.50347733373170 1.68449115544804
C 0.91887085437796 1.04042896653616 0.62684681076739
C -0.41377748619065 1.56753468818988 0.71540603556565
C -1.49331768359748 0.68178315037409 0.43801628210705
C -2.78218545795915 1.02170891190918 0.97535007948548
C -3.83639789919564 0.03154308764998 1.26327893798520
C -3.50444508043176 -1.10628571693345 2.01728623435674
C -4.48823055184441 -2.04259677891428 2.32698873786594
C -5.79699075721302 -1.86094986800819 1.87051377721818
C -6.12847422491630 -0.72753024363976 1.12193284433483
C -5.15614946171716 0.22761112134765 0.82864173254028
N -2.99912428168455 2.26830591818202 1.37304117973868
C -2.07948665707384 3.29437845716946 1.27640382355225
C -0.73936009196459 2.96126374929745 0.95709523190110
C 0.16170002614345 4.03675827113117 0.76491704560430
C -0.24444371023299 5.34462393978385 0.95854193424497
C -1.56457854082836 5.63927145209617 1.35605850538388
C -2.48649981669863 4.61937214823736 1.50553732417128
H -2.04981227086804 -3.14063884328818 -0.04913853132986
H -3.99448688126079 -4.25116307265880 -1.12110810180879
H -5.48325515726464 -2.96464444727788 -2.65309777624236
H -5.00965179491927 -0.55790178580016 -3.11168758143356
H -3.07844778058616 0.55817064972244 -2.01412757720731
H -0.49371089968631 -1.13691518692438 -3.31126166403815
H -0.03323117316912 -3.09955528257026 -4.76572081235041
H 1.00172651262411 -5.15412009700797 -3.80245917216368
H 1.57868784752416 -5.23408214233921 -1.37734974712480
H 1.11205765835960 -3.27315086162988 0.07853198286658
H 2.44575178022365 -1.29121915357625 -1.13047041791562
H 5.57608340820810 1.52994662030667 -0.94622572516574
H 7.67261186653184 0.68038649057857 -1.95880514113434
H 8.04389785654458 -1.77313467567535 -2.19777815160923
H 6.30053372687517 -3.37902588988814 -1.42366176428386
H 4.20500712836974 -2.54545102563681 -0.40496160260123
H 1.01040982970745 2.71057108159226 2.81693305885549
H 3.01051106551551 3.46285265806540 4.00685694199858
H 5.28297855695381 2.70492996514669 3.29603116730098
H 5.51278283786008 1.10606790209351 1.42941380359683
H -2.47759691396213 -1.24939044077144 2.36027502872163
H -4.22973400399869 -2.92432320513541 2.91812225677822
H -6.56265913780048 -2.60697610871410 2.09765122087412
H -7.14919586457068 -0.58946470218026 0.75763579182589
H -5.40947158601373 1.10161009459862 0.22427382216960
H -3.90762183921562 2.48626965981100 1.79028924457947
H 1.17712432620461 3.83791779035249 0.42786294150141
H 0.46515398075739 6.15784246181871 0.79119083573382
H -1.86806976105525 6.67561363453428 1.51982420846770
H -3.52504451256000 4.82279704223215 1.77589972176231

78
TS-XIV 2 1 NImag=1 CorrGibbs=0.55171 ZeroPointC=0.62079 dipole=[0.5405663,-3.0999655,1.166457] polarizability=[893.931003,20.3948119,1081.4880339,18.3840217,-42.6011502,552.2550715] E_bp86_TZVPD_GAS=-1914.154143279 E_b3lyp_TZVPD_GAS=-1912.881343961 E_wb97_TZVPD_GAS=-1913.169561945 G_solv_hfip=-0.18850456429551024 E_b3lyp_d3bj_tzvp_smd=-1914.2542988 E_b3lyp_d3bj_def2SVP_smd_hfip=-1912.21227802 LowestF=-525.9177
N -1.27130279142249 0.95132119208683 -4.82790543637464
N -0.60386336507216 0.80842210016831 -3.95479402627530
N 0.98312839137708 1.05558636907391 -3.37931224706001
C 0.97739473954410 1.58825487340472 -2.04158050548071
C 0.33773224258274 2.95451197639193 -1.90472879940107
C 0.14867902959285 3.47600607504608 -0.61667113732923
C -0.42673220354453 4.73385995552518 -0.44694818793879
C -0.81615993125079 5.48442760492125 -1.56175347436133
C -0.60433751902888 4.97723496816805 -2.84468271260410
C -0.01836416178672 3.71993543426332 -3.02001089679585
C 2.51352412359498 1.67184287642898 -1.94903569116083
C 2.97039050850432 0.83320155047546 -0.90452714365493
C 4.29946330168579 0.96869824991236 -0.49271914989863
C 5.14482259721112 1.84474180961691 -1.18463658024610
C 4.68676995071225 2.61193115019758 -2.26472211788344
C 3.34753447119428 2.54391391766216 -2.64856546413804
C 1.81919453974316 0.09757954476587 -0.34046841720888
C 0.64280335243084 0.57155602915392 -0.94415051228775
C -0.63175336348900 0.07790040693068 -0.61832782323811
C -1.80370701162482 0.28337403813999 -1.52301332622617
C -2.27747845205549 -0.83988548442745 -2.23328661371299
C -3.36195584699535 -0.72383028884951 -3.09643632889942
C -3.98916033780738 0.51629985302782 -3.26570576589570
C -3.54941521874023 1.62783817211242 -2.53713692000064
C -2.46839478241643 1.51496665343863 -1.66518303880751
C -0.75288405555039 -0.73508607800092 0.51977276072089
C -2.08543721361765 -1.29238044062351 0.86435582733718
C -2.26000316009436 -2.68353520295648 0.91230241938916
C -3.51041122642436 -3.22807760095660 1.20745603294768
C -4.59855582559283 -2.38789874785925 1.45624780772843
C -4.43307216843250 -0.99978252999759 1.40051167721146
C -3.18686826168131 -0.45436073754132 1.09945190245159
C 0.42883538137331 -1.12688570329201 1.22900924748069
C 1.72884607486042 -0.89937066024888 0.66382992535701
C 2.82055077655477 -1.74893697018027 1.10466289432795
C 2.65820514529897 -2.48274414730706 2.30341965345883
C 3.65916207748948 -3.33016896734560 2.80415530318375
C 4.81999311505945 -3.50437027799573 2.07045064275788
C 4.97115511163990 -2.85633182176197 0.82977375168154
C 3.99319261163507 -1.99695923908840 0.35570959575829
N 1.46644486151680 -2.34804397027325 2.99153340029335
C 0.37544376474789 -1.75396748483894 2.52631101199688
C -0.76658197889368 -1.69251820529414 3.45536349715188
C -1.38305570077186 -0.46034937638198 3.72414852853157
C -2.43705450410322 -0.39766041832327 4.63275568502796
C -2.88873972017090 -1.55936180916010 5.26614533092586
C -2.27177387381844 -2.78659514759326 5.00486436973067
C -1.20524026742830 -2.85581317186380 4.11057231893351
H 1.25226310169172 0.05710029744105 -3.35720697513908
H 0.44130571053660 2.89252787992269 0.25906711231047
H -0.57725614549569 5.12822044525925 0.56100528800239
H -1.27654429427730 6.46661946020642 -1.42875851919933
H -0.89260871951862 5.56181789444553 -3.72179313796990
H 0.15627948448673 3.35630651436757 -4.03280612169272
H 4.68446229432827 0.42628054431494 0.36942368480575
H 6.18480566615997 1.93823654752670 -0.86268987849634
H 5.37024400804352 3.28554397271877 -2.78584375984794
H 2.95372693095859 3.17234245910081 -3.44896582736084
H -1.77535669473289 -1.80131556712571 -2.10804970033770
H -3.71213223824789 -1.59718796992205 -3.65169044949642
H -4.83199223709356 0.61356083750251 -3.95446637209053
H -4.05323067941744 2.59067474462416 -2.64970238163151
H -2.14987391487350 2.37857893183698 -1.08410538815421
H -1.40904497463603 -3.34021430664096 0.71504724115934
H -3.63462235478473 -4.31333769290940 1.24429986803392
H -5.57683241762524 -2.81377749090382 1.69353211528453
H -5.27992734130303 -0.33842988444181 1.60016390920733
H -3.05753895631162 0.62901588119856 1.07719531059971
H 3.49683821365049 -3.85579087577626 3.74772021289950
H 5.60396427663711 -4.16809717663777 2.44162858239585
H 5.86303517422543 -3.04040935374450 0.22673449144416
H 4.11251621519804 -1.54923392214329 -0.62823509627449
H 1.42629579537430 -2.74023414585247 3.93533305132352
H -1.03045083984777 0.44401068476292 3.22517466472484
H -2.91326281524758 0.56284935770795 4.84310145539618
H -3.72638679851654 -1.50839718681691 5.96630086625669
H -2.62846173360066 -3.69625191082417 5.49367975342291
H -0.74040017137289 -3.81923657275961 3.88637670481745

78
TS-XI' 2 1 NImag=1 CorrGibbs=0.551143 ZeroPointC=0.620141 dipole=[-0.0204724,3.2346999,0.9188467] polarizability=[918.1532231,-12.324167,994.1850513,-12.7963367,57.1670199,586.8222943] E_bp86_TZVPD_GAS=-1914.14762835 E_b3lyp_TZVPD_GAS=-1912.87584999 E_wb97_TZVPD_GAS=-1913.167532217 G_solv_hfip=-0.187108266518691 E_b3lyp_d3bj_tzvp_smd=-1914.2499064 E_b3lyp_d3bj_def2SVP_smd_hfip=-1912.20660797 LowestF=-598.9611
N 3.89149710227574 3.98890576833946 -1.71187882017606
N 2.86268216975281 3.60574797677042 -1.58703000807340
N 1.83930988837578 2.28920891093630 -1.30146908502222
C 1.53722908209247 2.23428547719600 0.11930842637206
C 0.71976475399727 3.35111426665000 0.72020393097945
C 0.32261836794020 4.47813569393891 -0.00800223499711
C -0.44857338519664 5.47016559413791 0.60403126920867
C -0.83314069735569 5.33831909224894 1.93823942569736
C -0.43609813026030 4.21167847323404 2.66871505631421
C 0.33895464744122 3.22496475122654 2.06501181527864
C 2.80864136690422 1.85795908725096 0.86018424192032
C 2.95380058549617 0.45521253399058 0.92106904306702
C 4.03440268408776 -0.08657433941689 1.62998467960053
C 4.96836136928050 0.77998037010785 2.20631839176240
C 4.83443911249257 2.16747215002676 2.09349262779132
C 3.73407981489466 2.72192582268272 1.42169208380564
C 1.77229620223027 -0.16140275611834 0.29790472791677
C 0.84814183723280 0.85356449158343 -0.04822511300801
C -0.47592861906982 0.60475711493213 -0.46761605236177
C -1.36626892722199 1.70823347105243 -0.92757856880013
C -1.35025122584443 2.12412217016672 -2.26640936674940
C -2.15845467146055 3.18854987867040 -2.67904500265150
C -2.98995774841509 3.83274757702170 -1.76070312192404
C -3.02353245086128 3.40450338857907 -0.42937735343661
C -2.21913672839346 2.34480609501769 -0.01388073500310
C -0.93141925918556 -0.71578314842114 -0.41709036774580
C -2.26678813276142 -1.05599779966452 -0.98178415770126
C -2.30138460914254 -1.75168091125475 -2.20096086779309
C -3.52271646041269 -2.09035185957770 -2.78610337799494
C -4.71915442373178 -1.74519026415762 -2.15373114991800
C -4.68937695793707 -1.04599402249947 -0.94231528767680
C -3.47092538945933 -0.69162576780899 -0.36416557642991
C -0.02178773425631 -1.75662486946757 -0.03864951752186
C 1.38065792216992 -1.51334130227241 0.11907643957934
C 2.28868896433786 -2.63951713440919 0.00918515243070
C 1.74989819937468 -3.94807043710375 0.07632094437019
C 2.55099087379329 -5.09392616270881 -0.04394052571017
C 3.90425586856195 -4.94269489628844 -0.29348552051309
C 4.45242177110989 -3.65474296047081 -0.44500838366527
C 3.66285660936337 -2.52556290960190 -0.29677301550032
N 0.38775349045496 -4.08161604354907 0.28092878288469
C -0.49125413080057 -3.09410700374579 0.22669843789348
C -1.87973869064132 -3.42065684571033 0.61003208490288
C -2.41783611238180 -2.80555688970849 1.75169833354061
C -3.70794668826433 -3.12883706827235 2.16686569495124
C -4.47018506174253 -4.04459891841578 1.43566867393223
C -3.93267498703679 -4.65687314009474 0.29948635949973
C -2.63171982939055 -4.36114302163613 -0.10793045600712
H 1.03156334305786 2.65545208674296 -1.83745776194002
H 0.57050700674510 4.60082661712195 -1.06396403320136
H -0.75951515421927 6.34182870318479 0.02374351038509
H -1.44466381704729 6.11053964050972 2.41156178209439
H -0.73312023875757 4.10309880868033 3.71472868787049
H 0.64396383380891 2.34549016271715 2.63687373130222
H 4.14813975654051 -1.16118768979847 1.76404973773919
H 5.80945440262481 0.36185786673397 2.76415994027191
H 5.57112938228757 2.82591064931743 2.55945659651723
H 3.59523778109408 3.80438851723237 1.37725924336271
H -0.71718455455907 1.60237320892016 -2.98995175630943
H -2.13999765324424 3.50891065288336 -3.72381949881252
H -3.61809279888426 4.66723438615069 -2.08278777950041
H -3.67407471859194 3.90489719768131 0.29178285349631
H -2.23136663488902 2.02238324007121 1.02823153801277
H -1.36431253077060 -2.02829387138340 -2.69051226598643
H -3.53831113876681 -2.63173657519900 -3.73546457347571
H -5.67694357559611 -2.01977148796871 -2.60312098669476
H -5.62278735386780 -0.77898176975540 -0.44039467736480
H -3.45227071164599 -0.16317675336343 0.58906842356371
H 2.09319778746840 -6.08175998735548 0.04276993416677
H 4.53960853233129 -5.82476351001969 -0.39868602222413
H 5.50979821226016 -3.54168945887574 -0.69418198965887
H 4.09915997227956 -1.54306036294048 -0.46560995660794
H 0.03386806910230 -5.01556063600716 0.50681874452692
H -1.82191704175138 -2.08014781346144 2.30957758082617
H -4.12409977694658 -2.65523563886938 3.05926434495542
H -5.48908049961229 -4.28200163164475 1.75171823888611
H -4.53077075336186 -5.36694337092346 -0.27650865953005
H -2.21331157256716 -4.82646589319448 -1.00293354019491

74
VIII 2 1 NImag=0 CorrGibbs=0.532887 ZeroPointC=0.5978 dipole=[1.1165645,3.0098725,0.0086484] polarizability=[1284.7536736,13.4560578,998.1810769,24.8559219,-66.5531673,455.4394962] E_bp86_TZVPD_GAS=-1749.287584806 E_b3lyp_TZVPD_GAS=-1748.128147937 E_wb97_TZVPD_GAS=-1748.398334353 G_solv_hfip=-0.1839256374127257 E_b3lyp_d3bj_tzvp_smd=-1749.409415 E_b3lyp_d3bj_def2SVP_smd_hfip=-1747.55758563 LowestF=16.3199
C -1.24168944287176 -0.44629980492696 -0.38647029382039
C -2.33643629902762 -1.19294635379072 -1.05954490227519
C -2.87458992249998 -2.34657565598312 -0.47130593492071
C -3.91852433342570 -3.02936942888333 -1.09568545835652
C -4.42255794415386 -2.57577442670688 -2.31816626666651
C -3.87041425770041 -1.44173513144648 -2.92140063443943
C -2.82926403237823 -0.75460224352481 -2.29762191104360
C -0.00267188274085 -1.08279720000014 -0.18975981469779
C 0.23232943948549 -2.48520169168623 -0.63416989319091
C 0.21619230887594 -2.80969075156469 -2.00022628547756
C 0.42777834130756 -4.12592087899843 -2.41044875769680
C 0.65655753795958 -5.12998673146786 -1.46376548924350
C 0.67788224758020 -4.81340750874650 -0.10328280143724
C 0.46746219758259 -3.49611171069275 0.31205324799357
C 1.04962180921985 -0.35524541311973 0.40356224346700
N 2.29529366285047 -0.94949310341083 0.46546336738643
C 3.40742175277538 -0.40066764929675 0.95223398148196
C 4.65344840243614 -1.14774315769714 0.76203653165472
C 5.82319578532026 -0.47949802414001 0.34942919059368
C 6.98886855193215 -1.20438380974234 0.11231701689498
C 7.00561924501575 -2.59028289480828 0.29733212430025
C 5.84701053161813 -3.25879146384942 0.70698693652280
C 4.67138130918578 -2.54704119385584 0.92926029965000
C 3.29278470406025 0.84342690282281 1.65580067230282
C 2.03500516779898 1.52639869225549 1.65336175829410
C 1.87161593971228 2.61561444126619 2.52566634522513
C 2.92953715073430 3.07415008575714 3.30112622178543
C 4.18168997992544 2.44145065685805 3.25457755139356
C 4.35558234342385 1.32219976058538 2.45939226696104
C 0.93807712746344 0.99954461252327 0.84892615973703
C -0.23929040259498 1.66301661737000 0.48378336471526
C -1.35054269364759 0.91926394191404 -0.01784769761645
C -2.47312957670709 1.83233237522422 -0.18213289293074
C -3.83897381553432 1.49122534480248 -0.47863606018798
C -4.55508724975192 2.20811227252797 -1.46729303107607
C -5.84803101537391 1.82187842367352 -1.80157274340439
C -6.46040345450110 0.76051507657408 -1.12555810157317
C -5.77507782889369 0.07258750123824 -0.11428065366967
C -4.46836500648173 0.41732685787813 0.19637512172061
C -1.98546135468299 3.17129473992217 0.04025764483508
C -0.61257582750953 3.10510766921718 0.42731818873033
C 0.10705292826923 4.27762480514942 0.56230177561266
C -0.56001100577715 5.51334071062105 0.38423291326717
C -1.92332404575694 5.57704830382961 0.10401768721520
C -2.65709568610141 4.39573380425075 -0.06521503887866
H -2.48966456940231 -2.69470237427943 0.48922049296365
H -4.34334709212519 -3.91844405911428 -0.62281082781890
H -5.24354447372817 -3.10924616023275 -2.80409665811889
H -4.25325397450495 -1.08853313821450 -3.88206286744490
H -2.39889432840232 0.13219292518800 -2.76773731334734
H 0.03426350463232 -2.02644166325596 -2.73898965922846
H 0.41267379686054 -4.36975573809165 -3.47572395487263
H 0.81828784034157 -6.16110591719947 -1.78833343957638
H 0.85163223181419 -5.59496115454186 0.64063331426160
H 0.46730359097899 -3.25142140971686 1.37747063613821
H 2.37441606917738 -1.86593378053234 0.01441258044618

H 5.80669802573115 0.59751774159365 0.17583178525224
H 7.88870951779239 -0.68447510583521 -0.22436722952375
H 7.92503675373212 -3.15338132279034 0.11856145693864
H 5.86085120186078 -4.34054951244873 0.85831010173452
H 3.77624445523939 -3.07568289918764 1.26574008779018
H 0.89392321615049 3.07881070858808 2.63763904629307
H 2.77201198918357 3.91956496748083 3.97513670876744
H 5.00263031401413 2.79977797153721 3.87892074571014
H 5.29909219712846 0.77940167406084 2.49284489041451
H -4.06035209337239 3.00324614808640 -2.02618624442434
H -6.38119520223955 2.34463433236317 -2.59873827662209
H -7.47913333419445 0.46471048913729 -1.38821639889302
H -6.26372107497741 -0.74333890945967 0.42221668617174
H -3.93171862741439 -0.10827814340295 0.98568491557236
H 1.17076720338363 4.28287824320397 0.79398391235044
H 0.01344470437199 6.43727434437138 0.49115782968976
H -2.41944251446489 6.54479694784479 0.00930484171632
H -3.72636130893928 4.43717369072701 -0.27583822455177

76
X 2 1 NImag=0 CorrGibbs=0.550335 ZeroPointC=0.616808
dipole=[0.0004473,2.2387775,0.0001962] polarizability=[1070.240617,-
0.0369585,910.0410787,19.2956498,0.0273934,446.7815192] E_bp86_TZVPD_GAS=-
1804.731283355 E_b3lyp_TZVPD_GAS=-1803.54048706 E_wb97_TZVPD_GAS=-
1803.824977575 G_solv_hfip=-0.18426995093934104 E_b3lyp_d3bj_tzvp_smd=-1804.8496585
E_b3lyp_d3bj_def2SVP_smd_hfip=-1802.93673602 LowestF=20.6919
C -1.37599212479976 0.54971177491839 0.42215781568439
C -2.64583383159600 1.13982680073870 0.92381686453594
C -3.50184072682444 0.38134535153421 1.73851353469553
C -4.70024041724630 0.92883740035802 2.19656540559113
C -5.05812994573260 2.23415777445553 1.84392158052052
C -4.21249970677177 2.99337456136032 1.03125502077293
C -3.01003756659528 2.45064927701257 0.57237522021622
C -1.37563490259901 -0.55086635393059 -0.42182481017944
C -2.64511146564130 -1.14196231293908 -0.92325348944445
C -3.50180570964262 -0.38413819248049 -1.73784107952201
C -4.69988903920807 -0.93251781161409 -2.19565694785923
C -5.05677353269529 -2.23807800702165 -1.84288093681114
C -4.21045179090883 -2.99664436831766 -1.03032618179905
C -3.00830104958836 -2.45302874407093 -0.57168028525305
C -0.12354740706778 -1.12730529641436 -0.78374222179996
N -0.12371742745531 -2.20168972785259 -1.65227366861431
C 0.94285621027963 -2.90157970662941 -2.02814403815859
C 0.75173133054959 -3.91462533496531 -3.07533698103688
C -0.37047017800304 -4.76432302585266 -3.04764106984488
C -0.56309468252860 -5.69613665333341 -4.06550119927293
C 0.34773131201900 -5.77903300102882 -5.12334529305838
C 1.45653673312686 -4.92824644847668 -5.16219197241673
C 1.66597848538060 -4.00198582937651 -4.14202956268026
C 2.17623062288776 -2.64675826395371 -1.35031518333072
C 2.25666300891797 -1.53388461728575 -0.45067801202661
C 3.39978823378729 -1.43189113251565 0.37231484690559
C 4.44384673601643 -2.33278379251030 0.25571726922698
C 4.38959112795369 -3.38605413193971 -0.67732842926390
C 3.26290778129529 -3.55306450565217 -1.45671368941293
C 1.11564960338427 -0.63976799798275 -0.32444379533163
C 1.11519362626344 0.64065976012284 0.32419267456834
C -0.12429096264549 1.12715867705981 0.78381286011138
N -0.12512708959052 2.20151808480585 1.65237453019492
C 0.94096515701529 2.90226834269616 2.02800848741643
C 0.74925558134068 3.91512029748128 3.07527794143737
C -0.37370026993487 4.76383001076211 3.04790604388791
C -0.56685602549071 5.69546693518324 4.06582644048567
C 0.34419641958895 5.77915332617647 5.12341423783529
C 1.45375457094713 4.92933430850064 5.16194587614039
C 1.66372125164122 4.00326466506991 4.14171782568634
C 2.17436769115804 2.64850994803696 1.34983392737567
C 2.25547390424377 1.53575331640543 0.45011619279918
C 3.39842854859569 1.43477502331124 -0.37323538720810
C 4.44175509502407 2.33655178844860 -0.25691484353761
C 4.38688944481807 3.38972670873142 0.67620452683214
C 3.26030552985320 3.55573763664714 1.45594728462734
H -3.22395196667274 -0.63846655921378 2.01245729068628
H -5.35990718959416 0.33258276550916 2.83200718549216
H -5.99939894854949 2.65906635911132 2.20186207305111
H -4.49185575492196 4.01030798566952 0.74456740268046
H -2.36087471234483 3.03668173390386 -0.08348375460417
H -3.22469860077700 0.63586184439398 -2.01188012116815
H -5.36009563426604 -0.33677146064245 -2.83101489017459
H -5.99779743312202 -2.66368485861818 -2.20063680382211
H -4.48902196126019 -4.01376699867761 -0.74354461687118
H -2.35859531619202 -3.03855997340824 0.08408637103570
H -1.02567976666697 -2.43866605072711 -2.07413208638822
H -1.07684227619432 -4.72055253991530 -2.21471191614155
H -1.42614113824074 -6.36503289324343 -4.02933029457105
H 0.19179804290315 -6.50920002187258 -5.92147809968884
H 2.16156884077722 -4.98230995670526 -5.99506979799901
H 2.51916664559100 -3.32265647418005 -4.18841347839909
H 3.45346997039986 -0.65765722094312 1.13478537279164
H 5.30986352975028 -2.23594422914828 0.91482603264261
H 5.21653333279466 -4.09523531377354 -0.75239079191890
H 3.18590751289431 -4.41311804941138 -2.12062264751705
H -1.02718730324540 2.43778716768563 2.07442287597076
H -1.08027605092354 4.71943120718524 2.21517938863453
H -1.43049906985503 6.36360578524505 4.02990387635792
H 0.18784899329576 6.50917731782604 5.92159705345397
H 2.15896992528840 4.98400529961066 5.99462901394243
H 2.51751438990208 3.32468109306281 4.18785748681159
H 3.45251682460833 0.66062576110601 -1.13576178151032
H 5.30765028025673 2.24048866402865 -0.91629662870251
H 5.21325056032901 4.09960804217133 0.75104793125414
H 3.18277586994938 4.41568413498238 2.11993351498203

78
syn-III 1 1 NImag=0 CorrGibbs=0.550563 ZeroPointC=0.619581 dipole=[-
4.5247469,1.3811825,1.7925047] polarizability=[772.0079469,-7.5935327,816.9650056,-
4.3590282,34.2182907,589.0042025] E_bp86_TZVPD_GAS=-1934.932560714
E_b3lyp_TZVPD_GAS=-1933.655870174 E_wb97_TZVPD_GAS=-1933.964517129
G_solv_hfip=-0.09269577975158855 E_b3lyp_d3bj_tzvp_smd=-1934.9063591
E_b3lyp_d3bj_def2SVP_smd_hfip=-1932.83908581 LowestF=22.9938
O 1.01462504236167 3.75151891716120 0.46723329573871
C 0.66021277013594 2.73991850247843 1.40307825443717
C -0.62154003999265 3.08313397751290 2.16032774334343
C -1.15235645744543 2.16429890472601 3.07705152416359
C -2.35840670340410 2.42973865452845 3.72551113332779
C -3.04560125432786 3.62234007953065 3.47001274800147
C -2.51087867721543 4.54669320959044 2.57042774718015
C -1.30234784572044 4.27916221786539 1.91967157756936
C 1.81667053753659 2.42381053346455 2.34326634205512
C 2.35802812983216 1.14832662931979 2.07527929884390
C 3.36095864454128 0.64913025730450 2.91453750651335
C 3.84827208469581 1.45080366797608 3.95401691445241
C 3.33585992537490 2.73260615696820 4.17682255016165
C 2.29500223164365 3.21983632514346 3.37520001065462
C 1.60476183844860 0.54944754244817 0.94906922115466
C 0.54005744859298 1.42818572197928 0.61913129190803
C -0.47505537401820 1.10505123197326 -0.28013605878114
C -1.60550526653475 2.03733863406096 -0.55230157656743
C -1.42585681056653 3.16558208364015 -1.36368429171757
C -2.48388306661491 4.05197714833725 -1.58152286647137
C -3.72814633221826 3.82079334785286 -0.98909497991334
C -3.91569040005639 2.69121823948603 -0.18616779172678
C -2.86285242597772 1.80035750027309 0.02409146745110
C -0.43510174005329 -0.16594088595490 -0.90548039523626
C -1.50326223907389 -0.59985839849599 -1.85279327367972
C -1.38117268473921 -0.32605933504388 -3.22596013863568
C -2.33331848963674 -0.80911671696529 -4.13133077171471
C -3.41996803456860 -1.55595130007998 -3.67056656113248
C -3.56198006695554 -1.80408980024709 -2.30161649904980
C -2.61160315896900 -1.32818173581347 -1.39769094311484
C 0.65495861892779 -0.99264528792290 -0.64618082135448
C 1.71163056932275 -0.65581517772680 0.23652300237044
C 2.72909728786868 -1.73009608461395 0.16232899097069
C 2.24218012798371 -2.75928441572857 -0.67372009461017
C 2.96818309952592 -3.91100287095002 -0.94283205558980
C 4.24850520552664 -4.03402614520724 -0.38775457792566
C 4.76981828640401 -3.00212316662070 0.39814624980650
C 4.02356615886526 -1.85004432975352 0.67712441067254
C 0.89637674326844 -2.37087998492634 -1.25575008506938
C -0.21626404714847 -3.37759447356691 -1.00101224324700
C -0.67947141020838 -3.50283824274722 0.31710050601213
C -1.71799501073894 -4.38270445382765 0.61403405012780
C -2.30721335001236 -5.14524846338861 -0.40096606988257
C -1.83419222647333 -5.03532718593046 -1.70838410492749
C -0.78562648258142 -4.15965213096119 -2.00911111858909
N 1.03952418735658 -2.22971801677258 -2.77163026016436
N 2.16294333606122 -1.93770546002424 -3.26433088856182
N 3.17771273028029 -1.73741679151468 -3.68671313379330
H 1.42824133971323 4.49139357993178 0.93876164744298
H -0.62591123264345 1.22589586705941 3.27145144727777
H -2.76774794233133 1.70164440540607 4.43123752255165
H -3.99490459040877 3.82783184432643 3.97196382843316
H -3.04152679417164 5.47966359300350 2.36208108901878
H -0.90066593837633 4.98926398980595 1.19610711945300
H 3.74259010837358 -0.36326588161768 2.80614393905470
H 4.63152155420305 1.05849442067799 4.60779040508613
H 3.72696239729547 3.34293838314132 4.99485322434124
H 1.85030879193003 4.19853550624985 3.57468993847093
H -0.44651542072875 3.35926202393013 -1.80570031476916
H -2.33245558922341 4.93201429711471 -2.21243264952955
H -4.55203055685646 4.52079576726474 -1.15204477913858
H -4.88486354278707 2.50606701099745 0.28425504670613
H -3.00535977846349 0.92864041781847 0.66532036837553
H -0.54430052089986 0.28122537640476 -3.58295582589456
H -2.22538537092103 -0.59401245888980 -5.19749217632278
H -4.16141762209825 -1.93791453909924 -4.37703229204449
H -4.41411497079635 -2.38162973056187 -1.93478992750499
H -2.71598080597520 -1.54148890393234 -0.33328255249606
H 2.55497840417354 -4.69553458735767 -1.58181819824177
H 4.84387796133893 -4.92891005391644 -0.58322784094962
H 5.78139157635871 -3.08961843533280 0.80261812988761
H 4.47688788480971 -1.05205896612076 1.25999522116826
H -0.23562166127394 -2.89835111887526 1.11112268433953
H -2.07561490127568 -4.46778038195259 1.64327550858051
H -3.13031581671283 -5.82590622628565 -0.16921994538580
H -2.28230286116407 -5.62992748285910 -2.50830714379987
H -0.43756883901187 -4.08993206419356 -3.03996833704481
H 0.22648194368403 -1.87322499117014 -3.31471285878586

74
II 1 1 NImag=0 CorrGibbs=0.527762 ZeroPointC=0.593107 dipole=[2.8139359,2.4781134,-
0.2333268] polarizability=[1079.5343475,23.5034977,971.3985207,-75.8276246,-
60.7502891,502.0042748] E_bp86_TZVPD_GAS=-1770.052512981 E_b3lyp_TZVPD_GAS=-
1768.882462681 E_wb97_TZVPD_GAS=-1769.161492976 G_solv_hfip=-0.08552951344668899
E_b3lyp_d3bj_tzvp_smd=-1770.0401893 E_b3lyp_d3bj_def2SVP_smd_hfip=-1768.15914622
LowestF=17.2884
O -1.01393483044627 -1.98609844708717 -3.04206112149400
C -0.30369562543287 -2.34659952178736 -1.86820770570915
C -1.09128384022460 -3.31297530835667 -0.98482271266381
C -0.56403779782310 -3.70740070597251 0.25333684365593
C -1.30286810532919 -4.53135963548638 1.10287028553697
C -2.57310232638676 -4.97623238282059 0.71937933925065
C -3.09253891571599 -4.59824870920118 -0.52092812042575
C -2.35475459320623 -3.76876534071155 -1.37027059218102
C 1.08547019721130 -2.85590305707350 -2.22552498021638
C 2.08791615547357 -1.95341080030427 -1.81410370651666
C 3.41200844389432 -2.17240254275525 -2.21739522656534
C 3.71725375932842 -3.31608900482435 -2.95959745427599
C 2.72116164728396 -4.23910343717303 -3.30386519277886
C 1.38908046474224 -4.00582094980332 -2.94349538784872
C 1.45554948178853 -0.86996770810259 -1.04196566968684
C 0.02786457482958 -1.05475537107238 -1.11139840324267
C -0.88987427150902 -0.21716074334474 -0.49715284836670
C -2.34115028504156 -0.55986409349718 -0.46636416718515
C -2.90355416849906 -1.03603901445689 0.72784223331309
C -4.24655966368663 -1.40839480926303 0.77476578504857
C -5.04600459860543 -1.29107301698383 -0.36705563590219
C -4.49510561094306 -0.79899466831907 -1.55303843676210
C -3.14669941155304 -0.43732237295837 -1.60524828562449
C -0.40344714509598 0.92810414053494 0.20523147314267
C -1.35959549376926 1.77592141867573 0.95498217622840
C -2.42298544666817 2.41577621976900 0.29873882241631
C -3.29185438273068 3.24120483716601 1.01110780001841
C -3.12259383942198 3.41943936608899 2.38765534749801
C -2.08311892514884 2.76147062420262 3.05199705152212
C -1.20722238497598 1.94219577640366 2.34073483032140
C 0.99175020316384 1.14682251184934 0.23646404730671
C 1.93444883121554 0.18798225857010 -0.28922761182541
C 3.28003956493216 0.61449832818673 0.16150506373727
C 3.13120650881119 1.88082835596761 0.81014556607362
C 4.23828153131210 2.59056568821165 1.29749807756493
C 5.49883516567742 1.99166152635120 1.22755504607911
C 5.63227480545520 0.71482706352107 0.67673307012924
C 4.53078007092522 0.01861547898039 0.13457830675121
C 1.73546901095795 2.23498907542009 0.83319312269605
C 1.22041735960880 3.49520689170743 1.32813791745069
C 0.28754095423037 4.23072547000281 0.56466827851666
C -0.23427294591484 5.41913975939303 1.05863703278907
C 0.14326294803877 5.87875492484156 2.32725115825133
C 1.07074360245018 5.16105961845910 3.08849698064287
C 1.62620968434174 3.98628953524619 2.58824332943891
H -0.95135284247008 -2.70626898865449 -3.68970598313525
H 0.42381785953242 -3.35498578990650 0.56252358458011
H -0.88774436735821 -4.82600140427798 2.07044992114260
H -3.15506433874655 -5.61679797798006 1.38732847665270
H -4.08476450671086 -4.93963879561535 -0.82769641599127
H -2.77282319350094 -3.44985214001438 -2.32532833232343
H 4.19703929473360 -1.45231949219432 -1.99994086500565
H 4.74861398469207 -3.48873779318202 -3.27693853844226
H 2.98305188950368 -5.13560594438968 -3.87137675192858
H 0.60189367646029 -4.70811997176115 -3.23055108592345
H -2.27747776158145 -1.13238704182997 1.61748205166675
H -4.67038017574617 -1.79556194222850 1.70490849714834
H -6.09901612602887 -1.58279517896218 -0.33102535204454
H -5.11713399312491 -0.70062510406092 -2.44671092085869
H -2.71126048607375 -0.06950417217999 -2.53545678324319
H -2.54627974846890 2.29359434373696 -0.77837053304053
H -4.10400501505508 3.75206587976539 0.48761136189679
H -3.80381705215527 4.06834888990417 2.94408766077899
H -1.95231442225856 2.88765352259013 4.12960753024123
H -0.39450437578856 1.42867525066154 2.85877891639951
H 4.12270532499758 3.58817201483189 1.72299385757655
H 6.37590760068375 2.51736965843413 1.61015353121861
H 6.61686223606832 0.24124700390503 0.64897031959720
H 4.68699380749140 -0.97809244919417 -0.27017032665708
H -0.00077342215104 3.87031808707415 -0.42307511587294
H -0.94799301185455 5.98813109669917 0.45873125160747
H -0.28957561885013 6.80089024767385 2.72316932484981
H 1.35724901570041 5.51519053540591 4.08157977452073
H 2.31615328368990 3.40525118075976 3.20193883092374

24
SI_I 0 1 NImag=0 CorrGibbs=0.15188 ZeroPointC=0.188223 dipole=[0.2865145,-
0.0381799,0.3151181] polarizability=[287.5291587,-0.7754198,202.5805901,-
54.4850527,2.0623986,113.8102817] E_bp86_TZVPD_GAS=-613.9934182654
E_b3lyp_TZVPD_GAS=-613.6010843687 E_wb97_TZVPD_GAS=-613.7000460127
G_solv_hfip=-0.031257026085882315 E_b3lyp_d3bj_tzvp_smd=-613.957565 E_ccsd_t_=-
612.675762526087 E_b3lyp_d3bj_def2SVP_smd_hfip=-613.277856848 LowestF=81.6587
O 0.78785750863667 -2.44170584032718 -2.78179053891529
C 0.40941318018028 -1.07604542155154 -2.68365274969160
C -1.08765717019681 -0.89776181948382 -2.90007772244579
C -1.71000359812744 -0.37029568211475 -1.74909777175887
C 0.58001472703523 -0.52031743081528 -1.26310607844964
C -0.67845262531573 -0.19406305645815 -0.69782975554874
C -0.71219254532457 0.24601154003735 0.63646776659189
C -1.79869626134592 0.45728281891274 1.62189346323199
C -1.22874952898268 0.94178575805519 2.81746768610517
C 0.28407129814204 1.04812353279860 2.70477957216857
O 0.60952772861038 2.42433065192967 2.83463704356347
C 0.52116041103035 0.51442722070072 1.28260237277353
C 1.75962472889923 0.25897272696615 0.69417599180323
C 1.79066984463564 -0.31391455444835 -0.60256703151209
H 0.24784982500912 -2.95984135125346 -2.16511936254364
H 1.56070970666524 2.54714738330565 2.68978000339559
H -2.77436422524828 -0.15799848793694 -1.63136829268077
H -1.59069188571456 -1.15761537482256 -3.83408559874556
H 1.01342547619820 -0.52560296902219 -3.42371012721459
H 2.73806010571463 -0.57188884878210 -1.08412016989152
H 2.68754293200918 0.46344870765336 1.23671231822447
H 0.80356708783509 0.43196483719170 3.46270485015492
H -1.77870451655998 1.21293135066445 3.72166790947041
H -2.86169116766273 0.29122246458893 1.43843197303935

20
SI_III 2 1 NImag=0 CorrGibbs=0.123624 ZeroPointC=0.156272 dipole=[-
0.0002173,0.1364971,0.0001163]
polarizability=[417.9593385,0.3379767,226.2843223,11.736183,0.0466164,64.4202308]
E_bp86_TZVPD_GAS=-461.5475700734 E_b3lyp_TZVPD_GAS=-461.2411244771
E_wb97_TZVPD_GAS=-461.2990507724 G_solv_hfip=-0.22015289921440379
E_b3lyp_d3bj_tzvp_smd=-461.8043058 E_ccsd_t_=-460.470978670311
E_b3lyp_d3bj_def2SVP_smd_hfip=-461.31267974 LowestF=185.4969
C -1.95424572140532 0.57932415580774 0.44195884283568
C -1.95409164900612 -0.57997319539388 -0.44169994537537
C -0.72569069166713 -1.08343052704012 -0.87000090170956
C -0.43844482339338 -2.17960171372383 -1.79193817349682
C 0.96635946493835 -2.43677609597541 -1.73379740203858
C 1.59272171680327 -1.48608960950605 -0.85996110309551
C 0.54908944865314 -0.56998641664900 -0.37926529008541
C 0.54894318870559 0.57019233766598 0.37913227595620
C -0.72594128192999 1.08331070037759 0.86995234097123
C -0.43891673269532 2.17947367929531 1.79197086620823
C 0.96584423329856 2.43696225565455 1.73381760684979
C 1.59236194388106 1.48658751688728 0.85975111994265
H 2.65138201701829 1.44191115043556 0.60172006784021
H 1.48591878197250 3.23049733222780 2.27003929203377
H -1.17625149645718 2.71378578468834 2.39361103844850
H -2.89535984472099 1.01593269893557 0.78114285067928
H -2.89537747224109 -1.01656654340989 -0.78045124335863
H -1.17487282553066 -2.71484734121176 -2.39385969536890
H 1.48519309790182 -3.23077494705717 -2.27056445955253
H 2.65143316496107 -1.43989665900954 -0.60115927249687

24
SI_Ia 0 1 NImag=0 CorrGibbs=0.151052 ZeroPointC=0.18767 dipole=[-1.0212276,-
0.4132608,0.4404174] polarizability=[289.0118346,-
17.9669967,174.4018421,54.589387,44.1349351,141.6397572] E_bp86_TZVPD_GAS=-
613.9899496922 E_b3lyp_TZVPD_GAS=-613.5976895065 E_wb97_TZVPD_GAS=-
613.6964258291 G_solv_hfip=-0.033496737290391654 E_b3lyp_d3bj_tzvp_smd=-613.9560406
E_ccsd_t_=-612.672655757787 E_b3lyp_d3bj_def2SVP_smd_hfip=-613.275775646
LowestF=80.8065
O 1.77625730225729 -3.06121949364403 -1.21596973183152
C 0.41839150393987 -2.66234784932786 -1.10815627252398
C -0.09555988915172 -3.21165162448623 0.21337747898979
C -0.51391725050008 -2.17312568964077 1.07145297777784
C 0.26087466541476 -1.15267957613741 -0.89195301878318
C -0.35104610625483 -0.88330389558187 0.35893816201454
C -0.64293971472516 0.45430700372895 0.67450248342860
C -1.43228615327441 1.10831919778337 1.74452193111810
C -1.33261015560174 2.50703853065958 1.58125060123138
C -0.46704420062173 2.85818453760231 0.37628510528636
O 0.77321635233908 3.43047977499979 0.77873262727617
C -0.16115638711482 1.46831803589538 -0.19229782616203
C 0.47436214618459 1.19984427082573 -1.40333037112928
C 0.65068897794867 -0.15570261175524 -1.78182710273280
H 2.18265974831609 -2.60970028174097 -1.97394394592189
H 0.60546223313589 4.13297632549809 1.42575070151199
H -1.97303416575359 0.59076540753473 2.53861128504895
H -1.79500324190898 3.25572585068278 2.22880362027201
H 0.80700062446757 2.01473568840593 -2.05145632633917
H 1.10469050181233 -0.41088229986066 -2.74372087709869
H -0.91437034235842 -2.29341263799659 2.07925246948360
H -0.07992936046656 -4.27539109208127 0.45860136523232
H -0.99539677053475 3.50652996455194 -0.34916287145755
H -0.18441308094098 -3.00863115323557 -1.97142347538067

22
SI_II 1 1 NImag=1 CorrGibbs=0.136965 ZeroPointC=0.171199 dipole=[0.4650195,-
0.9036735,0.5432694] polarizability=[346.0948619,-
38.4052081,197.6281801,133.770495,37.8632305,175.1117359] E_bp86_TZVPD_GAS=-
537.8528441598 E_b3lyp_TZVPD_GAS=-537.5018242229 E_wb97_TZVPD_GAS=-
537.5789751315 G_solv_hfip=-0.07290960836590155 E_b3lyp_d3bj_tzvp_smd=-537.8939894
E_ccsd_t_=-536.650976327722 E_b3lyp_d3bj_def2SVP_smd_hfip=-537.309577326 LowestF=-
143.0185
O 3.32549784377009 -0.21651745315506 -0.62950577767766
C 2.15306599575623 -0.86001255013247 -1.10236202174510
C 1.88292444002707 -2.12467387737594 -0.29962398157053
C 0.73809538897295 -1.98638065032597 0.51180423637459
C 0.09746402230297 -0.69629155804227 0.20355136307237
C 0.95476189124247 0.01362619176403 -0.71206209978693
C 0.66851806433188 1.27066757669883 -1.22078000622550
C -0.51852064134089 1.93096438924564 -0.77741057380428
C -1.34014988791507 1.26535651225601 0.15909592355637
C -1.09618870581839 -0.09781247174382 0.56723953260989
C -2.29149970754915 -0.54256902914230 1.32144193119319
C -3.14676669702682 0.59375763891586 1.47434574833733
C -2.55852636001438 1.71851820729684 0.79425367839947
H 4.04277454865925 -0.86655197360549 -0.55874983359712
H 2.18329518779323 -1.04537704874567 -2.19222415127641
H 0.34617296466812 -2.72918814689171 1.20756760411795
H 2.54240440976323 -2.99558767338450 -0.31605279978728
H -2.47372815681391 -1.54974559140949 1.70008746651412
H -4.09354568947203 0.60351724293182 2.01332765754868
H -2.97020009002027 2.72835022239554 0.74316819908220
H 1.33484793831865 1.74460579680063 -1.94386010803176
H -0.77507068087016 2.93121452066266 -1.13418019561438

10 Copies of ^{1}H and ^{13}C NMR spectra

1,1'-(Ethyne-1,2-diylbis(2,1-phenylene))bis(prop-2-yn-1-ol) (S3f)

^{1}H NMR (400 MHz, $CDCl_3$, 25 °C)

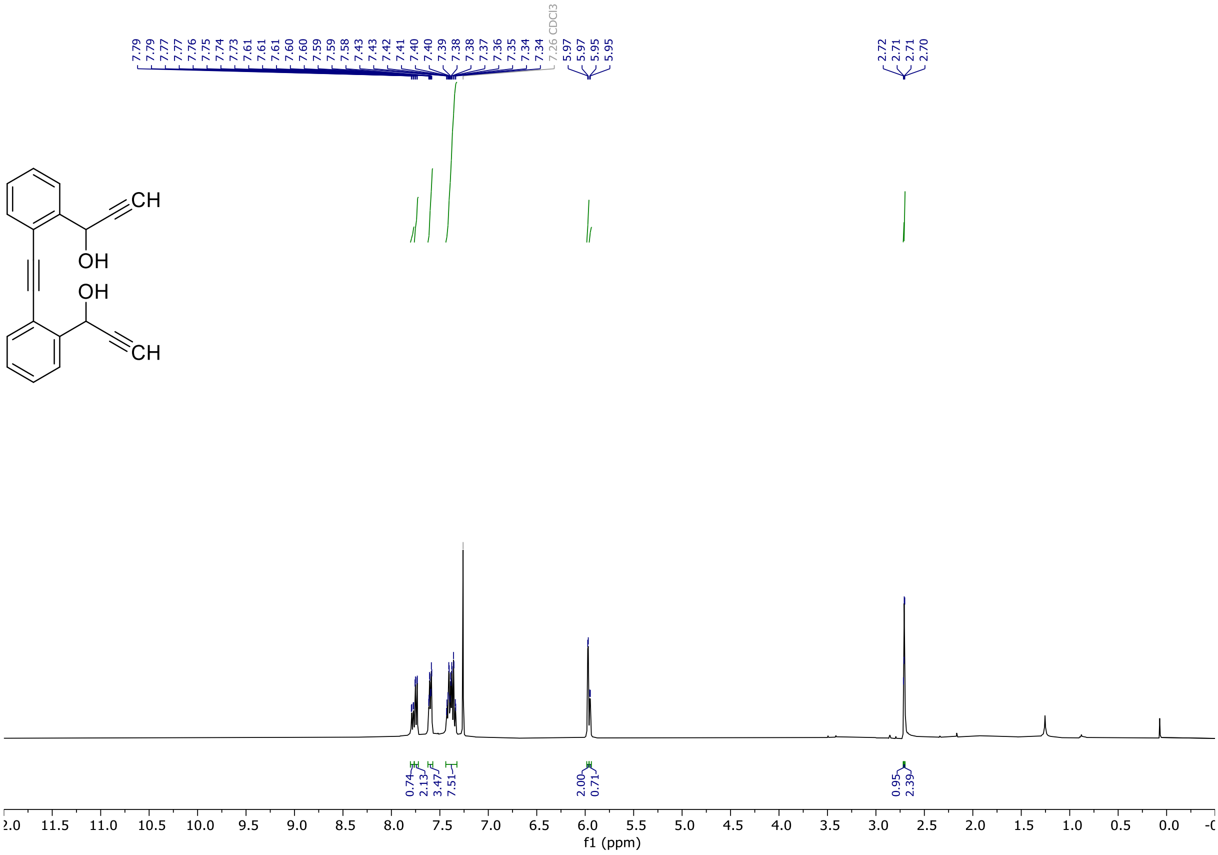

^{13}C NMR (101 MHz, $CDCl_3$, 25 °C)

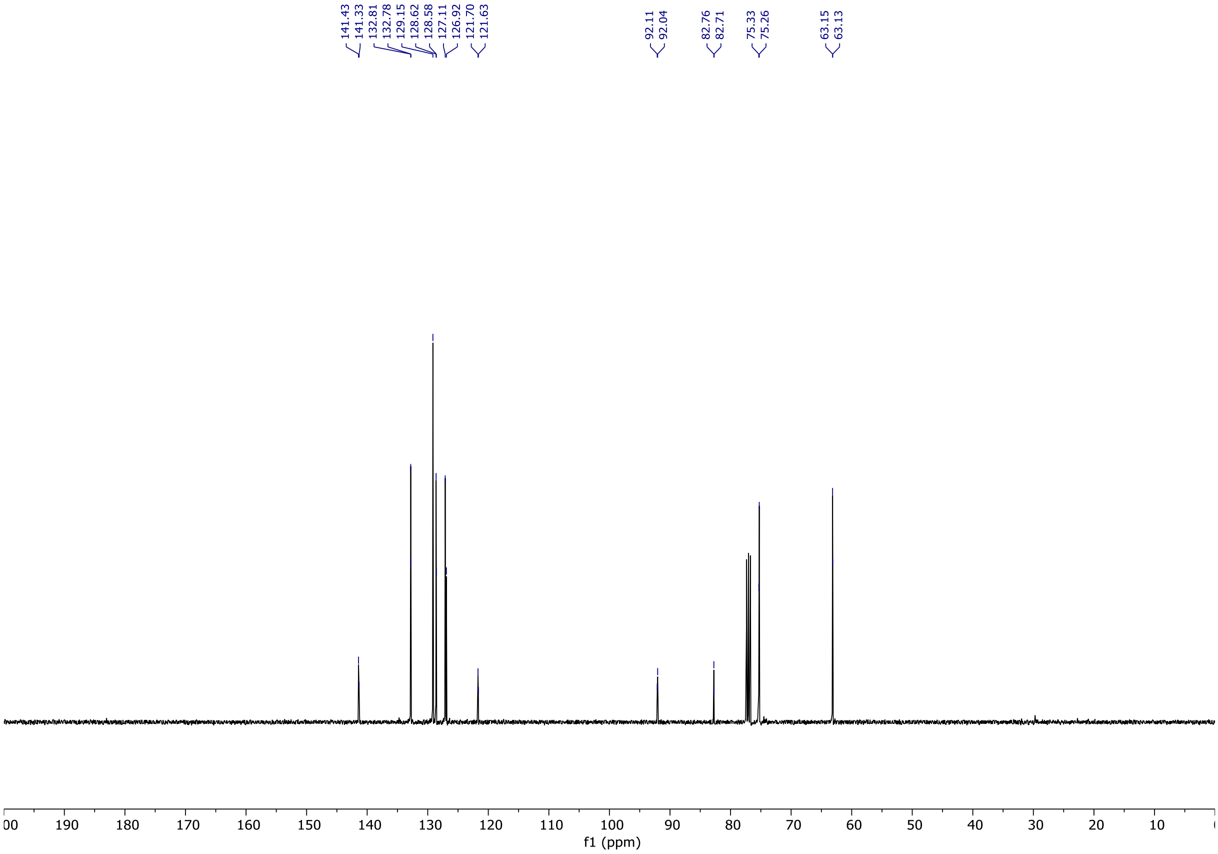

1,1'-(Ethyne-1,2-diylbis(4-methoxy-2,1-phenylene))bis(3-(*p*-tolyl)prop-2-yn-1-ol) (S3g)

^{1}H NMR (400 MHz, $CDCl_3$, 25 °C)

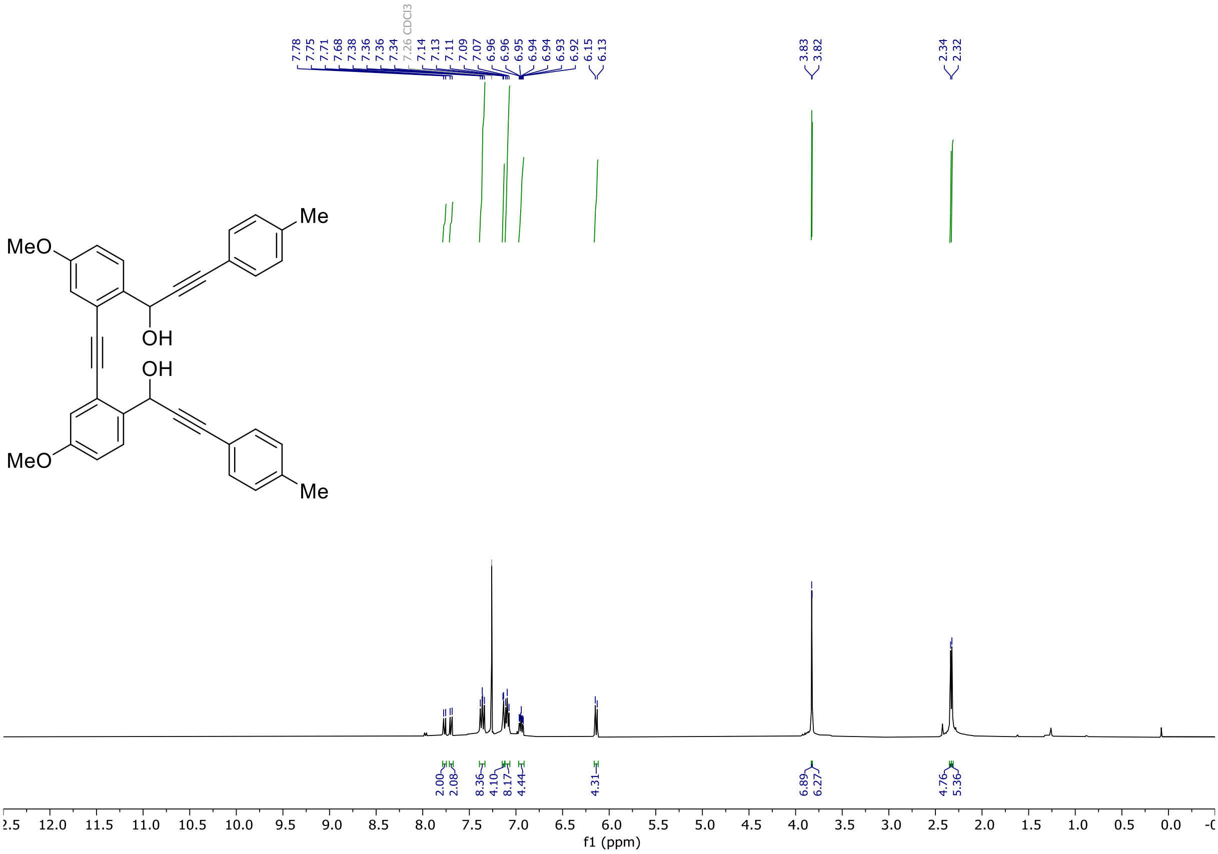

^{13}C NMR (101 MHz, $CDCl_3$, 25 °C)

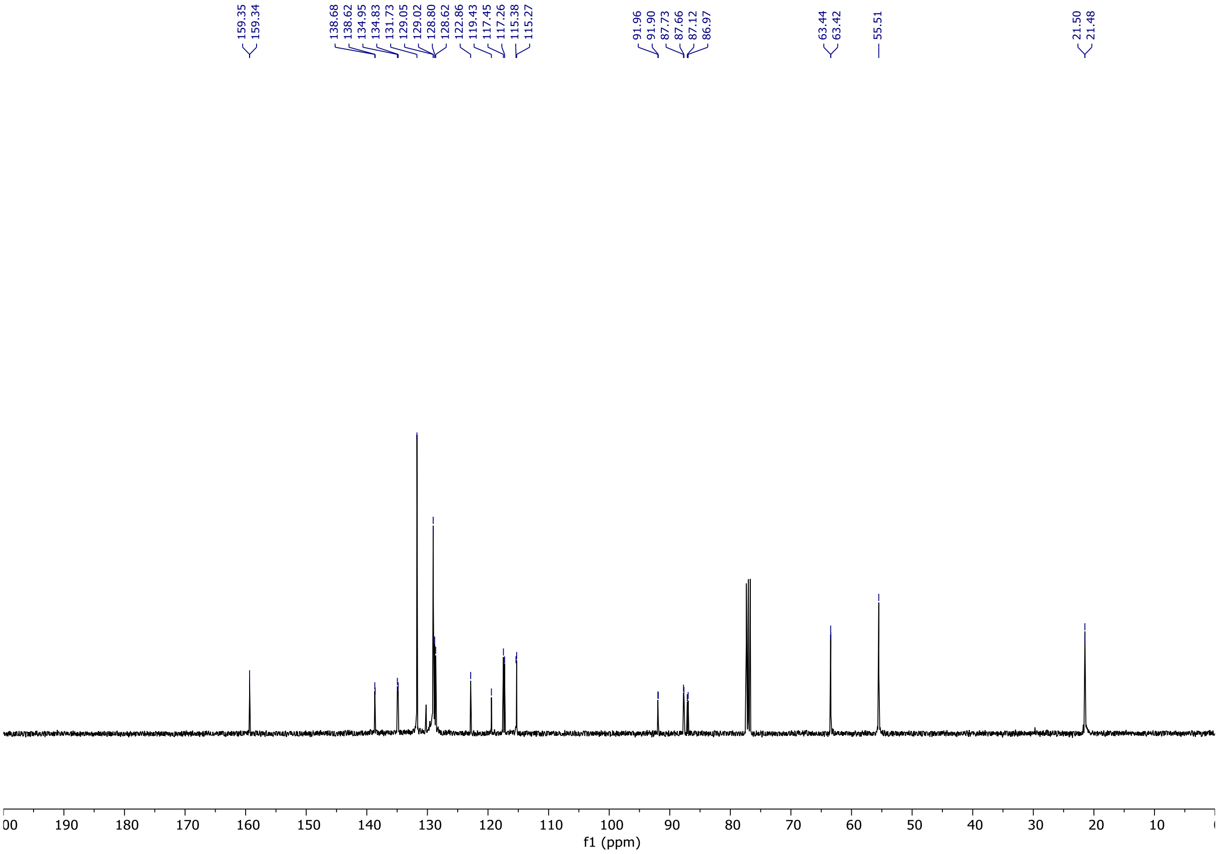

Indeno[2,1-*c*]fluorene-5,8-dione (S4f)

^{1}H NMR (400 MHz, $CDCl_3$, 25 °C)

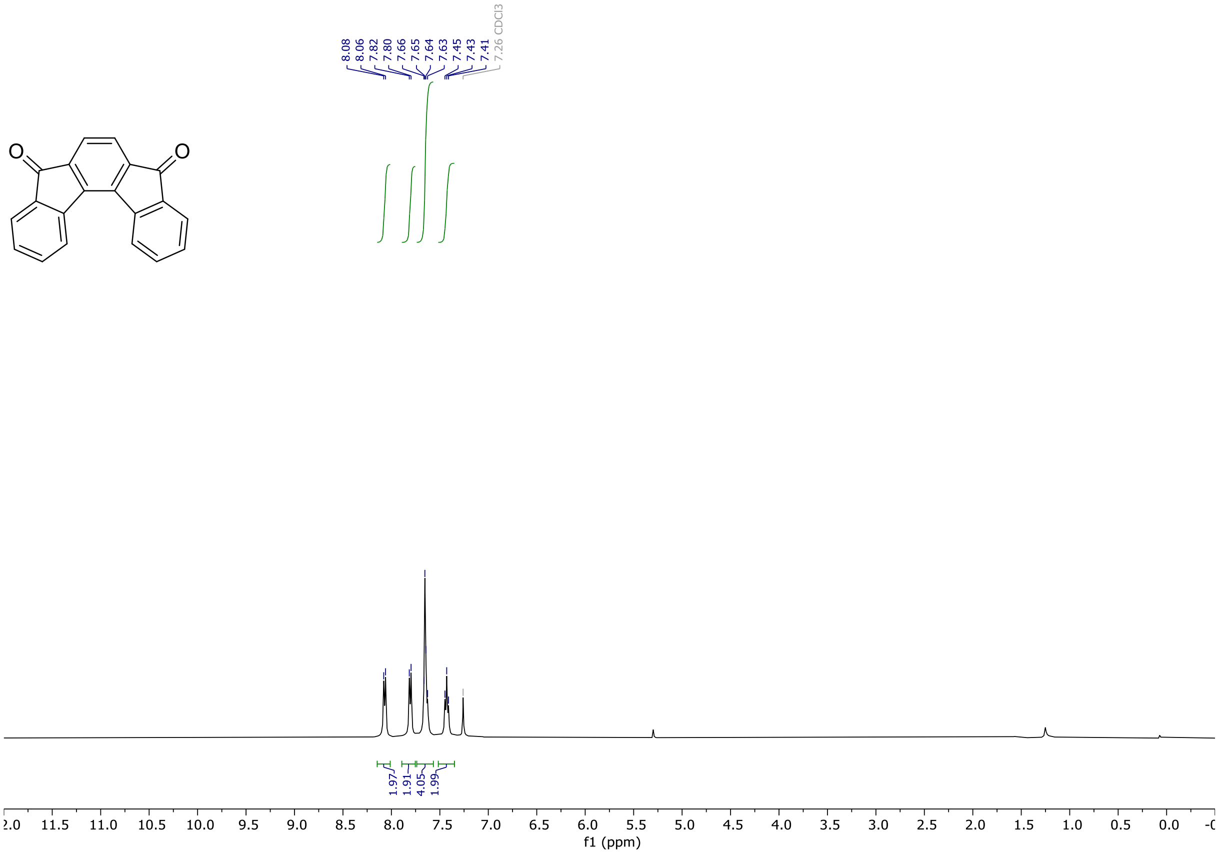

^{13}C NMR (101 MHz, $CDCl_3$, 25 °C)

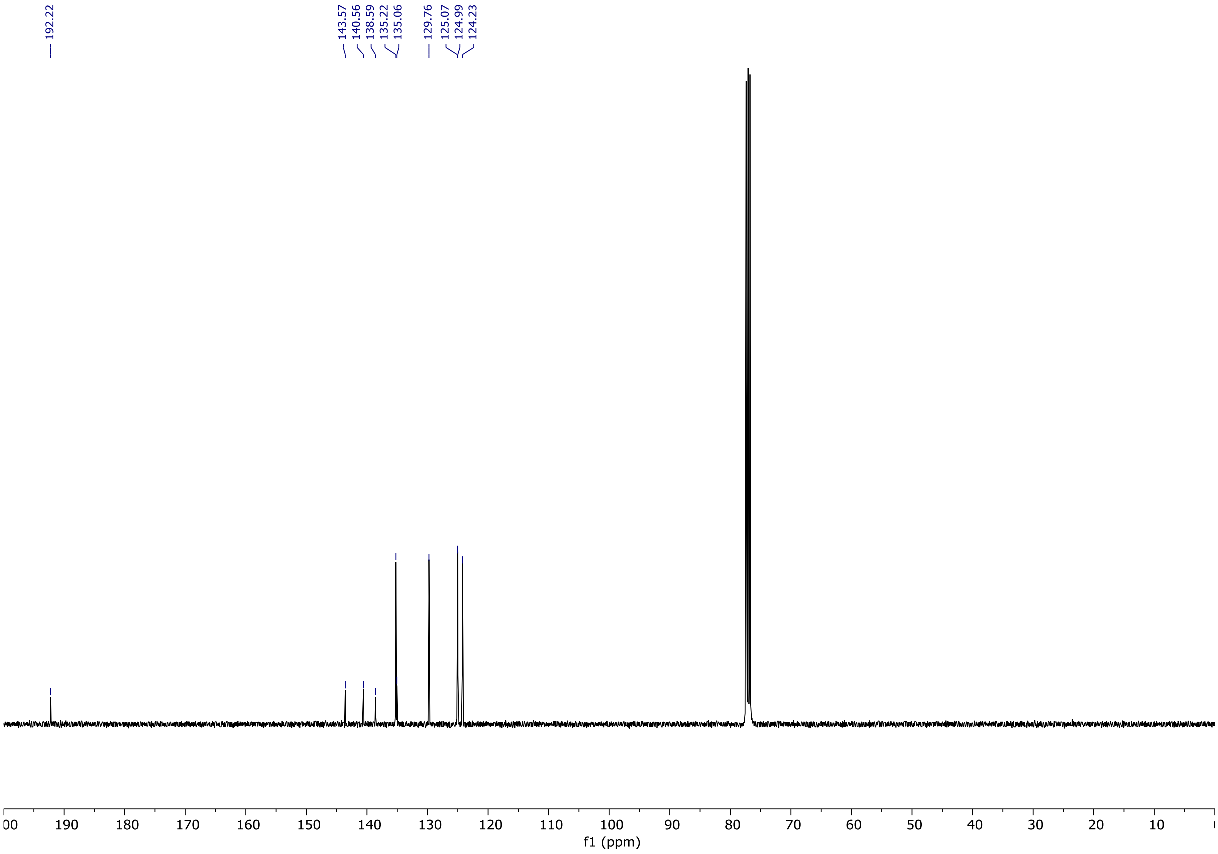

2,11-Dimethoxy-6,7-di-*p*-tolylindeno[2,1-*c*]fluorene-5,8-dione (S4g)

^{1}H NMR (400 MHz, $CDCl_3$, 25 °C)

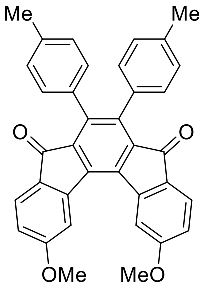

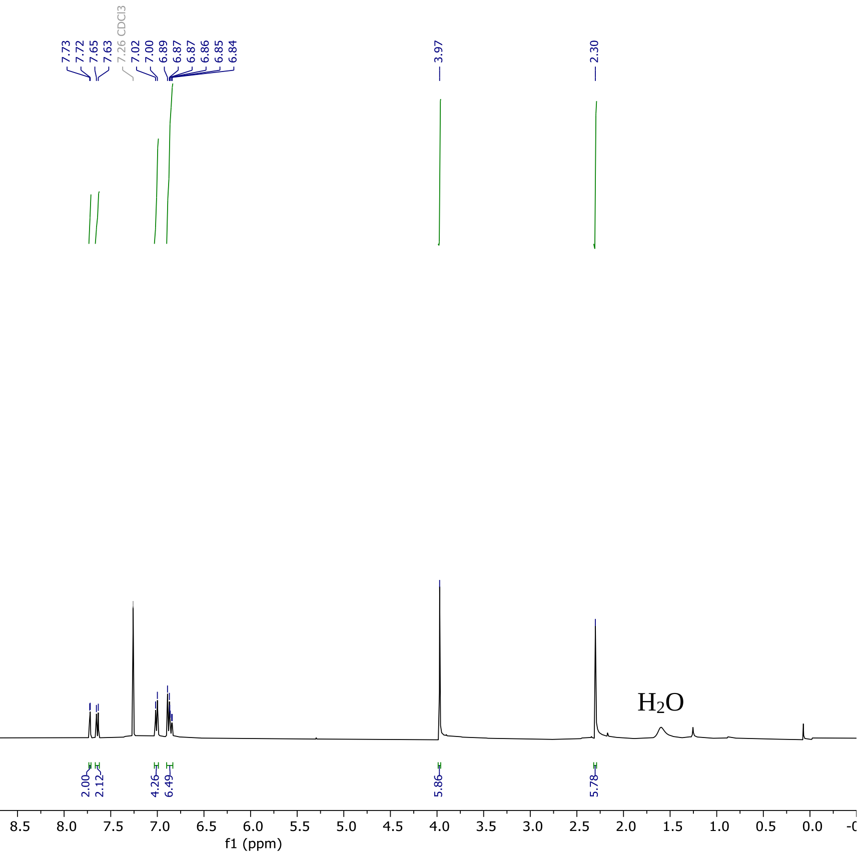

^{13}C NMR (101 MHz, $CDCl_3$, 25 °C)

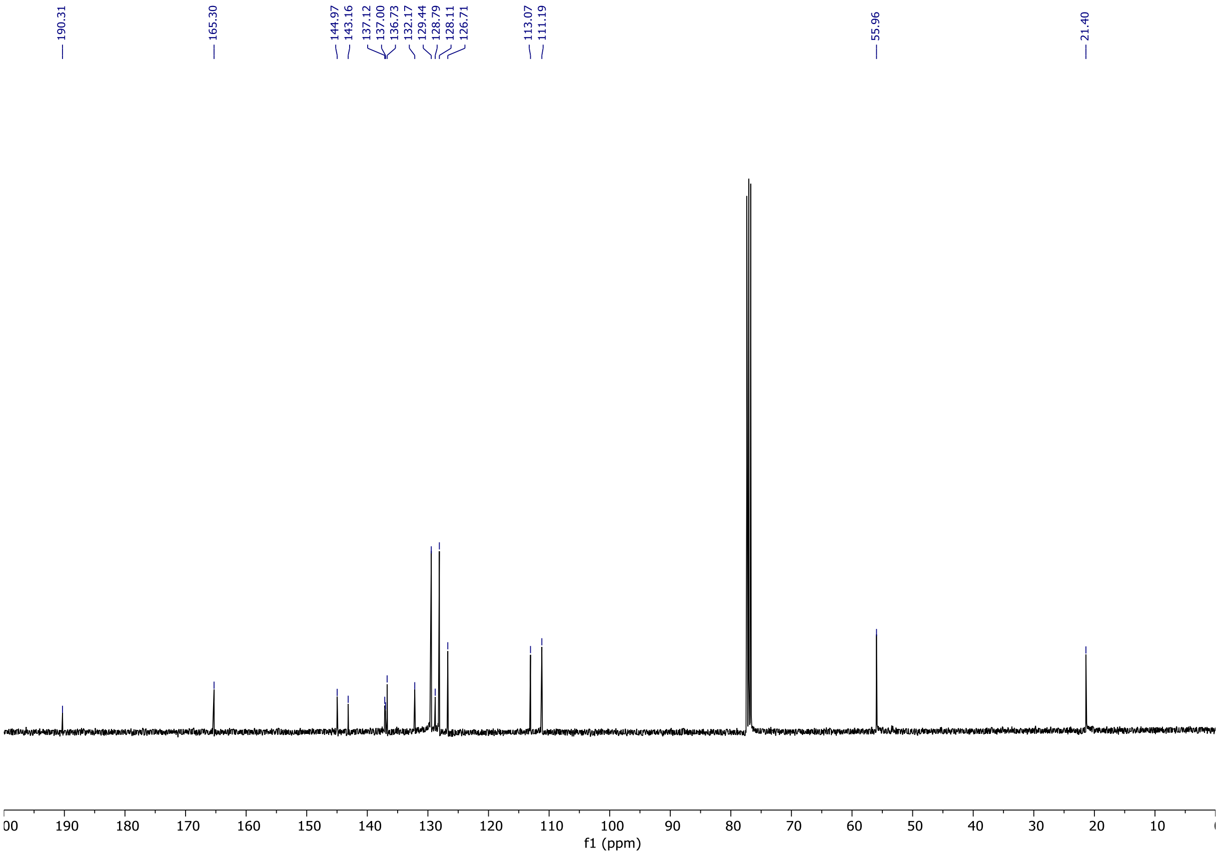

8,9-Bis(4-methoxyphenyl)benzo[*c*]benzo[6,7]indeno[1,2-*g*]fluorene-7,10-dione (S6)

^1H NMR (400 MHz, $CDCl_3$, 25 °C)

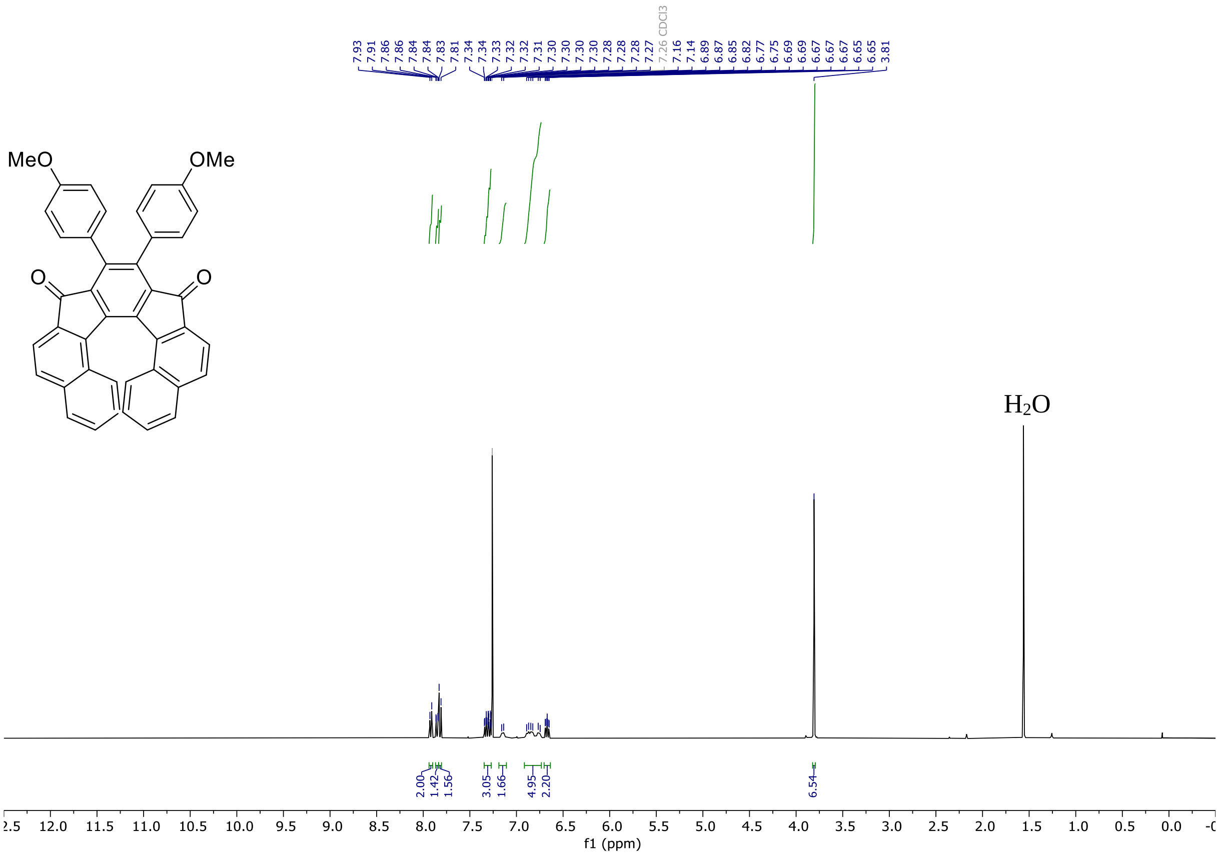

^{13}C NMR (101 MHz, $CDCl_3$, 25 °C)

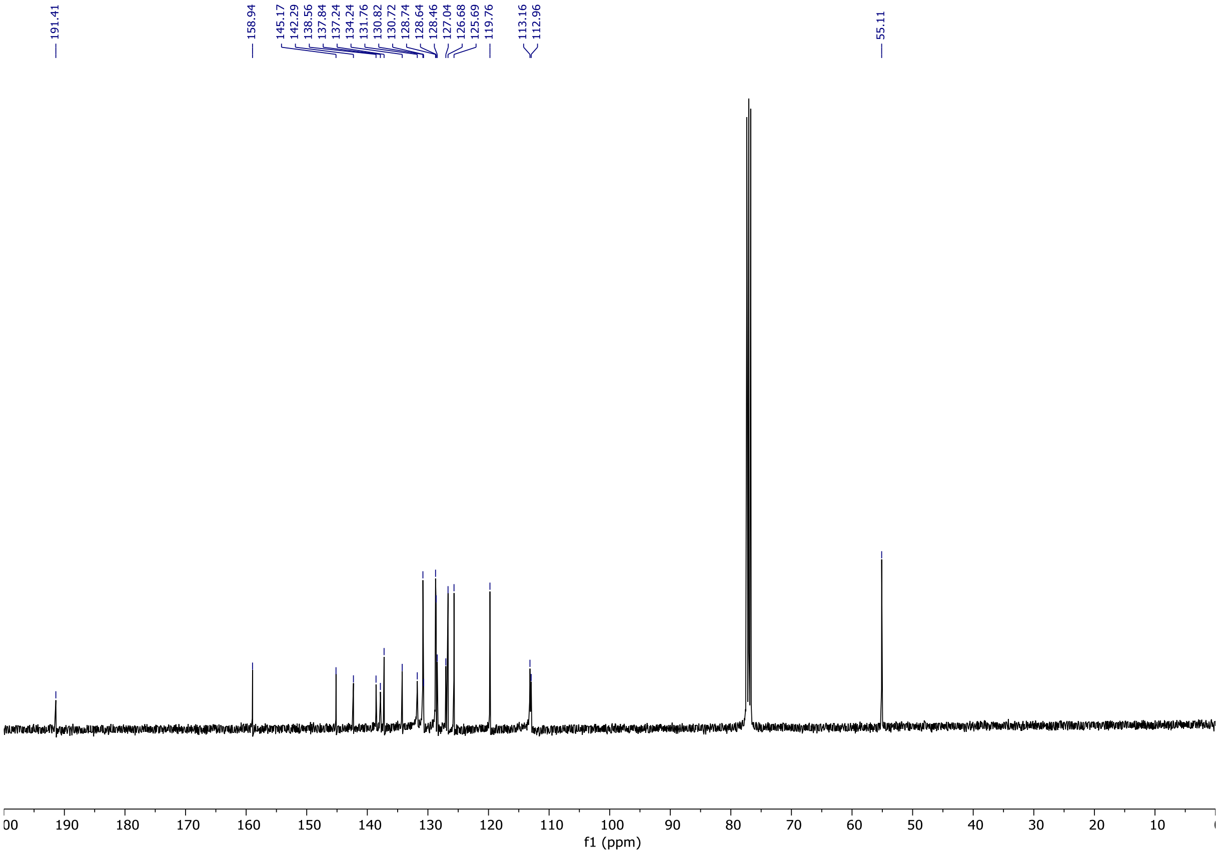

6,7-Bis(4-methoxyphenyl)-5,8-di-*p*-tolyl-5,8-dihydroindeno[2,1-*c*]fluorene-5,8-diol (1a)

^{1}H NMR (400 MHz, $CDCl_3$, 25 °C)

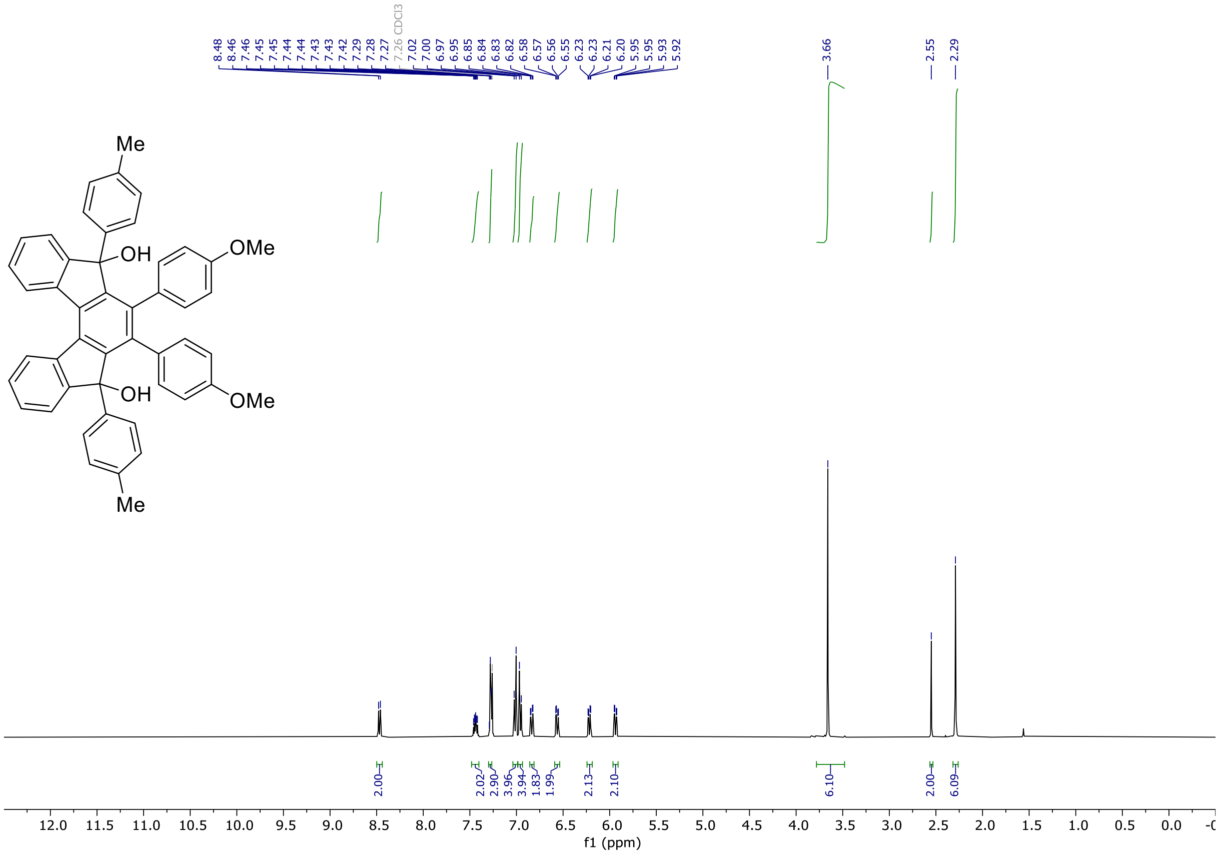

^{13}C NMR (101 MHz, $CDCl_3$, 25 °C)

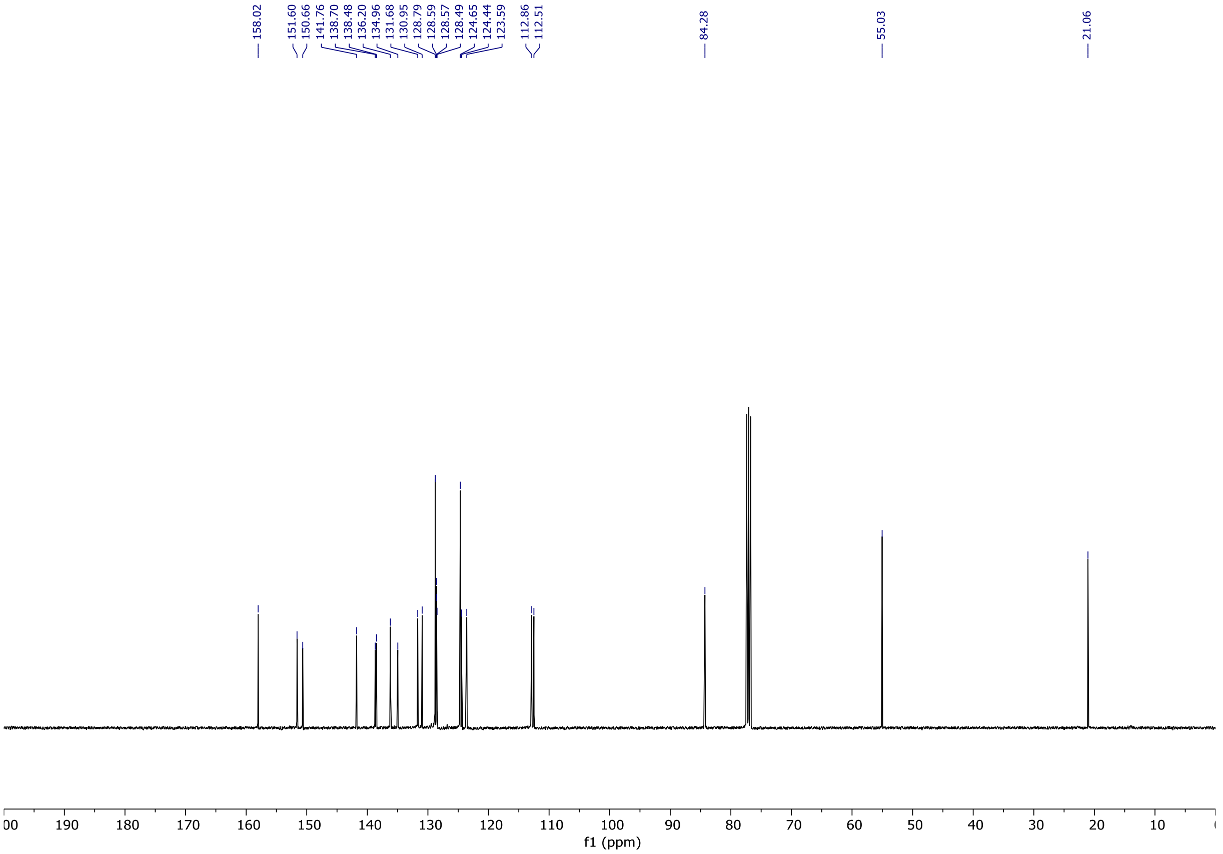

6,7-Bis(4-methoxyphenyl)-5,8-di-*o*-tolyl-5,8-dihydroindeno[2,1-*c*]fluorene-5,8-diol (1b)

^{1}H NMR (400 MHz, $CDCl_3$, 25 °C)

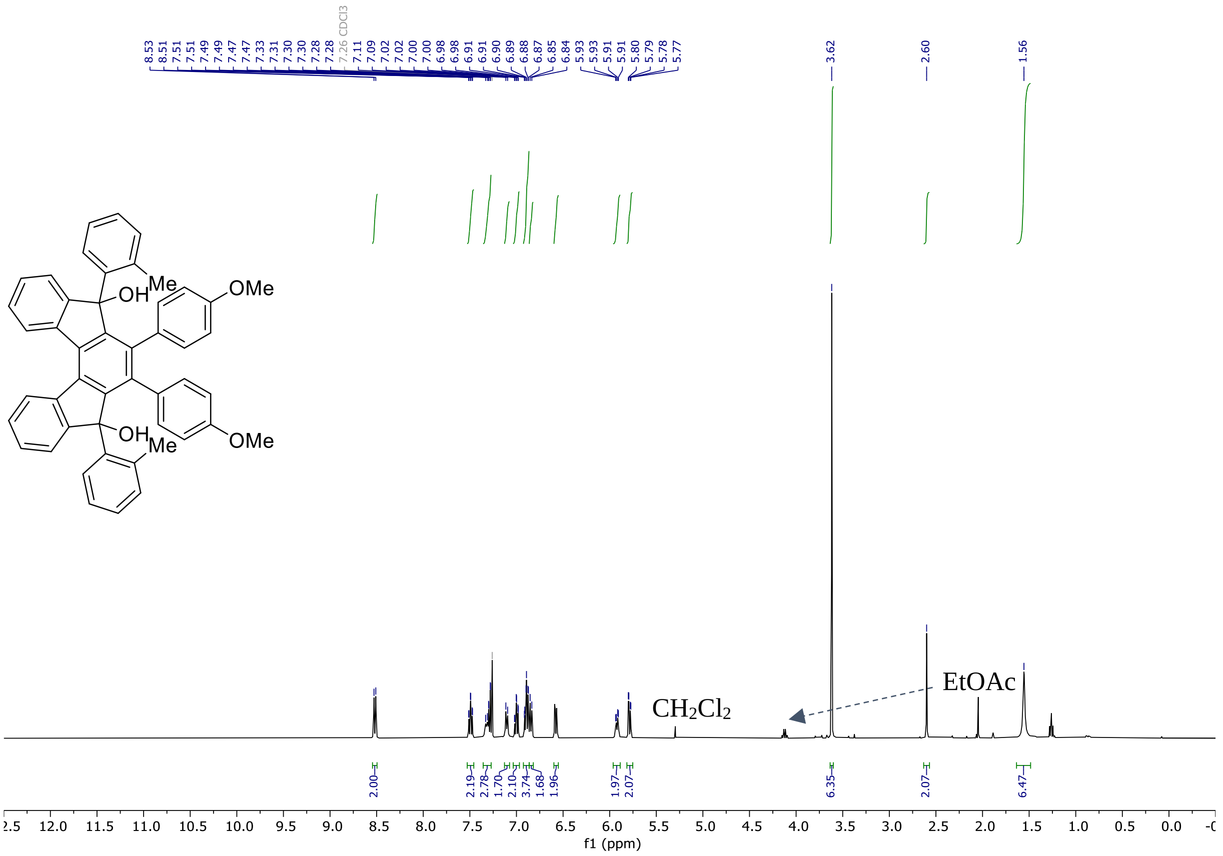

^{13}C NMR (101 MHz, $CDCl_3$, 25 °C)

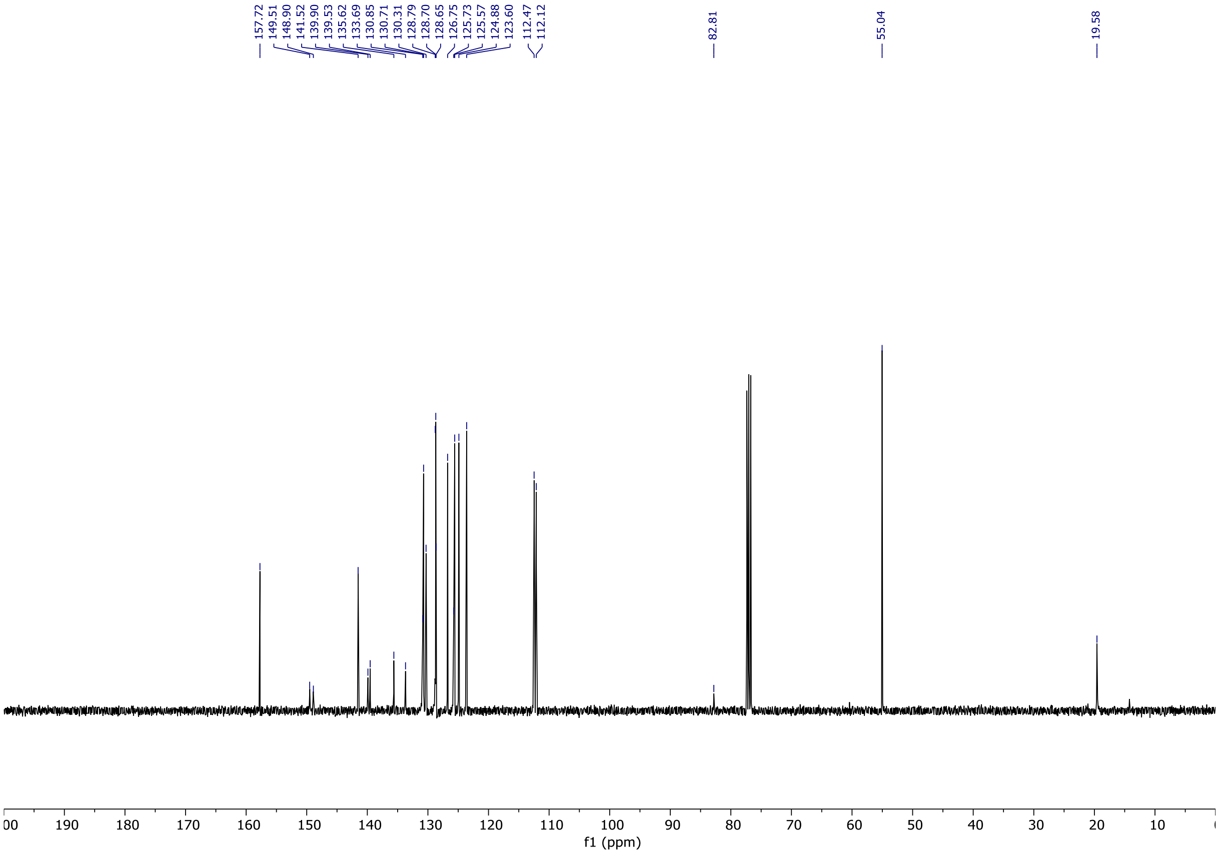

5,8-Dibutyl-6,7-bis(4-methoxyphenyl)-5,8-dihydroindeno[2,1-*c*]fluorene-5,8-diol (1c)

^{1}H NMR (400 MHz, $CDCl_3$, 25 °C)

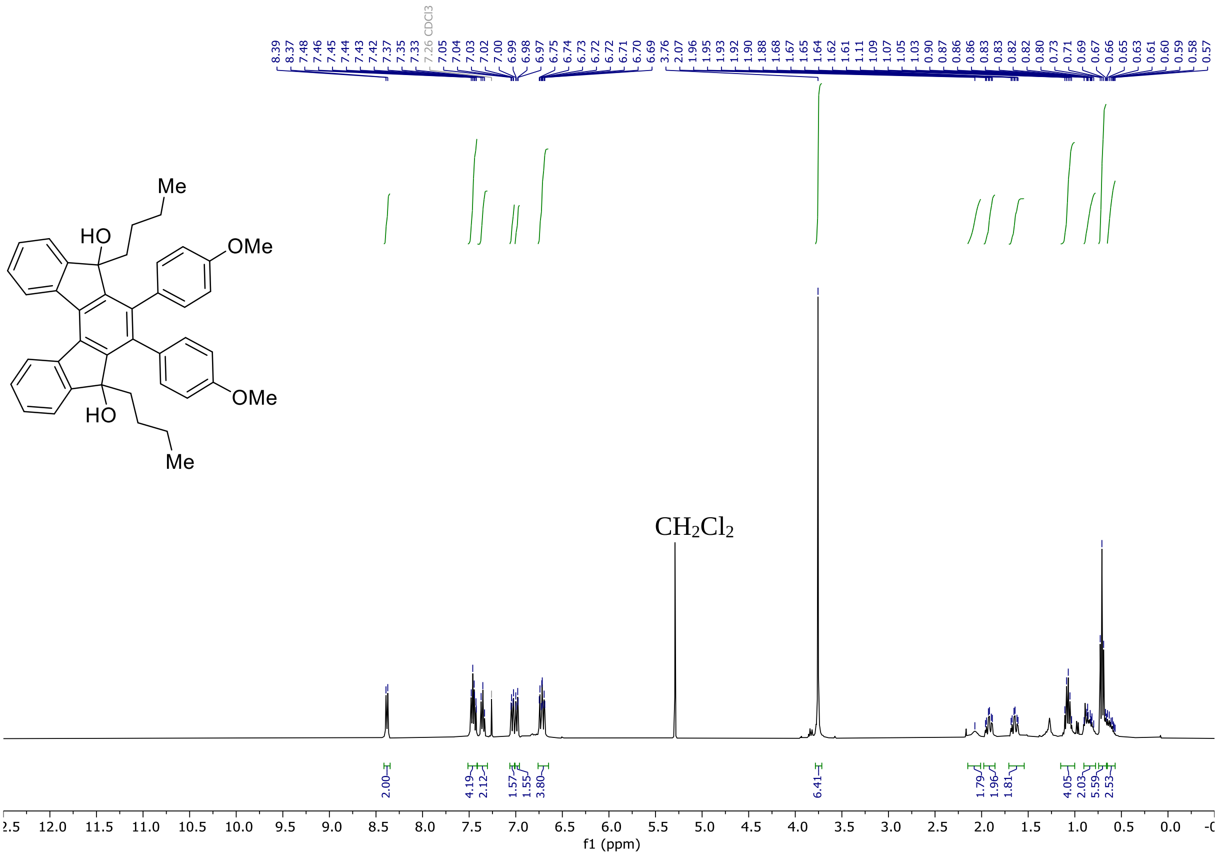

^{13}C NMR (101 MHz, $CDCl_3$, 25 °C)

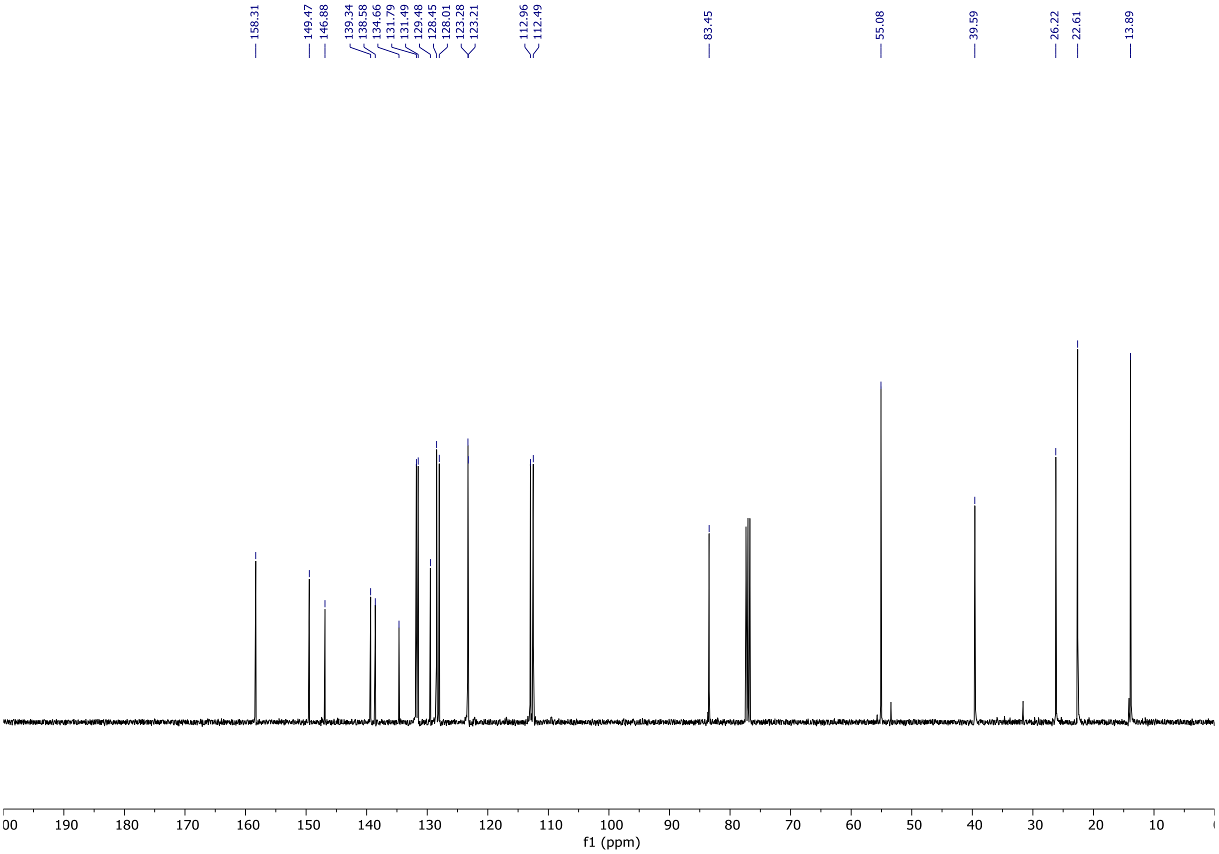

5,8-Dibutyl-6,7-bis(4-methoxyphenyl)-5,8-dihydroindeno[2,1-*c*]fluorene-5,8-diol (1c)

^{1}H NMR (400 MHz, $CDCl_3$, 25 °C)

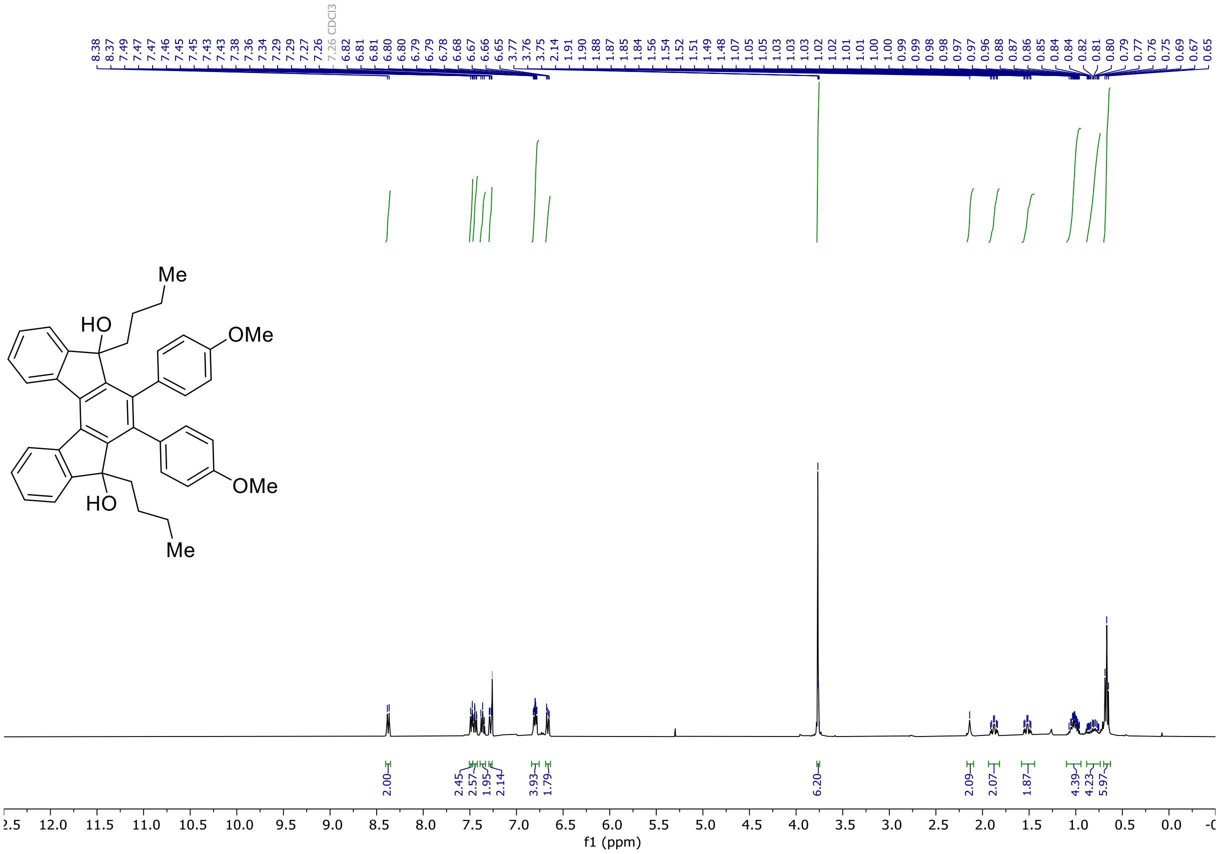

^{13}C NMR (101 MHz, $CDCl_3$, 25 °C)

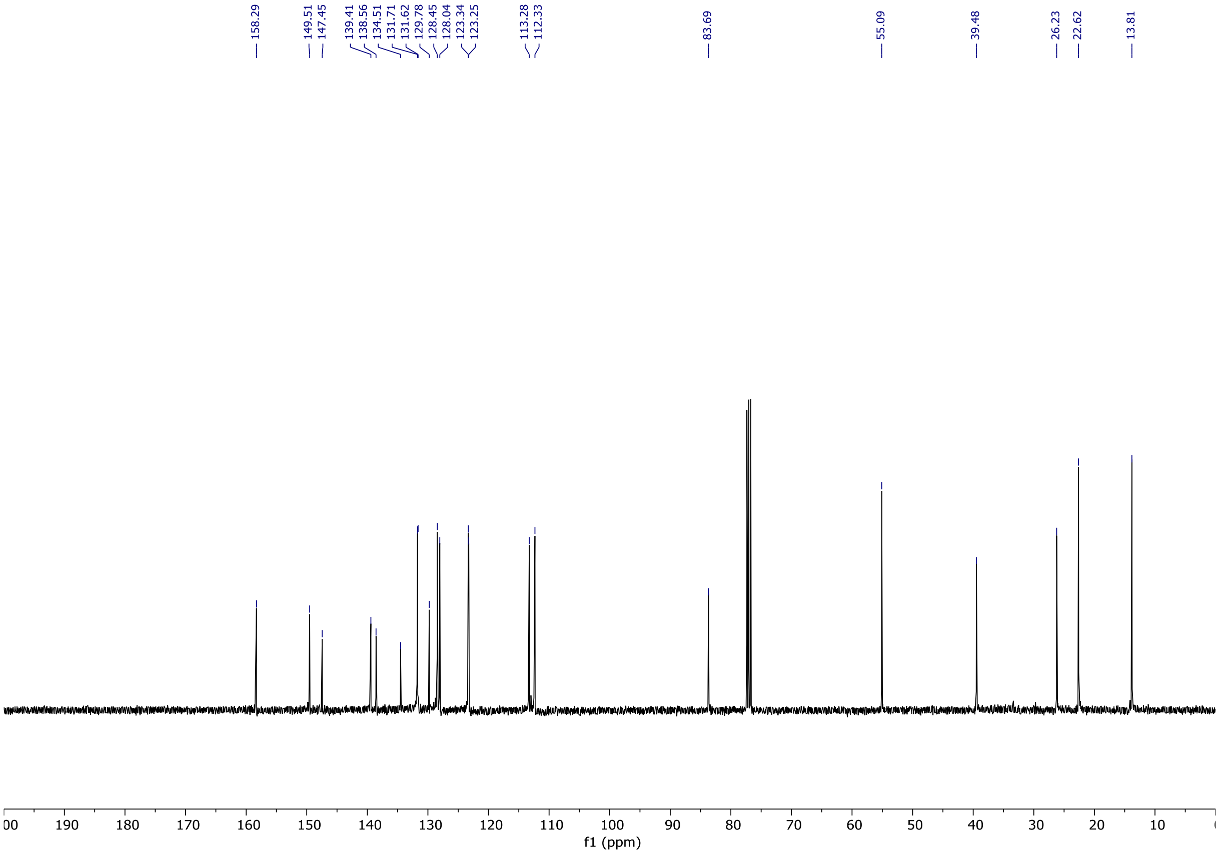

6,7-Bis(4-methoxyphenyl)-5,8-bis(4-(trifluoromethyl)phenyl)-5,8-dihydroindeno[2,1-*c*]-fluorene-5,8-diol (1d)

^{1}H NMR (400 MHz, $CDCl_3$, 25 °C)

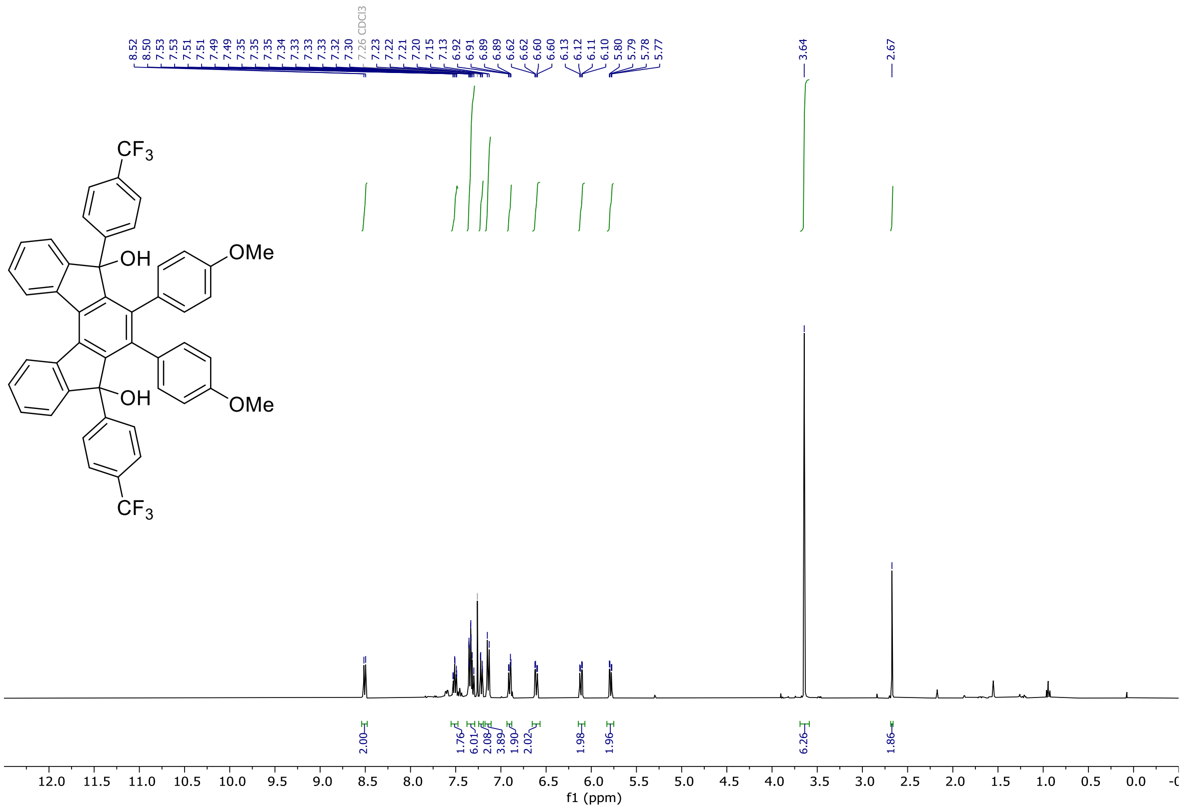

^{13}C NMR (101 MHz, $CDCl_3$, 25 °C)

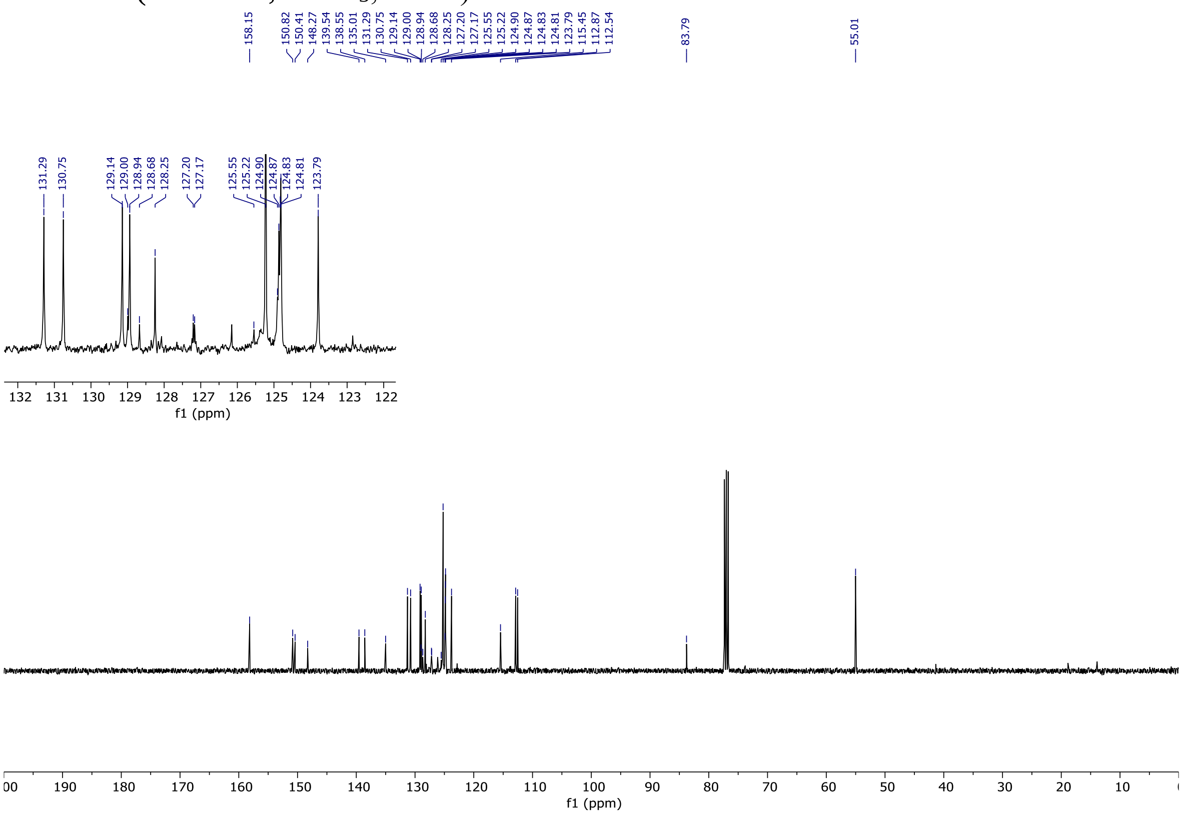

^{19}F NMR (376.5 MHz, $CDCl_3$, 25 °C)

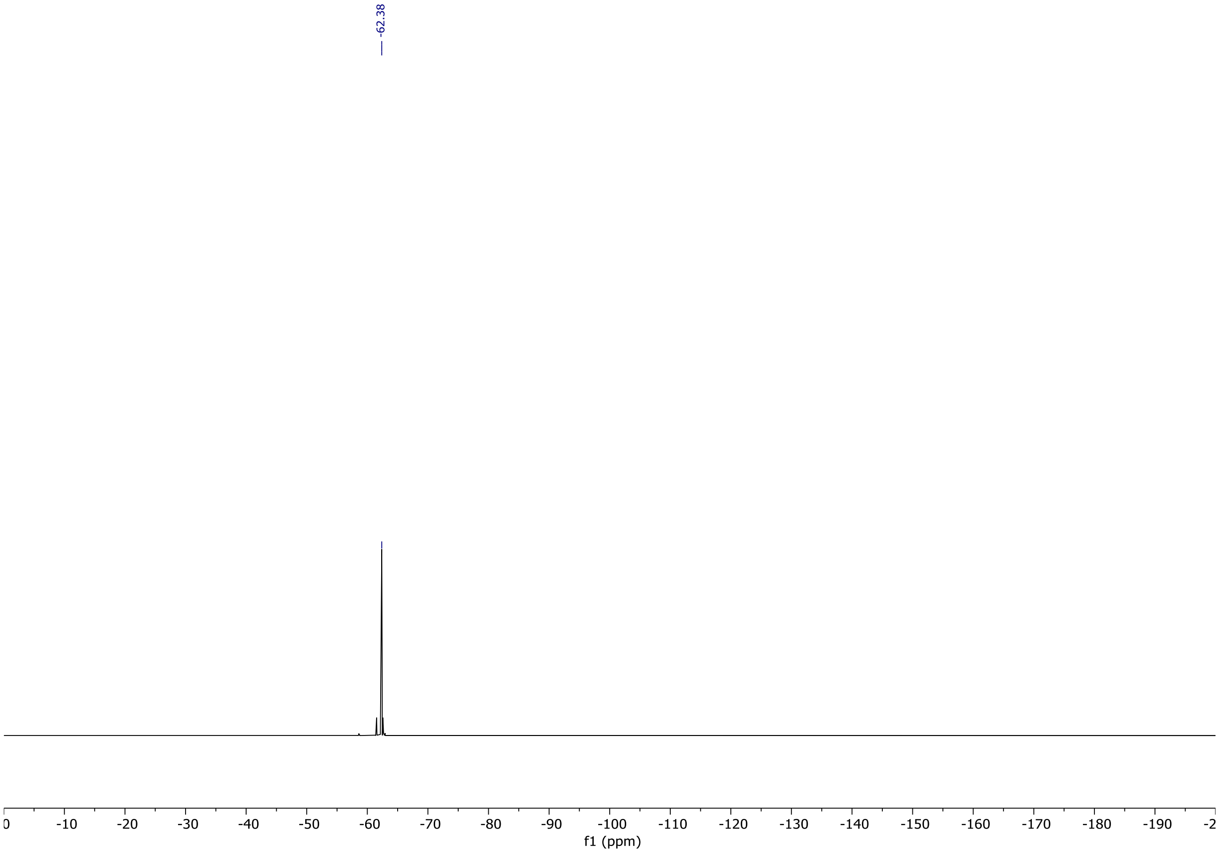

5,8-Di-*p*-tolyl-6,7-bis(4-(trifluoromethyl)phenyl)-5,8-dihydroindeno[2,1-*c*]fluorene-5,8-diol (1e)

^{1}H NMR (400 MHz, $CDCl_3$, 25 °C)

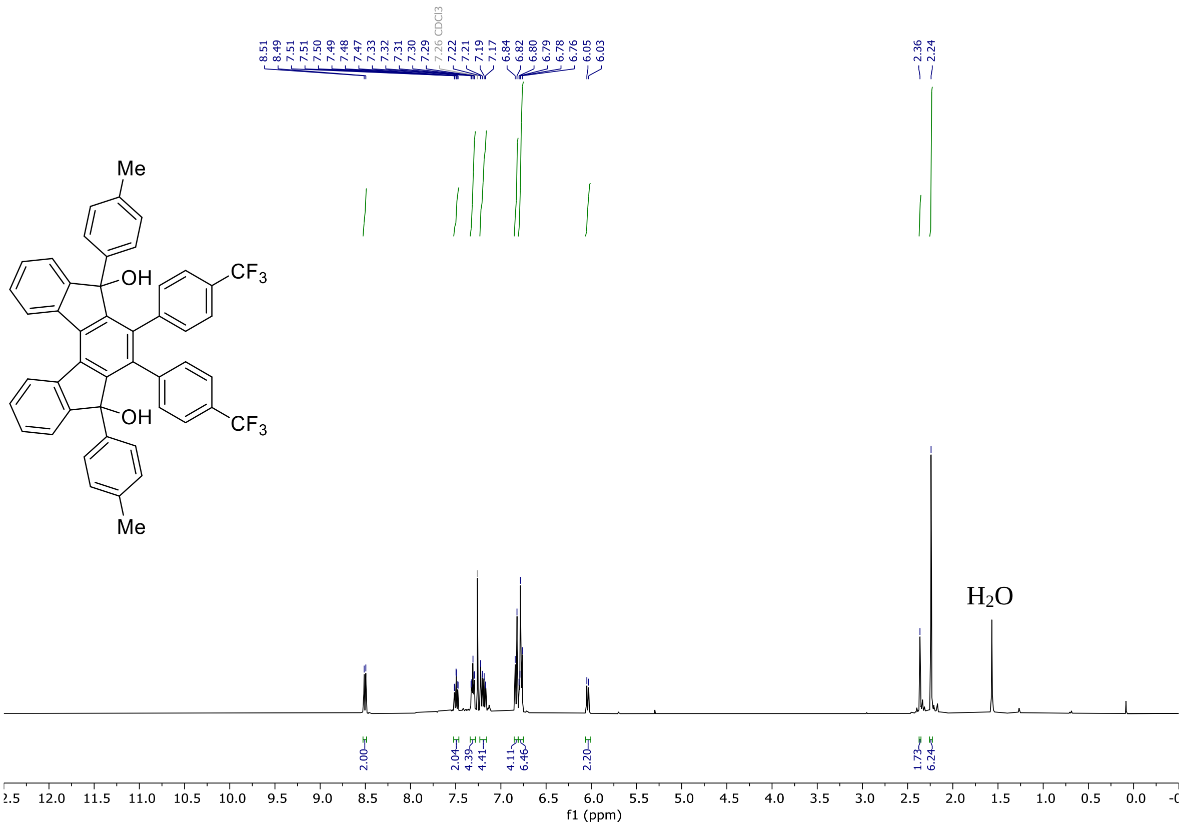

^{13}C NMR (101 MHz, $CDCl_3$, 25 °C)

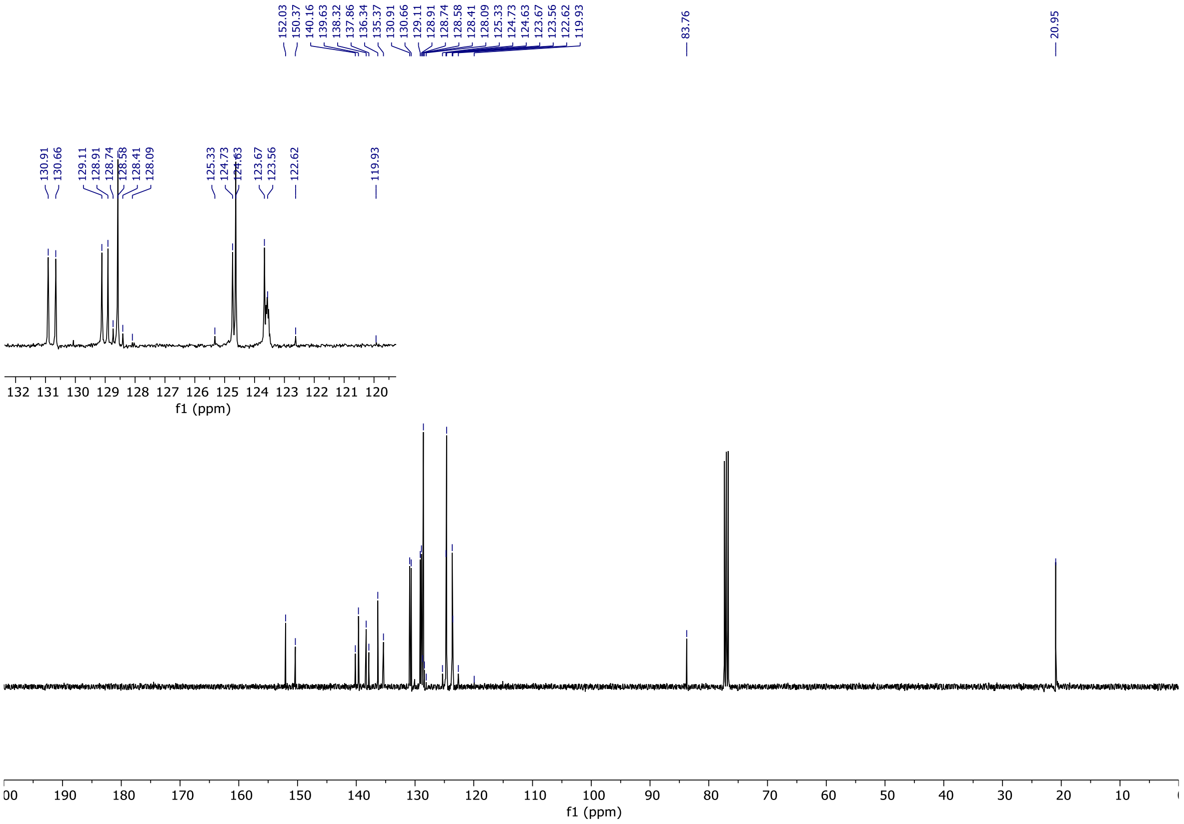

^{19}F NMR (376.5 MHz, $CDCl_3$, 25 °C)

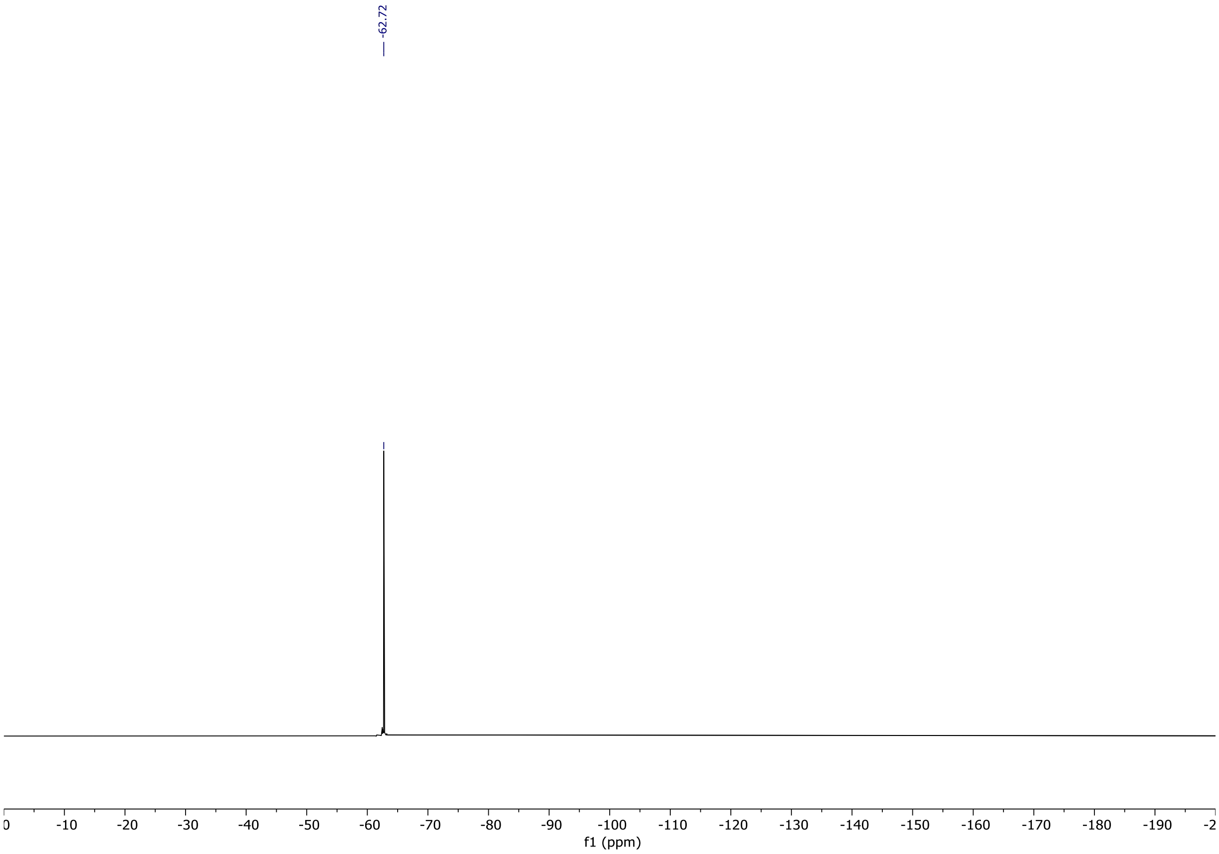

5,8-Di-*p*-tolyl-5,8-dihydroindeno[2,1-*c*]fluorene-5,8-diol (1f)

^{1}H NMR (400 MHz, $CDCl_3$, 25 °C)

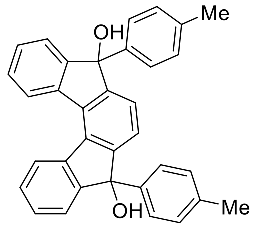

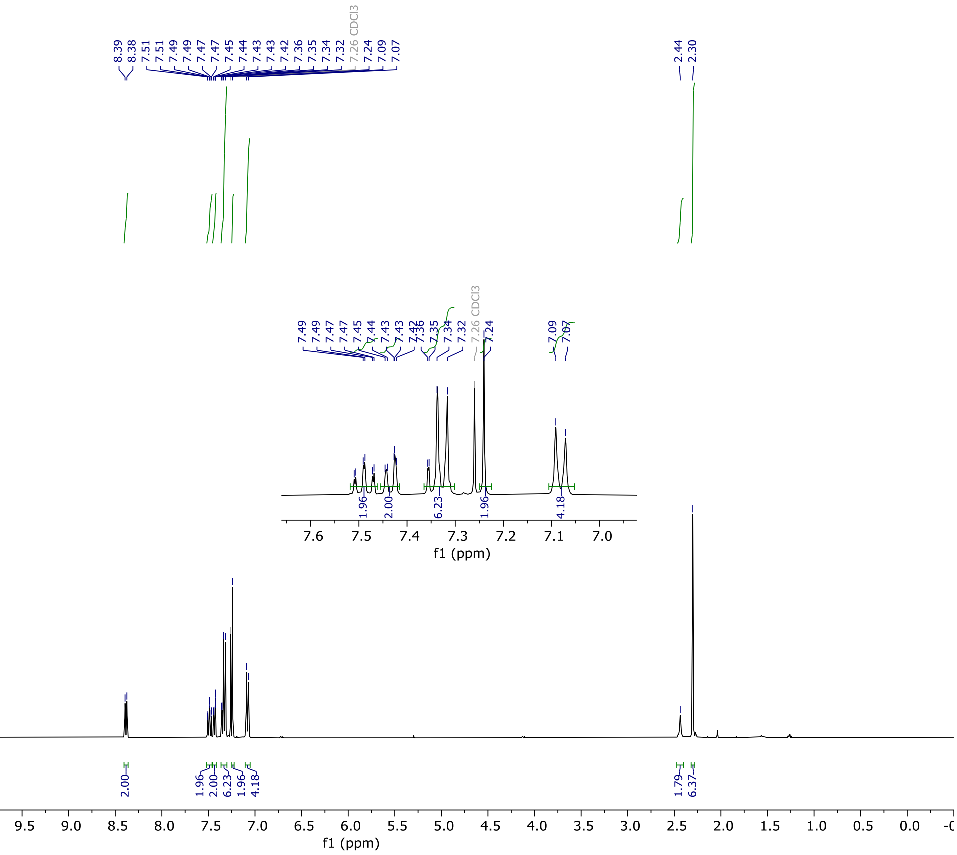

^{13}C NMR (101 MHz, $CDCl_3$, 25 °C)

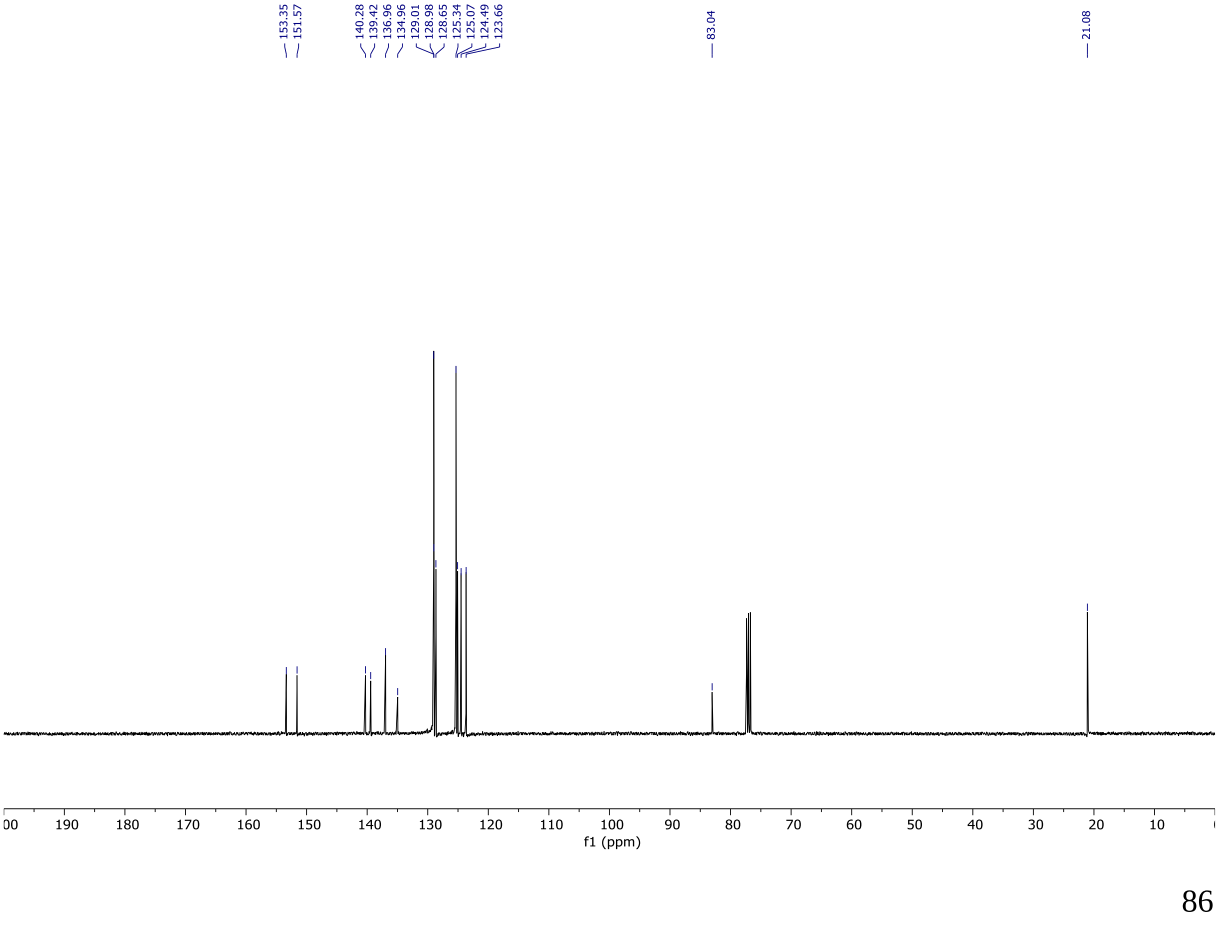

5,8-Di-*p*-tolyl-5,8-dihydroindeno[2,1-*c*]fluorene-5,8-diol (1f)

^{1}H NMR (400 MHz, $CDCl_3$, 25 °C)

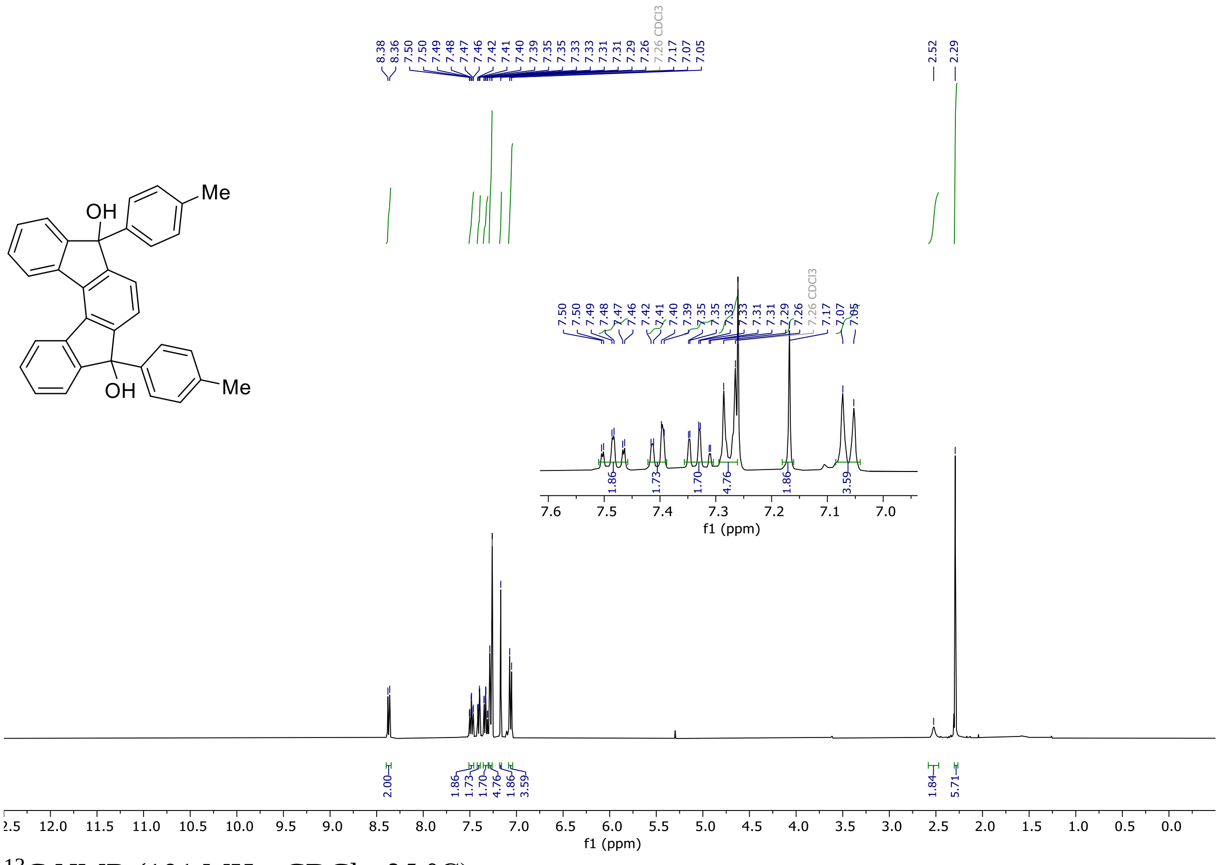

^{13}C NMR (101 MHz, $CDCl_3$, 25 °C)

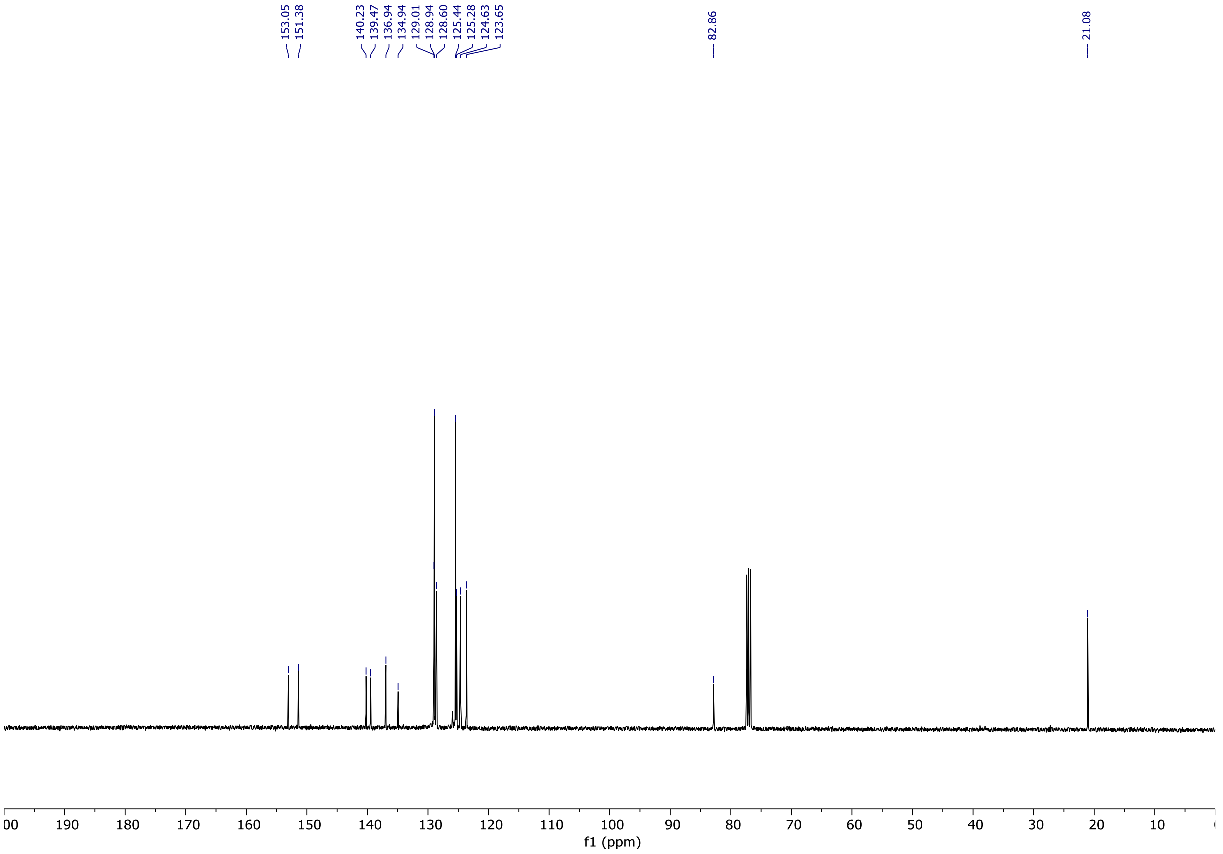

2,11-dimethoxy-5,8-diphenyl-6,7-di-*p*-tolyl-5,8-dihydroindeno[2,1-*c*]fluorene-5,8-diol (1g)

^{1}H NMR (400 MHz, $CDCl_3$, 25 °C)

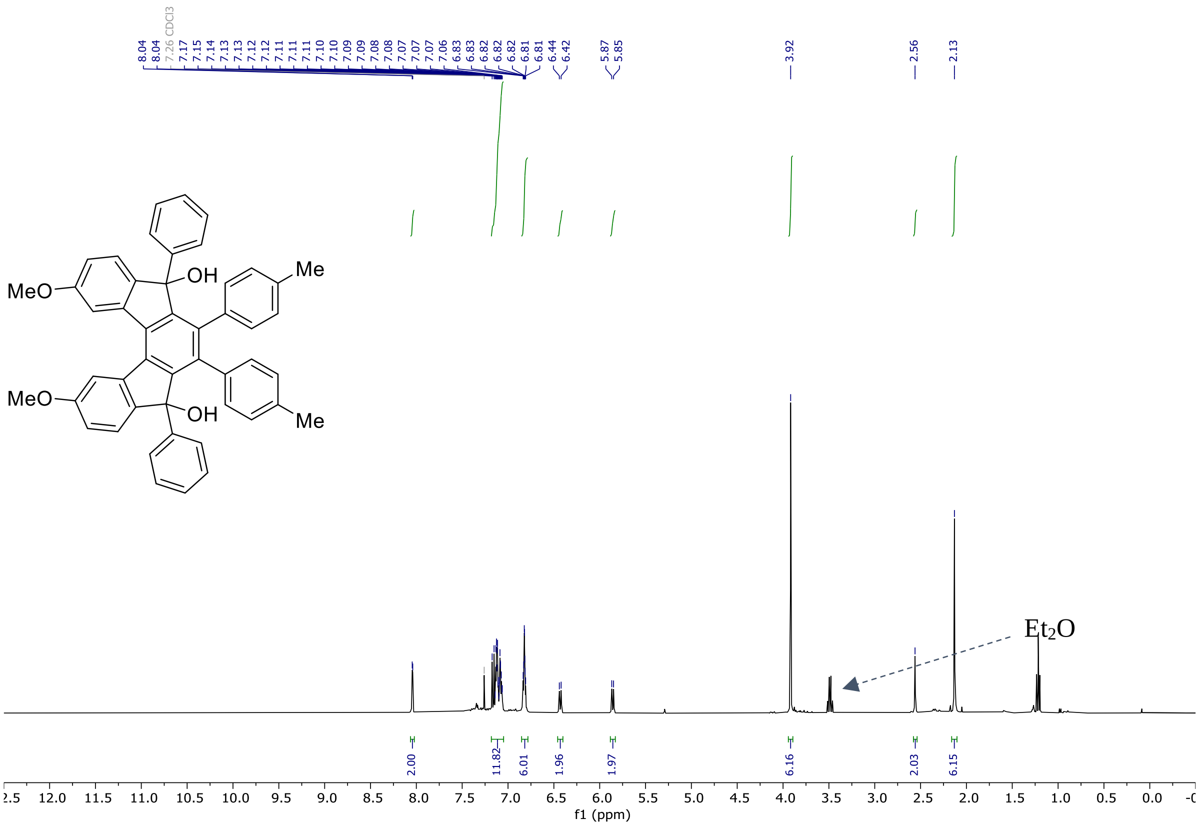

^{13}C NMR (101 MHz, $CDCl_3$, 25 °C)

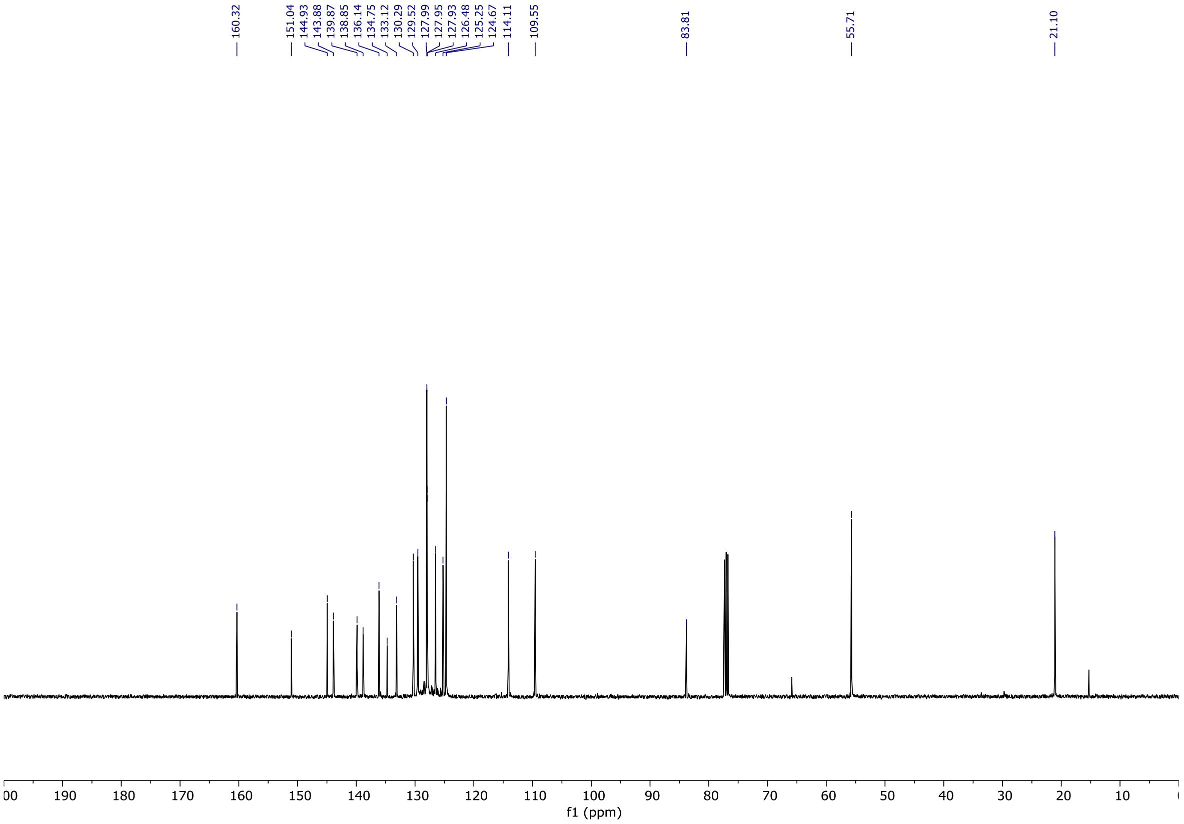

8,9-Bis(4-methoxyphenyl)-7,10-di-*p*-tolyl-7,10-dihydrobenzo[c]benzo[6,7]indeno[1,2-*g*]-fluorene-7,10-diol (8)

^{1}H NMR (400 MHz, CDCl$_3$, 25 °C)

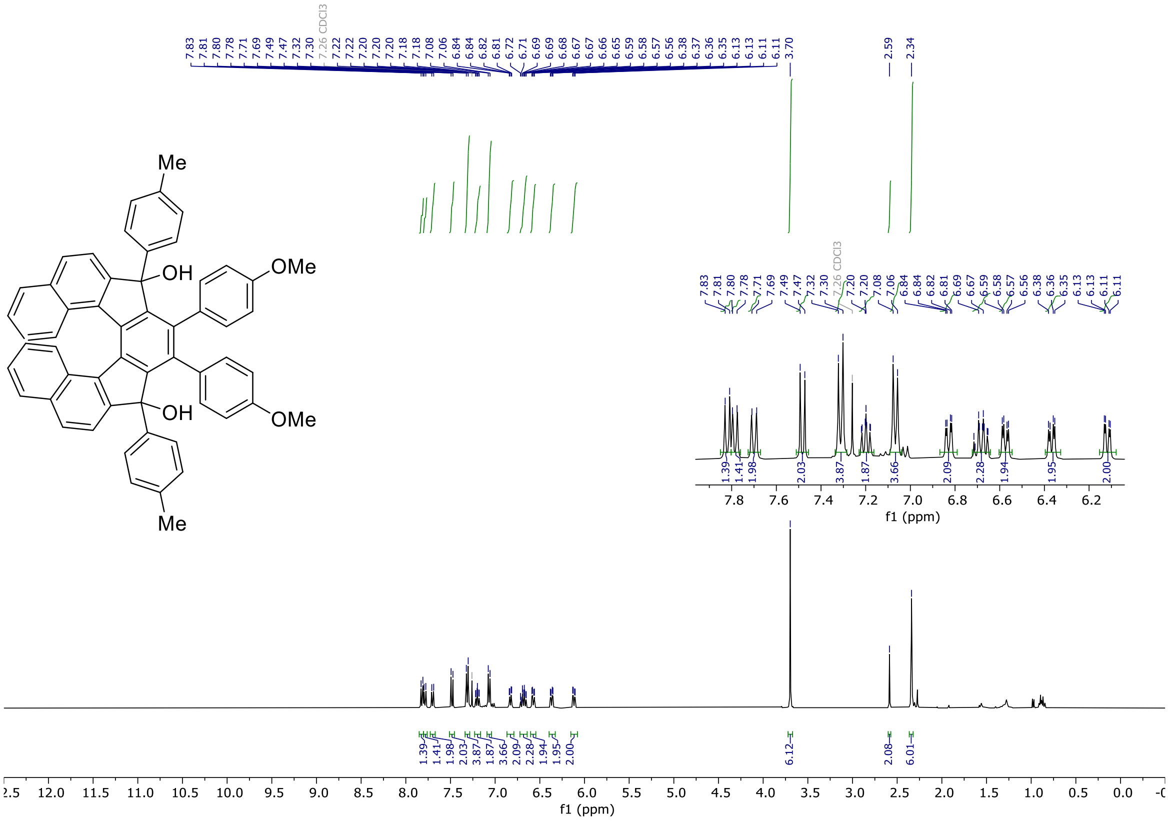

^{13}C NMR (101 MHz, CDCl$_3$, 25 °C)

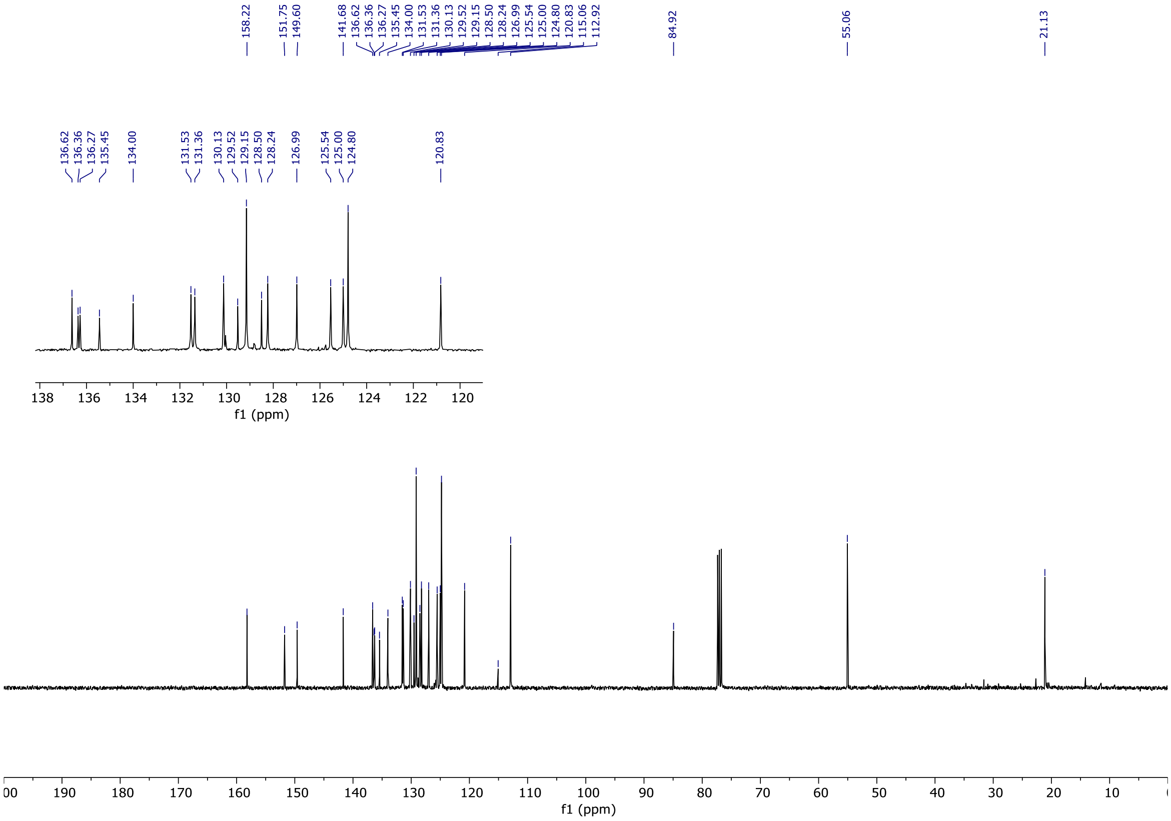

9-Azido-7,8-bis(4-methoxyphenyl)-5,9-di-p-tolyl-9H-indeno[1,2-*a*]phenanthridine (2a)

^{1}H NMR (400 MHz, $CDCl_3$, 25 °C)

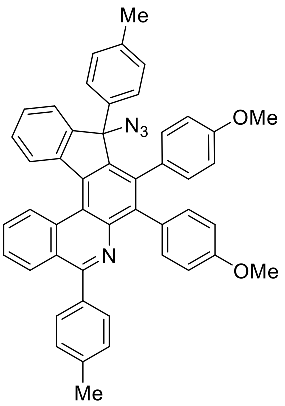

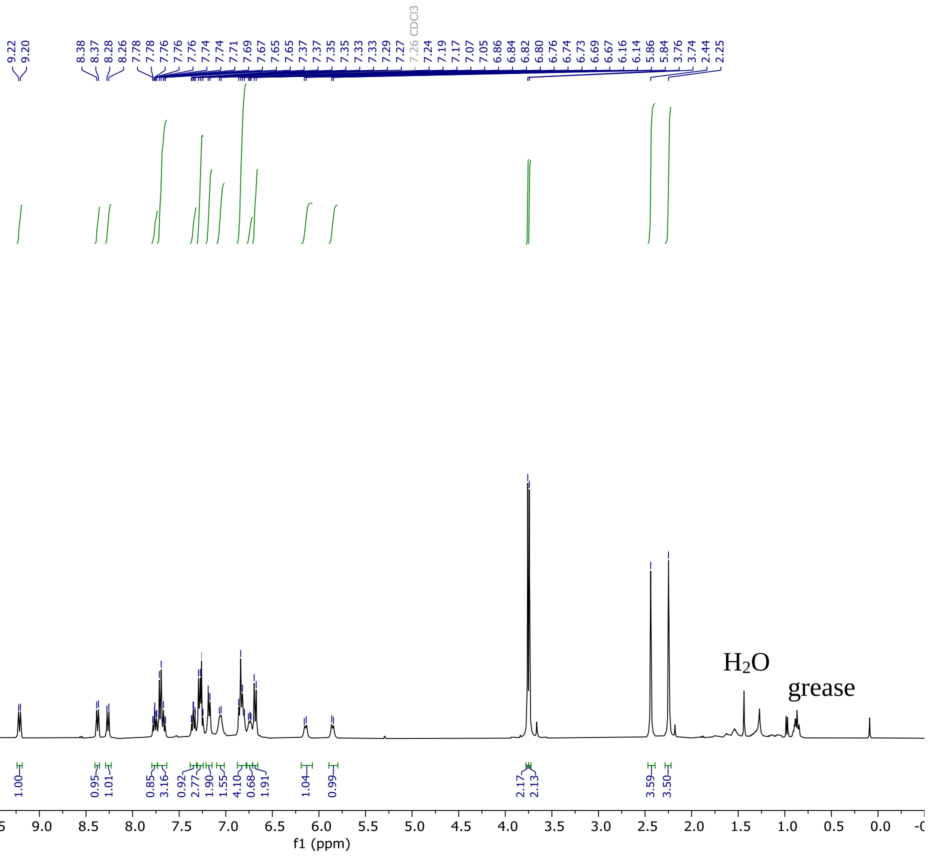

^{13}C NMR (101 MHz, $CDCl_3$, 25 °C)

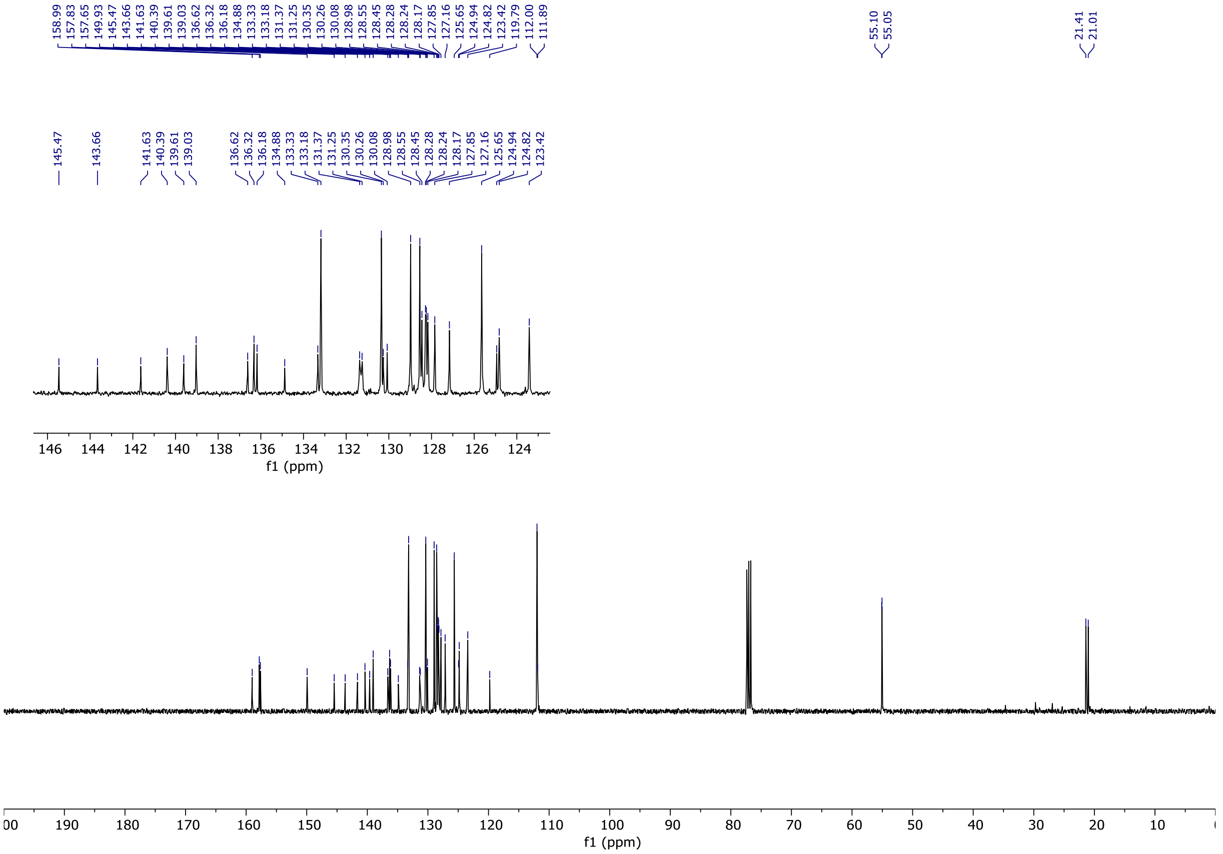

9-Azido-7,8-bis(4-methoxyphenyl)-6,9-di-p-tolyl-9H-indeno[2,1-*k*]phenanthridine (3a)

^{1}H NMR (400 MHz, $CDCl_3$, 25 °C)

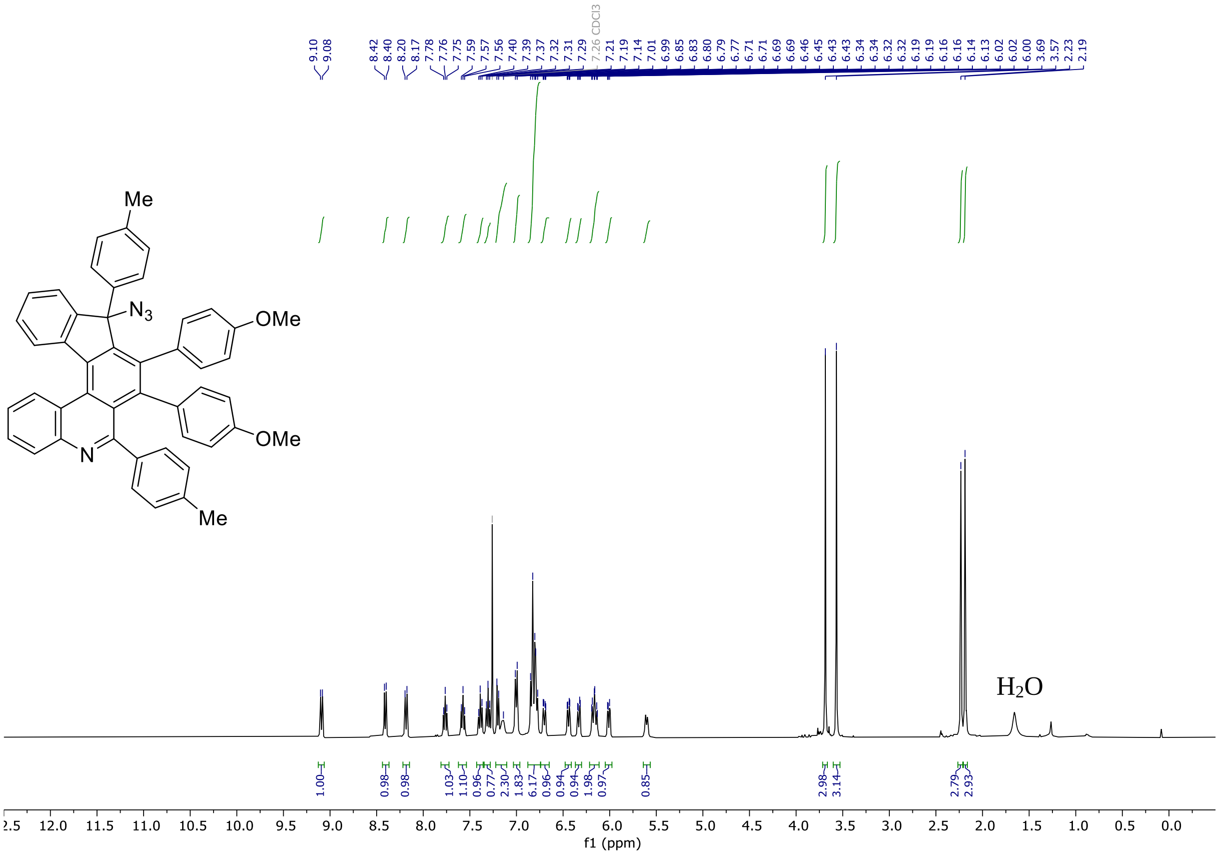

^{13}C NMR (101 MHz, $CDCl_3$, 25 °C)

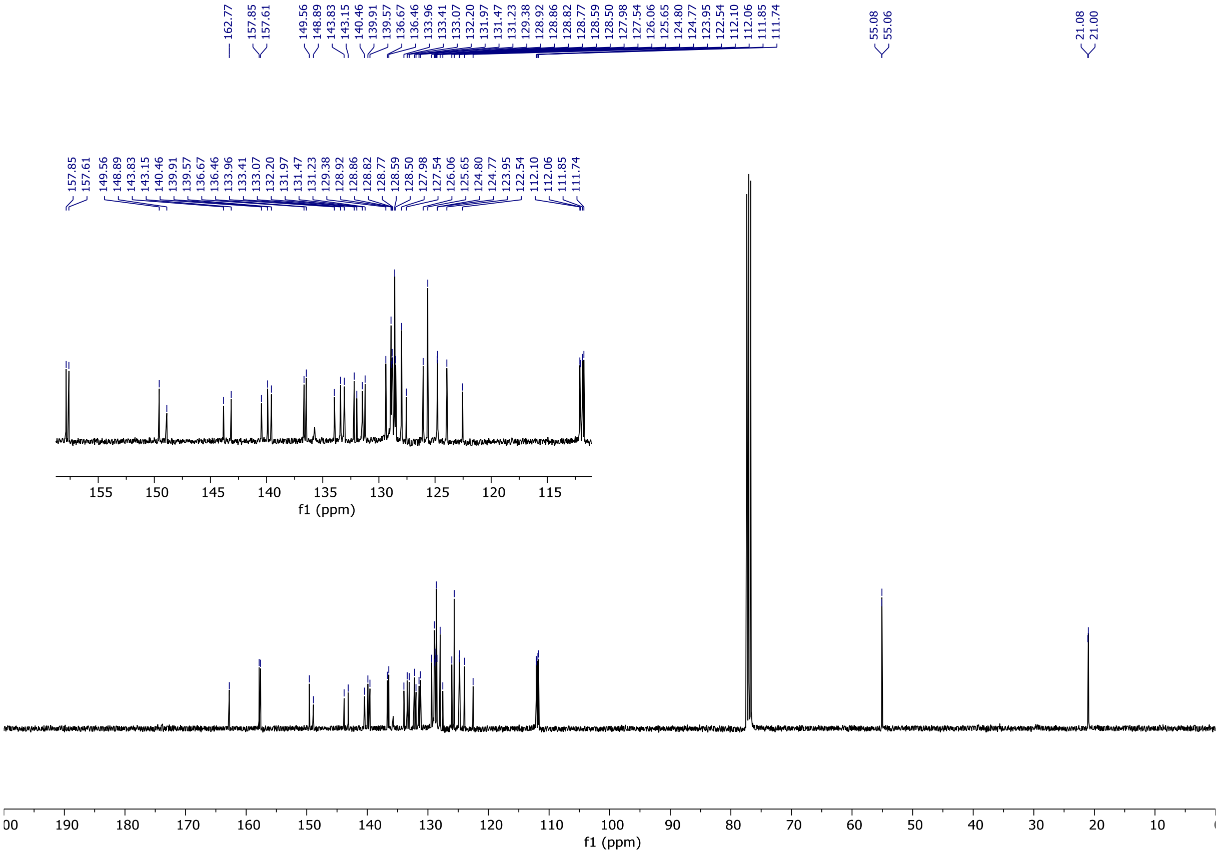

3,4-Bis(4-methoxyphenyl)-1,6-di-*p*-tolyldibenzo[*a*,*k*][4,7]phenanthroline (4a)

^{1}H NMR (400 MHz, $CDCl_3$, 25 °C)

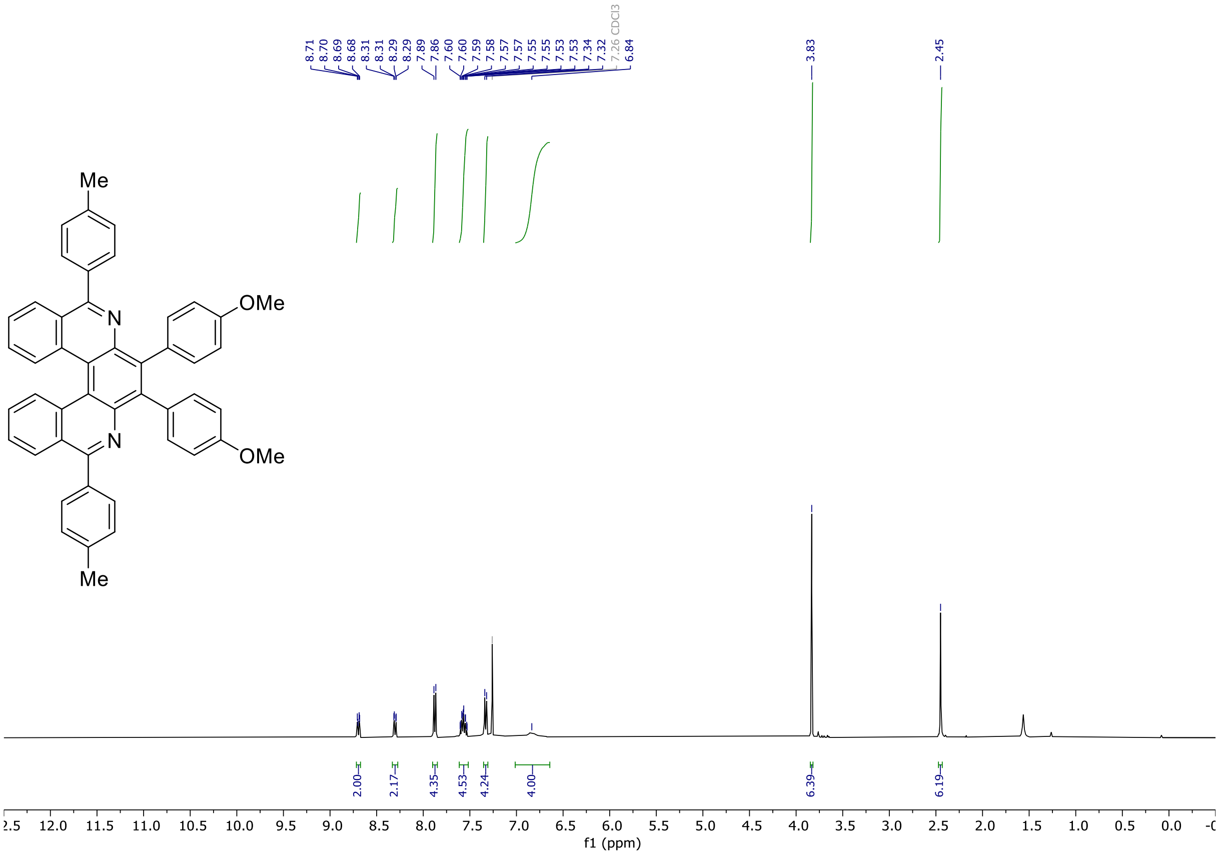

^{13}C NMR (101 MHz, $CDCl_3$, 25 °C)

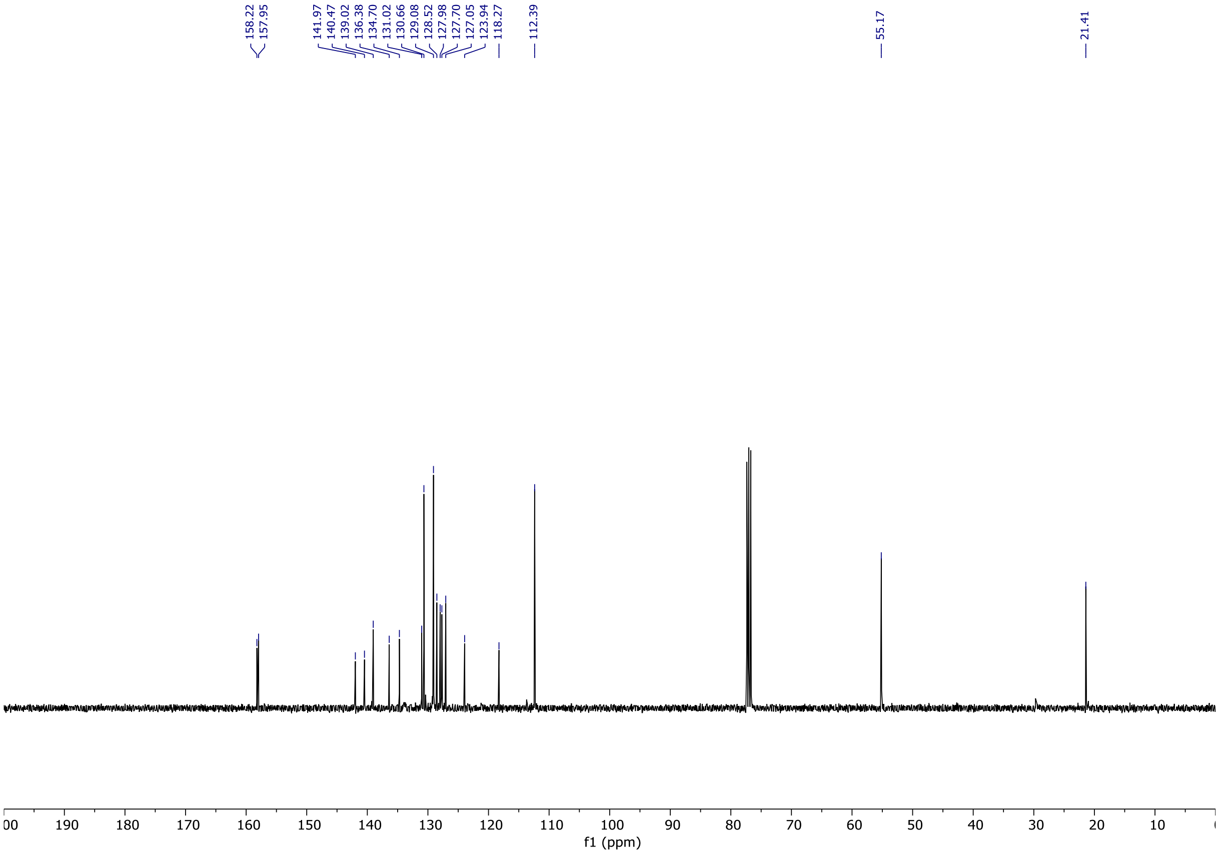

3,4-Bis(4-methoxyphenyl)-2,5-di-*p*-tolyldibenzo[*a*,*k*][3,8]phenanthroline (5a)

^{1}H NMR (400 MHz, $CDCl_3$, 25 °C)

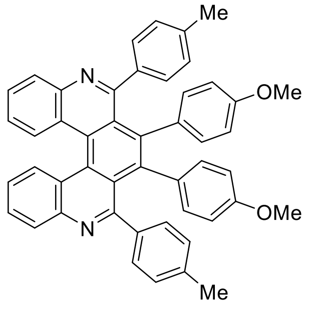

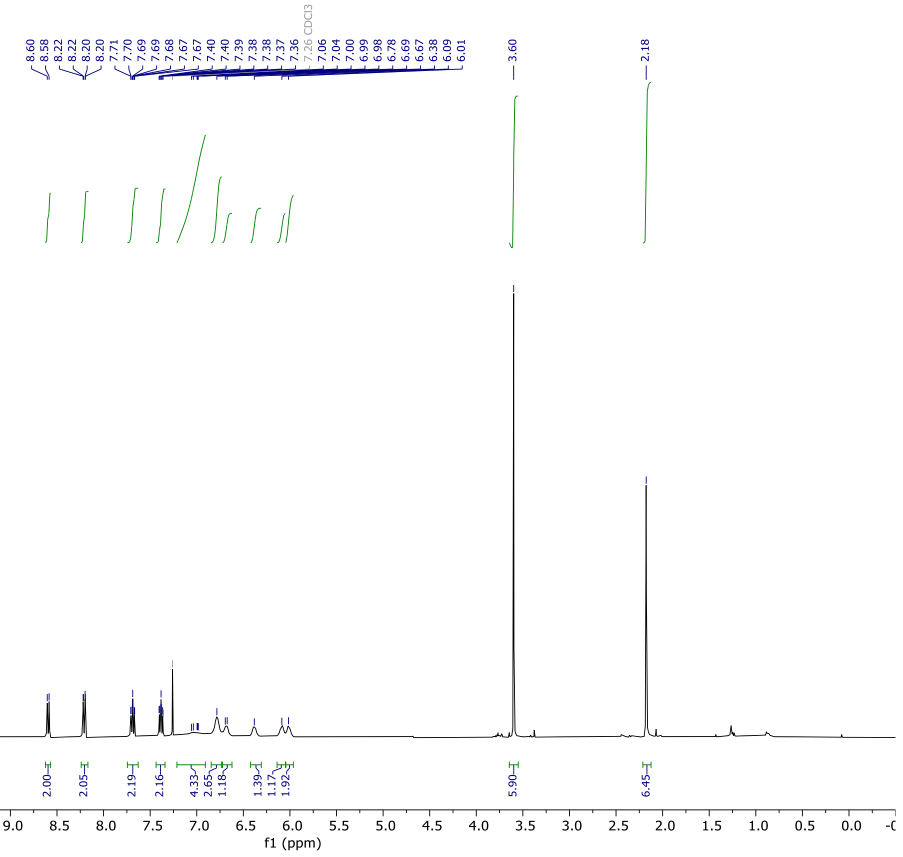

^{13}C NMR (101 MHz, $CDCl_3$, 25 °C)

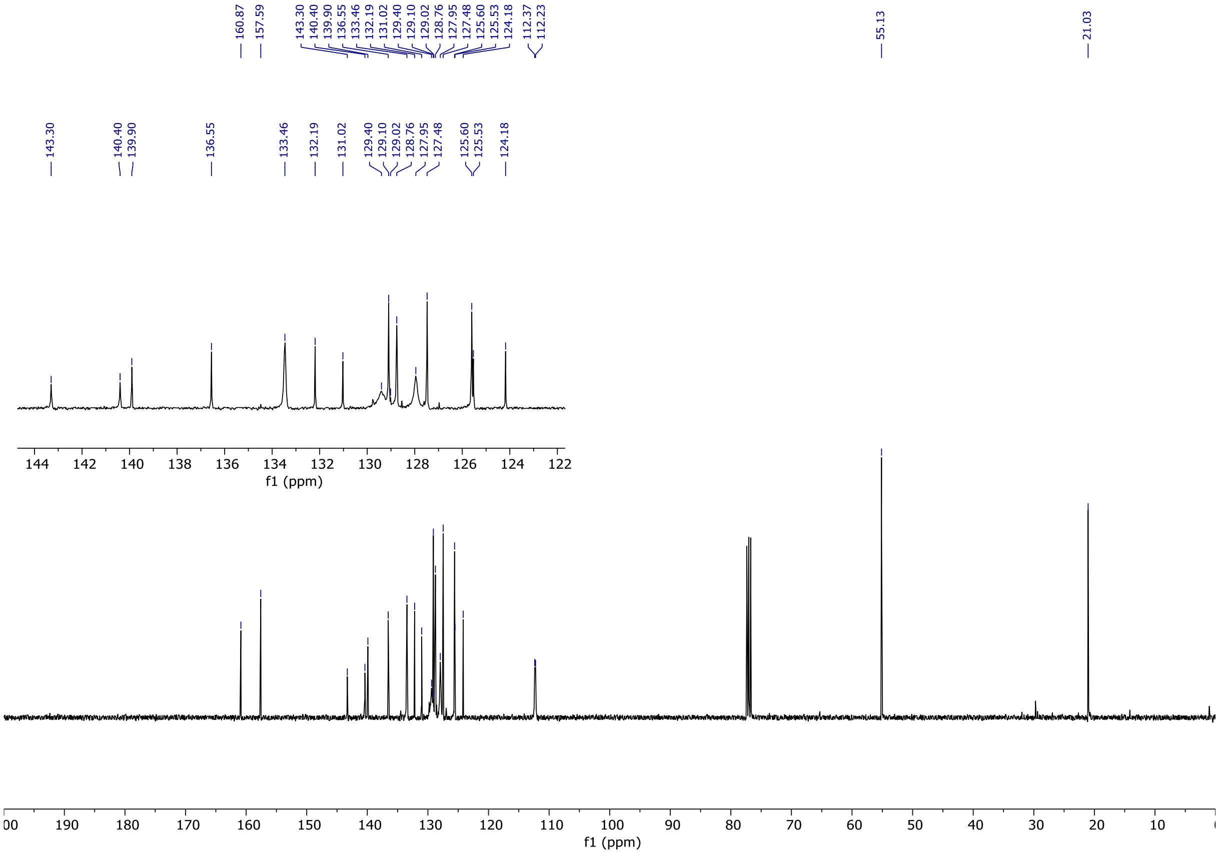

3,4-Bis(4-methoxyphenyl)-2,6-di-*p*-tolyldibenzo[*a,k*][3,7]phenanthroline (6a)

^{1}H NMR (400 MHz, C_6D_6, 25 °C)

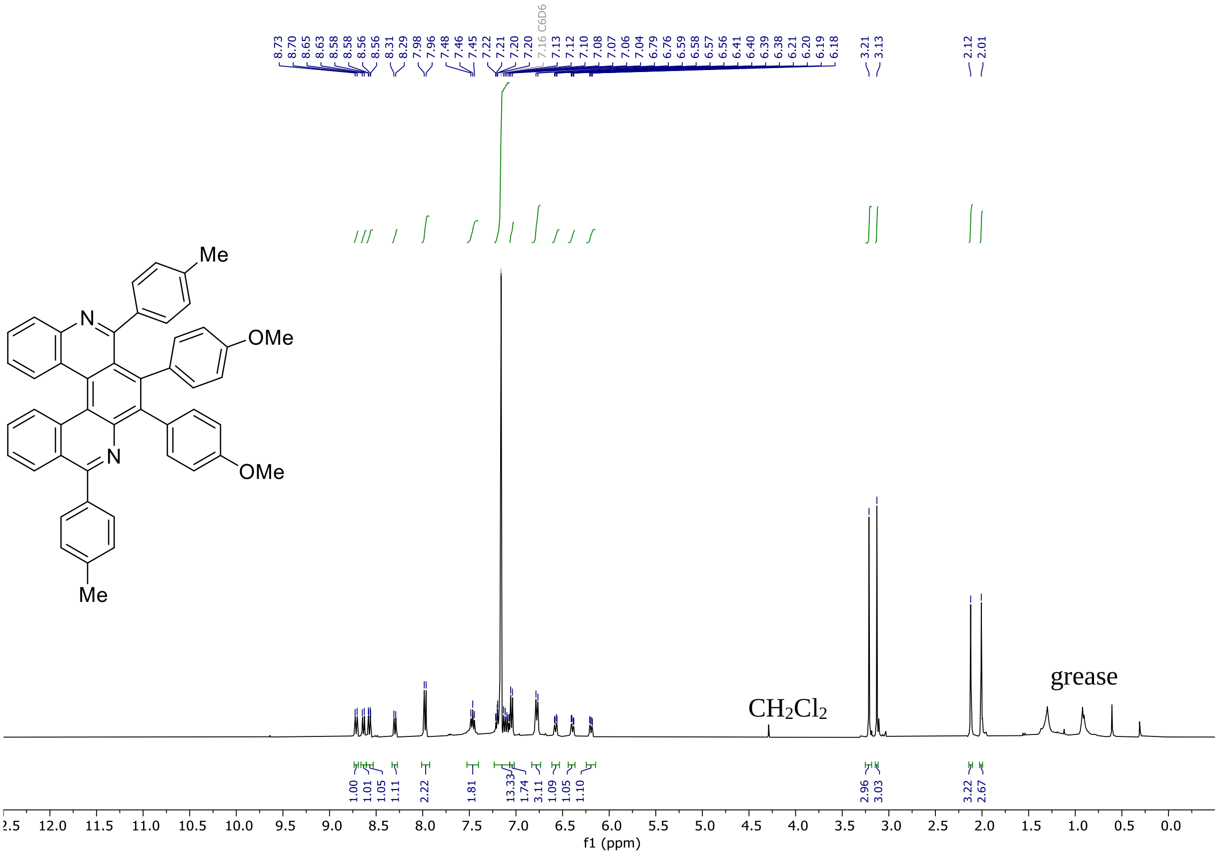

^{13}C NMR (101 MHz, C_6D_6, 25 °C)

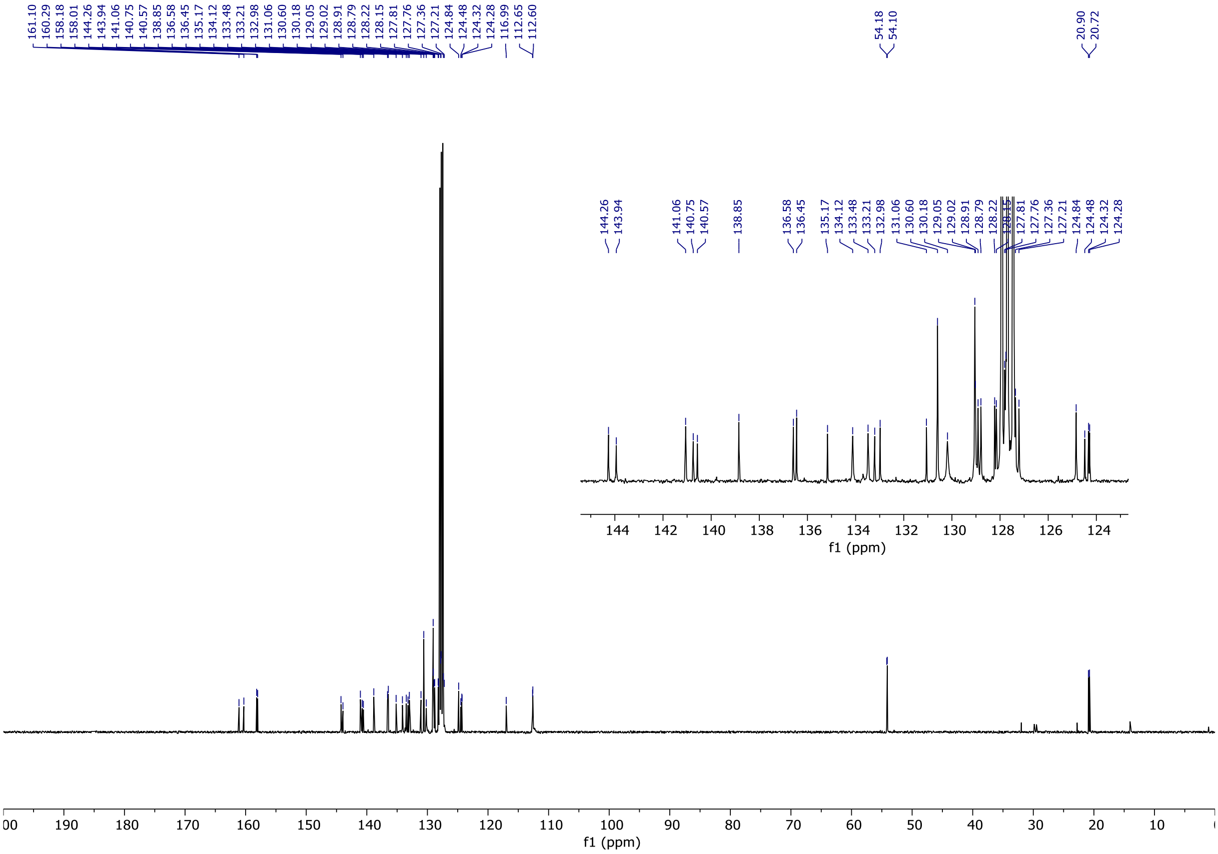

5,8-Diazido-6,7-bis(4-methoxyphenyl)-5,8-di-*p*-tolyl-5,8-dihydroindeno[2,1-*c*]fluorene (7a)

^{1}H NMR (400 MHz, $CDCl_3$, 25 °C)

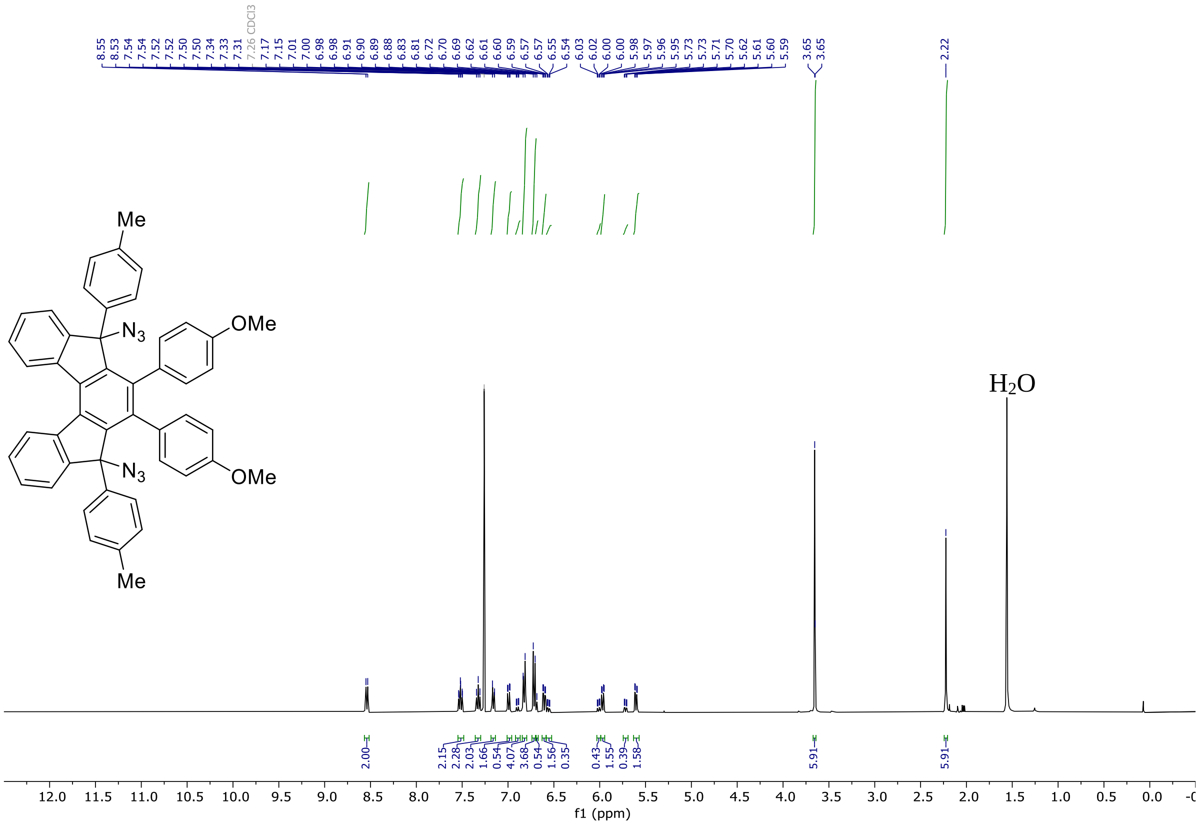

^{13}C NMR (101 MHz, $CDCl_3$, 25 °C)

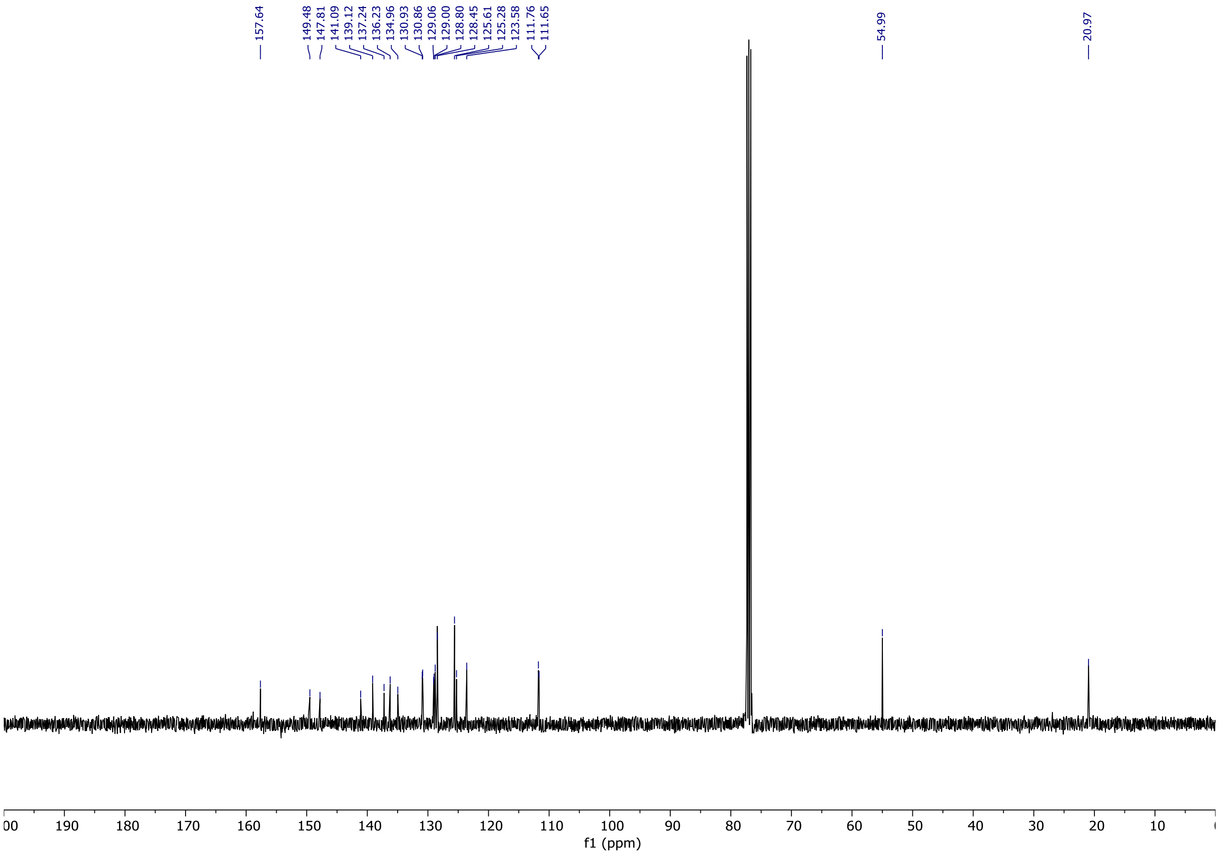

3,4-Bis(4-methoxyphenyl)-1,6-di-*o*-tolyldibenzo[*a*,*k*][4,7]phenanthroline (4b)

^{1}H NMR (400 MHz, $CDCl_3$, 25 °C)

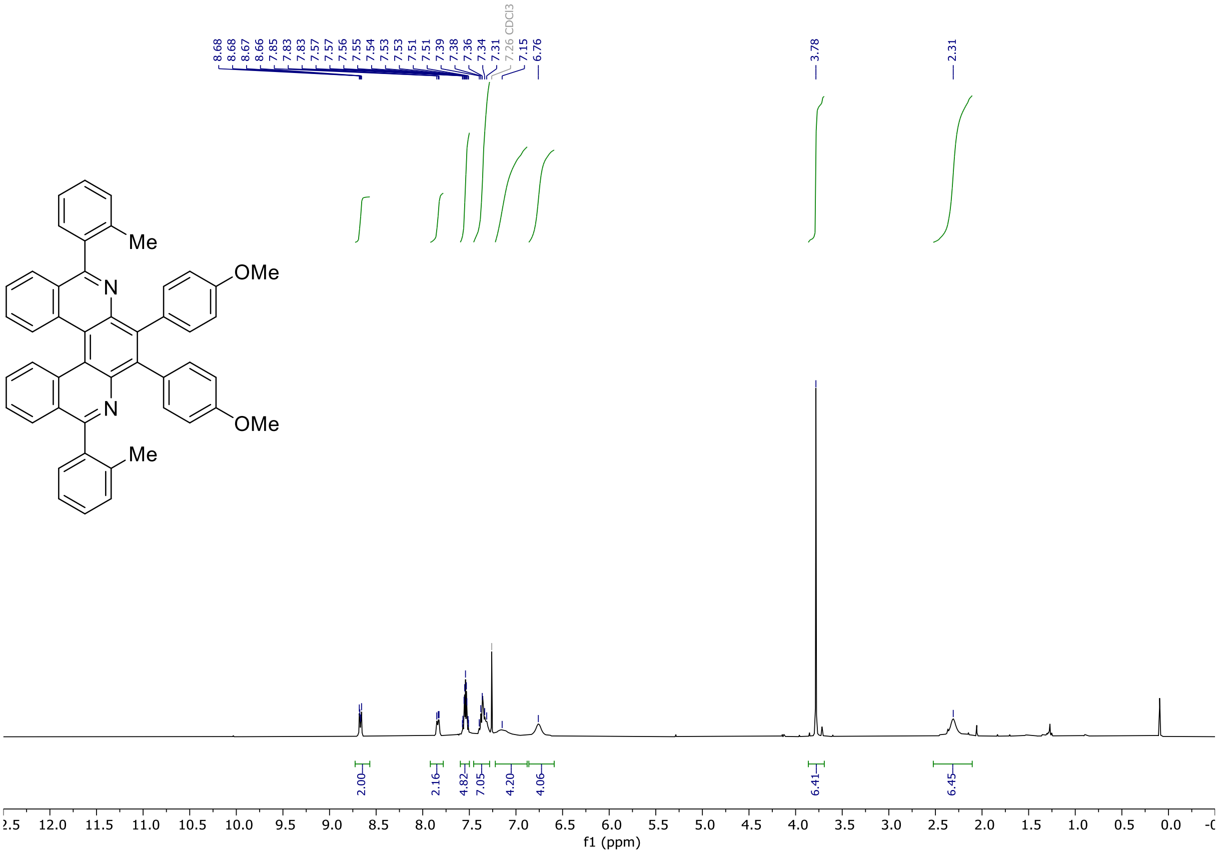

^{13}C NMR (101 MHz, $CDCl_3$, 25 °C)

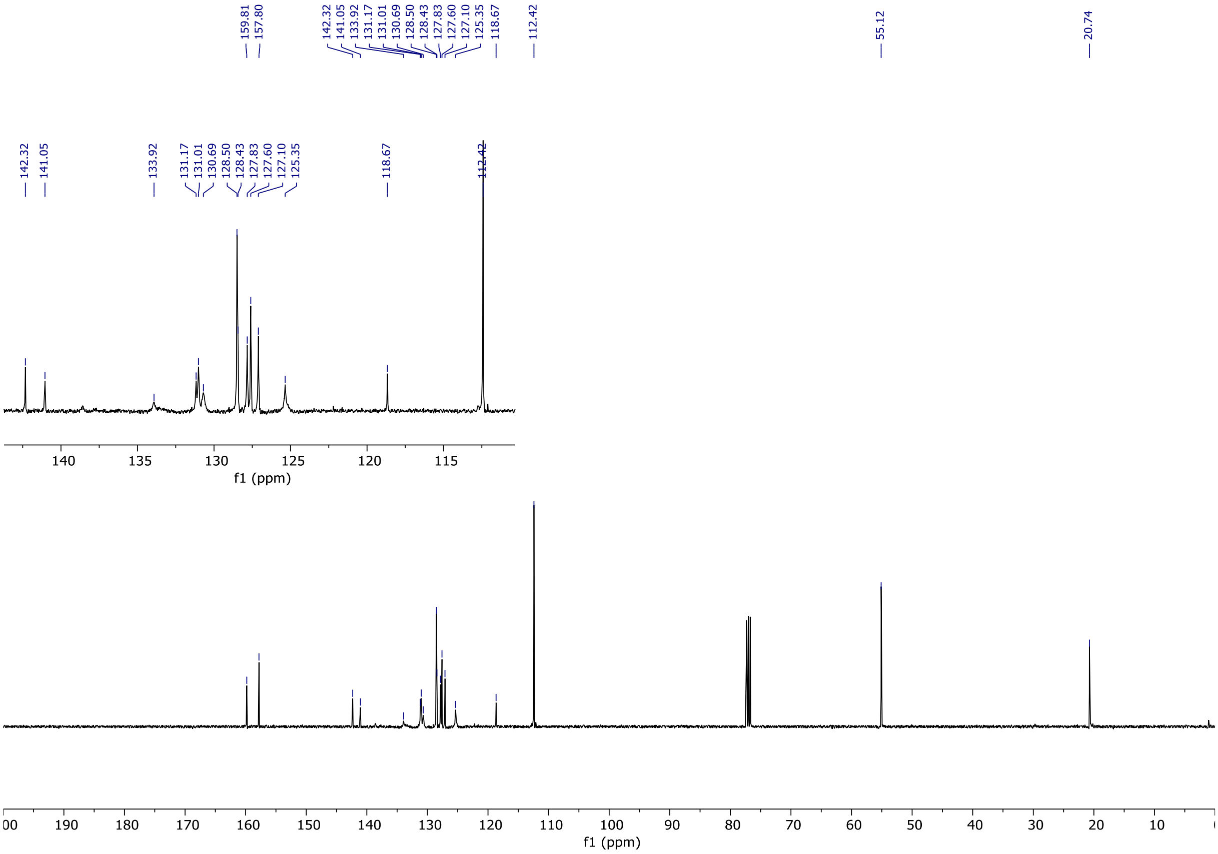

3,4-Bis(4-methoxyphenyl)-2,6-di-*o*-tolyldibenzo[*a*,*k*][3,7]phenanthroline (6f)

^{1}H NMR (400 MHz, $CDCl_3$, 25 °C)

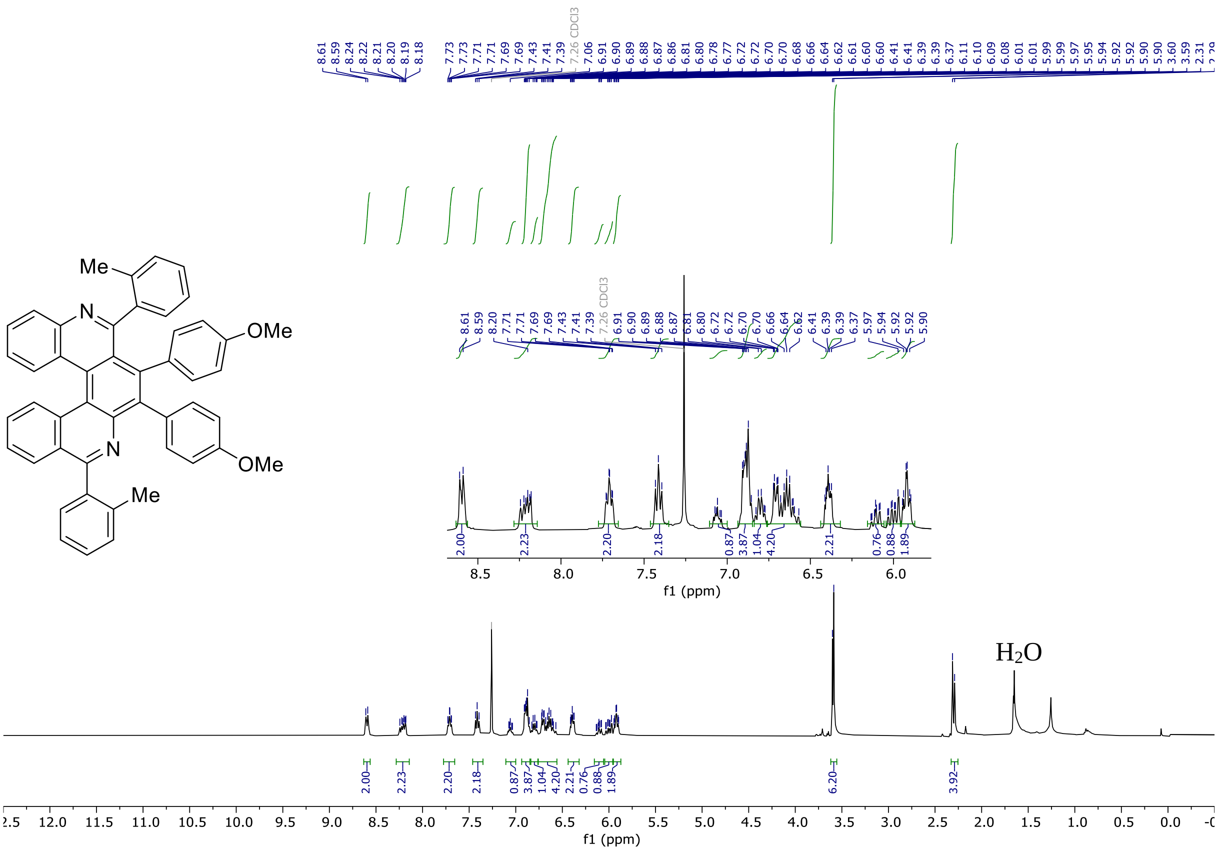

^{13}C NMR (101 MHz, $CDCl_3$, 25 °C)

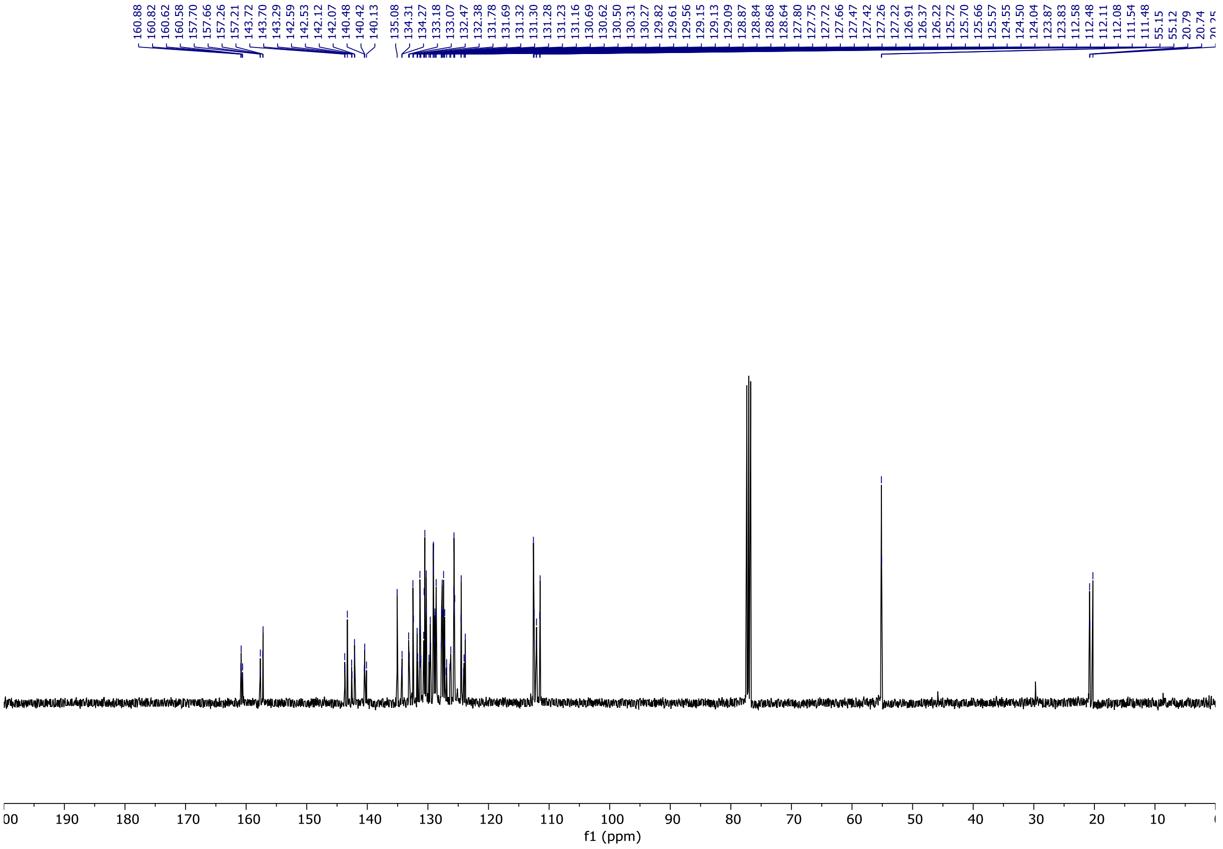

1,6-Dibutyl-3,4-bis(4-methoxyphenyl)dibenzo[*a*,*k*][4,7]phenanthroline (4c)

^{1}H NMR (400 MHz, $CDCl_3$, 25 °C)

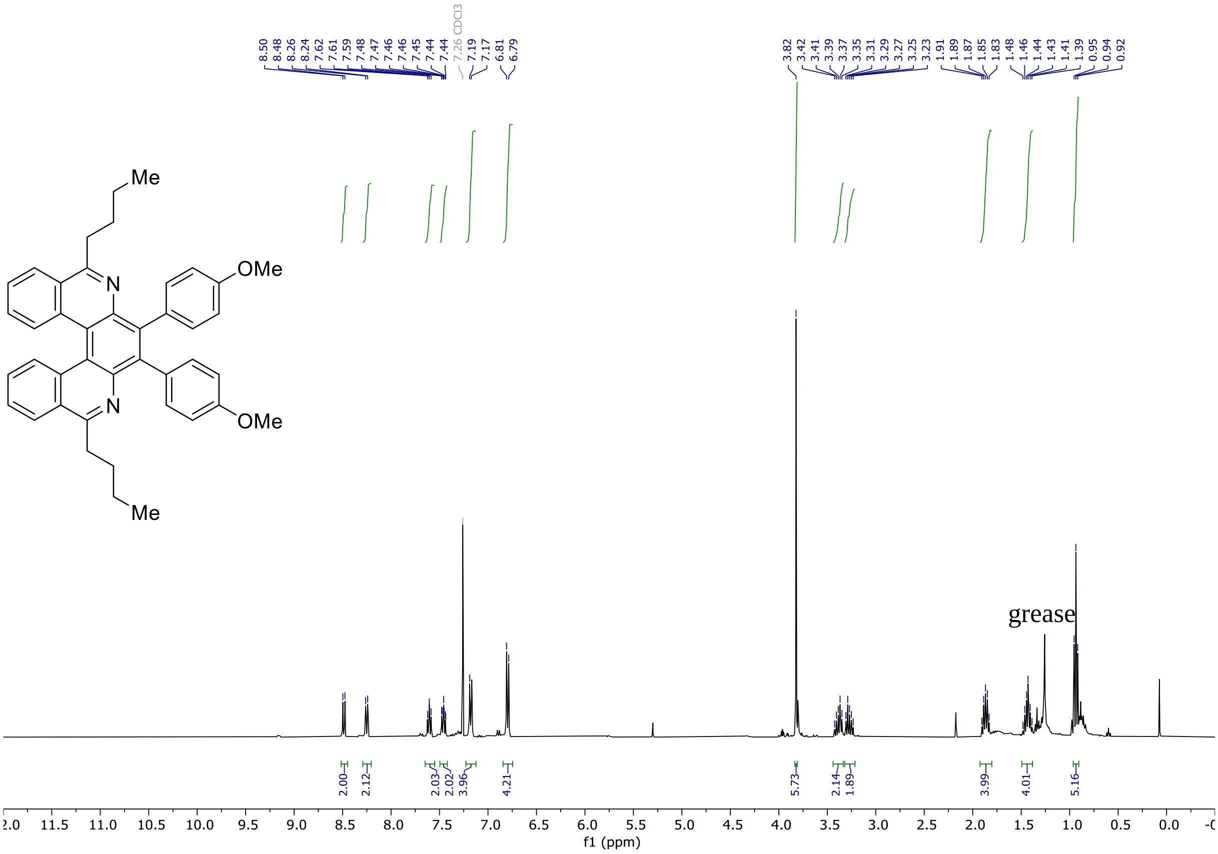

^{13}C NMR (101 MHz, $CDCl_3$, 25 °C)

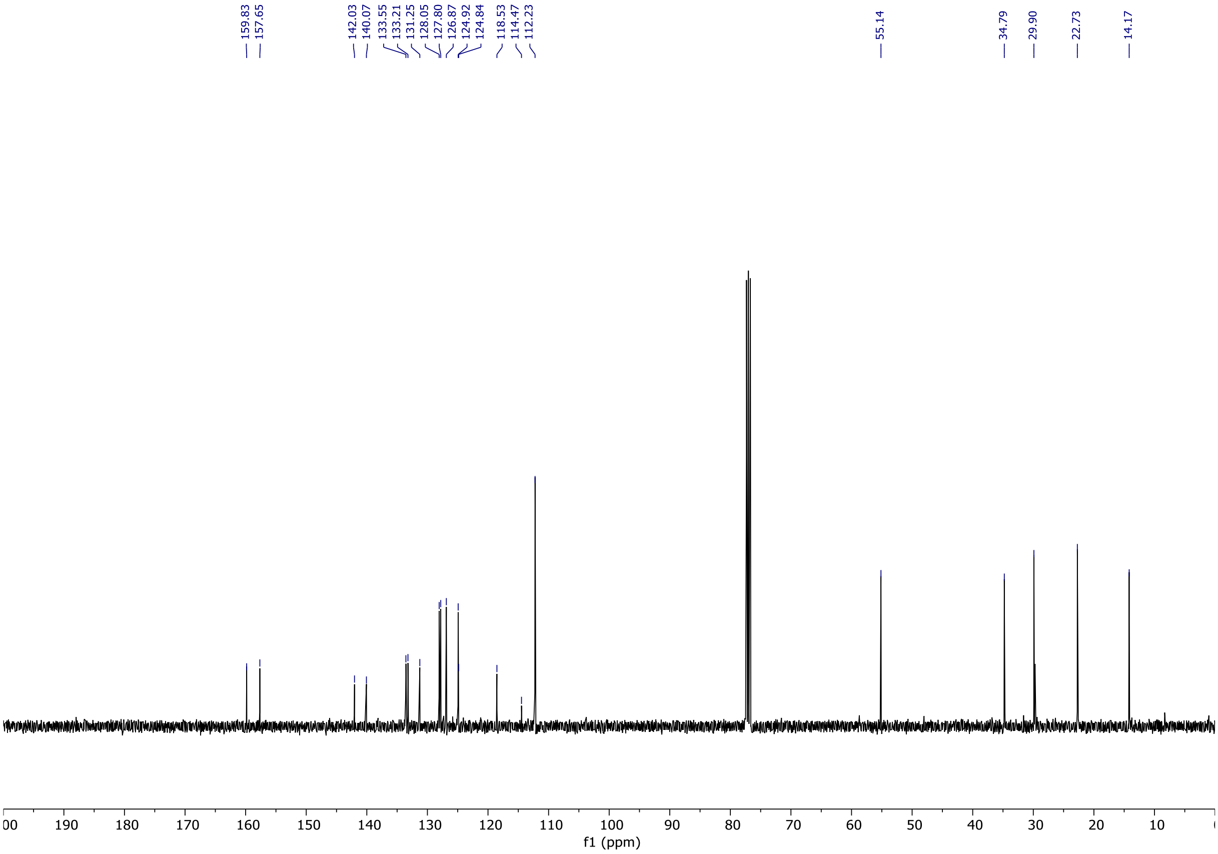

2,6-Dibutyl-3,4-bis(4-methoxyphenyl)dibenzo[*a*,*k*][3,7]phenanthroline (6c)

^{1}H NMR (400 MHz, C_6D_6, 25 °C)

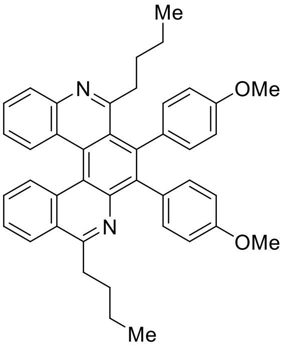

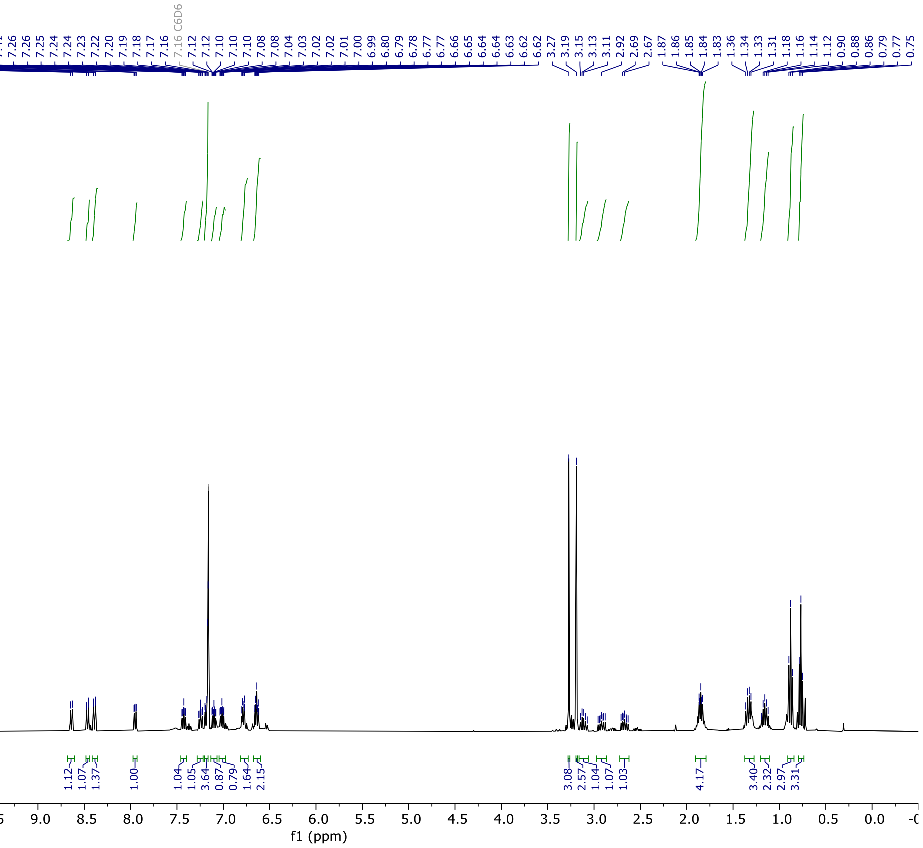

^{13}C NMR (101 MHz, C_6D_6, 25 °C)

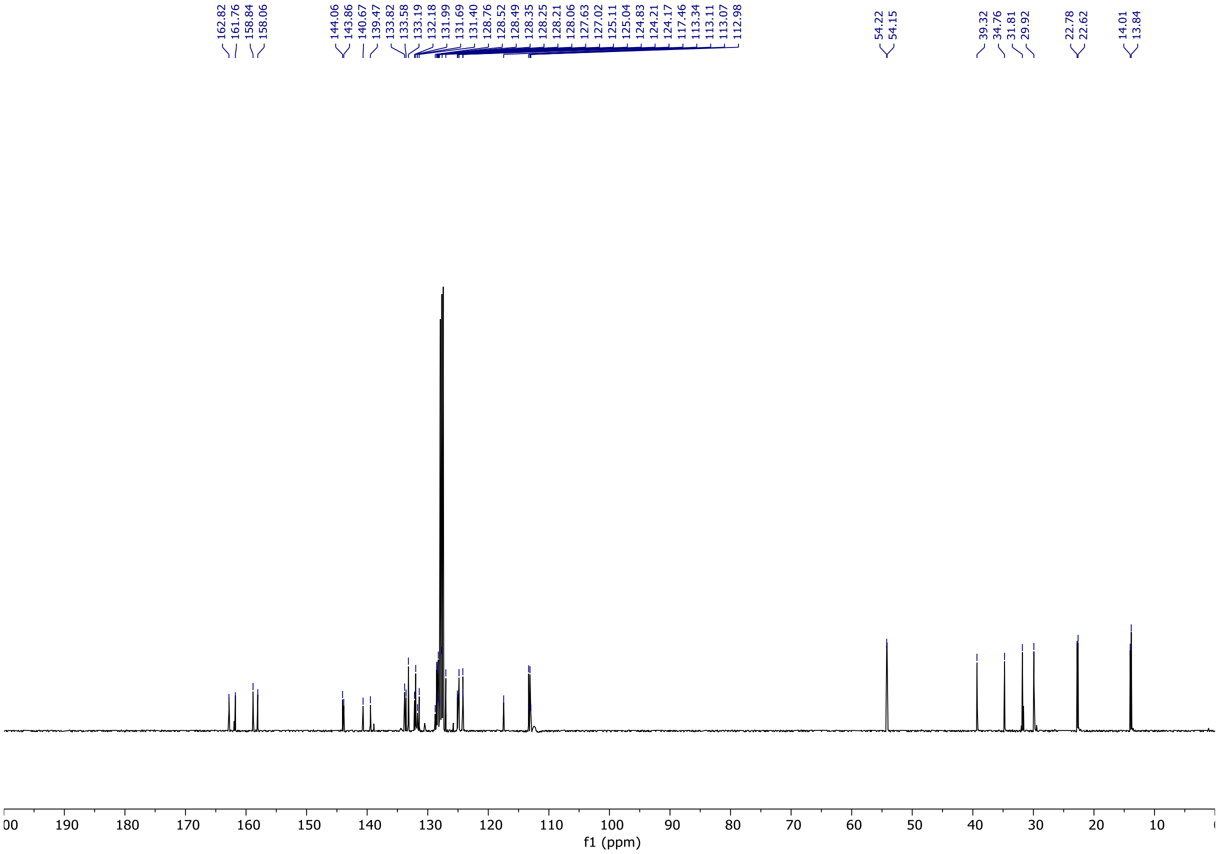

3,4-Bis(4-methoxyphenyl)-1,6-bis(4-(trifluoromethyl)phenyl)dibenzo[*a*,*k*][4,7]phenanthroline (4d)

^{1}H NMR (400 MHz, $CDCl_3$, 25 °C)

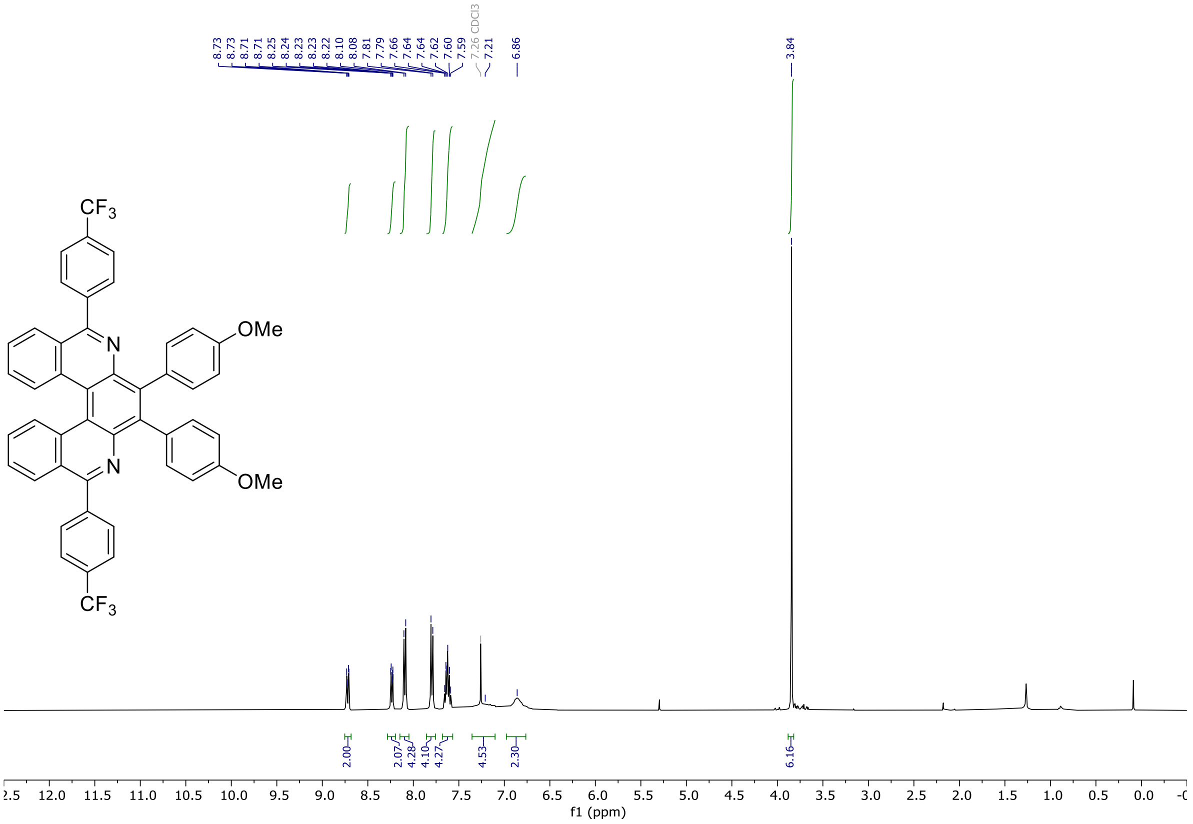

^{13}C NMR (101 MHz, $CDCl_3$, 25 °C)

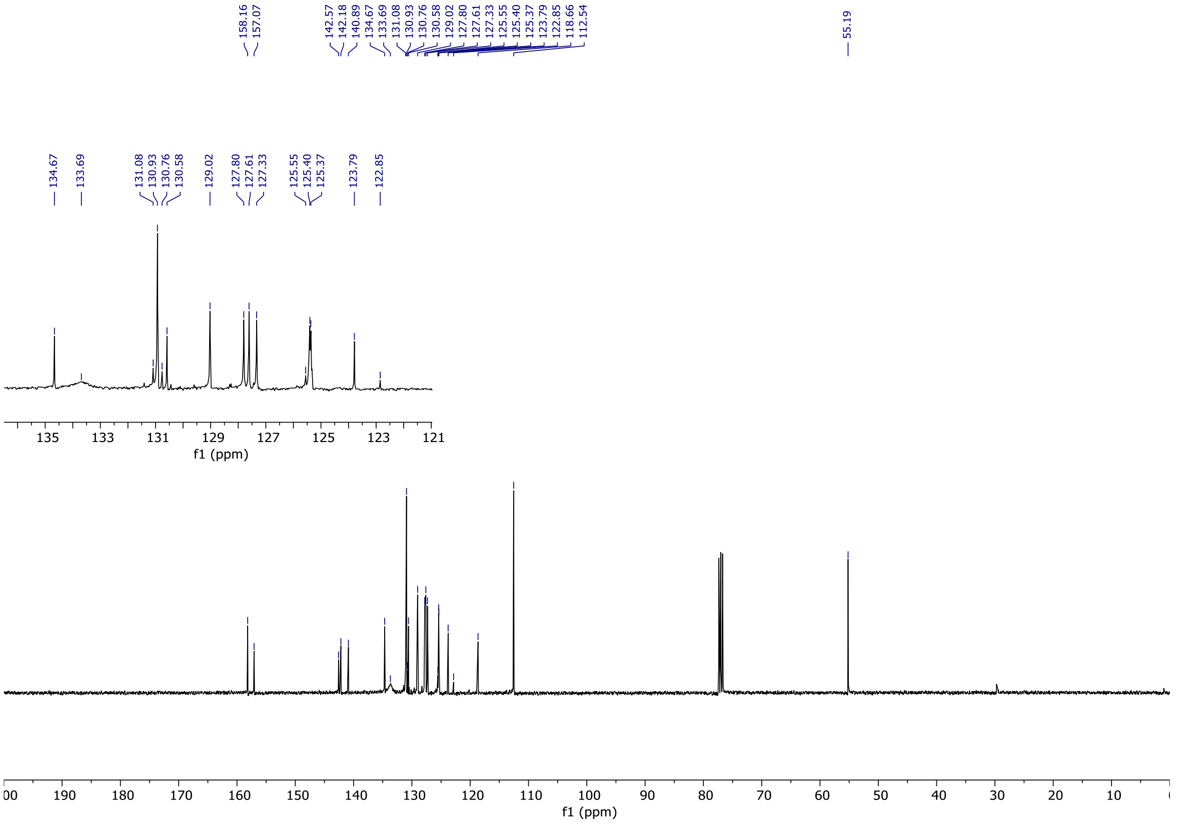

^{19}F NMR (376.5 MHz, $CDCl_3$, 25 °C)

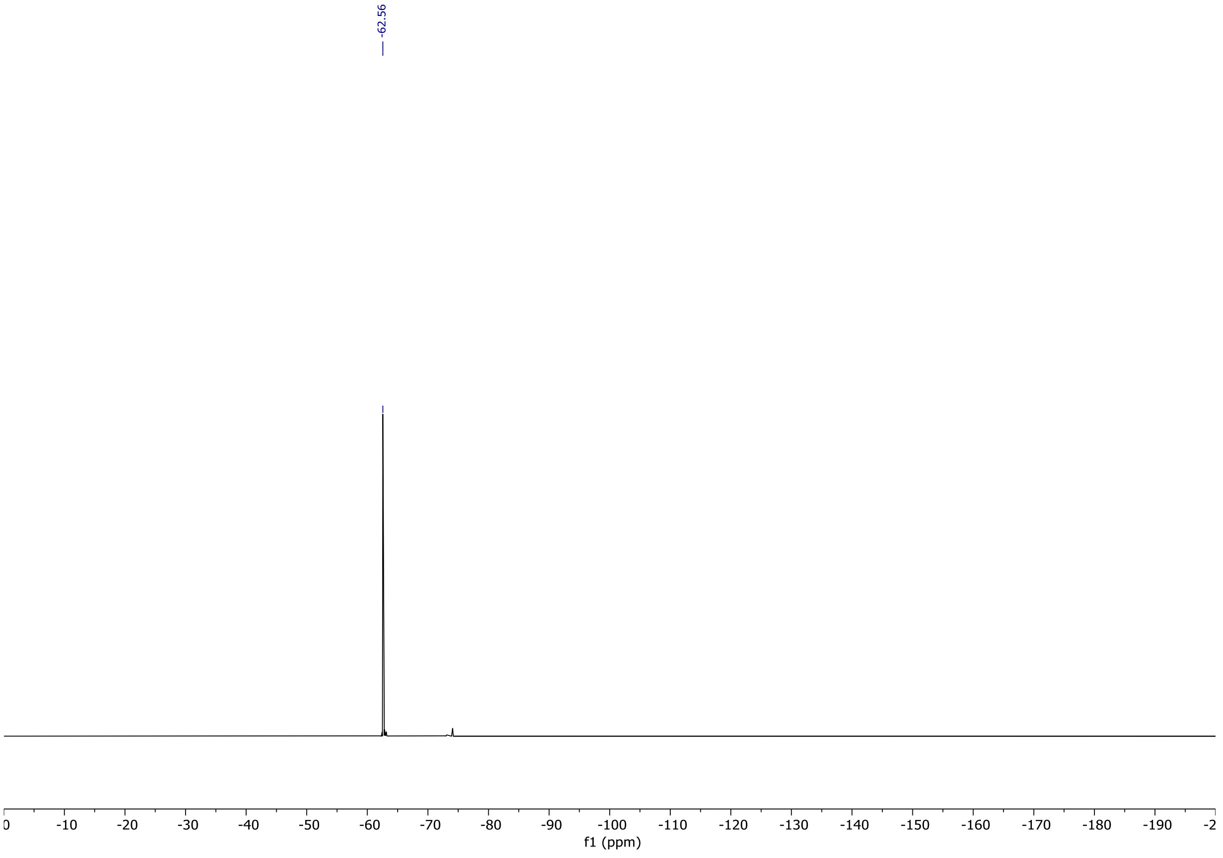

3,4-Bis(4-methoxyphenyl)-2,5-bis(4-(trifluoromethyl)phenyl)dibenzo[*a*,*k*][3,8]phenanthroline (5d)

^{1}H NMR (400 MHz, $CDCl_3$, 25 °C)

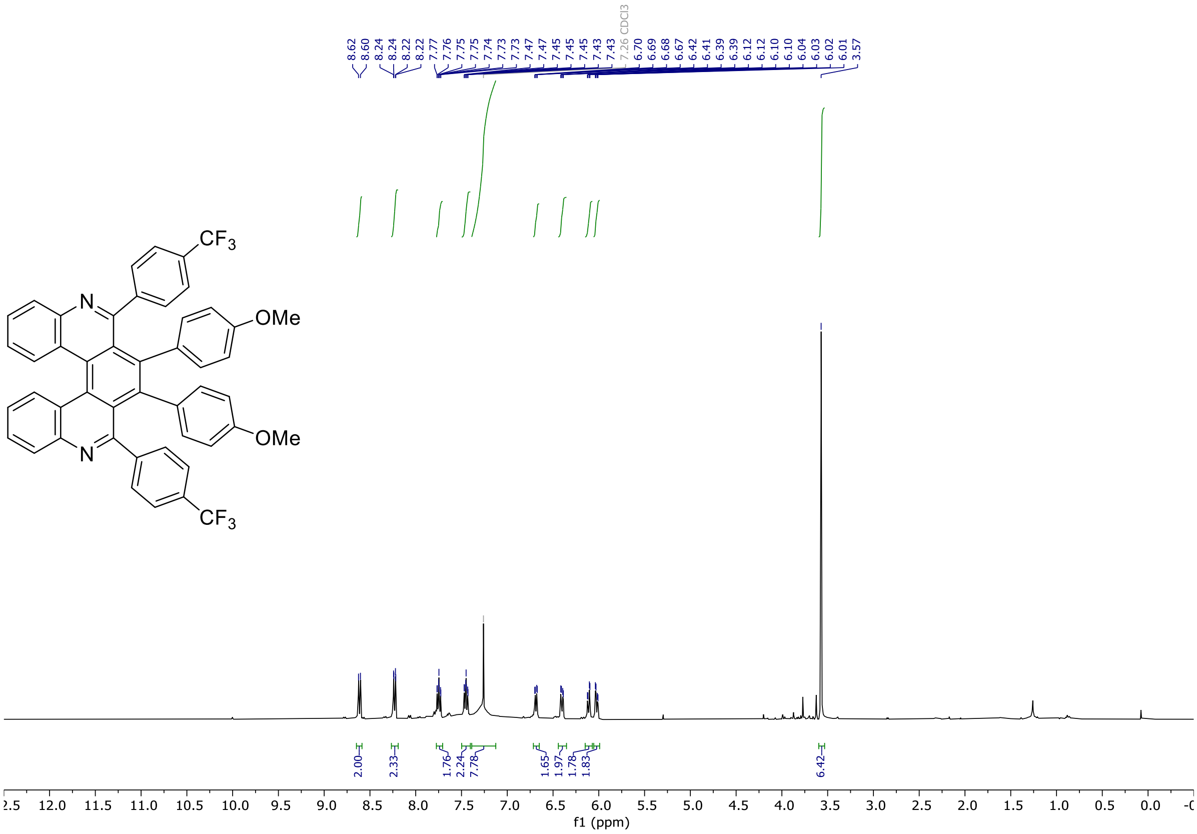

^{13}C NMR (101 MHz, $CDCl_3$, 25 °C)

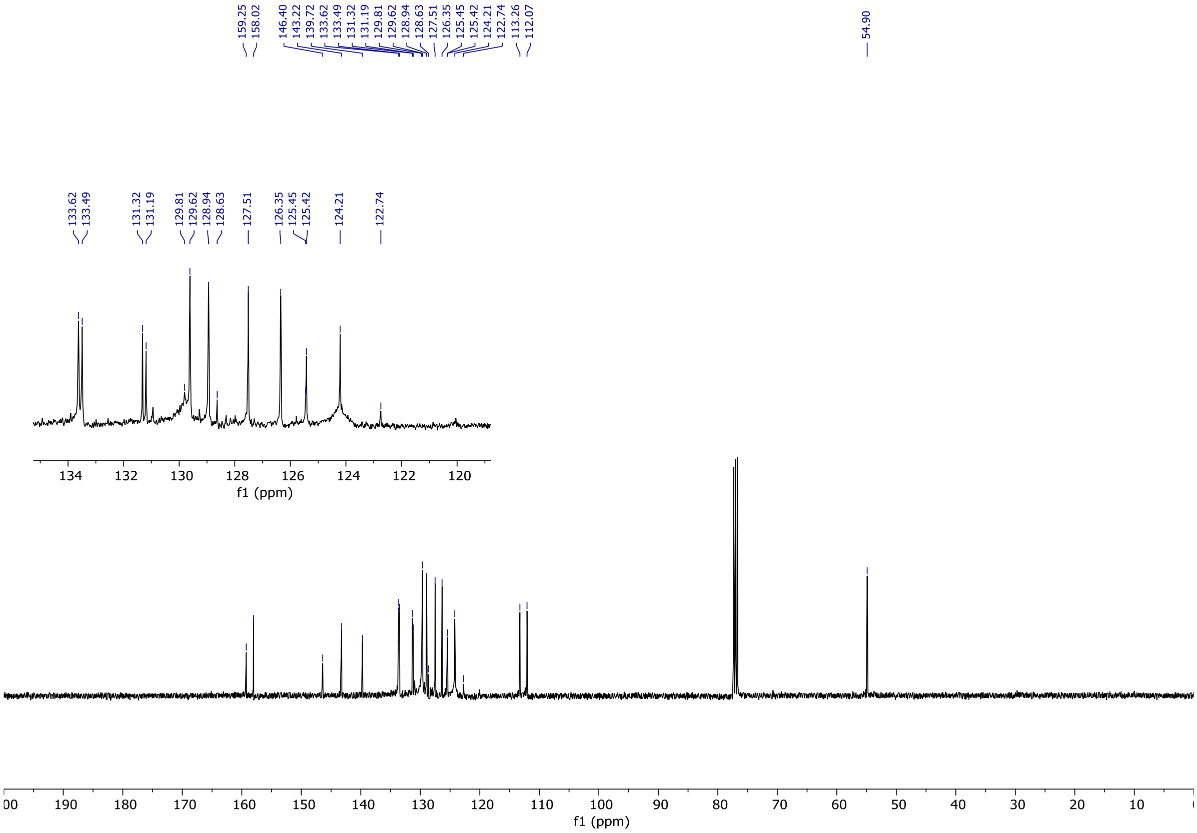

^{19}F NMR (376.5 MHz, $CDCl_3$, 25 °C)

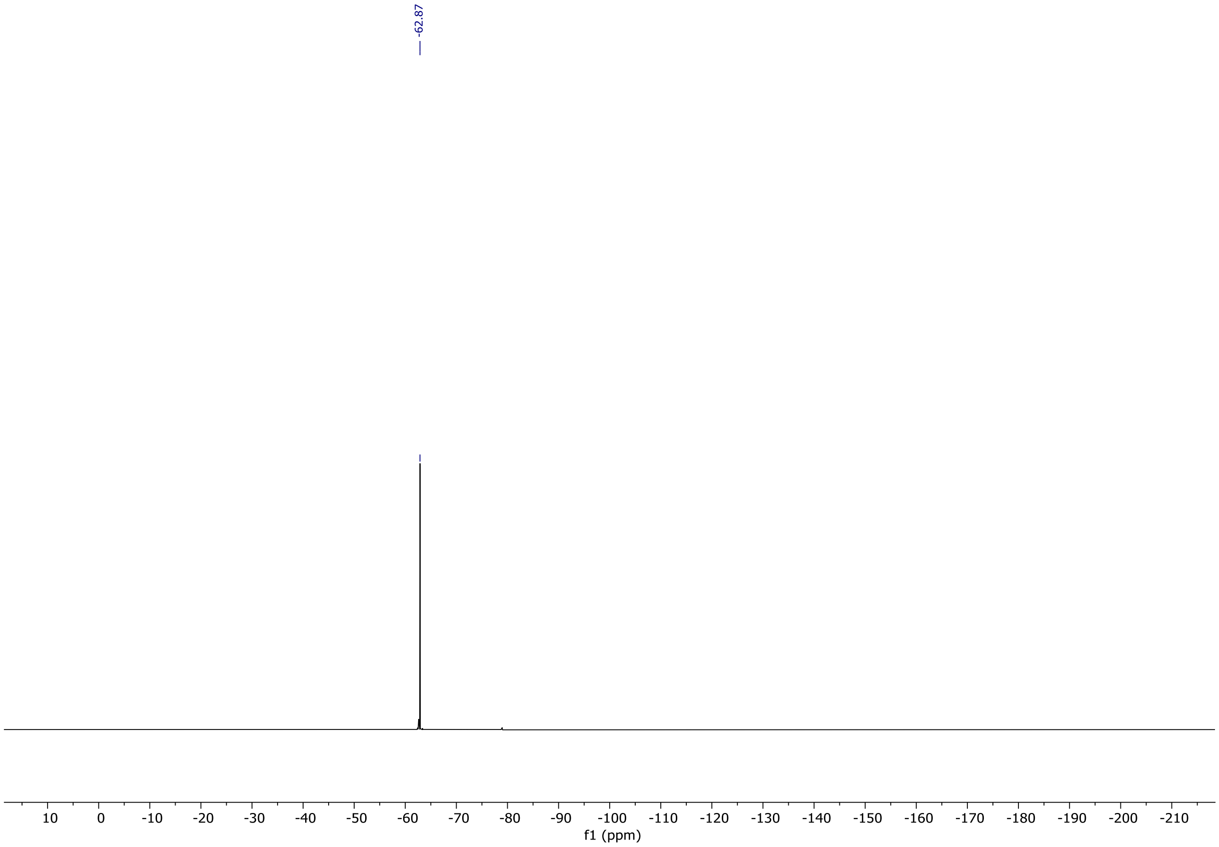

3,4-Bis(4-methoxyphenyl)-2,6-bis(4-(trifluoromethyl)phenyl)dibenzo[a,k][3,7]phenanthroline (6d in a mixture with **5d)**

^{1}H NMR (400 MHz, $CDCl_3$, 25 °C)

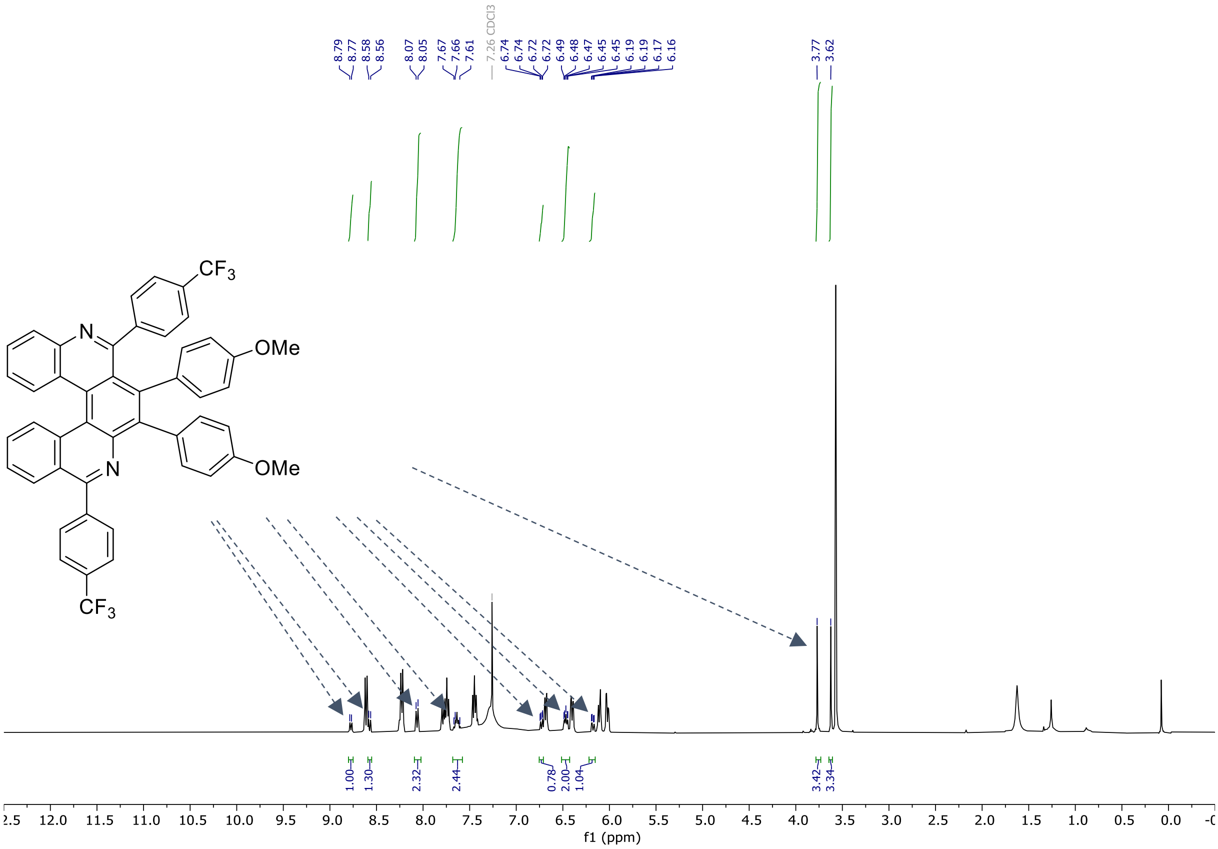

^{13}C NMR (101 MHz, $CDCl_3$, 25 °C)

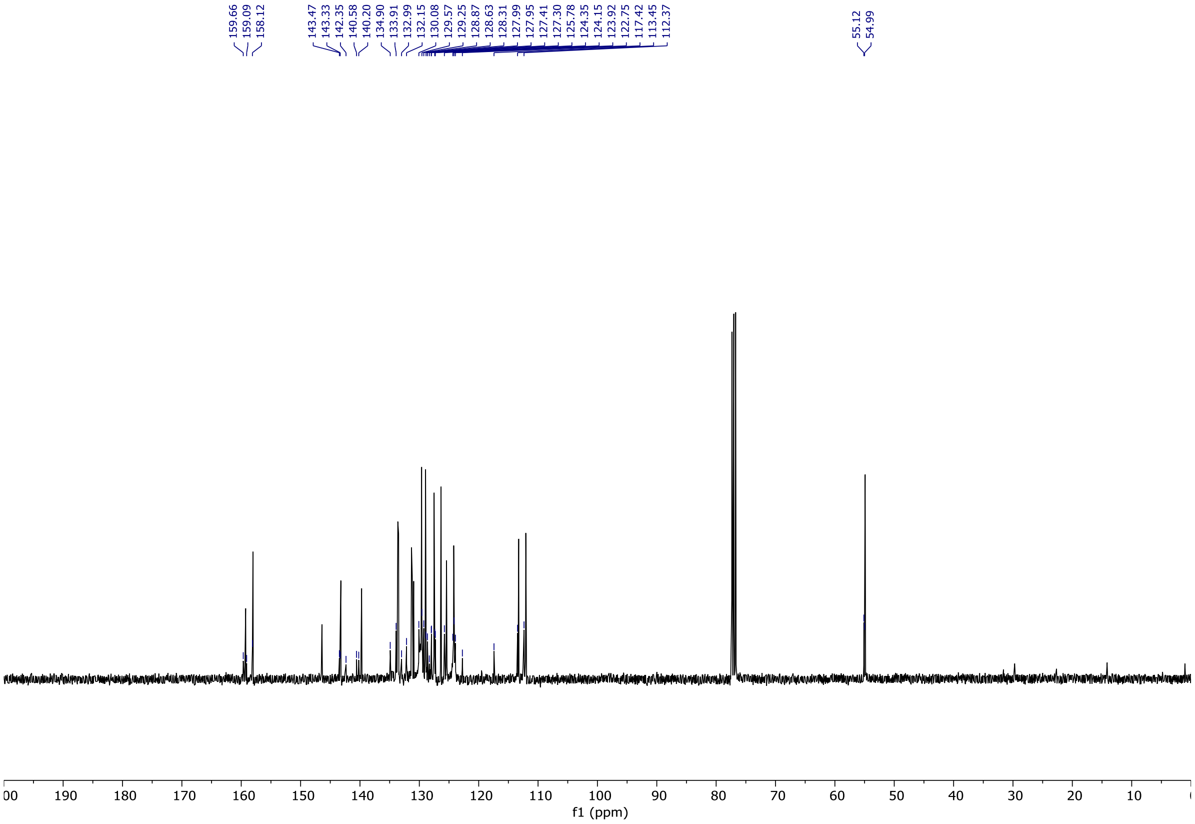

^{19}F NMR (376.5 MHz, $CDCl_3$, 25 °C)

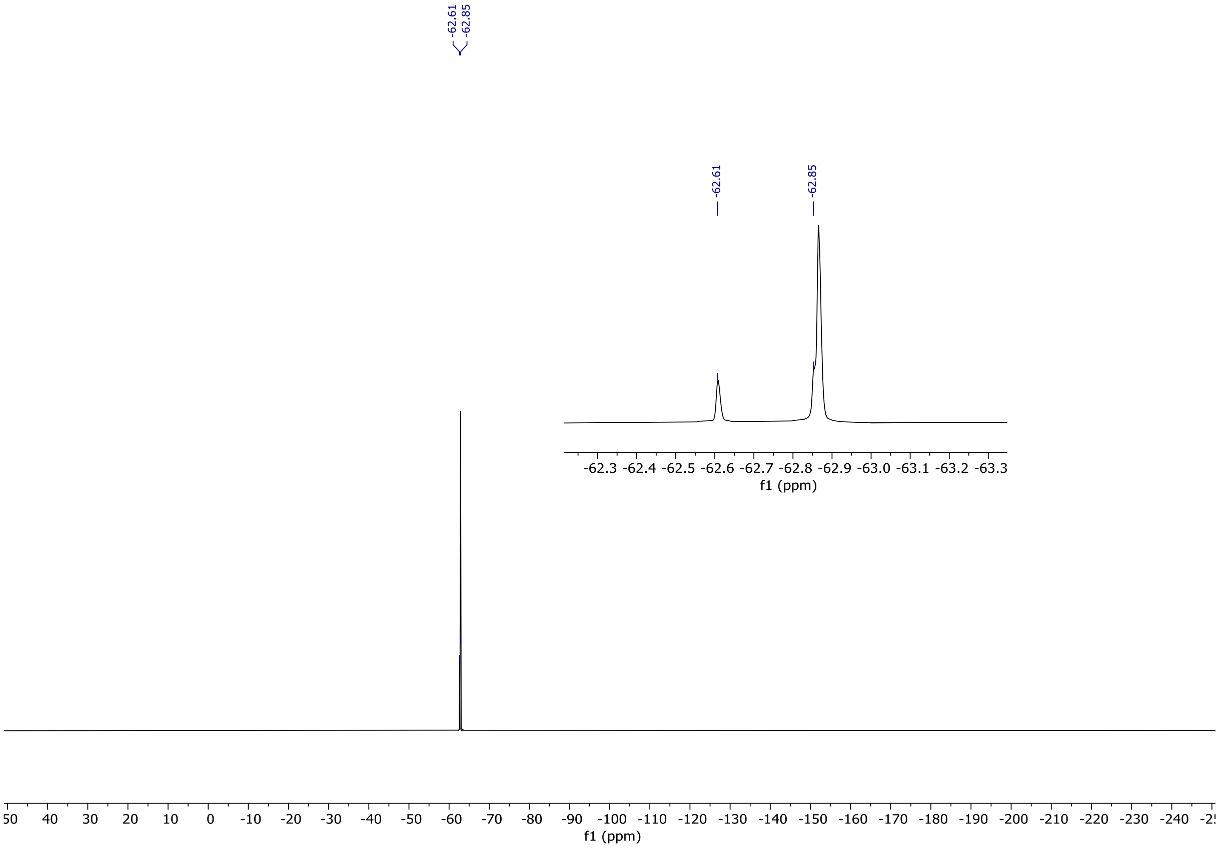

1,6-Di-*p*-tolyl-3,4-bis(4-(trifluoromethyl)phenyl)dibenzo[*a*,*k*][4,7]phenanthroline (4e)

^{1}H NMR (400 MHz, $CDCl_3$, 25 °C)

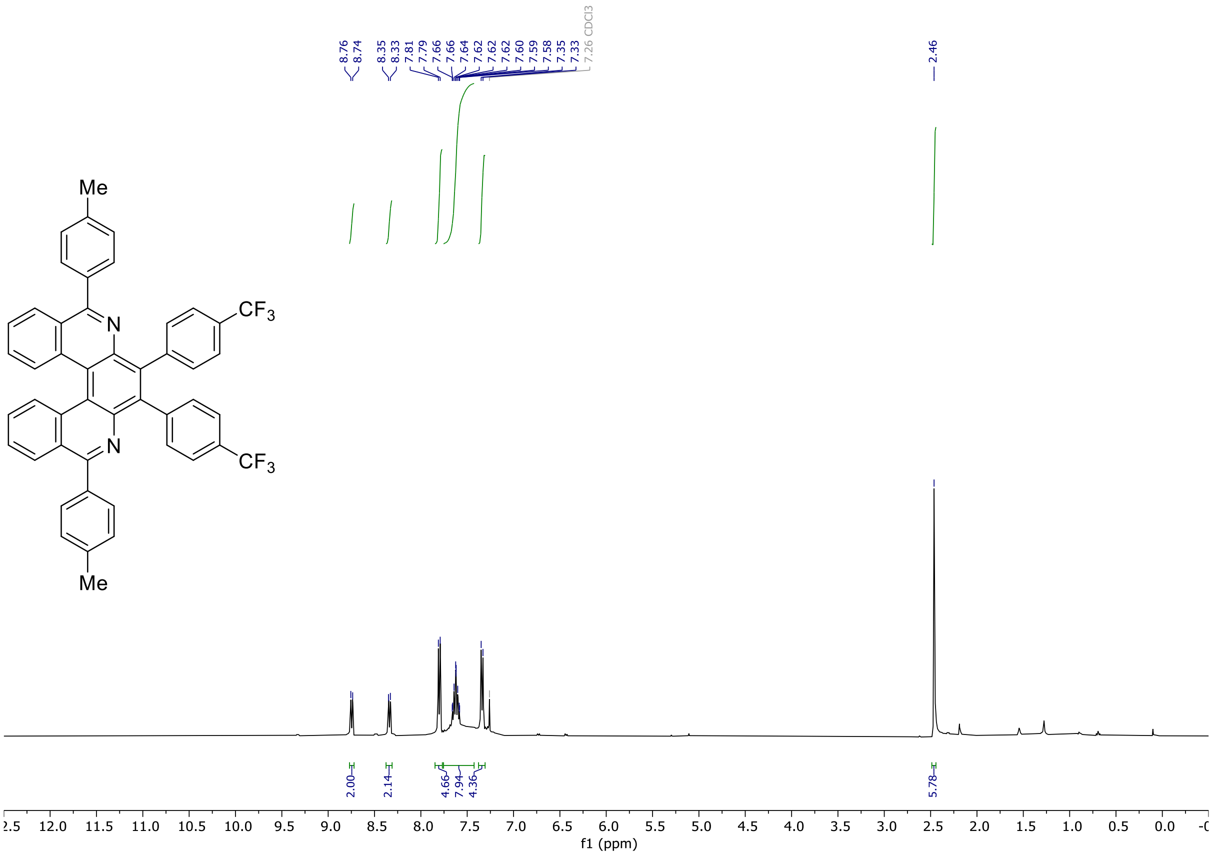

^{13}C NMR (101 MHz, $CDCl_3$, 25 °C)

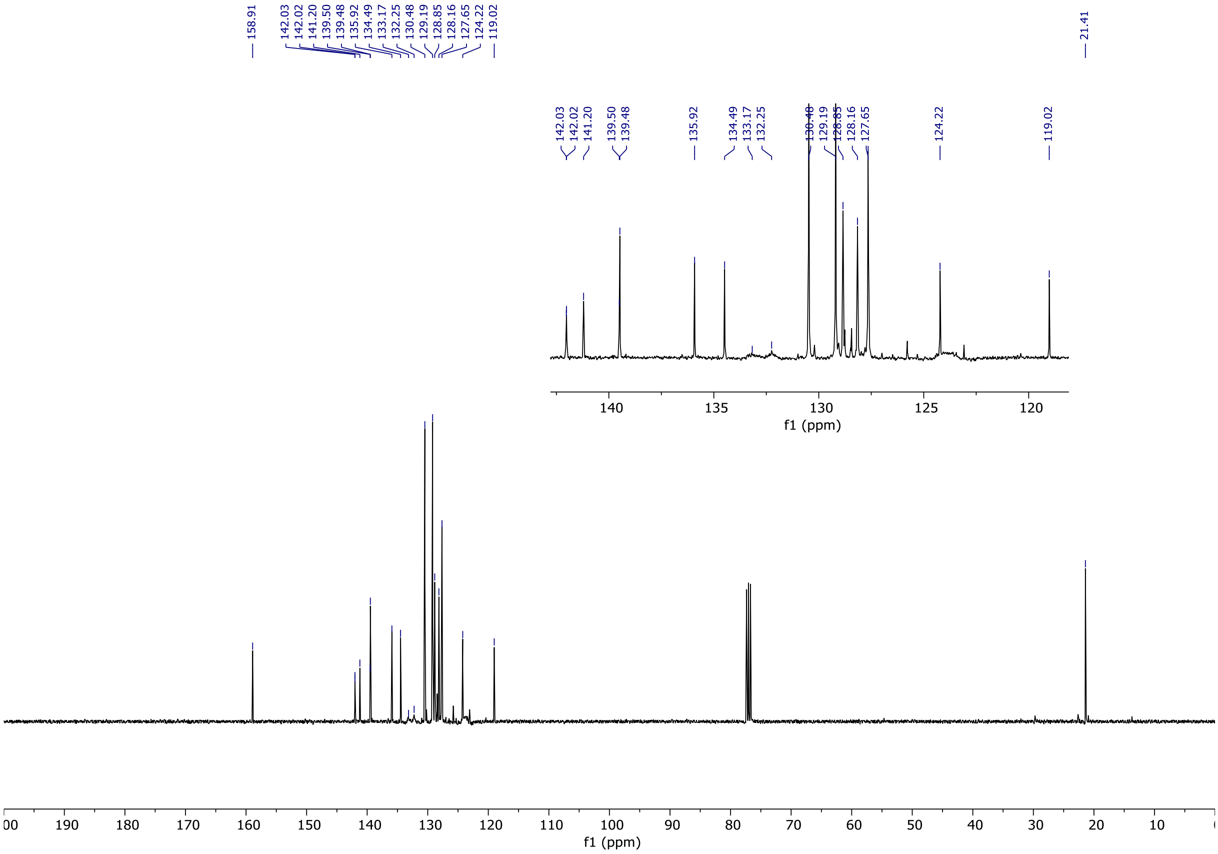

^{19}F NMR (376.5 MHz, $CDCl_3$, 25 °C)

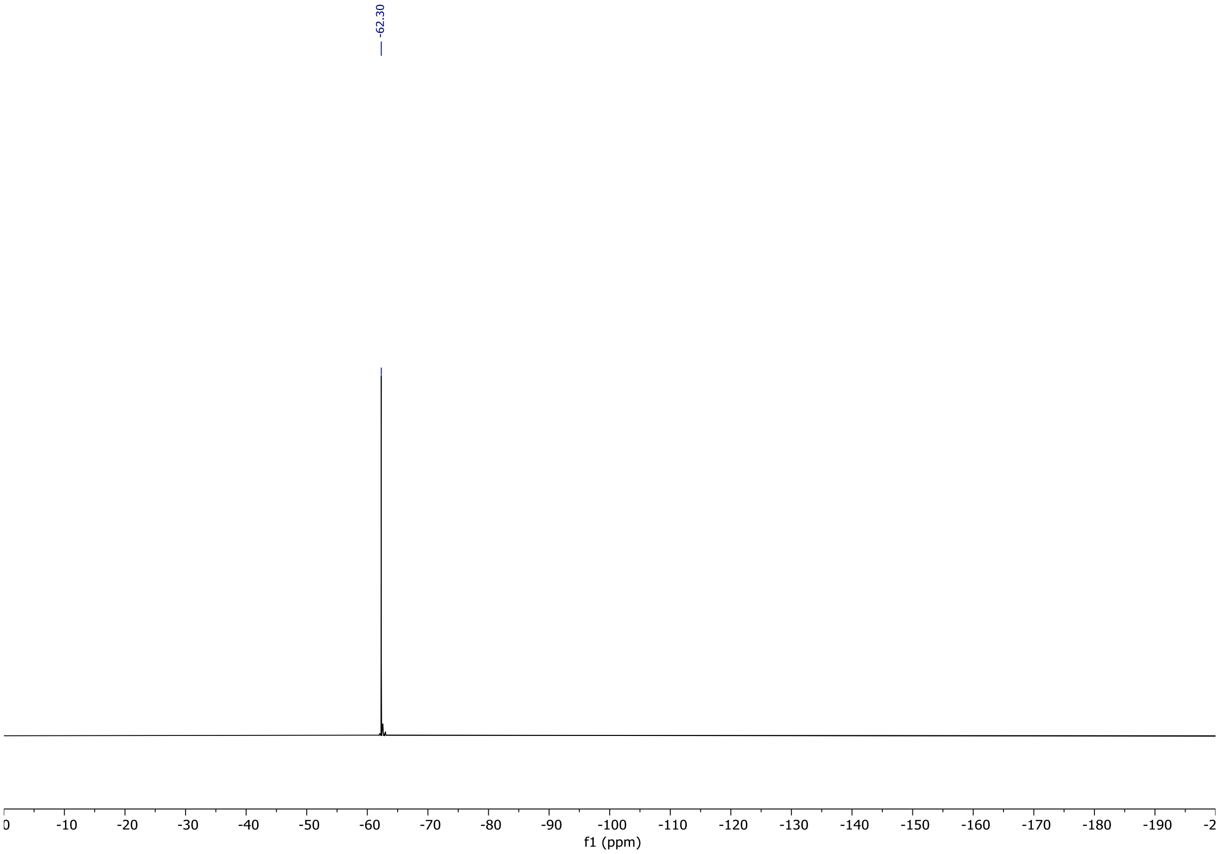

2,5-Di-*p*-tolyl-3,4-bis(4-(trifluoromethyl)phenyl)dibenzo[*a*,*k*][3,8]phenanthroline (5e)

^{1}H NMR (400 MHz, $CDCl_3$, 25 °C)

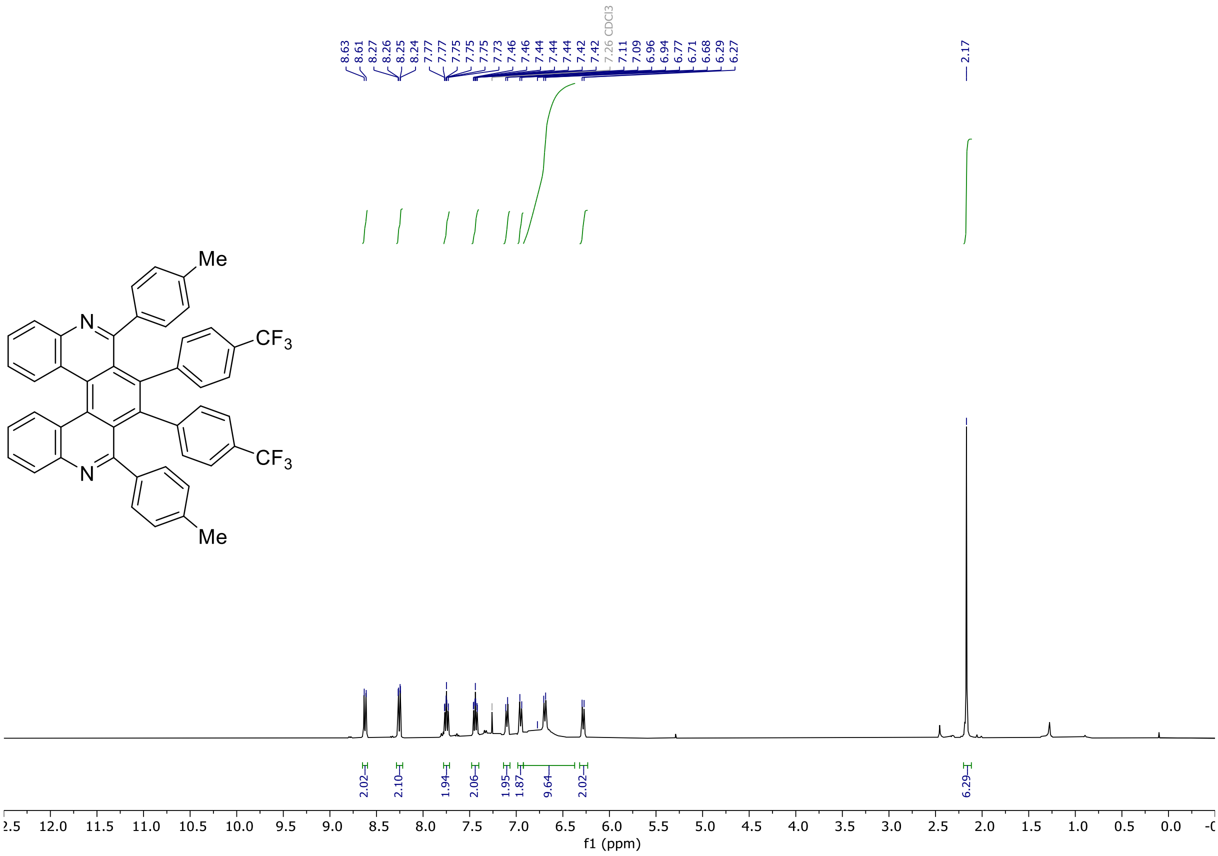

^{13}C NMR (101 MHz, $CDCl_3$, 25 °C)

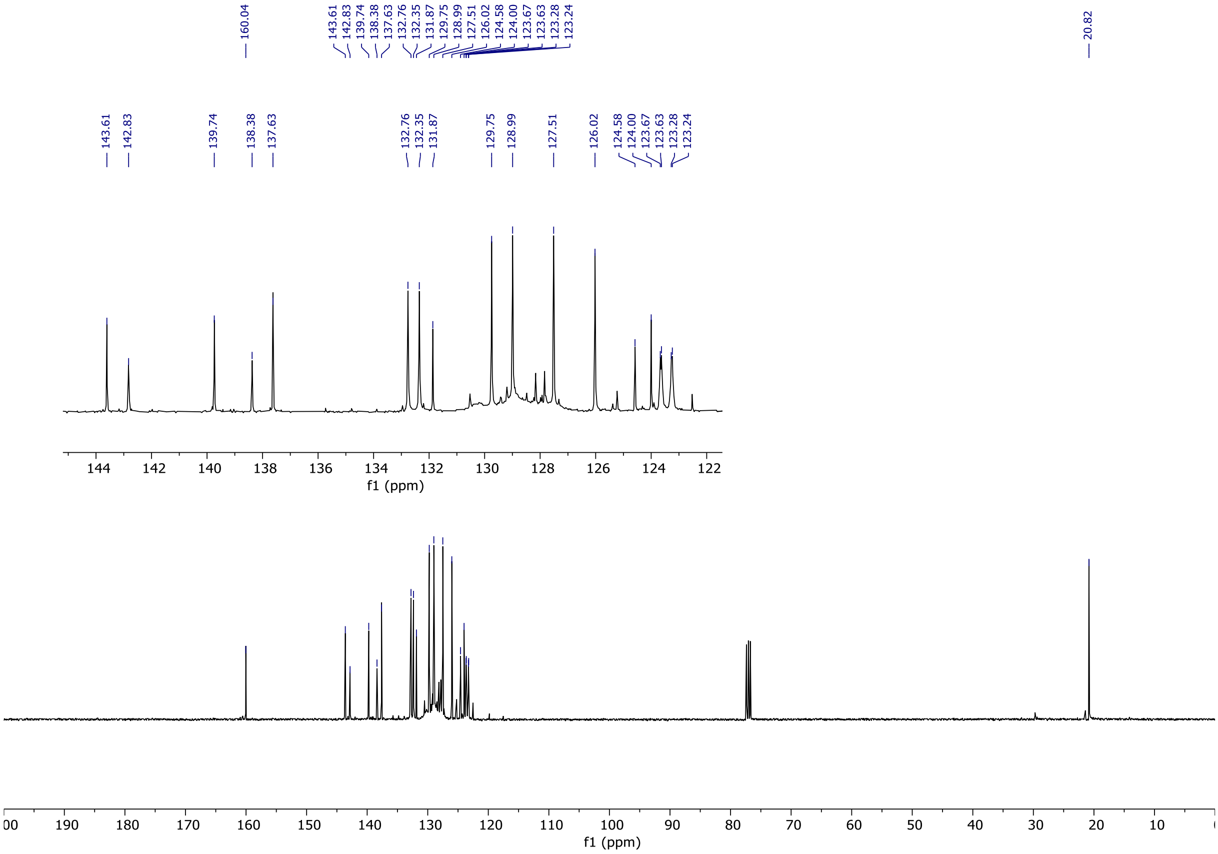

^{19}F NMR (376.5 MHz, $CDCl_3$, 25 °C)

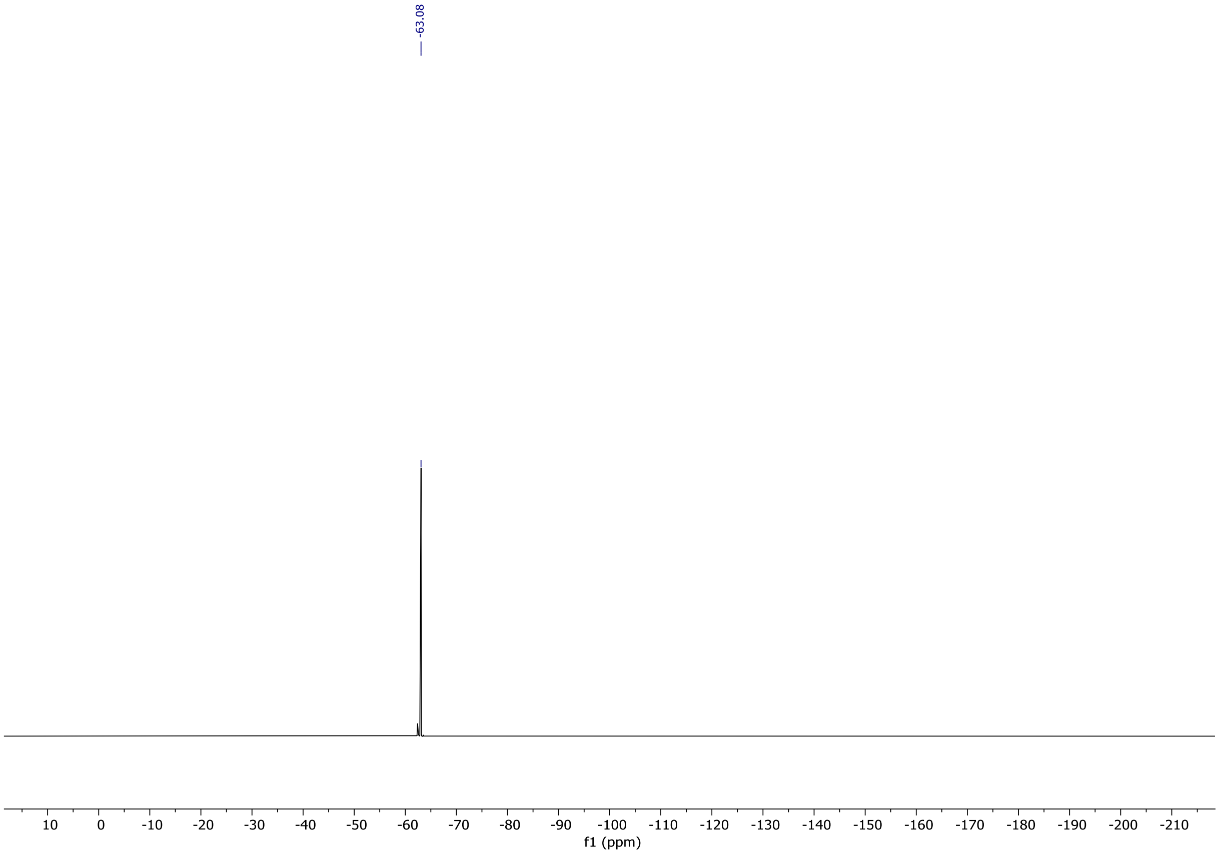

2,6-Di-*p*-tolyl-3,4-bis(4-(trifluoromethyl)phenyl)dibenzo[*a*,*k*][3,7]phenanthroline (6e in a mixture with **5e)**

^{1}H NMR (400 MHz, $CDCl_3$, 25 °C)

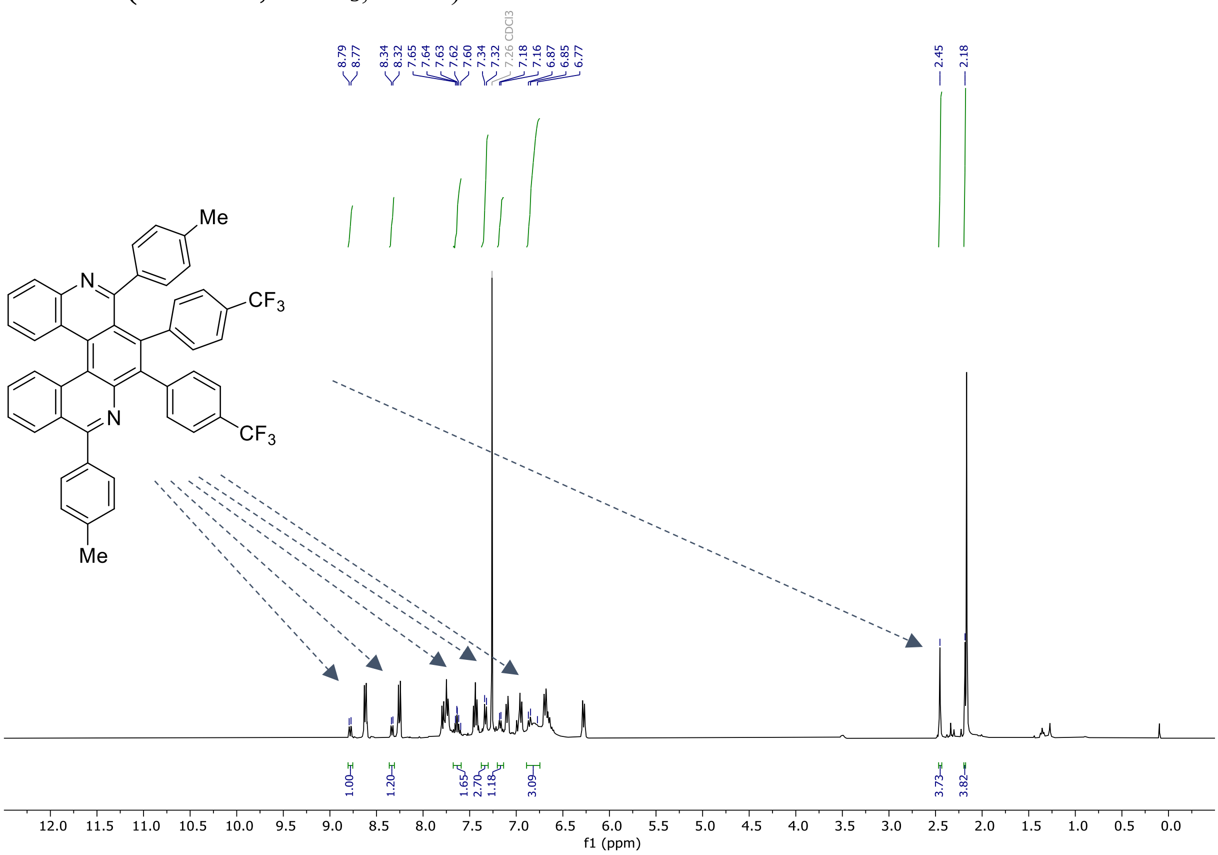

^{13}C NMR (101 MHz, $CDCl_3$, 25 °C)

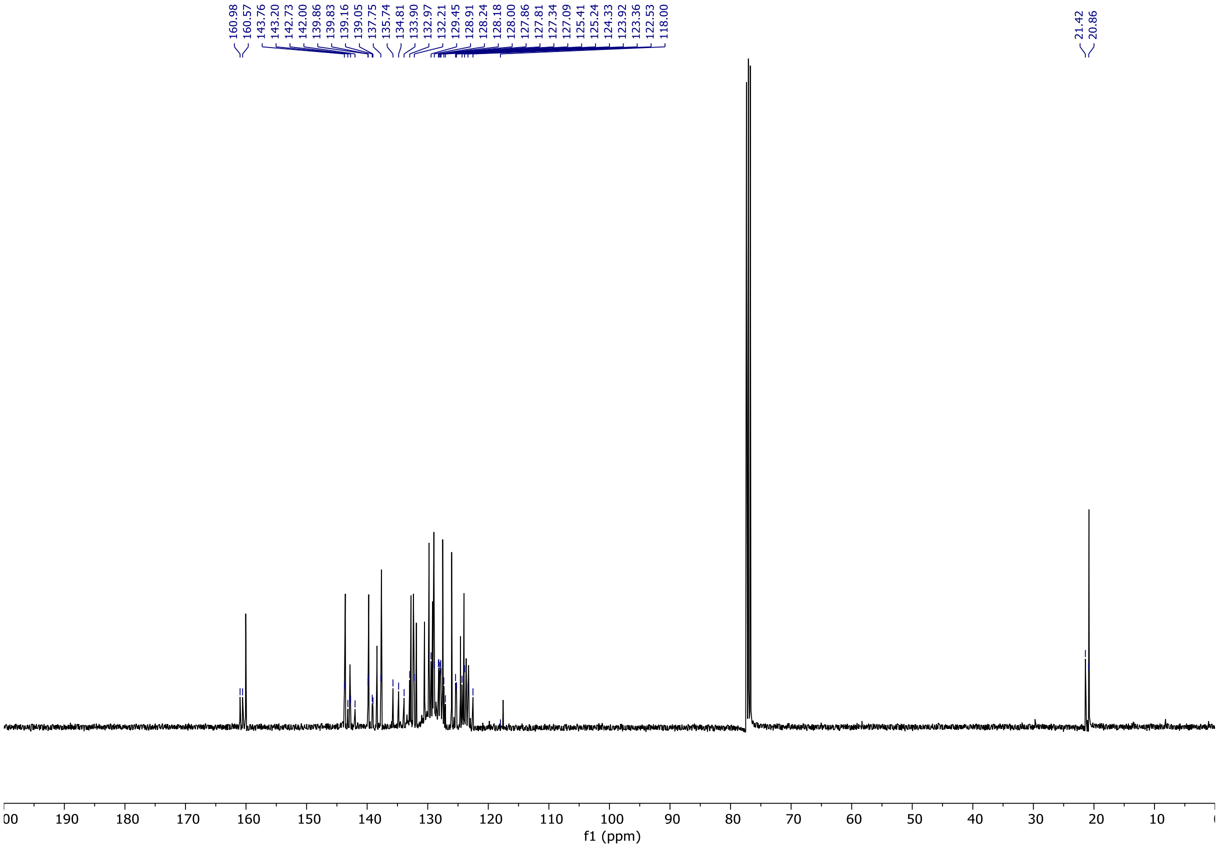

^{19}F NMR (376.5 MHz, $CDCl_3$, 25 °C)

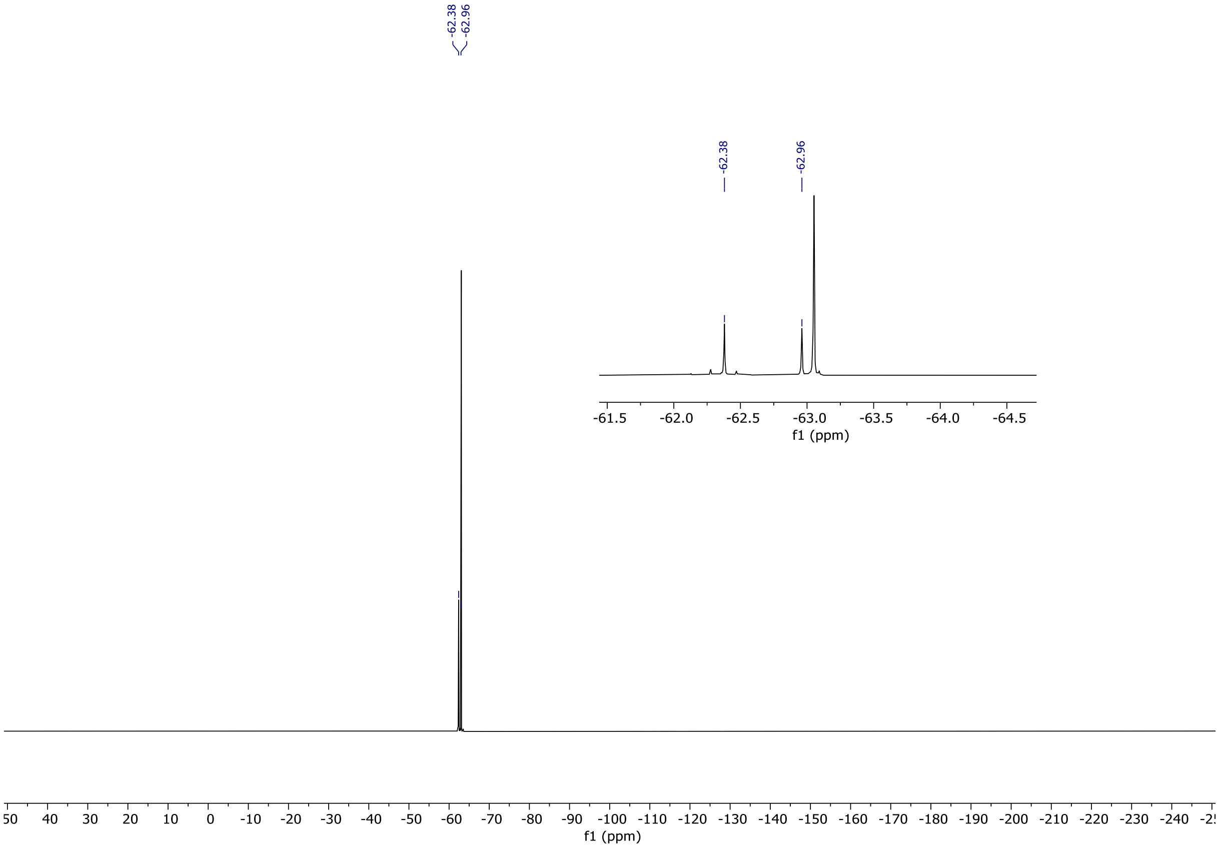

2,5-Di-*p*-tolyldibenzo[*a*,*k*][3,8]phenanthroline (5f)

^{1}H NMR (400 MHz, $CDCl_3$, 25 °C)

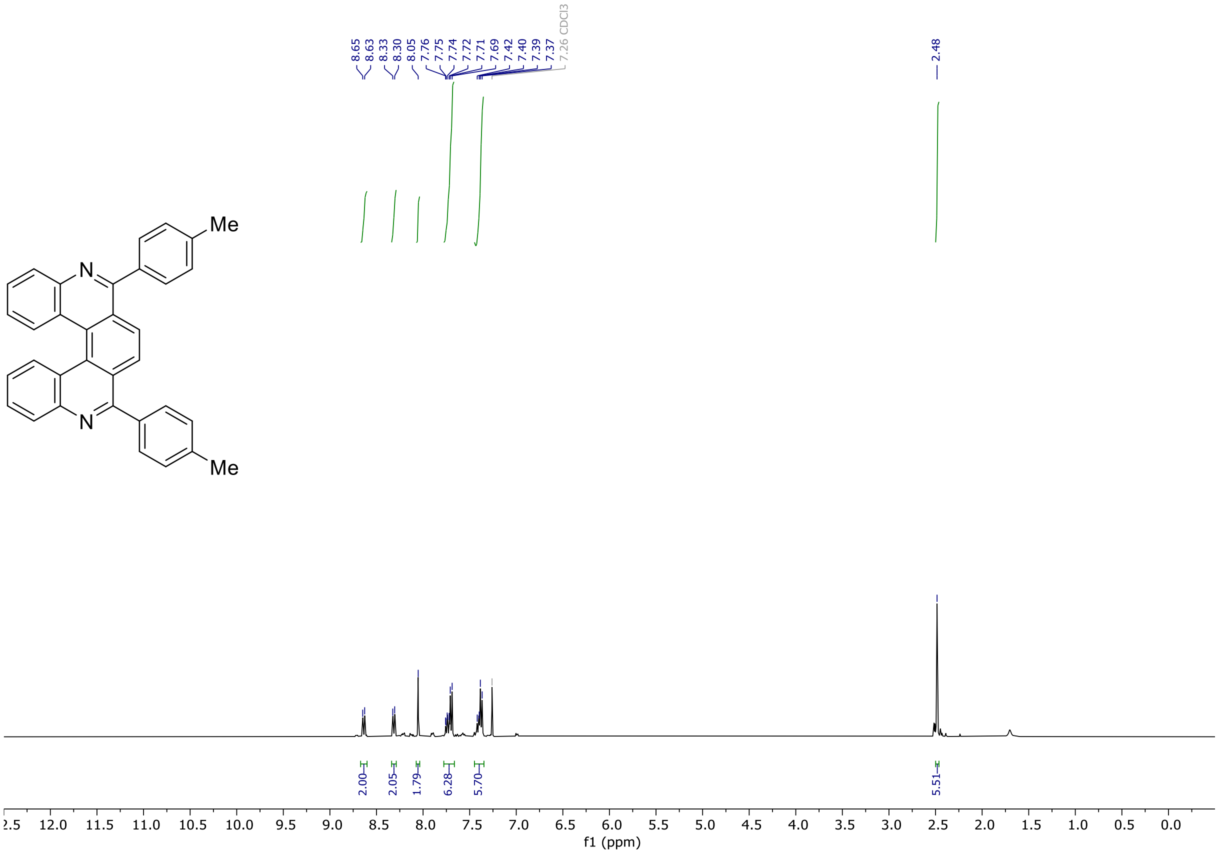

^{13}C NMR (101 MHz, $CDCl_3$, 25 °C)

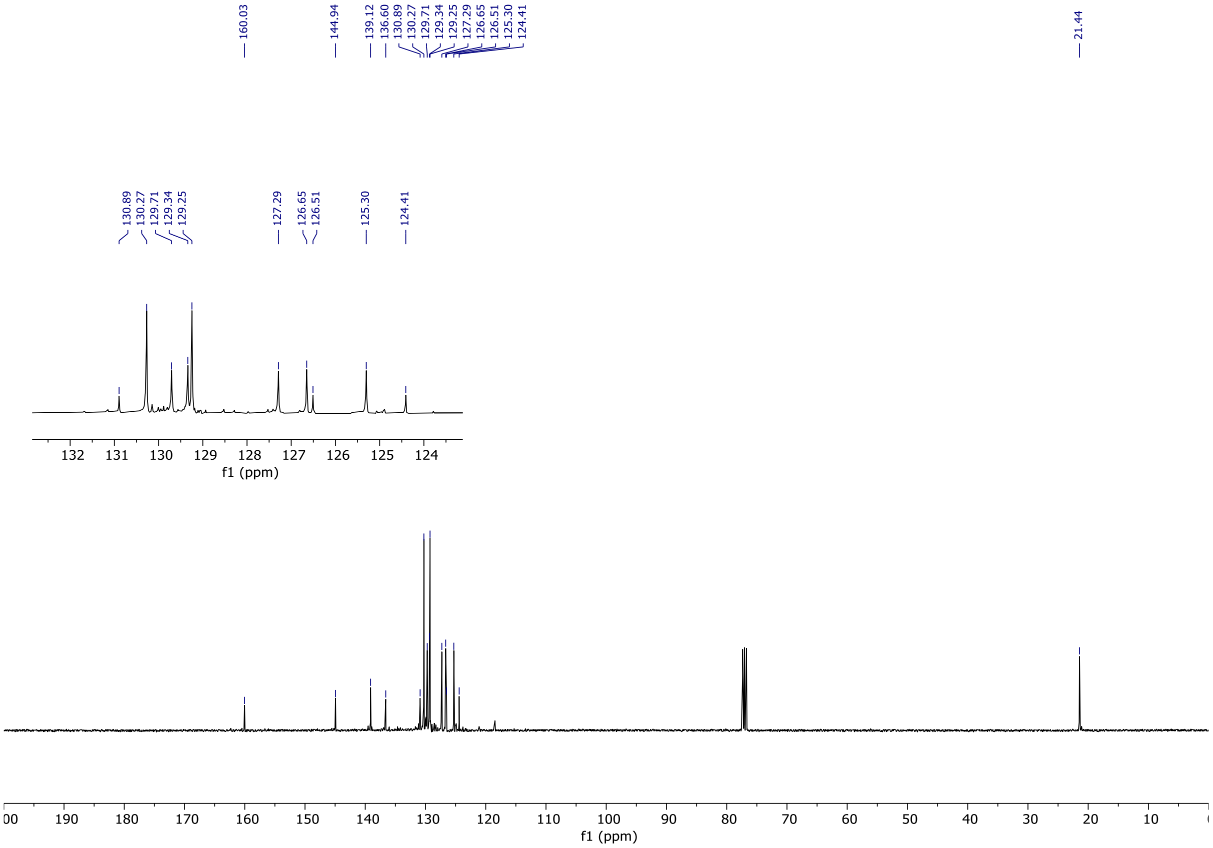

9,12-Dimethoxy-3,4-diphenyl-2,5-di-*p*-tolyldibenzo[*a*,*k*][3,8]phenanthroline (5g)

^{1}H NMR (400 MHz, $CDCl_3$, 25 °C)

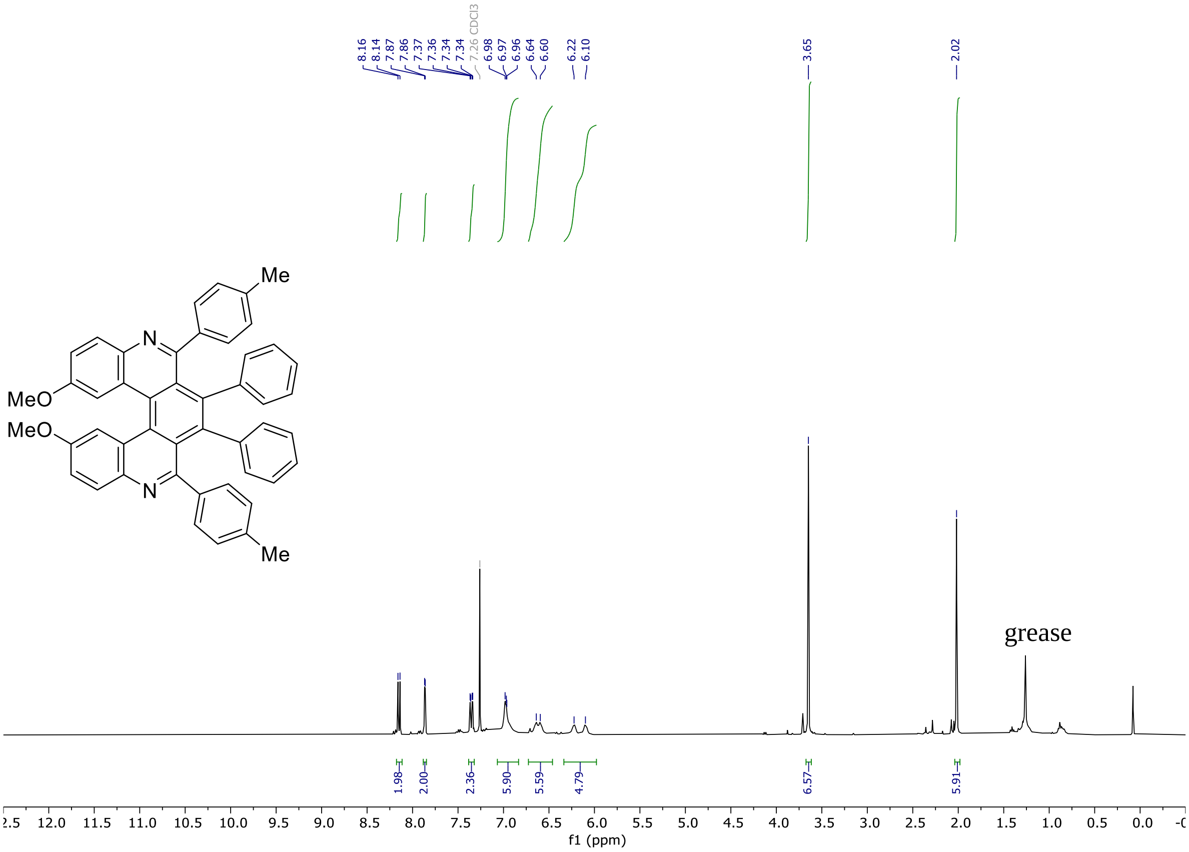

^{13}C NMR (101 MHz, $CDCl_3$, 25 °C)

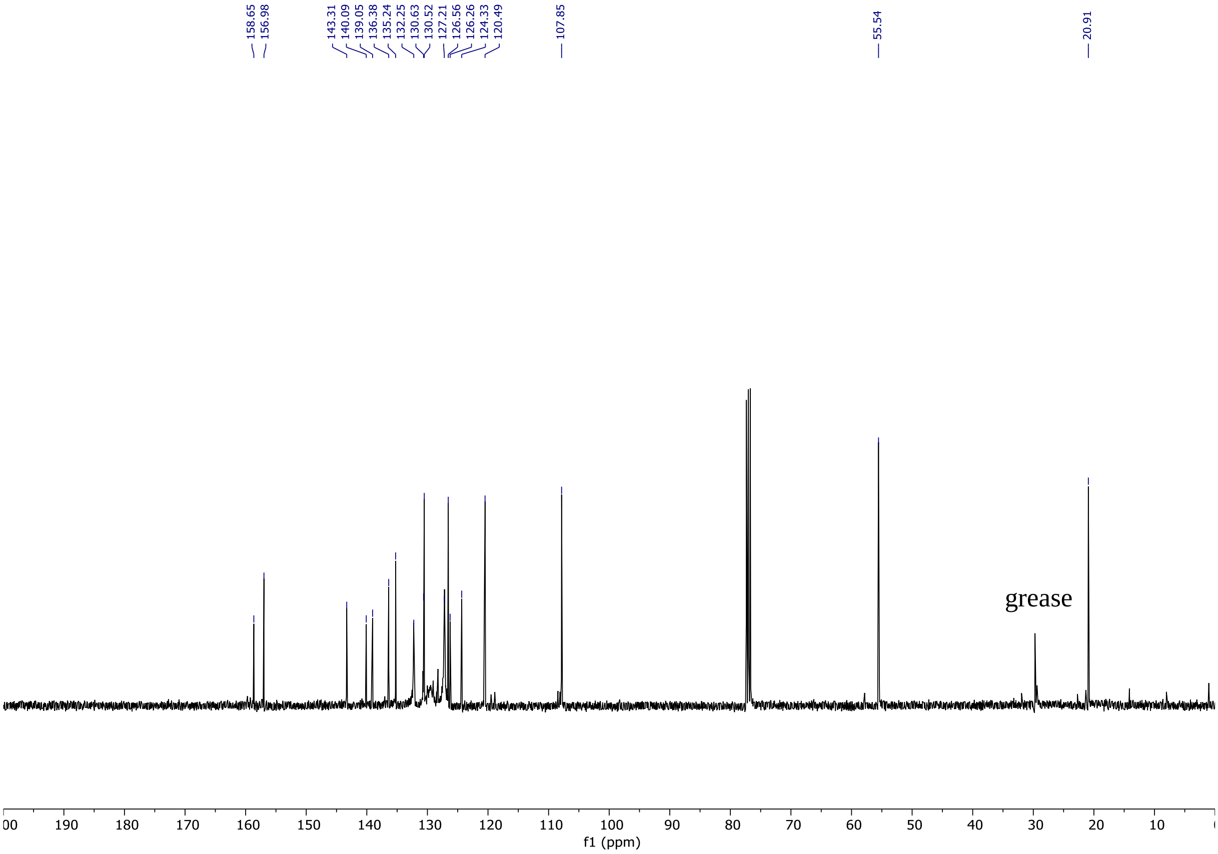

3,4-Bis(4-methoxyphenyl)-1-methyl-2,5-di-p-tolyldibenzo[a,k][3,8]phenanthrolin-1-ium iodide (5a$^+$)

^{1}H NMR (400 MHz, CDCl$_3$, 25 °C)

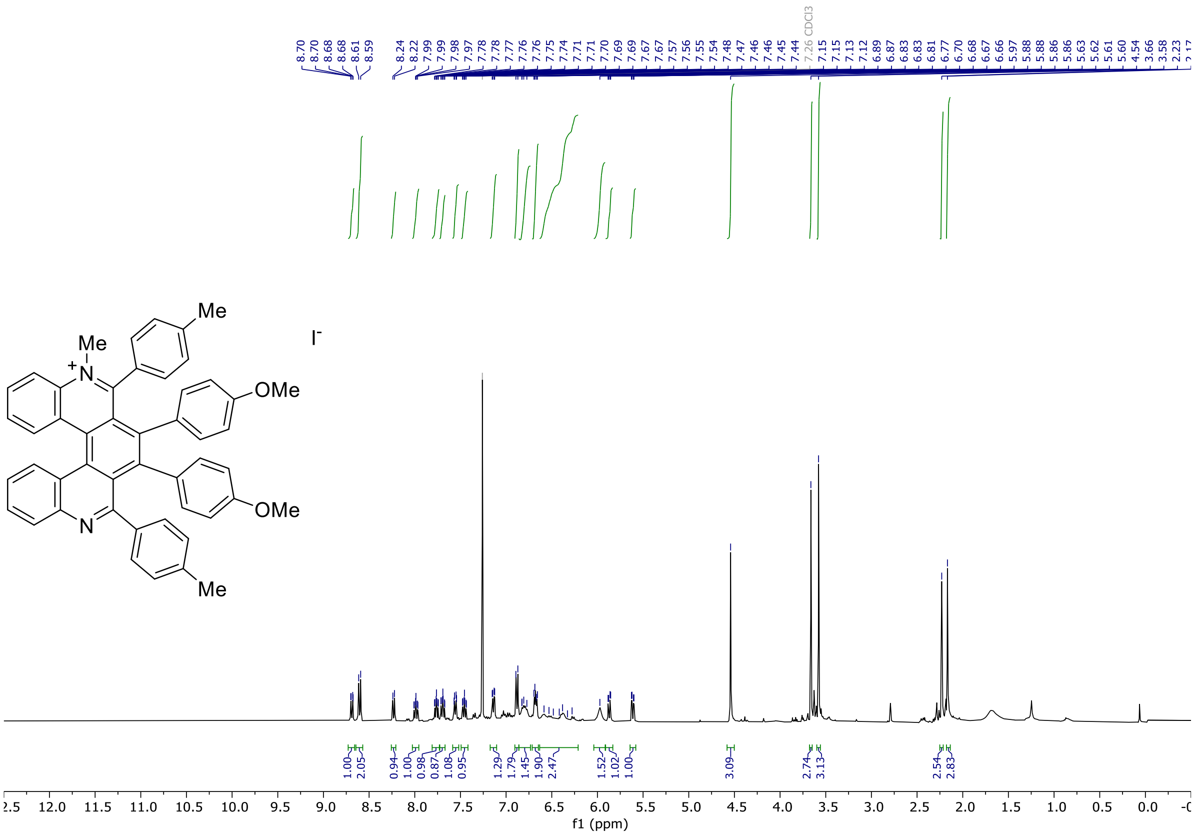

^{13}C NMR (101 MHz, CDCl$_3$, 25 °C)

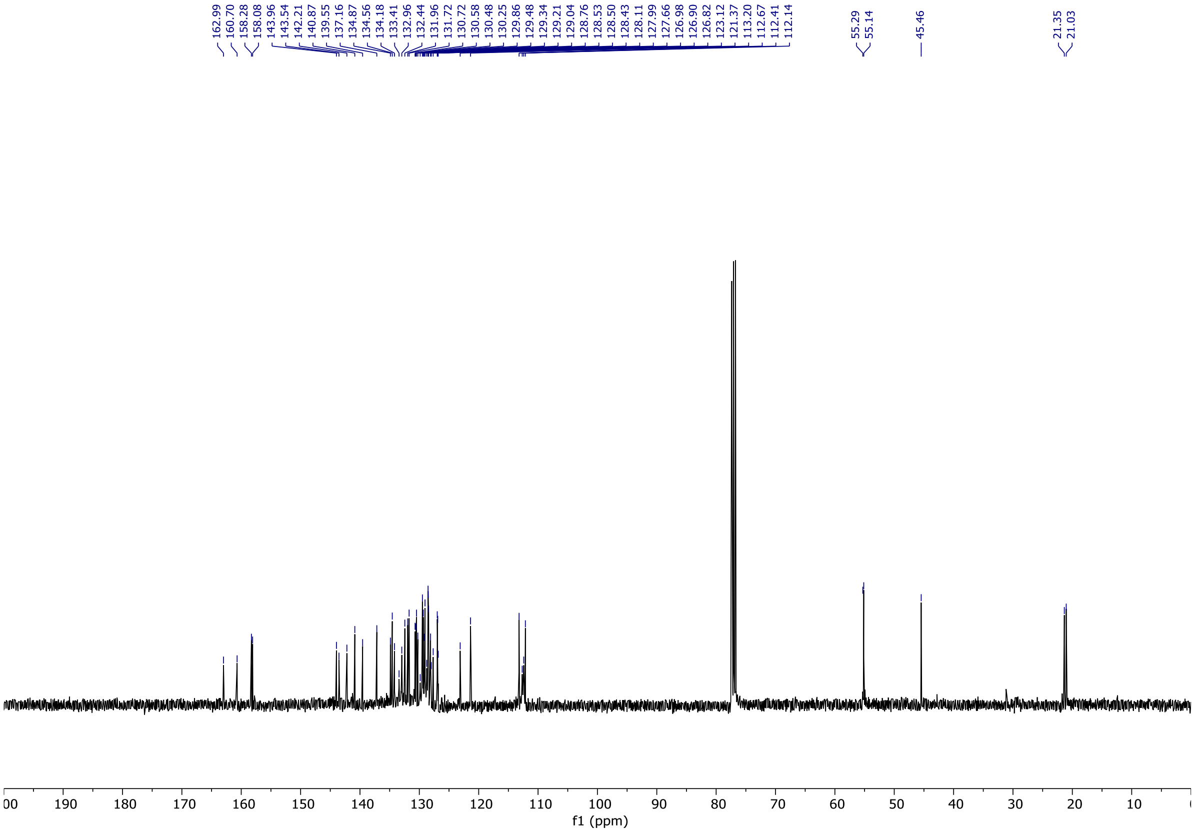

9,10-Bis(4-methoxyphenyl)-7,12-di-*p*-tolyldinaphtho[1,2-*a*:2',1'-*k*][4,7]phenanthroline (9)

^{1}H NMR (400 MHz, $CDCl_3$, 25 °C)

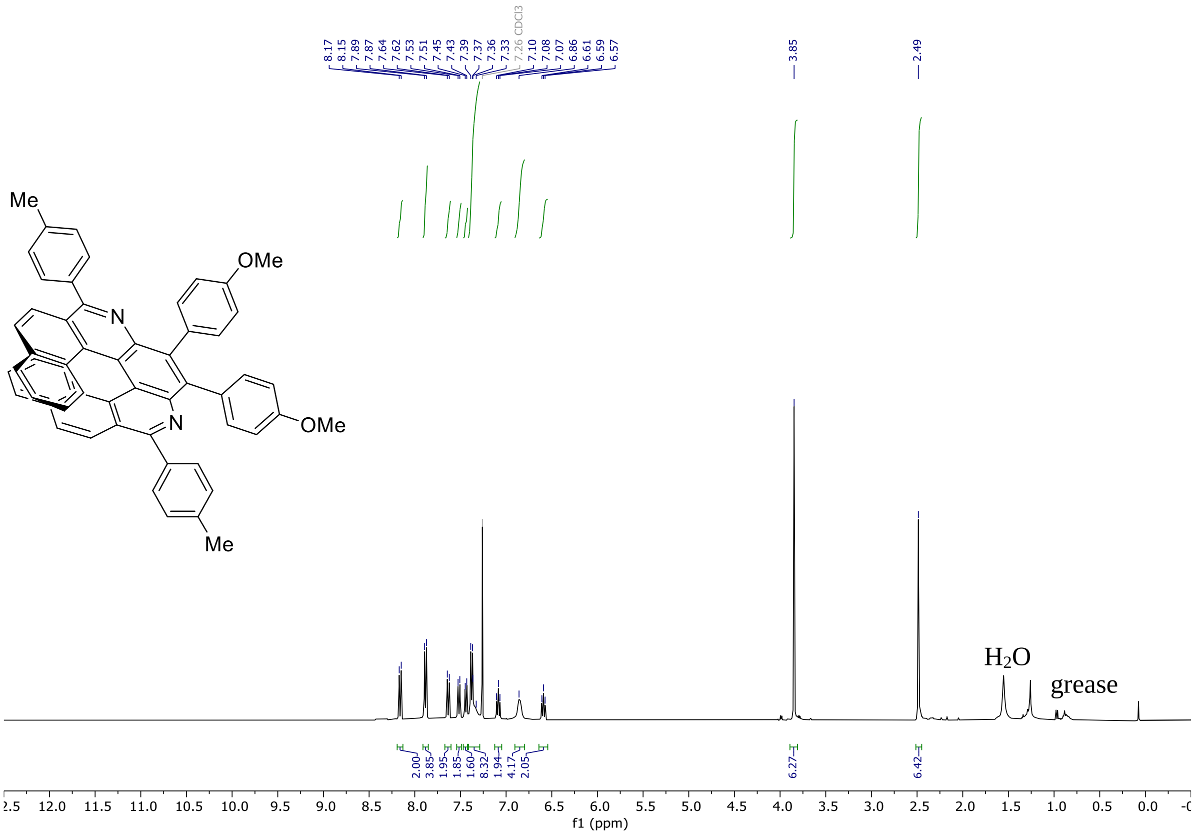

^{13}C NMR (101 MHz, $CDCl_3$, 25 °C)

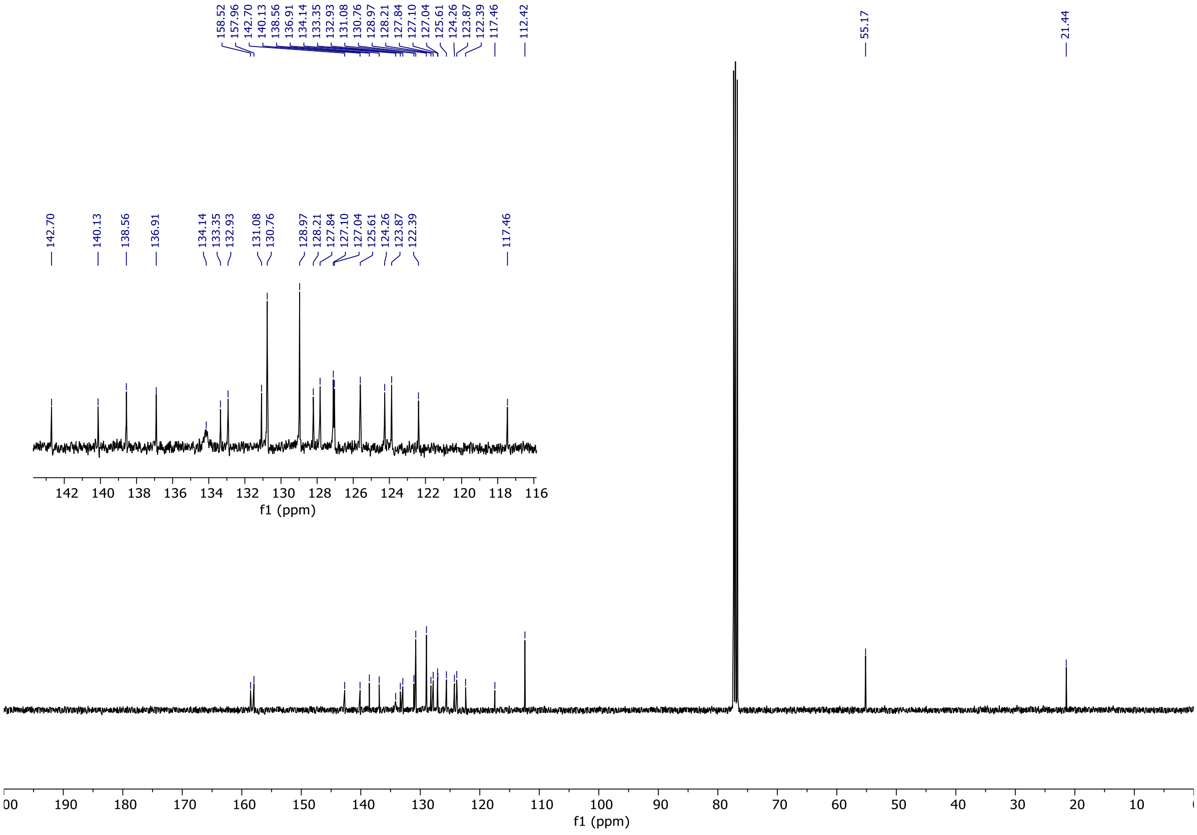

9,10-Bis(4-methoxyphenyl)-8,11-di-*p*-tolyldinaphtho[1,2-*a*:2',1'-*k*][3,8]phenanthroline (10)

^{1}H NMR (400 MHz, $CDCl_3$, 25 °C)

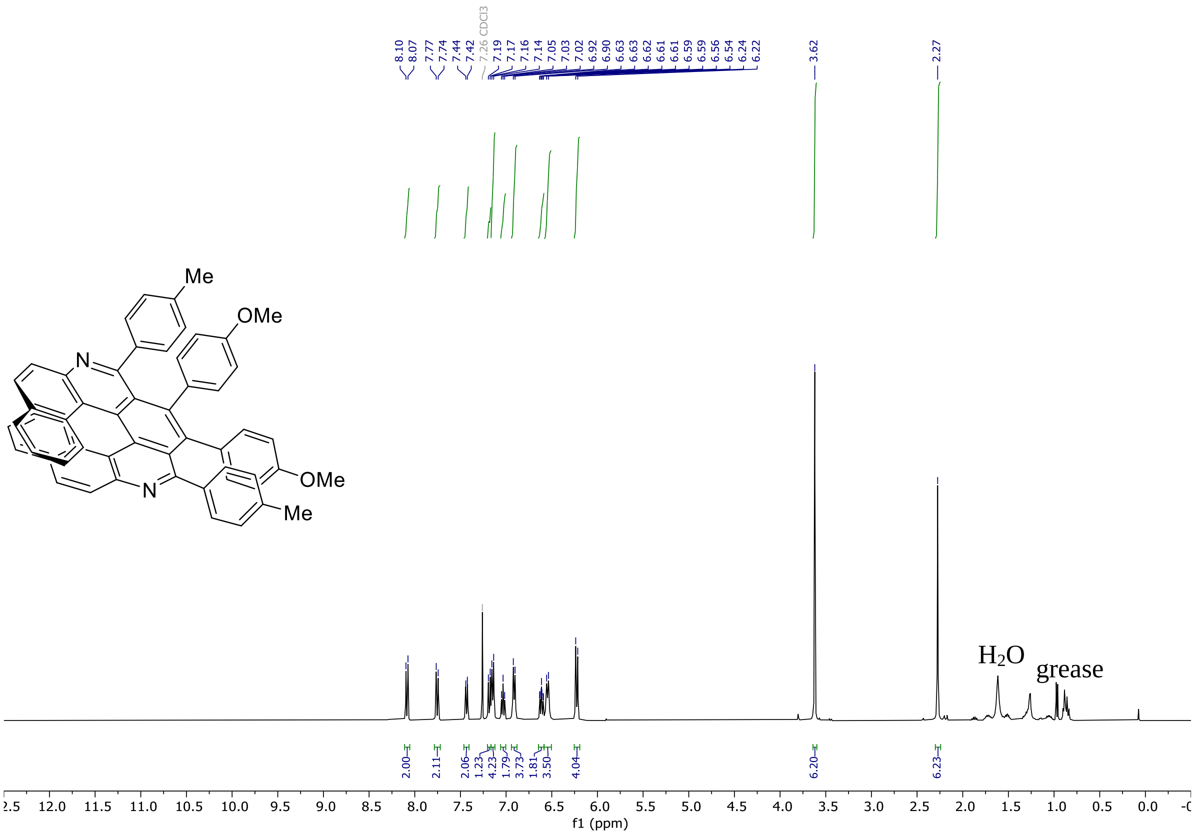

^{13}C NMR (101 MHz, $CDCl_3$, 25 °C)

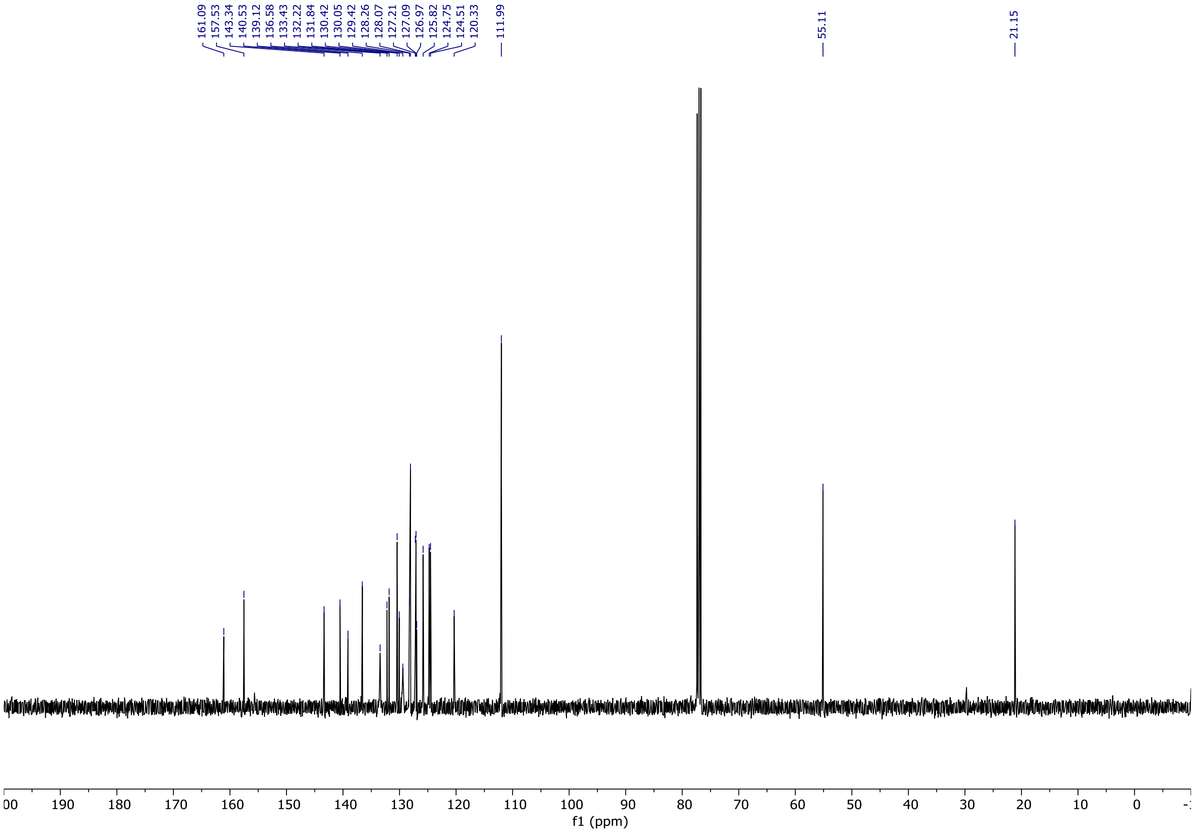

9,10-Bis(4-methoxyphenyl)-8,12-di-*p*-tolyldinaphtho[1,2-*a*:2',1'-*k*][3,7]phenanthroline (11)

^{1}H NMR (400 MHz, $CDCl_3$, 25 °C)

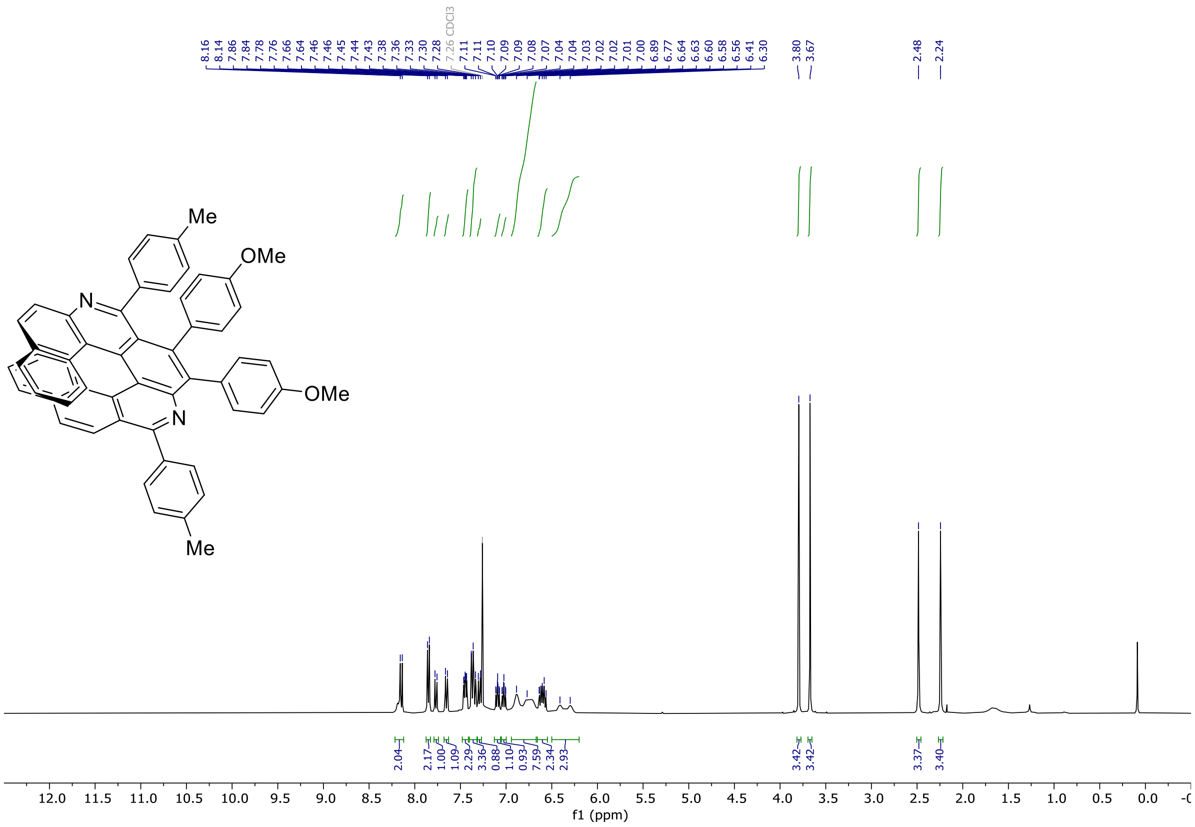

^{13}C NMR (101 MHz, $CDCl_3$, 25 °C)

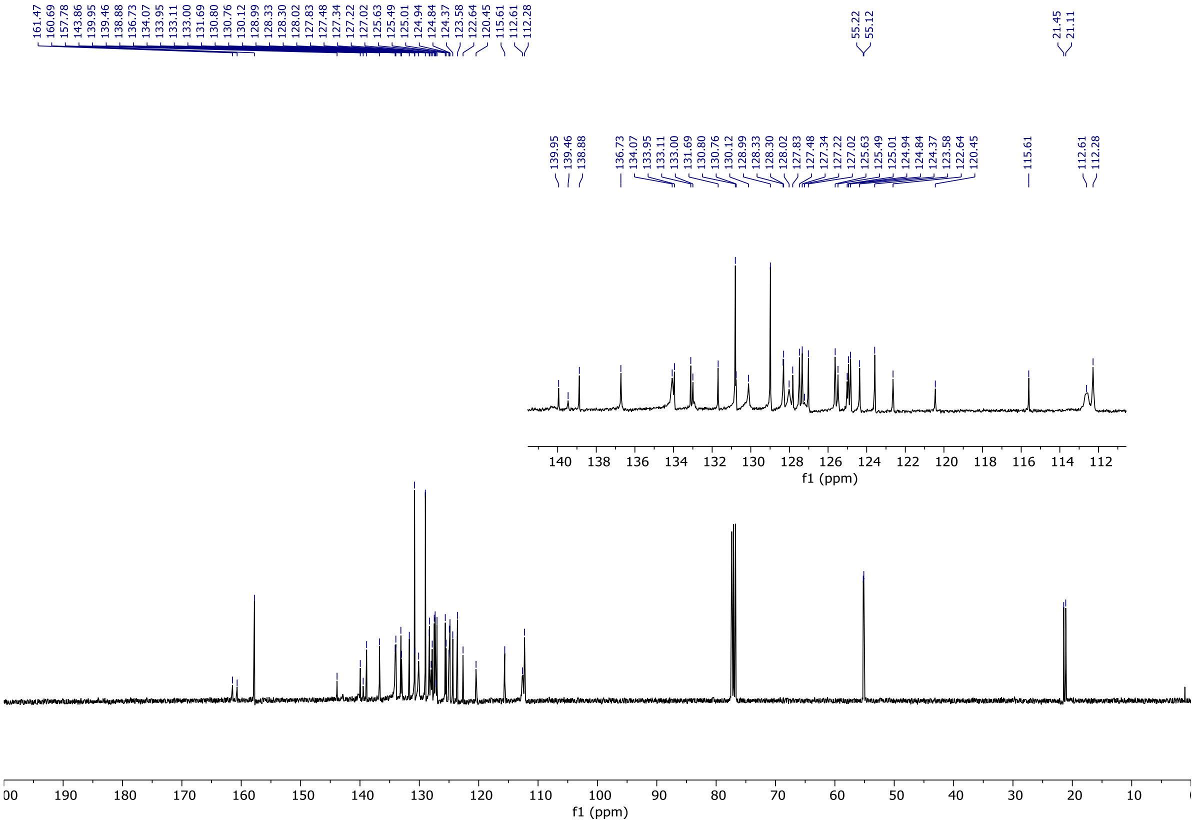

Supplement: Supplementary file 1 [file au5c00729_si_001.pdf]
